# “Mommy Blogs” and the Vaccination Exemption Narrative: Results From A Machine-Learning Approach for Story Aggregation on Parenting Social Media Sites

**DOI:** 10.2196/publichealth.6586

**Published:** 2016-11-22

**Authors:** Timothy R Tangherlini, Vwani Roychowdhury, Beth Glenn, Catherine M Crespi, Roja Bandari, Akshay Wadia, Misagh Falahi, Ehsan Ebrahimzadeh, Roshan Bastani

**Affiliations:** ^1^Center for Digital HumanitiesUniversity of California, Los AngelesLos Angeles, CAUnited States; ^2^Department of Electrical EngineeringUniversity of California, Los AngelesLos Angeles, CAUnited States; ^3^Fielding School of Public Health, Jonsson Comprehensive Cancer CenterUCLA Kaiser Permanente Center for Health EquityUniversity of California, Los AngelesLos Angeles, CAUnited States

**Keywords:** vaccination, social media, machine learning, personal narratives, Internet, health knowledge, attitudes, practice

## Abstract

**Background:**

Social media offer an unprecedented opportunity to explore how people talk about health care at a very large scale. Numerous studies have shown the importance of websites with user forums for people seeking information related to health. Parents turn to some of these sites, colloquially referred to as “mommy blogs,” to share concerns about children’s health care, including vaccination. Although substantial work has considered the role of social media, particularly Twitter, in discussions of vaccination and other health care–related issues, there has been little work on describing the underlying structure of these discussions and the role of persuasive storytelling, particularly on sites with no limits on post length. Understanding the role of persuasive storytelling at Internet scale provides useful insight into how people discuss vaccinations, including exemption-seeking behavior, which has been tied to a recent diminution of herd immunity in some communities.

**Objective:**

To develop an automated and scalable machine-learning method for story aggregation on social media sites dedicated to discussions of parenting. We wanted to discover the aggregate narrative frameworks to which individuals, through their exchange of experiences and commentary, contribute over time in a particular topic domain. We also wanted to characterize temporal trends in these narrative frameworks on the sites over the study period.

**Methods:**

To ensure that our data capture long-term discussions and not short-term reactions to recent events, we developed a dataset of 1.99 million posts contributed by 40,056 users and viewed 20.12 million times indexed from 2 parenting sites over a period of 105 months. Using probabilistic methods, we determined the topics of discussion on these parenting sites. We developed a generative statistical-mechanical narrative model to automatically extract the underlying stories and story fragments from millions of posts. We aggregated the stories into an overarching narrative framework graph. In our model, stories were represented as network graphs with actants as nodes and their various relationships as edges. We estimated the latent stories circulating on these sites by modeling the posts as a sampling of the hidden narrative framework graph. Temporal trends were examined based on monthly user-poststatistics.

**Results:**

We discovered that discussions of exemption from vaccination requirements are highly represented. We found a strong narrative framework related to exemption seeking and a culture of distrust of government and medical institutions. Various posts reinforced part of the narrative framework graph in which parents, medical professionals, and religious institutions emerged as key nodes, and exemption seeking emerged as an important edge. In the aggregate story, parents used religion or belief to acquire exemptions to protect their children from vaccines that are required by schools or government institutions, but (allegedly) cause adverse reactions such as autism, pain, compromised immunity, and even death. Although parents joined and left the discussion forums over time, discussions and stories about exemptions were persistent and robust to these membership changes.

**Conclusions:**

Analyzing parent forums about health care using an automated analytic approach, such as the one presented here, allows the detection of widespread narrative frameworks that structure and inform discussions. In most vaccination stories from the sites we analyzed, it is taken for granted that vaccines and not vaccine preventable diseases (VPDs) pose a threat to children. Because vaccines are seen as a threat, parents focus on sharing successful strategies for avoiding them, with exemption being the foremost among these strategies. When new parents join such sites, they may be exposed to this endemic narrative framework in the threads they read and to which they contribute, which may influence their health care decision making.

## Introduction

Over the past decade and a half, the explosion in social media and the concomitant rise in informational websites has changed the manner in which people access health care information [1-4]. Various sites dedicated to conversations about child rearing and parenting, colloquially referred to as “mommy blogs,” attract millions of users [3,5]. Although straightforward data mining techniques such as topic modeling exist for determining *what* parents are talking about on these sites and other similar sites, few techniques exist for determining *how* they are talking about those topics.

Among the many topics discussed on these parenting sites, few topics garner as much attention and vigorous discussion as childhood vaccination. Despite the fact that safe and effective vaccines exist, sporadic outbreaks of vaccine preventable diseases (VPDs) point to the continuing tension between public programs intended to make these vaccinations easily accessible and broadly adapted and parents who resist vaccination based largely on ideological principles [6-9]. Reduced rates of vaccination have jeopardized the elimination of diseases that have been on the cusp of such elimination for decades and, as recent outbreaks attest, threaten the hard-won herd immunity developed through long-term vaccination programs [6,10]. The role of exemptions in precipitating outbreaks in vaccine-communicable disease is increasingly being considered, although little evidence is currently available to directly support this link [11].

Although simple inspection of parenting sites and standard text mining approaches can confirm that vaccination is a topic of frequent discussion on these sites, such methods cannot determine the structure of those discussions. This is the objective of our research.

## Methods

### Introduction

**Figure 1:**
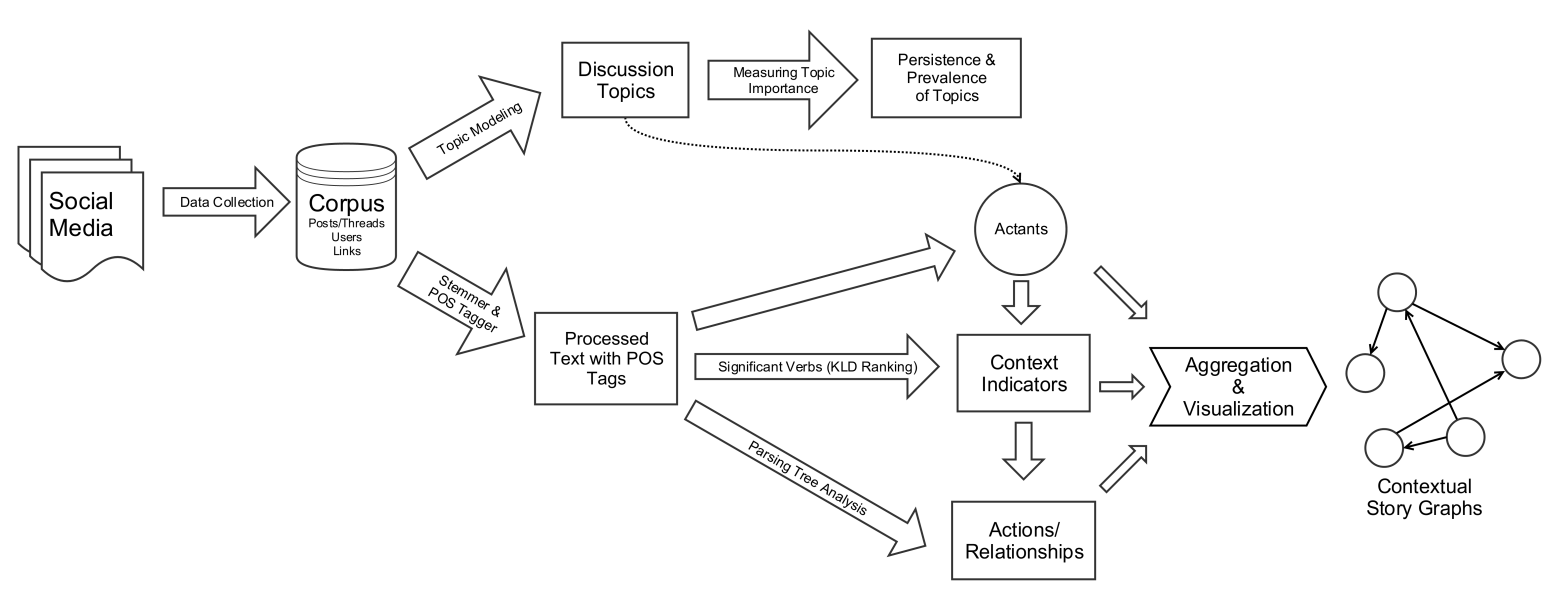
Workflow for aggregate narrative framework discovery.

In this research, we analyzed 1.99 million posts contributed by 40,056 users and viewed 20.12 million times indexed from 2 popular parenting sites over a period of 105 months ending in 2012. Beyond simply identifying the main topics of discussion on the sites, we discovered the underlying narrative frameworks that explain the stories circulating in these various discussions, an approach that extends recent work on personal experience and health knowledge exchange in Internet forums [12-16]. In addition to delineating the narrative framework that parents activate in their storytelling, we provided a fine-grained view of actant interactions and relationships in these stories, offering insight into individuals’ shifting attitudes toward vaccination. Figure 1 shows the pipeline describing the steps of this workflow.

Data for this study were obtained from 2 popular social media sites dedicated to parenting. We chose these 2 sites because of their popularity among new parents, with a membership comprised primarily of people who self-identify as mothers [2,17]. As mothers are on the “frontline” of discussions about the health of their infant children, these sites offer important information about how they approach decisions related to vaccination [18]. Although the second site has a more ideologically diverse group of active posters than the historically anti-vaccination mothering.com, both draw members from a wide range of backgrounds with broad geographic diversity, although largely from the United States and Canada. The language of both blogs is English. We indexed posts that appeared in forums related to childhood vaccination, recursively visiting and storing all publicly available discussion threads, and date-time–data, while creating an anonymized index of any accessible user data, resulting in a corpus of 299,778 posts from 12,376 users on mothering.com, based on 105 months of indexed data (2004-2012) and 1,700,086 posts from 27,790 users on a second site (unnamed due to terms of service), based on 60 months of indexed data (2008-2012) (UCLA IRB #16-000456). These posts comprised the corpus for analyses.

### Story Topics

For the 2 parenting sites, we determined the topics of discussion and the stories circulating in those discussions through an automated content analysis process. We started by computing dominant topics in the forums using 2 different probabilistic approaches, Latent Dirichlet Allocation (LDA) and Contextual Random Walk Traps (CRWT) [19,20]. LDA, a generative probabilistic topic modeling algorithm, proposes that each document in a corpus comprised consists of a mixture of a small number of topics, and that the appearance of a word in a document can be attributed to its membership in one of the document’s topics. CRWT creates a cooccurrence network for all words in the corpus and then decomposes this network into a hierarchy of random-walk traps. Each such random-walk trap, comprising a series of document vocabularies (bag of words) weighted by their respective steady-state probabilities, serves as a topic in the CRWT. In both cases, we considered each thread (comprising a set of posts by different users) as 1 document. We use topic modeling solely to discover topics of discussion on these websites and derive ranked lists of nouns.

### Stories

To understand how people talked about the discovered topics, we developed a story model, the actant-relationship context model, and used it to extract the underlying stories from posts across the entire set of 1.99 million discussion posts, recognizing that forum posts frequently include only parts of stories or comments on story parts as opposed to complete stories. We conceptualize story parts as relationships among actants [21]. These relationships map well onto the vaccine story motifs described by Kitta [22] and make frequent use of the tropes discovered by Kata [3]. We developed a generative statistical-mechanical network model, in which actants (actors and objects) are the nodes, and the relationships between nodes are the edges. The edges are labeled with the nature of the relationship, the context in which the relationship was found, and its likelihood. Actants and relationships are then aligned with a modified version of Labov’s 4-part structural map for personal experience narrative consisting of (1) orientation, where the community of interest is defined; (2) complicating action: threat, where something threatens the community delimited in the orientation; (3) complicating action: strategy, where the actants in the story devise a strategy to deal with the threat; and (4) resolution, where the outcome of using the proposed strategy to thwart the threat is reported [23,24].

In our model, to generate a social media post, a user picks a set of actants and draws from the distribution of relationships among those actants. The user then composes the post according to the outcomes in the first step. In a social media corpus, the underlying probabilistic model including both the primary actants and their contextual relationships is hidden. Consequently, our task was to estimate this hidden model from the posts. We accomplished this through a computationally scalable estimation algorithm that requires minimal supervision. Because the data were large scale and the story signals were persistent, we found that a computationally scalable inference algorithm using minimal information (such as nouns and verbs) from Natural Language Processing (NLP) tools gave us accurate results for our dataset.

#### Actants

We used the automatically discovered topics to determine the important actants in the topic space, recognizing that topics could cut across the siloes of forum classifications. To do so, we extracted a pool of actant terms based on a ranked list of high-frequency nouns. These nouns were, in turn, aggregated to derive actant categories. In topics associated with vaccination, we discovered 3 main categories of actants: individual actants, comprising parents, children, and medical professionals; institutional actants, comprising government institutions, religious institutions, pharmaceutical companies, and schools; and objects, comprising vaccines, exemptions, VPDs, and adverse effects. The words associated with an actant consist of both synonyms for the actant and entities that have the actant as a super-category. For example, the actant “government” includes the colloquial synonym “the Feds” as well as the government institution, the “CDC,” where “government” is the super-category for CDC.

#### Actant⇔Actant Contexts

We characterized the context between a pair of actants by a set of verbs that are significant when the 2 actants are discussed simultaneously. Verbs are known to capture binary relationships in large-scale corpora [25]. The contexts defined by verbs have discriminative power as they capture the different roles played by the same actants in different contexts.

In order to establish the significance of a verb for a particular pair of actants (ie, a context), we compared the conditional probability of the verb appearing with both actants to its marginal probability: A verb is *contextually significant* if P_pair_=Prob (verb **|**the sentence has both actants) >> P_corpus_=Prob (verb in any sentence in the corpus). This approach attenuates the effect of commonly occurring verbs such as “has,” “is,” and “are” (for which P_pair_≈P_corpus_), while accentuating topical verbs that describe meaningful relationships between actants.

**Figure 2:**
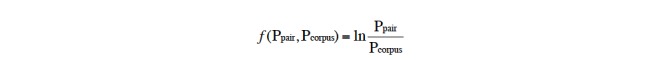
Calculation of the Kullback-Leibler divergence metric as a weighting function to rank the significance of different verbs.

As there are many verbs involved in any context, we ranked the relative significance of the different verbs via a scoring or weighting function *f* (P_pair_, P_corpus_), and then selected the top ones as the verb set to characterize the context. We empirically tested various scoring functions, including term frequency-inverse document frequency (TF-IDF) style scoring functions, and discovered that the Kullback–Leibler (KL) divergence metric (Figure 2), produced the best results [26]. Whereas the results are largely invariant to the particular choice of the ranking method, we found that KL divergence was better able to filter out noise such as the prevalence of modal and auxiliary verbs in the corpus. For any verb, the higher the KL score, the more significant that verb is to the pair.

**Figure 3:**
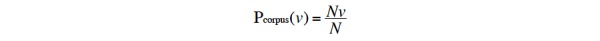
Calculation of the marginal probability of the verb appearing in any sentence in the corpus.

**Figure 4:**
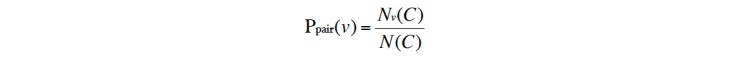
Calculation of the conditional probability of a verb appearing with both actants in a particular context.

**Figure 5:**
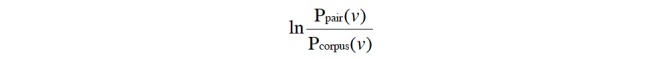
Calculation of ranking to determine the set of top verbs characterizing a given context.

To implement the above idea computationally, we tagged the entire corpus with parts of speech (POS) tags, using the Natural Language Toolkit (NLTK) library in Python [27]. As we extracted the verbs, we recorded their stemmed versions using the Porter stemmer in that toolkit. For example, “funded,” “funds,” and so on, were all recorded as the base form “fund.” For every stemmed verb, *v*, we calculated the marginal probability of the verb appearing in any sentence in the corpus (Figure 3), where *N*_v_ is the number of times verb *v* occurred in the corpus, and *N* is the sum of the frequencies of all the verbs in the corpus. Then, for any given context, defined as the set of all sentences where the 2 actants cooccur, we computed the conditional probability of a verb appearing with both actants in a particular context (Figure 4), where *N*_v_*(C* *)* is the number of times verb *v* occurred in the given context, and *N(* *C* *)* is the sum of the frequencies of all the verbs in the context. Then we computed the ranking to determine the set of top verbs characterizing a given context (Figure 5) for all verbs and ranked them in decreasing order to obtain the set of top verbs that characterized the given context.

#### Actant⇔Actant Relationships

Once we had determined the ranked verb list for a context, we returned to the sentences for that context and determined the actant pairs that these significant verbs related using the POS tagger output. Recognizing that different verbs may capture the same type of relationship between actants, we grouped verbs into “relationship” categories, just as we grouped nouns into actant categories. Taking a cue from the narrative theory, we classified these relationships according to a series of binary oppositions between verbs, with highly ranked synonyms grouped together with their highly ranked antonyms, allowing us to align those relationships to the structure of the personal experience narrative as well [28]. For readability, we devised labels for these groups of oppositional verbs.

We identified 2 main categories of binary opposite relationships. The first set of these relationships were those between individuals and institutional actants, with the binary oppositions *require or resist*, *advise or question*, *protect or threaten*, *employ or ignore*, *accept or reject*, and *attend or avoid*. The second set of relationships were those between individual and institutional actants on the one hand and objects on the other hand, or relationships between objects, with the binary oppositions *seek or aver*, *grant or withhold*, *cause or not cause*, and *protect or threaten*.

To illustrate this process, consider the verb “use” which was determined to be a significant verb in the Exemption**⇔**Religious-Institutions context (Table 1). In the following contextual sentence from a post that includes the actants *Exemption* and *Religious-Institutions*, we have highlighted the relevant words:

Here is some New York info: (sample exemption letters here) Here is info about how you do not have to prove membership in a church in order to use a religious exemption

The verb “use” relates “you” (which is a Parent actant) with “church” (which is a Religious Institutions actant). The category of the verbs that connects the 2 actants becomes the significant relationship between those actants in that context. For example, in the above case, as the verb “use” falls into the *employ or ignore* category, there is a directed *employ or ignore* edge from the Parents node to the Religious-Institutions node. We repeated this process for all possible contexts (we include additional examples of individual posts in the Multimedia Appendix 1).

#### Story Graphs

We visualized each context as a network story graph, with the actants as the nodes, and the significant relationships as the edges connecting the actants, thereby capturing the rich structures of relationships among actants for any context. We then create a summary graph by aggregating the story graphs for each context into a single graph. We label this summary graph a narrative framework.

#### Story Signal Trends

To characterize temporal trends in new posting activity that concerned vaccination exemptions and new user activity concerning exemptions, for each site, we calculated (1) the monthly proportion of new posts that included the word “exemption” and (2) the proportion of new users each month who committed a post with the word “exemption” in it. As users have access to old as well as new posts, in order to characterize the fraction of post content pertaining to exemptions that would be visible to users of the forums, we also calculated over the study period the monthly cumulative proportion of posts that included the word “exemption.” We produced a log-linear plot of the distribution of user-activity duration (in days) for the mothering.com site using a bin width of 3 months.

## Results

### Story Topics

On our 2 target sites, topic modeling revealed that vaccinations and, interestingly, exemptions constitute significant topics of discussion (for a full listing of topics, see the Multimedia Appendix 1).

We ran LDA topic modeling in R at multiple levels of granularity, from k=20 to k=200 in intervals of 20 (samples of the LDA topic models are included in the Multimedia Appendix 1) [29]. Past the topic parameter K=60, we found that topics, such as “exemption,” were largely split into multiple “exemption” topics. In the mothering.com forum, the “exemption” topic already emerged among the top topics with topic parameter, K=20: Topics 10 and 14 (Top 5 words): exemption school religious state required. Whereas the second site data presented a much larger set of forums and posts, the “exemption” topic nevertheless emerged in the top-60 list as a distinct topic (it did not appear as a distinct topic for K=20 or 40, but rather as a significant part of larger topics such as vaccination): Topic 46 (Top 5 words): state religious exemption child form.

**Figure 6:**
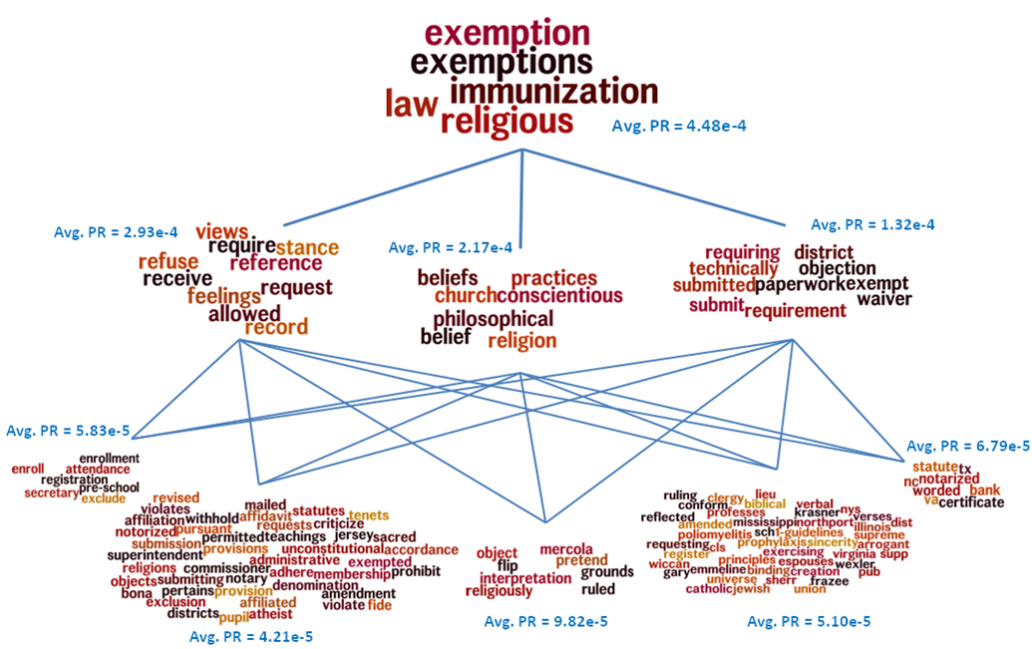
A hierarchical structure of topics related to exemption computed by the Contextual Random Walk Traps (CRWT) method from mothering.com posts. PR represents the page rank of the word-nodes in the co-occurrence network.

The CRWT method similarly yielded a more varied set of topics for the second site than for mothering.com, but the exemption-related topic was still distinct on both sites, constituted by some of the following words: “religious exemption beliefs exemptions belief belong supreme required.” As part of its output, CRWT yields a hierarchy of topics. For example, the “exemption” topic on mothering.com reveals a hierarchy where exemption is a super-category of “refusal,” “belief,” and “requirements,” as illustrated in Figure 6. These 2 topic modeling methods independently identify the importance of “exemption” as a topic of discussion in both forums at all levels of modeling that we used.

### Stories

The story model allows us to determine how people talk about the topics discovered through topic modeling. Recognizing that these topics can be discussed across the entire corpus, we do not assign documents to topics. Rather, we focus on discovering the underlying narrative framework, the activation of which in various posts, contributes to the structure of those discussions.

First, we determine the actants in the topic space (Table 1) and the relationship categories and associated verbs (Table 2). We find that the *Exemption*- and *Children*- related contexts are sufficient to derive a detailed understanding of the discussions and the stories that are embedded within them. The results for contexts based on exemptions (eg, exemptions and children) are shown in Table 3. The first column consists of the second actant in the relation, and the second column consists of the most significant verbs that occur in the related context. The stories clearly refer to families (orientation), with parents making health care decisions for their children. The verbs in the Parents**⇔**Exemption context reveal that parents try to acquire exemptions as a strategy (complicating action: strategy). The motivation for seeking exemptions becomes apparent when one examines the verbs in the Children**⇔**Vaccinations context (Table 4): seeking exemptions is a strategy to protect children from the (perceived) threat of vaccination (complicating action: threat). Results of the strategic use of exemptions vary from successfully securing exemptions to lamenting the inability to receive such exemptions.

**Figure 7:**
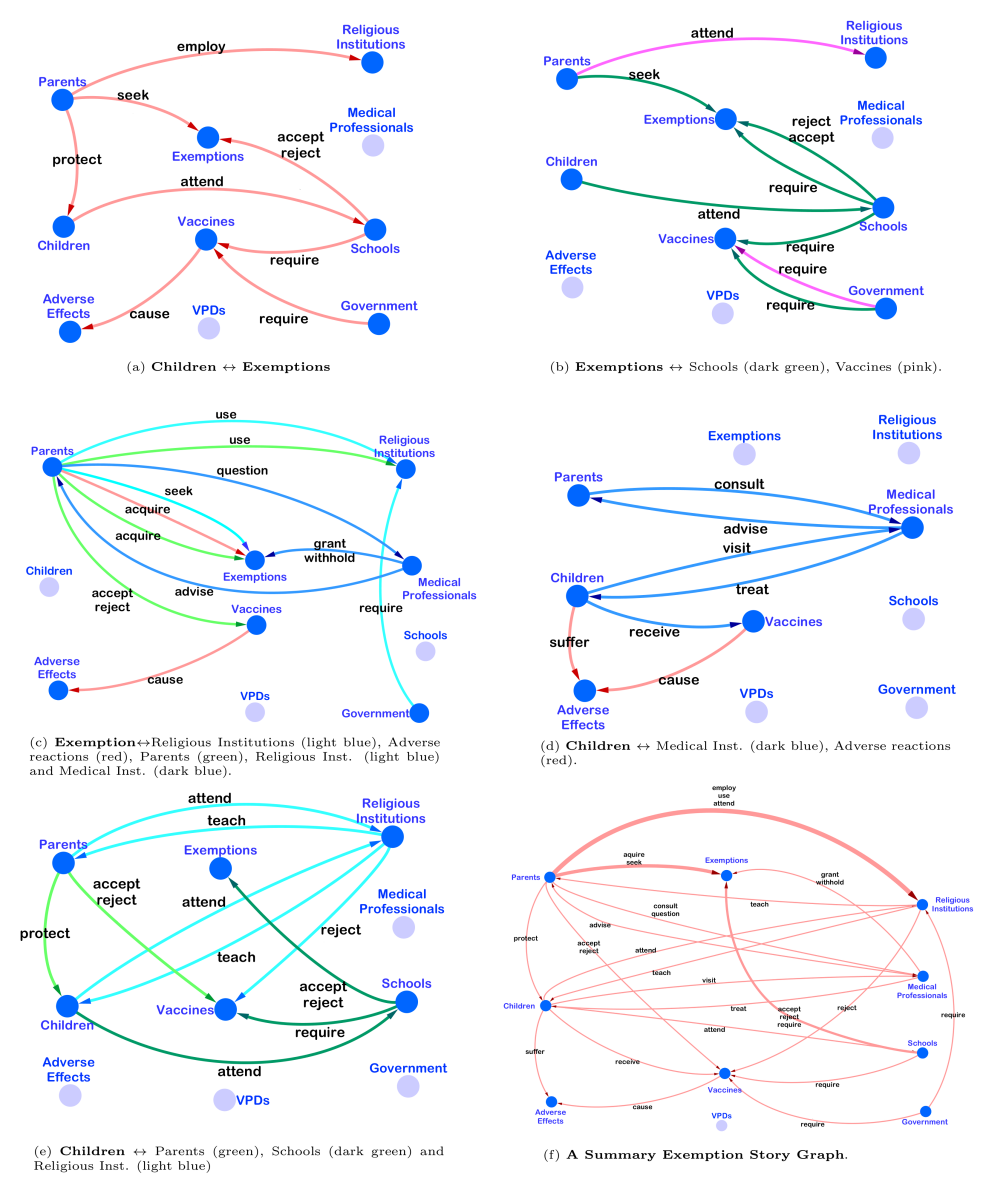
Story graphs and narratives: subfigures a-e illustrate the story graphs corresponding to different contexts in mothering.com, while subfigure f is an aggregate master narrative graph.

We illustrate these findings with story graphs for different contexts, aggregating these into a single narrative framework graph (Figure 7). Importantly, our approach recognizes that the relationships among actants can vary depending on the context. In the Children⇔Exemption story graph (Figure 7 a), one sees the central importance of exemptions for protecting children from required vaccines. The Exemption**⇔**Schools story graph (Figure 7 b) shows the role that schools and other government institutions play in requiring vaccines (dark green), establishing an adversarial relationship between parents on one hand, and government institutions on the other. In the Exemption**⇔**Religious Institutions story graph (Figure 7 c), an important relationship emerges between Religious Institutions and Parents (light blue), as we discover that parents and religious institutions are primarily connected on the basis of an *employ or ignore* relationship. This implies that parents use their affiliation with religious institutions (or the broader concept of faith) as a means to secure exemptions for their children. The contexts related to Children (Figure 7 d-e) further highlight the role that parents play in protecting their children from the adverse reactions that are allegedly caused by vaccination, as well as the role that affiliations to religious institutions can play in acquiring exemptions. In the Children**⇔**Religious-Institutions context, for instance, the relationship between parents and religious institutions changes to one of accept or reject, focused primarily on parents’ acceptance of church teachings.

**Table 1:** Actant model.

Entities (nodes)		Associated word set
**Individual actants**		
	Parents	parents, parent, i, we, us, you
	Children	child, kid, kids, children, daughter, daughters, son, sons, toddler, toddlers, kiddo, boy, d(ear)d(aughter), d(ear)s(on)
	Medical professionals	doctor, doctors, pediatrician, pediatricians, nurse, nurses, ped, md, dr
**Institutional actants**		
	Government	government, cdc, federal, feds, center for disease control, officials, politician, official, law
	Religious institutions	faith, religion, pastor, pastors, parish, parishes, church, churches, congregation, congregations, clergy
	Schools	teacher, teachers, preschools, preschool, school, schools, class, daycare, daycares, classes
	Pharmaceutical companies	pharma, big pharma, company, companies
**Objects**		
	Vaccines	vaccines, vax, vaccine, vaccination, vaccinations, shots, shot, vaxed, unvax, unvaxed, nonvaxed, vaccinate, vaccinated, vaxes, vaxing, vaccinating, substances, ingredients
	Exemptions	exemption, exempt
	VPDs^a^	varicella, chickenpox, flu, whooping cough, tetanus, pertussis, hepatitis, polio, mumps, measles, diphtheria
	Adverse effects	autism, autistic, fever, fevers, reaction, reactions, infection, infections, inflammation, inflammations, pain, pains, bleeding, bruising, diarrhea, diarrhea

^a^VPDs: vaccine preventable diseases.

**Table 2:** Relationship model.

Relationships (edges)		Associated word set (stemmed)
**Between individuals or institutional actants**		
	Require or resist	force, require, need, follow, mandate
	Advise or question	recommend, tell, said, object, ask, learn, teach
	Protect or threaten	protect, injure, damage
	Employ or ignore	use, submit, ignore
	Accept or reject	vaccinate, unvaccinate, vax, unvax, receive, have, had, get, inject, exclude, allow, exempt, believe, receive, request, deny, accept
	Attend or avoid	enter, enroll, attend, go, send, homeschool
**Between individual or institutional actants and objects or between objects**		
	Seek or aver	seek, file, sign, claim, submit, need, exercise, lie, claim
	Grant or withold	accept, approve, get, abuse, grant, oppose, deny
	Protect or threaten	protect, injure, damage
	Cause or not cause	expose, get, contract, cause, develop, suffer, die, vomit, diagnose

**Table 3:** Top 10 high-relevancy verbs (stemmed) that characterize the contexts comprising “Exemption” and each of the other major actant categories on the second site. The verbs are ordered according to the KL Divergence scores, but we have shown the frequency of the verbs in parenthesis for comparison. In the Exemption–Parent context, the verb “have” with a frequency count of 1561 is ranked fourth way before “exercise,” which has a frequency of only 275.

Actants in children context	Significant verbs in relationship to exemption (actant)
Parents	exempt(207), exercis(228), sign(275), have(1561), concern(196), claim(185), vaccin(241), belong(132), us(472), requir(199)
Children	exempt(220), exercis(191), concern(175), vaccin(202), vax(133), requir(152), sign(116), attend(99), enrol(56), allow(106)
Medical professionals	sign(101), exempt(21), give(72), write(34), requir(35), get(131), have(229), submit(16), obtain(17), file(17)
Government	bind(51), determin(51), requir(41), us(67), exempt(17), belong(22), accept(29), seek(19), furnish(8), obtain(12)
Religious institutions	belong(294), rule(114), offer(152), do(456), claim(105), us(191), have(445), find(149), bind(51), determin(50)
Schools	belong(146), rule(116), offer(160), requir(130), sign(120), attend(103), exempt(59), find(184), have(682), accept(104)
Vaccines	vaccin(377), exempt(173), requir(323), vax(252), claim(208), receiv(191), sign(150), request(106), allow(200), oppos(91)
VPDs^a^	requir(14), exempt(7), vaccin(15), sign(12), get(34), refus(9), have(55), prove(6), document(4), decid(8)
Adverse effects	had(64), exempt(12), obtain(12), requir(18), get(60), link(12), choose(9), follow(15), increas(11), qualifi(9)

^a^VPDs: vaccine preventable diseases.

**Table 4:** Top 10 high-relevancy verbs (stemmed) that characterize the contexts comprising “Children” and each of the other major actant categories on the second site. The verbs are ordered according to the KL Divergence scores, but we show the frequency of the verbs in parenthesis for comparison.

Actants in exemption context	Significant verbs in relationship to children (actant)
Parents	have(190359), give(27803), learn(18254), am(46173), choos(11446), want(46512), think(60272), rais(9756), know(61261), teach(8982)
Medical professionals	nurs(1728), vaccin(1450), told(1986), had(4091), said(2582), diagnos(893), take(2467), recommend(642), give(1782), took(926)
Government	vaccin(615), recommend(447), receiv(380), accord(291), mandat(144), ha(1211), caus(328), injur(139), report(214), includ(299)
Religious institutions	teach(2010), are(2873), is(3706), church(127), attend(221), go(937), rais(174), believ(335), allow(164), pray(73)
Schools	attend(2150), go(11284), ha(10885), send(2041), start(4659), work(4910), get(12171), daycar(621), need(5944), teach(1645), enrol(689)
Vaccines	vaccin(16262), vax(6176), receiv(3859), ha(8439), injur(1247), given(2321), unvaccin(930), caus(2315), recommend(1507), unvax(657), protect(1269)
VPDs^a^	vaccin(1409), receiv(1071), had(2622), get(2840), recommend(510), vax(450), given(590), develop(441), got(1005), expos(333)
Adverse effects	ha(17530), diagnos(4961), have(29879), had(9852), autism(1023), caus(2617), develop(1461), vaccin(1756), is(33778), affect(1064)

^a^VPDs: vaccine preventable diseases.

The summarized story graph (Figure 7 f), obtained by aggregating relationships across contexts, makes clear the underlying narrative framework. In the aggregate story, parents use religion or belief to acquire exemptions so as to protect their children from vaccines that are required by schools or government institutions, but (allegedly) cause adverse reactions such as autism, pain, compromised immunity, and even death.

The summarized story graph also reveals several notable substories. In one, religious institutions rather than schools play the role of “teacher.” In this substory, schools are relegated to the role of parental adversary, requiring vaccinations and wielding the power to accept or reject exemptions. In another substory, medical professionals play the role of the adversary. Parents question them over the necessity of vaccines, and resent them as the enforcers of vaccine requirements (threat). Yet, parents also need the medical professionals’ help, as they act as the grantors of exemptions (strategy). Two glaring omissions in this and all the substories on which the summary narrative framework graph is based are the near total absence of VPDs and pharmaceutical companies as actants. The only role that the VPDs play is a passive one: children contract them (see the penultimate row in Table 2). “Big pharma,” as pharmaceutical companies are often referred to, play no significant role in the contexts in which exemptions are discussed.

### Story Signal Trends

**Figure 8:**
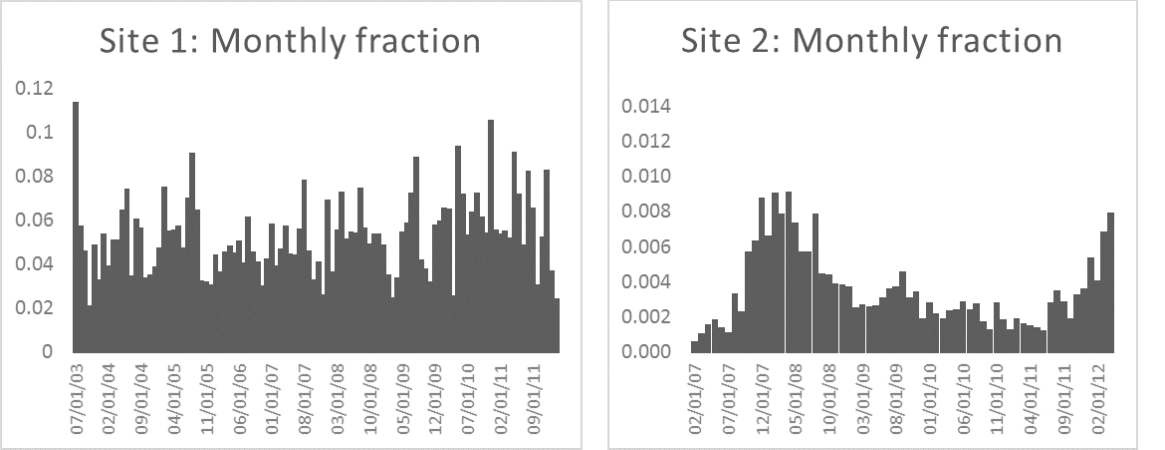
Monthly proportion of new posts that included the work “exemption” for the two sites.

Figure 8 shows the proportion of all new posts that included the word “exemption” by month for each site. On average about 5.32% of new posts on mothering.com included the word “exemption”; on the second site, the average was about 0.35%. The trends show that the exemption topic signal exhibited some variation over time but was not “bursty,” and there was some level of activity in all months, especially on mothering.com.

**Figure 9:**
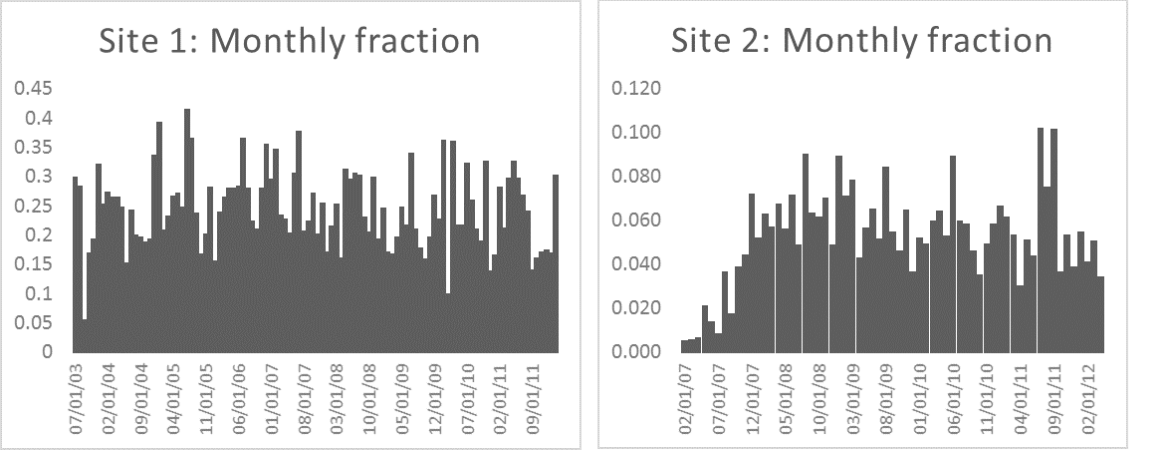
Monthly proportion of new users who committed a post that included the word “exemption” for the two sites.

Figure 9 shows the proportion of new users of each site who committed a post containing the word “exemption” by month. On average, about 24.42% and 4.34% of new users committed such a post at the 2 sites, respectively. The proportions were fairly constant over the study period, with the exception of early 2007 for the second site, which was founded shortly before we began data collection.

**Figure 10:**
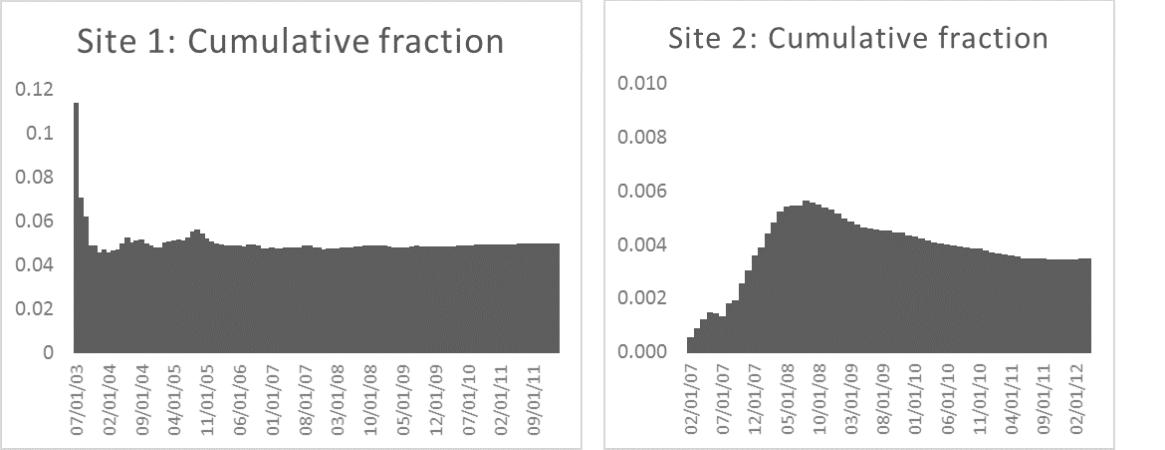
Cumulative proportion of posts that contained the word “exemption” for each site.

Figure 10 shows the monthly cumulative proportion of posts that included the word “exemption” for the 2 sites. As users can view old as well as new posts, this metric helps characterize the pervasiveness of exemption-related content on the sites. On mothering.com, the fraction of posts pertaining to exemptions stabilized at a proportion of about 4.81%. For the second site, the cumulative fraction had an initial rise that then stabilized at about 0.34%.

**Figure 11:**
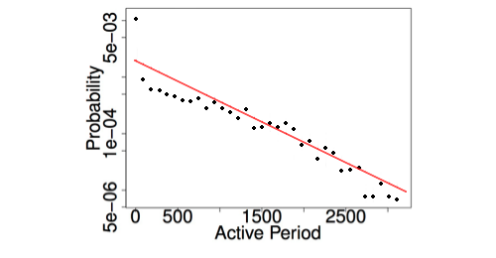
Log-linear plot of the distribution of user-activity duration (in days) for the mothering.com site.

A log-linear plot of the distribution of user-activity duration (in days) for the mothering.com site is presented in Figure 11. Note that the exponential tail (cut off) starts at around 2.5 years (ie, 1000 days), which is the typical age by which most children have received most of their vaccines. This suggests that new mothers are most active in the vaccination discussions and mothers become less active as their children pass the age when most vaccines have been administered.

## Discussion

### Principal Findings

The methods we have developed for this study allow us to discover stories circulating informally on social media sites. Our system can detect the presence, persistence, and pervasiveness of story signals on otherwise very noisy sites, aggregate these story signals into a narrative framework, and provide a clear mechanism for tracing the emergence of specific strategies endorsed in these stories that parents might adopt to counteract perceived health-related threats. The sites play an important role in exposing parents to the ideas of vaccinations as threat, and the use of exemptions as a strategy to combat that threat [30]. Any new parent joining these sites, irrespective of their orientation to vaccination, is exposed to stories that activate the narrative framework of vaccination as threat and exemption as strategy.

On the sites we studied, the narrative framework is one where vaccines pose a threat to children, and parents in their role as protectors devise strategies, most often the use of exemptions, to thwart that threat. The narrative framework is so widely dispersed that it has traversed many segments of the parenting sites. A strong, persistent signal in these discussions reveals that parents actively pursued information about exemptions on an ongoing basis. New parents who joined these sites were likely to be quickly exposed to the beliefs encoded in these stories and the underlying narrative framework.

Given the well-established 90-9-1 rule of social media, where 90% of visitors simply read without commenting (9%) or contributing (1%) [31], it is very likely that the narrative framework is reaching a much larger audience than simple user statistics suggest. Note that the 1.99 million posts we studied had an aggregate view count of more than 20 million views from registered users (unregistered users could view the posts, but their views were not recorded and therefore not tabulated). Even for parents who may not have initially believed that vaccines are harmful, the persistent circulation of stories about the potential harmfulness of vaccinations and the efficacy of the strategy of exemption to protect children from this alleged threat could convert some parents to embracing these beliefs [32].

### Limitations

Our work has certain limitations. As with all social media research, it is not clear that the sites on which we focused are representative of parents and health care decision makers as a whole. Although the 2 sites we used are very popular among parents, we recognize that they do not capture the broad range of discussions that take place in informal settings not easily observed, such as the playground, school gatherings, and other places where parents interact. In those settings, ethnographic fieldwork could provide important qualitative perspectives on vaccine-related discussions and storytelling [24]. At the same time, it is important to recognize that even settings that are more conducive to qualitative ethnographic methods are not immune to the influence of social media, which has increasing penetration into everyday life.

We recognize that parenting sites have certain biases. Mothering.com, for example, given its long-standing relationship with the now defunct *Mothering* magazine, has an anti-vaccination bias [11,12]. The well-known social network phenomenon of homophily might be creating an ideological echo chamber on these sites. Nevertheless, posts and user participation on the second site, which draws from a more ideologically diverse population, also reflect the persistent and prevalent nature of the vaccine-exemption narrative framework. Extending our approach to a broader sampling of parenting sites might mitigate the potential biases in these posts. At the same time, extending the approach to other types of social media where conversations are less organized, such as Facebook, might capture a broader series of narrative frameworks structuring vaccine-related conversations. Certain popular social media sites, such as Twitter, given the significant constraints on post length, are not included in this study.

Our work does not currently include sentiment detection. Although we advance the inclusion of social media in health care beyond topic discovery to an analysis of the underlying narrative structure of those discussions, we have not studied the manner in which those discussions are framed, which sentiment detection may be able to provide.

Data privacy continues to be a significant concern for social media research. In our study, we anonymized all of our data as part of the indexing process, and thus were unable to exploit certain features of individual user and user community data. The trade-offs between user privacy and research benefits are part of the constantly shifting terrain of social media research, and we chose to err on the side of privacy. Data access is becoming an equally significant problem, as social media corporations are greatly reducing access to data that people post and share on their sites. These limitations make it increasingly difficult to track large-scale conversations over long periods of time.

### Comparison With Prior Work

Vaccination decision-making has been broadly studied [33], and the impact of social media on health care decision-making is receiving increasing attention [34,35]. Several important studies have focused on people’s social media reactions to emerging health care crises such as disease outbreaks, where news stories often drive participation [35,36]. The role of narrative on social media sites as a persuasive rhetorical strategy in regard to health care decision making has also been explored, although this important work is largely preliminary [3,13,22,37]. Our work shifts attention away from bursts of activity on sites such as Twitter to a complementary examination of long-term conversations that evolve over many months and even years, with a primary focus on the emerging and endemic narrative frameworks that inform these conversations, aligning with other large-scale studies of attitudes toward health care decisions [38-40].

Narrative is recognized as a key means for shaping belief. Radzikowski et al [13], in their study of reactions to the California measles outbreak, develop a narrative model based on keyword cooccurrence in Tweets, an excellent first pass at such a model, given the limiting factors that Twitter imposes on Tweet length. Given the length of the posts in our data, we are able to develop a more elaborate narrative model, allowing us to extend the pairwise associations among actants. Our method not only shows that these pairwise relations are the key part of the conversations, but also reveals how actants are related in a context-dependent manner. Grant et al [5] provide clear evidence for the impact of personal experience narrative on vaccination attitudes through the qualitative comparison of 4 websites. Kitta [22], who worked with a similar structural model for vaccine narrative, develops an important typology of vaccine stories, whereas Kata [3] determines the tropes that are functional on anti-vaccine websites. Our automated methods allow us to extend that qualitative work to very large-scale data (millions of posts), thereby operationalizing aspects of traditional text analytic methods.

Random sampling methods are another approach for understanding developing attitudes toward health care. However, the narratives we discover would be difficult to identify using random sampling approaches. Whereas the use of focus groups has shown itself to be particularly helpful in devising messaging campaigns for specific communities [41,42], understanding the narratives operative in a community and at the scale of big data may help refine those messages [43-45]. Mathematical modeling for vaccine decision-making [46], while promising, makes specific assumptions that need to be validated from real-world data for the results to be actionable. Our approach may help provide that data.

### Conclusions

Injecting an idea, such as the efficacy of exemption as a strategy to avoid vaccination, into online communities has the potential to influence many people—the idea can, in a phrase, “go viral.” Given the persuasive nature of personal experience narrative, storytelling plays a central role in exposing people to ideas and converting people to particular beliefs. Importantly, people are inclined to believe first-hand accounts from members of their community, as opposed to official pronouncements [5]. Social network theory has established a strong tendency toward homophily in online communities that often results in shared trust between community members [47]. Notably, when “high degree” members (those with many connections in their network and to other networks) of a social network become exposed to beliefs and embrace them (in this case, the notion of exemptions), the conditions in the network become primed for rapid dissemination of those beliefs throughout the network. Unfortunately, once established, such beliefs are very diffcult to change [33]. We believe that the personal stories highly popular on these sites make use of the shared trust developed in online forums and thus act as an ideal method for converting nodes to the beliefs encoded in those narratives. In our study, the persistence of the exemption signal suggests a broad-scale susceptibility in these networks to the exemption strategy for dealing with the “vaccination threat.”

The sheer volume of discussions on social media sites dedicated to parenting along with the knowledge that many people use Internet resources as their first line of health care information mean that these forums deserve ongoing attention [39,48]. Identifying the vaccine exemption narrative framework and its activation through individual storytelling is an important step in understanding how people discuss this topic on these sites. Similarly, identifying endemic signals, those with the greatest persistence and pervasiveness, can help separate ideas that have a very brief life span from underlying narrative frameworks that provide a foundation for repeated stories that contribute to ideas and attitudes becoming entrenched. Ultimately, our goal is to contribute to a system that monitors health care–related websites for emerging beliefs and attitudes, and that recognizes the power of narrative to persuade and create communities of like-minded individuals. Our work brings us a step closer to such a system.

## Multimedia Appendix 1

Supplemental materials.
