# Supplementary material for: “Mommy Blogs” and the Vaccination Exemption Narrative: Results From A Machine-Learning Approach for Story Aggregation on Parenting Social Media Sites
Source: JMIR Public Health Surveill. 2016 Nov 22;2(2):e166. doi: 10.2196/publichealth.6586 (PMC5141339; doi:10.2196/publichealth.6586)
Supplement: Supplementary file 1 [file publichealth_v2i2e166_app1.pdf]

My Sites Reader

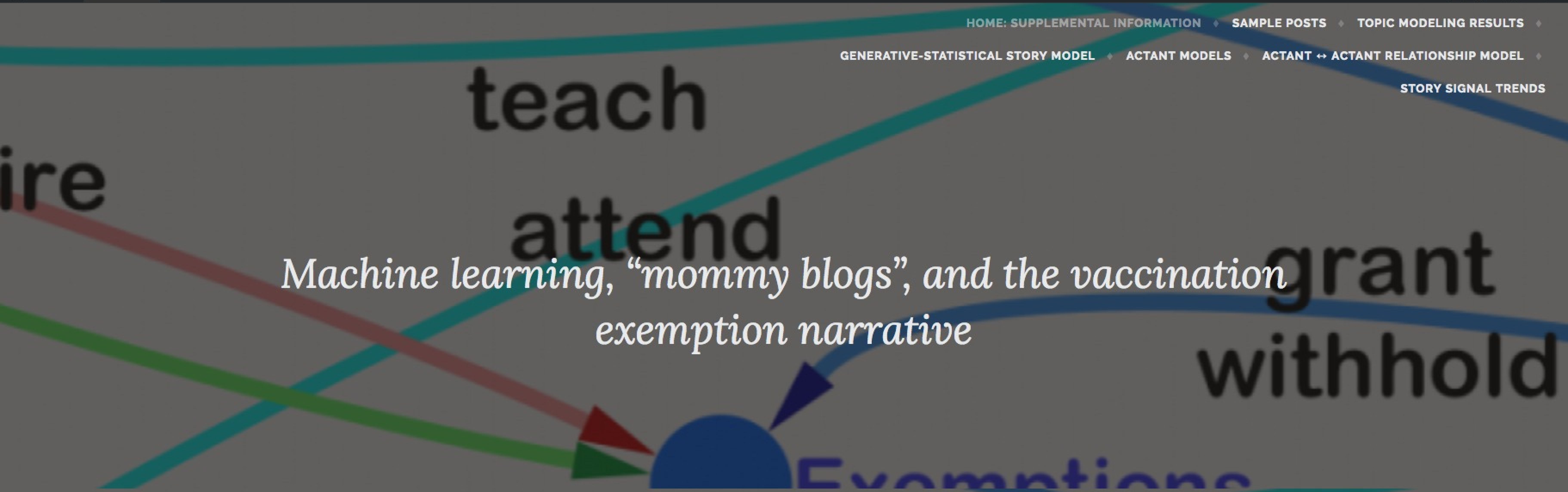

Machine learning, "mommy blogs", and the vaccination exemption narrative

HOME: SUPPLEMENTAL INFORMATION ♦ SAMPLE POSTS ♦ TOPIC MODELING RESULTS ♦
GENERATIVE-STATISTICAL STORY MODEL ♦ ACTANT MODELS ♦ ACTANT ↔ ACTANT RELATIONSHIP MODEL ♦
STORY SIGNAL TRENDS

Supplemental Information

Sample Posts

Topics from CRWT and LDA

Generative-Statistical Story Model

Actant Models

Actant ↔ Actant Relationship Model

Story Signal Trends

Contact corresponding authors:

Timothy R. Tangherlini (Center for Digital Humanities, UCLA)

Vwani Roychowdhury (Dept of Electrical Engineering, UCLA)

Edit

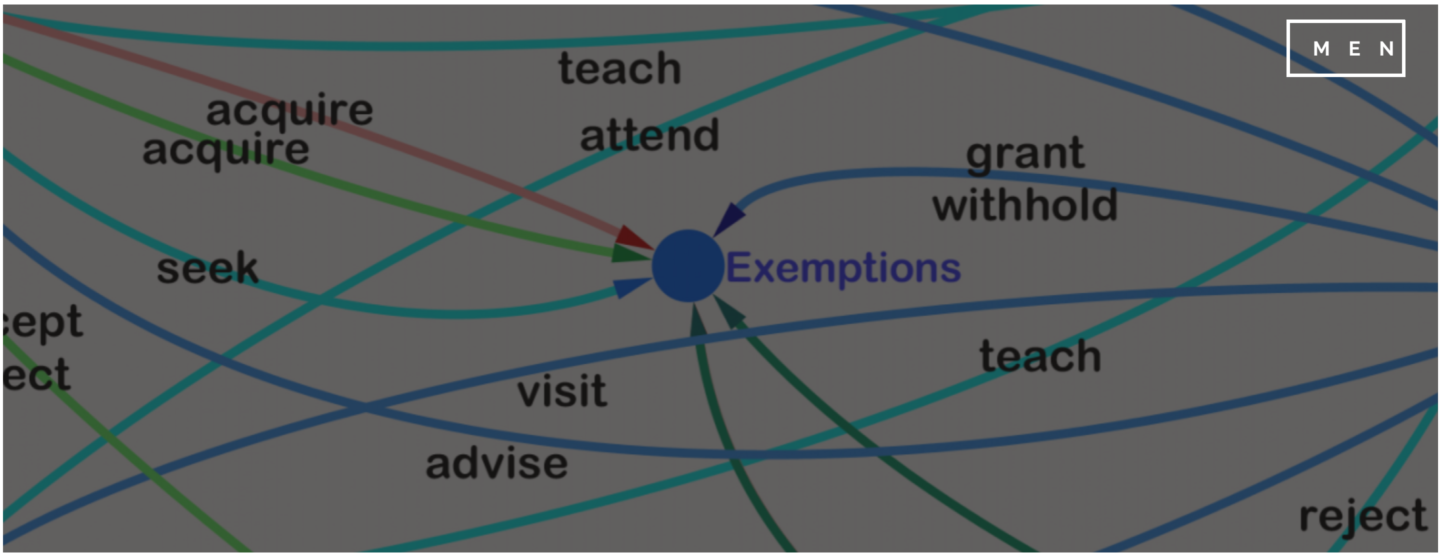

M E N

Below is a very small sample of some of the ~2 million posts with which we worked. In the sample posts below, all actants are in bold while significant ones are also italicized. The relational verb between the significant actants is underlined.

• My **hubby** and **I** are presently concerned about the ingredients – our **faith** prevents us from *allowing* certain **substances** into our **son**'s body.

• **I** have come to the conclusion that there is more of a likelihood that my **child** would *have* a bad **reaction** to certain **vaccines** than getting the actual disease itself .

• Every **vaccine** *has* a very small chance of more serious **side effects** (generally linked to allergic reactions , about which **parents** could not be expected to know before **vaccination**, because the **child** is so young), and a larger chance of less serious **reactions** (soreness , fever , redness) .

• He mentioned piggy backing off other's **immunizations** and how **autism** isn't *caused* by **vaxes** and a bunch of other things just to try to convince me to vax my **son** .

• It is ironic that the pro-vax crowd will laugh off the **autism–vaccines** link as if it is somehow preposterous – yet here is just one example of a **child** who was *diagnosed* with **autism** and the **government** *conceded* his **vaccine injury**.

•**I** certainly wouldn't want **my kids** to *suffer* socially because of our **decision not to vax** , but OTOH if I say something that can save one family from the pain of a serious **reaction** or **death** of a child , then maybe it's worth it .

• This concern is reinforced by a study which revealed that 1 in 175 **children** who completed the full DPT series *suffered* **severe reactions**", [7] and a **Dr**'s report for attorneys which found that 1 in 300 **DPT immunizations** *resulted* in **seizures**.

• If a **parent** wants to *exempt* their **child** only from the **MMR** , **Hep A** and **varicella vaccines** because of the aborted fetal tissue, the **religious exemption** would *be invalid* in almost every state .

• Even if the **Church** *told* all Catholic **parents** not to let their **child** get the **MMR** for instance , most **parents** would have to still be required to *submit* a **religious exemption** which would *exempt* all

vaccines.

• **I** think , first of all , that since **my kids** never *had* a **reaction** to **vaccines**, I just would never be able to forgive myself if they got a really horrible disease that would have been preventable through taxes .

• Here is the Hawaii **immunization** brochure , which states the **exemption** forms can also be *obtained* from the **school**: **Immunization** and TB code : Surprisingly, **I** don't see anything about religiously *exempting* a **child** from the TB screening requirement in the code .

• My **kids** are partially vaxed and **we** just *submitted* a **religious exemption** to the **school** she will be attending this fall.

• My younger **daughter** may be going to **preschool** next year and **I** am either going to have to *immunize* or *claim* a **religious exemption**, and I want my thoughts together on the topic so **I** can *advocate* for **her** .

• The **CDC** here told me they wouldn't *accept* the any **kids** w/out **shots**, even with a signed **exemption**.

• If the **school** has funding by the **government** then it is my understanding they must *accept* the **exemption**

• For Peace Corps, the US **Government** *requires* **vaccinations** BUT there is a **religious exemption** and disclaimer, I 'm sure of it – anyway, there was in 1998.

——————————————

Unmarked Examples

If submitting a religious exemption , I would " opt out " of any state registries .

I have stopped vaccinating my son (18 months) because I am taking time out to research so I have also been putting off vaccinating my DD .

I have stopped vaccinating my son (18 months) because I am taking time out to research so I have also been putting off vaccinating my DD .

MONEY Magazine , December , 1996 Volume 25 Number 12 has an entire article devoted to vaccine reactions , one of them being a young mother who got polio from her newborn daughter 's diaper after her polio vaccination .

I get the feeling that the principle of getting lots of children vaccinated is seen as more important than what happens to any individual child (why does this sound familiar ?) .

Â I Â am a former Â anti vax/delayed mom who is now more or less fully vaccinating my kids .

This is based on the fact that our lifestyle has changed : I 'm going back to work , the kids are in

school/preschool , and in four years ‘ time , we ‘re going around the world for a year as a family , and they will need a whole bunch of other vaccines , and I ‘d rather space them out a bit. Â Having kids that were not vaccinated Â no longer made sense for our Â lifestyle .

Â It is because of this core disagreement that I do n’t ever see myself ” fully ” vaccinating my children .

Â Kanna , what is the schedule of recommended vaccinations in Germany ? Â Do you have a link (in English , please) ? Â I ‘m just curious whether or not my children would be ” fully ” vaccinated to German standards. Â The U.S. supposedly recommends/requires more vaccines than any other western country. Â Hep A is actually rather easy to catch .

I held out and did n’t vaccinate at all up until my son was 6 months old .

I guess I am still not a fully vaxing parent , but I have very few qualms about vaccinating my kids at this point. Â sure if this was posted here yet.. : : : ; nav = 3YeX : Because abnormal paps and cervical cancer will just * poof * disappear when you get everyone vaxed .

Will children who are vaccinated now have children of tomorrow with severe birth defects ?

They have not compared a group of children who were vaccinated according to schedule with those who were vaccinated on a delayed schedule .

That is , supposedly only among kids who are currently being vaccinated or have had diseases that impart natural immunity .

I have and will expose my children to the chicken pox. Â One was vaccinated at whenever kids get that vaccine , but I exposed him anyway in hopes that he ‘d get it. Â I went to a friend ‘s house with 3 of my children for our pox party and at day 6 , two children had weird whelps on their abdomen that went away and then at day 14 , one of them got the chicken pox. Â I took pictures for proof of immunity. Â Â Â Now , I have a 2yo and when he gets closer to 3 , I will be looking for pox again. Â I did it mainly so that they could get it when it was convenient for all of us (spring-time) and get it over with young so that they would n’t get it when they were older .

Let ‘s stay on track with rabies here please . have decided to not vaccinate my new baby boy , or at least until he is 2/3 .

And just like the United States , pertussis is endemic in the UK : July 7 , 2006 Whooping cough still infecting millions of vaccinated children a friend of mine gets these little red spots on her torso and scalp .

But , I suppose her ” fear ” of the unvaccinated hordes giving measles to her new babies wanted her to ensure that her daughter was vaccinated prior to her giving birth .

she had to go and pay out of pocket to prove it was in fact rubella , even though her daughter had been vaccinated against it .

My best friend is like family to me , and I plan on raising my daughter to love her children like family

, so if I have to get her vaccinated in order to do that , then I wonder if I should ?

She had not been vaccinated for the same reasons I do n't want to vaccinate my daughter .

vax

My kid is already an adult , I did n't do anything special just because we did n't vax .

I guess I 'll be thinking twice about putting my toddlers into any classes (or other close contact) with other flumist vaxed toddlers .

She is so supportive , even though she does believe children should be vaxed routinely .

I do n't know much about the source of the article , but found it interesting . 'm wondering : Â On the forums , I found quite a few stories of moms that have vaxed older kids , but regret that decision and are now not vaxing/selectively vaxing/delayed vaxing their younger kids .

Â Â We did not vax either of our kids , and this decision was based on our lifestyle at the time Â (I was at home with the kids , we live in a smallish community) .

I 'm so sorry about your RA ! Â That totally stinks ! Â I 've been lucky so far to have an easily-controlled type , as does my daughter. Â My son 's asthma , however , is not autoimmune at this point – he has reactive airway-type/non-allergic asthma. Â His is n't triggered by pollen/foods/pets , but by cold air ; occasionally a virus takes a deep dive into his lungs. Â Â Interesting about your bout with mumps. Â I was completely vaxed and had the mumps anyway as well , around age 9. Â I had read somewhere that there 's a correlation between people who are vaxed and do n't show immunity after the MMRs and later development of autoimmune disease. Â I 'll try and find it again .

I 'm so sorry about your RA ! Â That totally stinks ! Â I 've been lucky so far to have an easily-controlled type , as does my daughter. Â My son 's asthma , however , is not autoimmune at this point – he has reactive airway-type/non-allergic asthma. Â His is n't triggered by pollen/foods/pets , but by cold air ; occasionally a virus takes a deep dive into his lungs. Â Â Interesting about your bout with mumps. Â I was completely vaxed and had the mumps anyway as well , around age 9. Â I had read somewhere that there 's a correlation between people who are vaxed and do n't show immunity after the MMRs and later development of autoimmune disease. Â I 'll try and find it again .

Â Â Â I did not vax either of my sons until they were 7 and 4 .

I still delay vaxed with my daughter , starting with 1 DTaP at 9 mos .

I guess I am still not a fully vaxing parent , but I have very few qualms about vaccinating my kids at this point. Â sure if this was posted here yet.. : : : ; nav = 3YeX : Because abnormal paps and cervical cancer will just * poof * disappear when you get everyone vaxed .

I guess I am still not a fully vaxing parent , but I have very few qualms about vaccinating my kids at this point. Â sure if this was posted here yet.. : : : ; nav = 3YeX : Because abnormal paps and cervical

cancer will just * poof * disappear when you get everyone vaxed .

I can understand vaxing your kid for pertussis to protect your kid , but do n't think by not vaxing for pertussis , you 're going to cause some kind of " pertussis epidemic " or whatever Izobelle meant by " how it would be if in three years , everyone made the same calculation as you .

I can understand vaxing your kid for pertussis to protect your kid , but do n't think by not vaxing for pertussis , you 're going to cause some kind of " pertussis epidemic " or whatever Izobelle meant by " how it would be if in three years , everyone made the same calculation as you .

In countries that do n't vax for CP routinely , it 's still a disease of 80-90 year olds. Â My boys are older now-teenagers. Â The shot came out when they were preschool age and it was n't mandatory yet. Â The doctor would ask if I wanted my boys to get it and I 'd say no , because I was hoping they 'd catch it on their own. Â (this is before I learned health reasons not to give shots , etc) Â But they did n't get it and two years later I gave in and said OK. Â If I could do it again , and I found a way to expose them to it , I would for sure ! Â It 's good for them to get it when they 're young , that way they have lifetime immunity and wo n't Â have any chance of getting Â it when they 're older. Â I wished my boys would have got the chicken pox on their own , way back then. Â Chicken pox is n't a big deal to get. Â It 's a pain , but they get over it just like they do colds and the flu. Â Plus it 's better to get it young before they 're in school and have to be out , or miss whatever other activities they may be into as an older child .

I 'll admit to feeling better once my son was past that age , but it would n't have been any different had we vax 'd him either way it 's still a risk and potentially dangerous for all infants regardless of their vax status .

I feel far safer with that to hand than I think I would if I 'd vaxed the kids .

He was normally vaxed as a kid and was in the navy for a short time , 7 years ago .

Not the same vax but my personal evidence that vax 's do n't work… yes it antidotel… My older son (fully vaxed and has always been on the recommended schedule) got mumps when he was two .

Their excuse was that vaxed kids can sometimes get it , but it looks so different it 's impossible to diagnose .

Yes , the incidence of CP in vaxed kids is incredibly underreported .

unvax

My unvaxed kids have reached the teen and pre-teen stage , and by the time you get there with unvaxed kids , I think you realize that there is nothing special needed. Â Great book suggestions !

My unvaxed kids have reached the teen and pre-teen stage , and by the time you get there with unvaxed kids , I think you realize that there is nothing special needed. Â Great book suggestions !

But with Hib , the " b " strain is basically gone now (yes , because of vaccination , but this is n't something that unvaxed kids are a big threat to " herd immunity " for , either) and the biggest risk

factors are being around smoke , being in daycare , and not being breastfed .

Â In many cases the chances of your child getting a disease are close to zero , in some the diseases are pretty mild , and in others the vax is n't overly effective. Â Â Â In general (not directed toward you) : I guess we all have our own thing – but the paranoia around the unvaxed drives me insane .

One person shoots up and sheds the virus to all vaxed and unvaxed kids to catch If you 're following the recommended schedule your babe would have already had 2 DTaP shots. Â Â Â Sorry , I meant not fully vaccinated .

I do n't think that finding breastfed , well-nourished vaxed children to compare to unvaxed would be that hard .

Since they 'll probably find some unvaxed kids with autism and adhd , they 'll need to ask the mother if she had Rhogam shots , dental work on fillings when pregnant , etc .

This expert has also stated that in his experience of treating vaxed and unvaxed children , (for over 20 years) the unvaxed are healthier .

This expert has also stated that in his experience of treating vaxed and unvaxed children , (for over 20 years) the unvaxed are healthier .

(There will be a mix of vaxed , selectively vaxed , and unvaxed kids at the party .

My daughter is now 5 months and unvaxed and we just had our first 'reminder ' , but I was wondering how nasty they could get ?

I usually just add that they should then find great comfort in their all-mighty vaccine from anything my unvaxed child might expose theirs to I would ask them to explain to me their expertise in herd immunity…then debunk all of especially that a bunch of the vaccines do not prevent transmission… how does that fit in with the theory ?

Even an adult male could theoretically contract the measles from an unvaxed child and become sterile as a result .

more likely being hurt by a vaccine than an unvaxed child .

Just do some research about the politics behind pharma , fda , biotech , and the cdc and how they all attain thier executive positions,patents and profits , then look at how scienctific journals and ghost writers exploit the use of an MDs signature and only the 'postive results ' get published so they can sell us more of this garbage they call vaccines and experimental pharmecuticals , kindof a legal way to get guinea pigs on board ; –not my child the idea of stating that unvaxed people put society at risk is morally an ethically unfounded .

In terms of the unvaxed child giving measles to an adult : Where did the measles come from in the first place ?

VPDs do n't spontaneously arise in unvaxed children .

Also , for example , pertussis exists mostly in adults who WERE vaccinated (and it ‘s since worn off) – it ‘s not that unvaxed kids are the reservoir , it ‘s just the general population .

Handled with care and commonsense (hygiene , good nutrition , staying away from coughing people , building proactively a strong immune system) no baby is more endangered by an unvaxed child than by a vaxed one .

B) how the heck is an unvaxed child supposed to transmit 6 or diseases at once ? ?

receiv

If you heard about the ” cases ” with the Amish – they were just kids who had antibodies to the vaccine strain of polio which meant that they had been exposed to someone who had received the oral polio vaccine and developed antibodies .

I love (not really) how they do n’t like to point out their statistics include people who are malnourished and do n’t receive medical care and/or have neglectful parents) * double post * sorry Yeah , my DD was fully vaxed through her toddler ones and was exposed to chicken pox last year .

; dopt = Abstract Duration of hepatitis B immunity in low risk children receiving hepatitis B vaccinations from birth .

Because three of these vaccines were combined into a single shot (DTP) , children received five shots by the time they were 2 years old and not more than one shot at a single visit .

Because six of these vaccines were combined into two shots (DTP and MMR) , and one , the polio vaccine , was given by mouth , children still received five shots by the time they were 2 years old and not more than one shot at a single visit .

Now , children could receive as many as 20 shots by 2 years of age and five shots in a single visit !

By 2005 , it is likely that children will also be recommended to receive a meningococcal vaccine , the influenza vaccine and the hepatitis A vaccine , all given as shots .

IF the vaccine was completely chemical , toxin and preservative free , and IF the vaccine was just the virus or bacteria of the disease , and IF the vaccine was applied topically in the nose or sprayed in the mouth so that the body ‘s immune response would be able to act as it is meant to do , rather than having to deal with something injected into the body , and IF the vaccine was able to induce a full immune response for LIFE , and IF the immunities of that vaccine would pass through breastmilk to my future grandbabies just as if the mother had gotten the true disease herself rather than just a vaccine , and IF the vaccine did not in any way alter the normal immune system any more than the disease itself does , but instead enhanced it , and IF the vaccine was guaranteed to not cause autoimmune problems later in life or any other awful consequences any more than the disease itself would , and IF the vaccine was given one at a time , as is done in real life when one is exposed to one thing at a time , and IF the vaccine came with a ” care ” packet of info – such as – ” Your child has just received a topical measles virus .

Debating vaccines with people that often do n’t even know WHAT their children received (” oh , my

Doctor ‘s office knows !

My intuition has prevented me from having my 5 year old receive his current round of vax , but both of my children received the course up to age 2 .

I just moved to Utah from Manitoba , and also received a letter – from the Canadian gov’t – about my son missing this year ‘s vax .

While most US children receive immunization for diphtheria and tetanus , many adults may not realize that over time the protection provided by the shots can wane .

A child ‘s chance of SIDS is 8 times higher after they receive that shot .

are you comfortable with the concept that your child , after having received the varicella vax (which is a live virus vaccine) , will be shedding the virus for up to 30 days after administration ?

I mean , if nearly every child receives the shot and now were learning about a drastic increase in childhood leukemia , who would the comparison group consist of and how would we isolate previous vit k administration from any and every other possible cause ?

If I have any more children , they will not receive even one shot , now that I have FINALLY educated myself with both pro-vaccine and anti-vaccine sources .

I asked her what I needed to turn in if my son had already received some vaccinations but I have since obtained a religious exemption .

I talked to his mom when she came to pick him up , and asked her which vaccines her son received today , and she could n’t remember .

At the same time , parents noticed that their healthy children were descending into silent , disturbed , and physically ill behavior after receiving vaccinations .

Discussions about tv shows are hosted in that forum . son will be 4 next month and has received all recommended vaccines until this point (his last one was easily a year ago) .

get

I do n’t get , why do we need a special book on raising a kid vax free ?

My unvaxed kids have reached the teen and pre-teen stage , and by the time you get there with unvaxed kids , I think you realize that there is nothing special needed. Â Great book suggestions !

-Angela Why not have a titer drawn (blood test to check for immunity) something like 95 % kids get immunity on the 1 shot .

I get the feeling that the principle of getting lots of children vaccinated is seen as more important than what happens to any individual child (why does this sound familiar ?) .

I get the feeling that the principle of getting lots of children vaccinated is seen as more important than what happens to any individual child (why does this sound familiar ?) .

Initially , I had planned on getting my middle child up-to-date on a very slow schedule (she was just turning 3 and had n't had any vaxes yet) , but it was emotionally traumatic for her , so I decided it would be better to get the shots out of the way quicker .

Â One fundamental question : What is the ROLE of a vaccine ? Â Â To me , a selective vaxer , and to public health authorities , the obvious answer is to protect children from getting and spreading particular diseases .

I guess I am still not a fully vaxing parent , but I have very few qualms about vaccinating my kids at this point. Â sure if this was posted here yet.. : : : ; nav = 3YeX : Because abnormal paps and cervical cancer will just * poof * disappear when you get everyone vaxed .

I have and will expose my children to the chicken pox. Â One was vaccinated at whenever kids get that vaccine , but I exposed him anyway in hopes that he ‘d get it. Â I went to a friend ‘s house with 3 of my children for our pox party and at day 6 , two children had weird whelps on their abdomen that went away and then at day 14 , one of them got the chicken pox. Â I took pictures for proof of immunity. Â Â Â Now , I have a 2yo and when he gets closer to 3 , I will be looking for pox again. Â I did it mainly so that they could get it when it was convenient for all of us (spring-time) and get it over with young so that they would n't get it when they were older .

I have and will expose my children to the chicken pox. Â One was vaccinated at whenever kids get that vaccine , but I exposed him anyway in hopes that he ‘d get it. Â I went to a friend ‘s house with 3 of my children for our pox party and at day 6 , two children had weird whelps on their abdomen that went away and then at day 14 , one of them got the chicken pox. Â I took pictures for proof of immunity. Â Â Â Now , I have a 2yo and when he gets closer to 3 , I will be looking for pox again. Â I did it mainly so that they could get it when it was convenient for all of us (spring-time) and get it over with young so that they would n't get it when they were older .

I have and will expose my children to the chicken pox. Â One was vaccinated at whenever kids get that vaccine , but I exposed him anyway in hopes that he ‘d get it. Â I went to a friend ‘s house with 3 of my children for our pox party and at day 6 , two children had weird whelps on their abdomen that went away and then at day 14 , one of them got the chicken pox. Â I took pictures for proof of immunity. Â Â Â Now , I have a 2yo and when he gets closer to 3 , I will be looking for pox again. Â I did it mainly so that they could get it when it was convenient for all of us (spring-time) and get it over with young so that they would n't get it when they were older .

I have and will expose my children to the chicken pox. Â One was vaccinated at whenever kids get that vaccine , but I exposed him anyway in hopes that he ‘d get it. Â I went to a friend ‘s house with 3 of my children for our pox party and at day 6 , two children had weird whelps on their abdomen that went away and then at day 14 , one of them got the chicken pox. Â I took pictures for proof of immunity. Â Â Â Now , I have a 2yo and when he gets closer to 3 , I will be looking for pox again. Â I did it mainly so that they could get it when it was convenient for all of us (spring-time) and get it over with young so that they would n't get it when they were older .

I have and will expose my children to the chicken pox. Â One was vaccinated at whenever kids get

that vaccine , but I exposed him anyway in hopes that he ‘d get it. Â I went to a friend ‘s house with 3 of my children for our pox party and at day 6 , two children had weird whelps on their abdomen that went away and then at day 14 , one of them got the chicken pox. Â I took pictures for proof of immunity. Â Â Â Now , I have a 2yo and when he gets closer to 3 , I will be looking for pox again. Â I did it mainly so that they could get it when it was convenient for all of us (spring-time) and get it over with young so that they would n’t get it when they were older .

I hope to expose him to natural pox a few more times during childhood as well , to hopefully help his immunity , and we ‘ll get him boosters or at the least titers at 10 and before he goes off to college. Â I would prefer for both kids to have good immunity to it when they are older , and be able to travel to England and non-CP-vaxxing-countries without worrying about their immunity to it. Â Â Everything else on the schedule both children got , on a slightly spread out schedule , but they were caught up by age 3. Â At 2 , they were no more ” dangerous to community health ” than an 11.9 month old who had n’t yet had the 12 month old series of shots .

In countries that do n’t vax for CP routinely , it ‘s still a disease of 80-90 year olds. Â My boys are older now-teenagers. Â The shot came out when they were preschool age and it was n’t mandatory yet. Â The doctor would ask if I wanted my boys to get it and I ‘d say no , because I was hoping they ‘d catch it on their own. Â (this is before I learned health reasons not to give shots , etc) Â But they did n’t get it and two years later I gave in and said OK. Â If I could do it again , and I found a way to expose them to it , I would for sure ! Â It ‘s good for them to get it when they ‘re young , that way they have lifetime immunity and wo n’t Â have any chance of getting Â it when they ‘re older. Â I wished my boys would have got the chicken pox on their own , way back then. Â Chicken pox is n’t a big deal to get. Â It ‘s a pain , but they get over it just like they do colds and the flu. Â Plus it ‘s better to get it young before they ‘re in school and have to be out , or miss whatever other activities they may be into as an older child .

In countries that do n’t vax for CP routinely , it ‘s still a disease of 80-90 year olds. Â My boys are older now-teenagers. Â The shot came out when they were preschool age and it was n’t mandatory yet. Â The doctor would ask if I wanted my boys to get it and I ‘d say no , because I was hoping they ‘d catch it on their own. Â (this is before I learned health reasons not to give shots , etc) Â But they did n’t get it and two years later I gave in and said OK. Â If I could do it again , and I found a way to expose them to it , I would for sure ! Â It ‘s good for them to get it when they ‘re young , that way they have lifetime immunity and wo n’t Â have any chance of getting Â it when they ‘re older. Â I wished my boys would have got the chicken pox on their own , way back then. Â Chicken pox is n’t a big deal to get. Â It ‘s a pain , but they get over it just like they do colds and the flu. Â Plus it ‘s better to get it young before they ‘re in school and have to be out , or miss whatever other activities they may be into as an older child .

In countries that do n’t vax for CP routinely , it ‘s still a disease of 80-90 year olds. Â My boys are older now-teenagers. Â The shot came out when they were preschool age and it was n’t mandatory yet. Â The doctor would ask if I wanted my boys to get it and I ‘d say no , because I was hoping they ‘d catch it on their own. Â (this is before I learned health reasons not to give shots , etc) Â But they did n’t get it and two years later I gave in and said OK. Â If I could do it again , and I found a way to expose them to it , I would for sure ! Â It ‘s good for them to get it when they ‘re young , that way they have lifetime immunity and wo n’t Â have any chance of getting Â it when they ‘re older. Â I wished my boys would have got the chicken pox on their own , way back then. Â Chicken pox is n’t a big deal to get. Â It ‘s a pain , but they get over it just like they do colds and the flu. Â Plus it ‘s better

to get it young before they ‘re in school and have to be out , or miss whatever other activities they may be into as an older child .

In countries that do n’t vax for CP routinely , it ‘s still a disease of 80-90 year olds. Â My boys are older now-teenagers. Â The shot came out when they were preschool age and it was n’t mandatory yet. Â The doctor would ask if I wanted my boys to get it and I ‘d say no , because I was hoping they ‘d catch it on their own. Â (this is before I learned health reasons not to give shots , etc) Â But they did n’t get it and two years later I gave in and said OK. Â If I could do it again , and I found a way to expose them to it , I would for sure ! Â It ‘s good for them to get it when they ‘re young , that way they have lifetime immunity and wo n’t Â have any chance of getting Â it when they ‘re older. Â I wished my boys would have got the chicken pox on their own , way back then. Â Chicken pox is n’t a big deal to get. Â It ‘s a pain , but they get over it just like they do colds and the flu. Â Plus it ‘s better to get it young before they ‘re in school and have to be out , or miss whatever other activities they may be into as an older child .

In countries that do n’t vax for CP routinely , it ‘s still a disease of 80-90 year olds. Â My boys are older now-teenagers. Â The shot came out when they were preschool age and it was n’t mandatory yet. Â The doctor would ask if I wanted my boys to get it and I ‘d say no , because I was hoping they ‘d catch it on their own. Â (this is before I learned health reasons not to give shots , etc) Â But they did n’t get it and two years later I gave in and said OK. Â If I could do it again , and I found a way to expose them to it , I would for sure ! Â It ‘s good for them to get it when they ‘re young , that way they have lifetime immunity and wo n’t Â have any chance of getting Â it when they ‘re older. Â I wished my boys would have got the chicken pox on their own , way back then. Â Chicken pox is n’t a big deal to get. Â It ‘s a pain , but they get over it just like they do colds and the flu. Â Plus it ‘s better to get it young before they ‘re in school and have to be out , or miss whatever other activities they may be into as an older child .

In countries that do n’t vax for CP routinely , it ‘s still a disease of 80-90 year olds. Â My boys are older now-teenagers. Â The shot came out when they were preschool age and it was n’t mandatory yet. Â The doctor would ask if I wanted my boys to get it and I ‘d say no , because I was hoping they ‘d catch it on their own. Â (this is before I learned health reasons not to give shots , etc) Â But they did n’t get it and two years later I gave in and said OK. Â If I could do it again , and I found a way to expose them to it , I would for sure ! Â It ‘s good for them to get it when they ‘re young , that way they have lifetime immunity and wo n’t Â have any chance of getting Â it when they ‘re older. Â I wished my boys would have got the chicken pox on their own , way back then. Â Chicken pox is n’t a big deal to get. Â It ‘s a pain , but they get over it just like they do colds and the flu. Â Plus it ‘s better to get it young before they ‘re in school and have to be out , or miss whatever other activities they may be into as an older child .

had

(I supposed I ‘d consider titers if I were in West Virgina or Mississippi and had to use schools or daycare) -Angela I gave my first vaccination ever to my 13 yo Â son ten days ago (we are moving overseas) – it was the MMR and he woke up this am slightly feverish , burning eyes and on and off nausea.. .

If you heard about the ” cases ” with the Amish – they were just kids who had antibodies to the vaccine strain of polio which meant that they had been exposed to someone who had received the

oral polio vaccine and developed antibodies .

If you heard about the " cases " with the Amish – they were just kids who had antibodies to the vaccine strain of polio which meant that they had been exposed to someone who had received the oral polio vaccine and developed antibodies .

If you heard about the " cases " with the Amish – they were just kids who had antibodies to the vaccine strain of polio which meant that they had been exposed to someone who had received the oral polio vaccine and developed antibodies .

Initially , I had planned on getting my middle child up-to-date on a very slow schedule (she was just turning 3 and had n't had any vaxes yet) , but it was emotionally traumatic for her , so I decided it would be better to get the shots out of the way quicker .

Initially , I had planned on getting my middle child up-to-date on a very slow schedule (she was just turning 3 and had n't had any vaxes yet) , but it was emotionally traumatic for her , so I decided it would be better to get the shots out of the way quicker .

Thank you , thank you , thank you for starting this thread ! Â Â We 've never vax 'd any of our kids – due to a strong family history of autoimmune diseases – but over the past winter our youngest developed asthma out of the blue ; he 's been in ; out of the hospital 4 times since December. Â We finally have that under control , but our FP ; I had a few long talks about it and we 've decided to very slowly start vaxing. Â Â I am terrified. Â Our oldest ds has a speech delay and sensory issues , our dd has juvenile RA and I have RA as well and I 'm just so scared that these will damage their immune systems [and mine] down the line. Â I feel so torn because the youngest is only 15 months old now – he 's so helpless in all of this – and we 're doing it for his benefit mostly ; in case we catch anything , hopefully the vaxes will at least make the disease less severe , and thus less likely to land him in the hospital. Â But how do I reconcile that with the damage this might do to my older 2 ? Â I would never forgive myself if something happened to them. Â Â I 'm down with the idea that as our lifestyles change we change along with them , but was it easy or hard for those of you going from non-vax to vax ? Â I 'm having such a moral dilemma about this. Â On the one hand , it 's a no-brainer , on the other if anything happens the fault is 100 % mine for giving the ok. Â I guess that 's the leap of faith we 're taking. Â So thanks for listening , sorry to highjack , and thank you OP for starting this very timely thread !

I 'm so sorry about your RA ! Â That totally stinks ! Â I 've been lucky so far to have an easily-controlled type , as does my daughter. Â My son 's asthma , however , is not autoimmune at this point – he has reactive airway-type/non-allergic asthma. Â His is n't triggered by pollen/foods/pets , but by cold air ; occasionally a virus takes a deep dive into his lungs. Â Â Interesting about your bout with mumps. Â I was completely vaxed and had the mumps anyway as well , around age 9. Â I had read somewhere that there 's a correlation between people who are vaxed and do n't show immunity after the MMRs and later development of autoimmune disease. Â I 'll try and find it again .

I 'm so sorry about your RA ! Â That totally stinks ! Â I 've been lucky so far to have an easily-controlled type , as does my daughter. Â My son 's asthma , however , is not autoimmune at this point – he has reactive airway-type/non-allergic asthma. Â His is n't triggered by pollen/foods/pets , but by cold air ; occasionally a virus takes a deep dive into his lungs. Â Â

Interesting about your bout with mumps. Â I was completely vaxed and had the mumps anyway as well , around age 9. Â I had read somewhere that there ‘s a correlation between people who are vaxed and do n’t show immunity after the MMRs and later development of autoimmune disease. Â I ‘ll try and find it again .

I wonder how many parents were informed AFTER the event that their children were given an extra vaccine . ‘ve been all over the map on vax ‘s in our family… DS had a number of vax ‘s before he was 1.5 , then nothing.. .

That is , supposedly only among kids who are currently being vaccinated or have had diseases that impart natural immunity .

I have and will expose my children to the chicken pox. Â One was vaccinated at whenever kids get that vaccine , but I exposed him anyway in hopes that he ‘d get it. Â I went to a friend ‘s house with 3 of my children for our pox party and at day 6 , two children had weird whelps on their abdomen that went away and then at day 14 , one of them got the chicken pox. Â I took pictures for proof of immunity. Â Â Â Now , I have a 2yo and when he gets closer to 3 , I will be looking for pox again. Â I did it mainly so that they could get it when it was convenient for all of us (spring-time) and get it over with young so that they would n’t get it when they were older .

Natural immunity is found to be longer lasting than vax immunity. Â Â That said , the other child we could n’t find CP in the wild before he needed titers or vax for preschool (and I did not want to go through the waiver process over one vaccine that I ‘m not specifically opposed to , I ‘d just prefer it naturally) , so he had the vax .

I hope to expose him to natural pox a few more times during childhood as well , to hopefully help his immunity , and we ‘ll get him boosters or at the least titers at 10 and before he goes off to college. Â I would prefer for both kids to have good immunity to it when they are older , and be able to travel to England and non-CP-vaxxing-countries without worrying about their immunity to it. Â Â Everything else on the schedule both children got , on a slightly spread out schedule , but they were caught up by age 3. Â At 2 , they were no more ” dangerous to community health ” than an 11.9 month old who had n’t yet had the 12 month old series of shots .

I had all required vaxes as a child back in the late 70 ‘s early 80 ‘s , but I made the decision a couple of years ago to stop vaxing my kiddos .

I ‘ll admit to feeling better once my son was past that age , but it would n’t have been any different had we vax ‘d him either way it ‘s still a risk and potentially dangerous for all infants regardless of their vax status .

It was funny when the ped tried to say it was mild due to the vax , then realized the vaxed kid actually had the worst case between the two of them .

I would not bring a baby (knowingly) around a newly vaxed kiddo That ‘s what I would think as well–I was surprised when this friend had her daughter vaxed , while she was pregnant .

she had to go and pay out of pocket to prove it was in fact rubella , even though her daughter had been vaccinated against it .

she had to go and pay out of pocket to prove it was in fact rubella , even though her daughter had been vaccinated against it .

have

My unvaxed kids have reached the teen and pre-teen stage , and by the time you get there with unvaxed kids , I think you realize that there is nothing special needed. Â Great book suggestions !

It does seem as though we have forced out the infectious diseases which are a normal part of childhood and replaced them with chronic diseases , but it would be good to have stats to quote at people who say that children are healthier due to vaccinations !

It does seem as though we have forced out the infectious diseases which are a normal part of childhood and replaced them with chronic diseases , but it would be good to have stats to quote at people who say that children are healthier due to vaccinations !

-Angela Why not have a titer drawn (blood test to check for immunity) something like 95 % kids get immunity on the 1 shot .

I have stopped vaccinating my son (18 months) because I am taking time out to research so I have also been putting off vaccinating my DD .

I have stopped vaccinating my son (18 months) because I am taking time out to research so I have also been putting off vaccinating my DD .

I do n't know much about the source of the article , but found it interesting . 'm wondering : Â On the forums , I found quite a few stories of moms that have vaxed older kids , but regret that decision and are now not vaxing/selectively vaxing/delayed vaxing their younger kids .

Thank you , thank you , thank you for starting this thread ! Â Â We 've never vax 'd any of our kids – due to a strong family history of autoimmune diseases – but over the past winter our youngest developed asthma out of the blue ; he 's been in ; out of the hospital 4 times since December. Â We finally have that under control , but our FP ; I had a few long talks about it and we 've decided to very slowly start vaxing. Â Â I am terrified. Â Our oldest ds has a speech delay and sensory issues , our dd has juvenile RA and I have RA as well and I 'm just so scared that these will damage their immune systems [and mine] down the line. Â I feel so torn because the youngest is only 15 months old now – he 's so helpless in all of this – and we 're doing it for his benefit mostly ; in case we catch anything , hopefully the vaxes will at least make the disease less severe , and thus less likely to land him in the hospital. Â But how do I reconcile that with the damage this might do to my older 2 ? Â I would never forgive myself if something happened to them. Â Â I 'm down with the idea that as our lifestyles change we change along with them , but was it easy or hard for those of you going from non-vax to vax ? Â I 'm having such a moral dilemma about this. Â On the one hand , it 's a no-brainer , on the other if anything happens the fault is 100 % mine for giving the ok. Â I guess that 's the leap of faith we 're taking. Â So thanks for listening , sorry to highjack , and thank you OP for starting this very timely thread !

Thank you , thank you , thank you for starting this thread ! Â Â We 've never vax 'd any of our kids – due to a strong family history of autoimmune diseases – but over the past winter our youngest

developed asthma out of the blue ; he ‘s been in ; out of the hospital 4 times since December. Â We finally have that under control , but our FP ; I had a few long talks about it and we ‘ve decided to very slowly start vaxing. Â Â I am terrified. Â Our oldest ds has a speech delay and sensory issues , our dd has juvenile RA and I have RA as well and I ‘m just so scared that these will damage their immune systems [and mine] down the line. Â I feel so torn because the youngest is only 15 months old now – he ‘s so helpless in all of this – and we ‘re doing it for his benefit mostly ; in case we catch anything , hopefully the vaxes will at least make the disease less severe , and thus less likely to land him in the hospital. Â But how do I reconcile that with the damage this might do to my older 2 ? Â I would never forgive myself if something happened to them. Â Â I ‘m down with the idea that as our lifestyles change we change along with them , but was it easy or hard for those of you going from non-vax to vax ? Â I ‘m having such a moral dilemma about this. Â On the one hand , it ‘s a no-brainer , on the other if anything happens the fault is 100 % mine for giving the ok. Â I guess that ‘s the leap of faith we ‘re taking. Â So thanks for listening , sorry to highjack , and thank you OP for starting this very timely thread !

Thank you , thank you , thank you for starting this thread ! Â Â We ‘ve never vax ‘d any of our kids – due to a strong family history of autoimmune diseases – but over the past winter our youngest developed asthma out of the blue ; he ‘s been in ; out of the hospital 4 times since December. Â We finally have that under control , but our FP ; I had a few long talks about it and we ‘ve decided to very slowly start vaxing. Â Â I am terrified. Â Our oldest ds has a speech delay and sensory issues , our dd has juvenile RA and I have RA as well and I ‘m just so scared that these will damage their immune systems [and mine] down the line. Â I feel so torn because the youngest is only 15 months old now – he ‘s so helpless in all of this – and we ‘re doing it for his benefit mostly ; in case we catch anything , hopefully the vaxes will at least make the disease less severe , and thus less likely to land him in the hospital. Â But how do I reconcile that with the damage this might do to my older 2 ? Â I would never forgive myself if something happened to them. Â Â I ‘m down with the idea that as our lifestyles change we change along with them , but was it easy or hard for those of you going from non-vax to vax ? Â I ‘m having such a moral dilemma about this. Â On the one hand , it ‘s a no-brainer , on the other if anything happens the fault is 100 % mine for giving the ok. Â I guess that ‘s the leap of faith we ‘re taking. Â So thanks for listening , sorry to highjack , and thank you OP for starting this very timely thread !

Vaccines , like everything else Â have risks , but as person who have seen people die long sad deaths from Hep B and few other things….I believe that benefit outweig the risks Â I ‘ve increased the number of vaxes for my children , but that ‘s the only change I ‘ve made over my parenting years .

Vaccines , like everything else Â have risks , but as person who have seen people die long sad deaths from Hep B and few other things….I believe that benefit outweig the risks Â I ‘ve increased the number of vaxes for my children , but that ‘s the only change I ‘ve made over my parenting years .

Â Kanna , what is the schedule of recommended vaccinations in Germany ? Â Do you have a link (in English , please) ? Â I ‘m just curious whether or not my children would be ” fully ” vaccinated to German standards. Â The U.S. supposedly recommends/requires more vaccines than any other western country. Â Hep A is actually rather easy to catch .

I ‘m so sorry about your RA ! Â That totally stinks ! Â I ‘ve been lucky so far to have an easily-controlled type , as does my daughter. Â My son ‘s asthma , however , is not autoimmune at this point – he has reactive airway-type/non-allergic asthma. Â His is n’t triggered by

pollen/foods/pets , but by cold air ; occasionally a virus takes a deep dive into his lungs. Â Â Interesting about your bout with mumps. Â I was completely vaxed and had the mumps anyway as well , around age 9. Â I had read somewhere that there ‘s a correlation between people who are vaxed and do n't show immunity after the MMRs and later development of autoimmune disease. Â I ‘ll try and find it again .

I guess I am still not a fully vaxing parent , but I have very few qualms about vaccinating my kids at this point. Â sure if this was posted here yet.. : : : ; nav = 3YeX : Because abnormal paps and cervical cancer will just * poof * disappear when you get everyone vaxed .

Will children who are vaccinated now have children of tomorrow with severe birth defects ?

They have not compared a group of children who were vaccinated according to schedule with those who were vaccinated on a delayed schedule .

That is , supposedly only among kids who are currently being vaccinated or have had diseases that impart natural immunity .

I have and will expose my children to the chicken pox. Â One was vaccinated at whenever kids get that vaccine , but I exposed him anyway in hopes that he ‘d get it. Â I went to a friend ‘s house with 3 of my children for our pox party and at day 6 , two children had weird whelps on their abdomen that went away and then at day 14 , one of them got the chicken pox. Â I took pictures for proof of immunity. Â Â Â Now , I have a 2yo and when he gets closer to 3 , I will be looking for pox again. Â I did it mainly so that they could get it when it was convenient for all of us (spring-time) and get it over with young so that they would n't get it when they were older .

I have and will expose my children to the chicken pox. Â One was vaccinated at whenever kids get that vaccine , but I exposed him anyway in hopes that he ‘d get it. Â I went to a friend ‘s house with 3 of my children for our pox party and at day 6 , two children had weird whelps on their abdomen that went away and then at day 14 , one of them got the chicken pox. Â I took pictures for proof of immunity. Â Â Â Now , I have a 2yo and when he gets closer to 3 , I will be looking for pox again. Â I did it mainly so that they could get it when it was convenient for all of us (spring-time) and get it over with young so that they would n't get it when they were older .

were

(I supposed I ‘d consider titers if I were in West Virgina or Mississippi and had to use schools or daycare) -Angela I gave my first vaccination ever to my 13 yo Â son ten days ago (we are moving overseas) – it was the MMR and he woke up this am slightly feverish , burning eyes and on and off nausea.. .

If you heard about the " cases " with the Amish – they were just kids who had antibodies to the vaccine strain of polio which meant that they had been exposed to someone who had received the oral polio vaccine and developed antibodies .

This is based on the fact that our lifestyle has changed : I ‘m going back to work , the kids are in school/preschool , and in four years ‘ time , we ‘re going around the world for a year as a family , and they will need a whole bunch of other vaccines , and I ‘d rather space them out a bit. Â Having

kids that were not vaccinated Â no longer made sense for our Â lifestyle .

Â Â Â I did not vax either of my sons until they were 7 and 4 .

I wonder how many parents were informed AFTER the event that their children were given an extra vaccine . ‘ve been all over the map on vax ‘s in our family… DS had a number of vax ‘s before he was 1.5 , then nothing.. .

I wonder how many parents were informed AFTER the event that their children were given an extra vaccine . ‘ve been all over the map on vax ‘s in our family… DS had a number of vax ‘s before he was 1.5 , then nothing.. .

They have not compared a group of children who were vaccinated according to schedule with those who were vaccinated on a delayed schedule .

I have and will expose my children to the chicken pox. Â One was vaccinated at whenever kids get that vaccine , but I exposed him anyway in hopes that he ‘d get it. Â I went to a friend ‘s house with 3 of my children for our pox party and at day 6 , two children had weird whelps on their abdomen that went away and then at day 14 , one of them got the chicken pox. Â I took pictures for proof of immunity. Â Â Â Now , I have a 2yo and when he gets closer to 3 , I will be looking for pox again. Â I did it mainly so that they could get it when it was convenient for all of us (spring-time) and get it over with young so that they would n’t get it when they were older .

I hope to expose him to natural pox a few more times during childhood as well , to hopefully help his immunity , and we ‘ll get him boosters or at the least titers at 10 and before he goes off to college. Â I would prefer for both kids to have good immunity to it when they are older , and be able to travel to England and non-CP-vaxxing-countries without worrying about their immunity to it. Â Â Everything else on the schedule both children got , on a slightly spread out schedule , but they were caught up by age 3. Â At 2 , they were no more ” dangerous to community health ” than an 11.9 month old who had n’t yet had the 12 month old series of shots .

I hope to expose him to natural pox a few more times during childhood as well , to hopefully help his immunity , and we ‘ll get him boosters or at the least titers at 10 and before he goes off to college. Â I would prefer for both kids to have good immunity to it when they are older , and be able to travel to England and non-CP-vaxxing-countries without worrying about their immunity to it. Â Â Everything else on the schedule both children got , on a slightly spread out schedule , but they were caught up by age 3. Â At 2 , they were no more ” dangerous to community health ” than an 11.9 month old who had n’t yet had the 12 month old series of shots .

And without CP vax or titers , they also were n’t any more of a danger than your average tourist child from Norway , Denmark , or England , where CP is not part of the vaccine schedule .

In countries that do n’t vax for CP routinely , it ‘s still a disease of 80-90 year olds. Â My boys are older now-teenagers. Â The shot came out when they were preschool age and it was n’t mandatory yet. Â The doctor would ask if I wanted my boys to get it and I ‘d say no , because I was hoping they ‘d catch it on their own. Â (this is before I learned health reasons not to give shots , etc) Â But they did n’t get it and two years later I gave in and said OK. Â If I could do it again , and I found a way to expose them to it , I would for sure ! Â It ‘s good for them to get it when they ‘re young , that way

they have lifetime immunity and wo n't Â have any chance of getting Â it when they 're older. Â I wished my boys would have got the chicken pox on their own , way back then. Â Chicken pox is n't a big deal to get. Â It 's a pain , but they get over it just like they do colds and the flu. Â Plus it 's better to get it young before they 're in school and have to be out , or miss whatever other activities they may be into as an older child .

About 3 years ago the pediatricians at NAS Corpus Christi (a naval base) were telling parents that they could not diagnose CP in vaccinated children .

If I were in your shoes , I absolutely would vaccinate ; but to be fair , I support vaccination and my child is going to be selectively/delayed vaxed .

Ugh and yet if they were so secure in their decision to vax why would they be afraid of your kids ?

Â One of the great parts of Mothering.com is that how it allows moms to glory in their strengths , to make decisions for their children that may not be popular , but that we believe in our guts are right for our kids. Â I try to take that feeling with me as we continue to navigate this family messiness. Â I still have a lot of anxiety. Â It 's hard to be ridiculed and turned into an outcast in any situation , but with one 's family , it 's just heart-wrenching. Â But everyone 's support and the words of wisdom help. Â I 'm trying to focus on what I 'm grateful for. Â And that list is very long. Â Â My son is seeing a new naturopathic doc who 's helping with the vaccine damage , which is a blessing. Â And my SIL wrote this message when I asked her if she and her husband were ever uncomfortable with our decision to not vaccinate when our boys are around her girls. Â (I was trying to understand my brother better ; SIL fully vaxes her kids .

She did n't do any vaccines with her two kids and they were really cool with that .

I can say that my particular college " debunked " all the vax myths , we were taught that the parents who did not vax were miseducated , and basically did not care about the well being of their children .

I can say that my particular college " debunked " all the vax myths , we were taught that the parents who did not vax were miseducated , and basically did not care about the well being of their children .

Just not something I 'm willing to chance with my own kids (since I have 2 cousins who will , most likely , never live alone and were fully vaxed) .

unvaccin

But , I suppose her " fear " of the unvaccinated hordes giving measles to her new babies wanted her to ensure that her daughter was vaccinated prior to her giving birth .

Of course it would be easy to compare vaccinated to unvaccinated kids by using unvaccinated kids that are that way because their parents want them to be , but this would n't be double blind .

Of course it would be easy to compare vaccinated to unvaccinated kids by using unvaccinated kids that are that way because their parents want them to be , but this would n't be double blind .

My dd is fully unvaccinated and all of my future children will not be vaxed either .

you do not become immune , you develope some antibodies…that ‘s it , you can still contract it , pass it along , get sick , it all boils down to one thing CURRENT STATE OF HEALTH nothing more What I would say is : ” How is my healthy unvaccinated child any threat to your vaccinated child if those vaccines have done what they were designed to do ?

When fronted with defending myself I found a quote ; use part of it ” How can an unvaccinated child be more of a risk than a vaccinated child if neither have the disease ?

If you believe in the efficacy of vaccines what are you worried about being around my unvaccinated child ? ?

And they do not compare vaccinated to unvaccinated children .

As I said before , I have spent a long time researching vaccination and also homeopathy , there are no toxins , it is completely safe and it provides parents of unvaccinated children the opportunity to treat diseases that conventional doctors do not know how to treat anymore .

Â To the OP : Many people will always believe that 1) all vaccines prevent transmission and provide herd immunity and 2) unvaccinated kids are thus disease magnets and a threat to public health when neither of these is true .

The New Zealand Immunisation Awareness Society ‘s survey [url] which compared the health of vaccinated children with the health of unvaccinated children was further confirmation of this .

Here ‘s the 1986 CDC recommendation for Hib polysaccharide : There have been a hord (well , more than a hord) of unvaccinated people who lived in the decades before the other vaccines were introduced and routinely given to only infants and school children , including HIb , and they never ” had anything ” either .

As for measles , mumps and rubella (MMR) immunization , unvaccinated children had slightly lower chances of having hay fever than those vaccinated in month 14 , although the difference was not statistically significant .

I ‘m really wondering why some pro-vax researchers do n’t get a study together showing how much healthier fully vaccinated kids are compared to totally unvaccinated kids .

The unvaccinated kids should just be a mess compared to the fully vaxed kids .

Heavy metal toxicity , encephalitis , acquired metabolic disorder , activated metabolic disorder , etc can all create autistic behaviors , and I am not trying to minimize or deny that , but any particularly clever debater , lawyer , or pro-vax pharma should be quick to point out that Autism is very provably a genetic occurrence that predates vaccines , happens more often to ” overly ” analytical parents , and happens to unvaccinated children .

I can see he is unvaccinated so he gets the measles but he infects vaccinated children That deserves an explanation from the vaccine manufacturers .

Only children who were fully vaccinated (i.e. , had either two doses if not previously vaccinated , or one dose if previously vaccinated) versus unvaccinated children were included in the analysis .

You can say your child is unvaccinated and still have them have some vaccines .

if you think the risk of shedding warrants quarantining vaccinated kids , then unvaccinated kids should never interact with people .

protect

Â One fundamental question : What is the ROLE of a vaccine ? Â Â To me , a selective vaxer , and to public health authorities , the obvious answer is to protect children from getting and spreading particular diseases .

I can understand vaxing your kid for pertussis to protect your kid , but do n't think by not vaxing for pertussis , you 're going to cause some kind of " pertussis epidemic " or whatever Izobelle meant by " how it would be if in three years , everyone made the same calculation as you .

NEW YORK – Merck ; Co. Inc. Tuesday said U.S. regulators have approved its combination vaccine to protect children 12 months to 12 years of age against measles , mumps , rubella and chickenpox .

And of course , like a PP said , is n't their fully vaccinated child supposed to be protected anyways ? ? ?

I worked in direct care of immumocompromised children , some of whom had too weak of an immune system to respond to vaccines and relied on the immunization of others to protect them , its those kids I think of when I get my flu shot .

* if * i were pro-vax , i wouyld rather have my teenager get hep-b shots than poor lil babies… at least this is the age where some kids are engaging in behavior that would make more sense to be " protected " by this vax…. just rambling , dont mind me.. .

Advantages of the DBO schedule include : 1) it 's simpler , 2) it get 's children protected earlier , and 3) opportunities for shot deferral are minimized .

i also like this article – facts/information do you give people when they say that THEIR vaccinated children are protecting our unvaccinated children from diseases ?

As for the elderly : which vaccine given to a child can protect an elderly person ?

They are protected , in your opinion , so my unvaxed kid should be of no concern .

The reason to not vaccinate is to protect the children , right ?

I do n't think there is anything wrong with not-vaccinating , people make that decision to protect their children just as I choose to vaccinate my child to protect her .

I do n't think there is anything wrong with not-vaccinating , people make that decision to protect

their children just as I choose to vaccinate my child to protect her .

{ quick history : I started researching and stopped vaxing when pg with # 4 so the older kids have varying degrees of vax from all done to doc is still pestering me to " finish the dose " } ANYWAY…. since dd was vaxed against pneumonia (which also claims to protect agasint ear infections) and she HAD pneumonia , I clicked on that one just for fun .

Do you have any resources that could help me learn how to protect my unvax child ?

vacation time Previous thread for flumist : So if you really wanted to protect your child from flu vaccine shedding , it 'd have to be a heck of a lot more than a day or two , unfortunately .

i just had a convo with my grandma the other day (late 70s) and i asked her if she had seen the commercials basically making people feel guilty enough to get a shot " for their grandparent " or " to protect their children " … i told my grandma that she was on her own i was n't getting a flu shot and her response was , " well im not getting one either " Except Theoretica , the evidence shows flu vaccines do not work … And the results of vitamin D supplementation is impressive .

I thought we (my family) were protected from unwanted vaccines when I wrote in big letters on my kids registration forms for school .

Common sense tells me to protect my baby from saminella and ecoli bacteria…so I put a blanket over the seat of the chair in the carts when going out (because some people put their raw meat in the seat of the cart) …my son is n't vaccinated…and we do n't act any differently…just use common sense… TO be honest , the only thing I worried about when my son was a baby (and to a point I still do) was his exposure to recently vaccinated people/children .

One of the pieces basically stated they wanted to vaccinate all children (who have mild to no symptoms) to protect the adults .

damag

Thank you , thank you , thank you for starting this thread ! Â Â We 've never vax 'd any of our kids – due to a strong family history of autoimmune diseases – but over the past winter our youngest developed asthma out of the blue ; he 's been in ; out of the hospital 4 times since December. Â We finally have that under control , but our FP ; I had a few long talks about it and we 've decided to very slowly start vaxing. Â Â I am terrified. Â Our oldest ds has a speech delay and sensory issues , our dd has juvenile RA and I have RA as well and I 'm just so scared that these will damage their immune systems [and mine] down the line. Â I feel so torn because the youngest is only 15 months old now – he 's so helpless in all of this – and we 're doing it for his benefit mostly ; in case we catch anything , hopefully the vaxes will at least make the disease less severe , and thus less likely to land him in the hospital. Â But how do I reconcile that with the damage this might do to my older 2 ? Â I would never forgive myself if something happened to them. Â Â I 'm down with the idea that as our lifestyles change we change along with them , but was it easy or hard for those of you going from non-vax to vax ? Â I 'm having such a moral dilemma about this. Â On the one hand , it 's a no-brainer , on the other if anything happens the fault is 100 % mine for giving the ok. Â I guess that 's the leap of faith we 're taking. Â So thanks for listening , sorry to highjack , and thank you OP for starting this very timely thread !

Homeopathic remedies have been also shown to aid dramatically in the healing of vaccine damaged kids .

I honestly feel it was mothers intuition or something I do feel now the child i was suppose to vax that was in preschool was damaged when she was little now that we look back and I think the next set of shot would have sent her over the edge so I say an angel was looking over us that day .

Seems the only absolute or probable contraindication currently recognized is immediate death) I read accounts of other children who had reacted in a similar way and been left brain damaged or otherwise disabled or had died following this vaccine , some of them with their first dose , others with a subsequent dose .

Another good source of information is the National Vaccine Information Center ( ) . Â They stay very up-to-date on legislation towards requirement of vaccines. Â Their main mission is to preserve our Â right to make infomed choices about whether or not to vaccinate. Â The founder ‘s son was damaged by a vaccination (DPT if I remember correctly) , so there is somewhat of an anti-vaccine slant , but mostly from the standpoint of making the dangers of vaccines known (as opposed to acting like all vaccines are benign , harmless necessities) . Â Â I am expecting my first child in September and have been doing lots of thinking on whether or not to vaccinate/partially vaccinate/etc. Â Thanks to all of you who have shared info so far. Â This has been quite helpful ! !

julie That would be admitting something else they already know , that they have damaged many kids with their poisonous needle . the bacterial meningitis vaccine the only vaccine out there for a bacterial infection ?

The tricky thing here is , did the kids get antibiotics because they already had a problem which was causing illness , or did the antibiotics damage the children and make them vulnerable to the vaccine problem , or was there a combination ?

Â Taking into account my own family history and what I see my child is sturggling with now (he ‘s unvaxed at 2.5 yrs old) , I KNOW had I vaxed him he would have been damaged .

Â Below are some interesting articles recommended to be by Jeanne Ohm , they ‘re meant to be informative not conclusive. Â I also recommend checking out hard data (not opinion based articles) on issues like how other countries vaccinate ; their autism rates (vs the US) , how US autism rates have changed as multiple vaccinations have become more prevalent , the prevalence of ” vaccine-preventable ” illnesses over time in the US ; Europe , the lists of vaccine ingredients on the CDC website (; add them all up to consider 8 + vaccines a visit) and so on. Â Plus just think about (; research) each disease ; what is the actual chance of getting it , the chance of having permanent health probs b/c of it , or the chances of dying vs the consequences of over-vax’ing. Â No one wants to risk their child dying or being permanently damaged , but I feel like the chances of contracting certain diseases let alone dying from are very low in many instances. Â This is overwhelming , I know , I ‘ve been there , but I think that educating ourselves is the only way we can reach the right conclusions for our families. Â I would be interested to know what research would lead a parent to follow the AAP schedule. Â That ‘s just an honest statement , no negativity implied .

And that why it ‘s no surprise that we have so many vaccine damaged kids .

I , for one , will not vaccinate my children since I have had vaccine reactions in the past , and I STRONGLY suspect my handicap sister as being vaccine damaged (she is in her 40 ‘s , still in diapers , still being cared for by my parents) .

When your child has a vax reaction and becomes brain damaged as a result , you tend to stop vaxing immediately .

And now since the boarders and wall has been removed , we see all the vaccine damaged kids .

It is even possible that the child had a damaged immune system from all the vaccines that caused her DS to be sick all the time .

[QUOTE = applejuice ; 11904744] Yes , I know of a doctor (surgeon) who has a vaccine damaged child (diagnosed with autism , but recovered through chelation therapy .

It might help explain why some children are not damaged by a vaccine , while others are .

I personally am not saying that at all , but I am saying that vaccines damaged my child in ways that he NEVER should have been damaged . would prefer not to vax .

I personally am not saying that at all , but I am saying that vaccines damaged my child in ways that he NEVER should have been damaged . would prefer not to vax .

At least in my city , several months ago they aired a segment on a local family whose daughter was damaged by vax ‘s .

I ‘ve spent nearly 20 years fighting vaccine damage cases , and quite a few of those kids would never have been damaged inthe first place , had parents had titres done before-hand .

ar

Â ‘s a collection of quotes from health experts , authors and doctors about how vaccines harm children : Vaccinations do not prevent illness , they cause illness-see link If you want to really know why mass vaccination policies are pure scientific bunk , read this article (and pass it on to others) : It might save your childrens life nak this should be moved to the vax forum .

It does seem as though we have forced out the infectious diseases which are a normal part of childhood and replaced them with chronic diseases , but it would be good to have stats to quote at people who say that children are healthier due to vaccinations !

It does seem as though we have forced out the infectious diseases which are a normal part of childhood and replaced them with chronic diseases , but it would be good to have stats to quote at people who say that children are healthier due to vaccinations !

(I supposed I ‘d consider titers if I were in West Virgina or Mississippi and had to use schools or daycare) -Angela I gave my first vaccination ever to my 13 yo Â son ten days ago (we are moving overseas) – it was the MMR and he woke up this am slightly feverish , burning eyes and on and off nausea.. .

I do n't know much about the source of the article , but found it interesting . 'm wondering : Â On the forums , I found quite a few stories of moms that have vaxed older kids , but regret that decision and are now not vaxing/selectively vaxing/delayed vaxing their younger kids .

This is based on the fact that our lifestyle has changed : I 'm going back to work , the kids are in school/preschool , and in four years ' time , we 're going around the world for a year as a family , and they will need a whole bunch of other vaccines , and I 'd rather space them out a bit. Â Having kids that were not vaccinated Â no longer made sense for our Â lifestyle .

I 'm so sorry about your RA ! Â That totally stinks ! Â I 've been lucky so far to have an easily-controlled type , as does my daughter. Â My son 's asthma , however , is not autoimmune at this point – he has reactive airway-type/non-allergic asthma. Â His is n't triggered by pollen/foods/pets , but by cold air ; occasionally a virus takes a deep dive into his lungs. Â Â Interesting about your bout with mumps. Â I was completely vaxed and had the mumps anyway as well , around age 9. Â I had read somewhere that there 's a correlation between people who are vaxed and do n't show immunity after the MMRs and later development of autoimmune disease. Â I 'll try and find it again .

Will children who are vaccinated now have children of tomorrow with severe birth defects ?

That is , supposedly only among kids who are currently being vaccinated or have had diseases that impart natural immunity .

I hope to expose him to natural pox a few more times during childhood as well , to hopefully help his immunity , and we 'll get him boosters or at the least titers at 10 and before he goes off to college. Â I would prefer for both kids to have good immunity to it when they are older , and be able to travel to England and non-CP-vaxxing-countries without worrying about their immunity to it. Â Â Everything else on the schedule both children got , on a slightly spread out schedule , but they were caught up by age 3. Â At 2 , they were no more " dangerous to community health " than an 11.9 month old who had n't yet had the 12 month old series of shots .

In countries that do n't vax for CP routinely , it 's still a disease of 80-90 year olds. Â My boys are older now-teenagers. Â The shot came out when they were preschool age and it was n't mandatory yet. Â The doctor would ask if I wanted my boys to get it and I 'd say no , because I was hoping they 'd catch it on their own. Â (this is before I learned health reasons not to give shots , etc) Â But they did n't get it and two years later I gave in and said OK. Â If I could do it again , and I found a way to expose them to it , I would for sure ! Â It 's good for them to get it when they 're young , that way they have lifetime immunity and wo n't Â have any chance of getting Â it when they 're older. Â I wished my boys would have got the chicken pox on their own , way back then. Â Chicken pox is n't a big deal to get. Â It 's a pain , but they get over it just like they do colds and the flu. Â Plus it 's better to get it young before they 're in school and have to be out , or miss whatever other activities they may be into as an older child .

Still , I would opt out regardless if your ped is enrolled in the registry b/c these days a child may be entered into the registry at birth (if new mother signs forms at the hospital opting in) , even if no vaccines are entered by a participating provider .

Too many parents are questioning the number of shots their kids are getting on a regular basis .

Too many parents are questioning the number of shots their kids are getting on a regular basis .

And like you said , the immunity wears off (if it ‘s even there to start with , DTaP is n’t a very effective vax to start with) so you ‘re susceptible to it , your DH is susceptible to it , your child ‘s dr is , etc etc etc unless you ‘ve all been keeping up with the boosters (which I think are now or will soon be recommended every 5 years instead of 10) .

I love (not really) how they do n’t like to point out their statistics include people who are malnourished and do n’t receive medical care and/or have neglectful parents) * double post * sorry Yeah , my DD was fully vaxed through her toddler ones and was exposed to chicken pox last year .

huh , this must be the same rule they go by when they tell parents who ‘s children are seizing and screaming after their shots that it ” could n’t be related to the shots ” .

He said it ‘s ridiculous that kids are forced to do Hep B , and he said that it ‘s up to me if I do varicella or any shot and he completely understands if I want to delay .

I understand it is a devasting disease but so are the diseases that children get from being vaccinated .

My two older daughters are fully and partially vaxed .

give

more than likely she ‘s immune anyway , only a few kids really need it but they give it to all kids just in case… the more vaccines a child has injected the more of a chance that there is a reaction or some long term side effects .

Thank you , thank you , thank you for starting this thread ! Â Â We ‘ve never vax ‘d any of our kids – due to a strong family history of autoimmune diseases – but over the past winter our youngest developed asthma out of the blue ; he ‘s been in ; out of the hospital 4 times since December. Â We finally have that under control , but our FP ; I had a few long talks about it and we ‘ve decided to very slowly start vaxing. Â Â I am terrified. Â Our oldest ds has a speech delay and sensory issues , our dd has juvenile RA and I have RA as well and I ‘m just so scared that these will damage their immune systems [and mine] down the line. Â I feel so torn because the youngest is only 15 months old now – he ‘s so helpless in all of this – and we ‘re doing it for his benefit mostly ; in case we catch anything , hopefully the vaxes will at least make the disease less severe , and thus less likely to land him in the hospital. Â But how do I reconcile that with the damage this might do to my older 2 ? Â I would never forgive myself if something happened to them. Â Â I ‘m down with the idea that as our lifestyles change we change along with them , but was it easy or hard for those of you going from non-vax to vax ? Â I ‘m having such a moral dilemma about this. Â On the one hand , it ‘s a no-brainer , on the other if anything happens the fault is 100 % mine for giving the ok. Â I guess that ‘s the leap of faith we ‘re taking. Â So thanks for listening , sorry to highjack , and thank you OP for starting this very timely thread !

I live in NJ and this is what the law states : A written statement should be submitted by the student ,

or the student ‘s parent or guardian if the student is a minor , explaining how the administration of the vaccine conflicts with the bona fide religious tenets or practices of the student , or the parent or guardian , as appropriate ; except that a general philosophical or moral objection to the vaccination shall not be sufficient for an exemption on religious grounds .

That ‘s the last I ‘ll say about it , since as I recall , this thread is in regards to the finer points of submitting a religious exemption in NJ , not debating the infallability of the Catholic Church .

Yes , I am good with just submitting a religious exemption – was just wondering if there was a form that would be best .

However , if the parent submits an exemption , then the vax record is not needed by the school .

We even began kindergarten a few years ago with a titer check waiver for the measles and then submitted an exemption two years after that .

They did n’t even request , nor did I have to submit , an exemption for anything .

Even if the Church told all Catholic parents not to let their child get the MMR for instance , most parents would have to still be required to submit a religous exemption which would exempt all vaccines .

Were it not for the diocesan rule , parents could choose not to have the vaxes with aborted fetal tissue (or no vaxes , or whatever) , file an exemption , and submit the exemption to the schooln (although the school could refuse to accept it pursuant to the Texas AG ‘s opinion stating that private schools do not have to accept exemptions ; although I strongly feel that if anyone ever mounted a serious legal challenge on this point , the courts would come to a different conclusion on this issue) .

I have written and submitted our religious exemption form for all of his vaccinations to the school and am awaiting their decision .

My kids are partially vaxed and we just submitted a religious exemption to the school she will be attending this fall .

I would submit your exemption without prior vax history when you register for preschool and again for kindergarten .

Please let us know where you live so that we can give you the most accurate information on how to submit your exemption .

My now 8 yr old is in 2nd grade , same as my 12 yr old , his exemption was submitted with his initial school enrollment package . seen it on the health call on fox news this morning I think he was saying how its easy for kids…And how the virus is so weak..Well I dont want my lo getting the flu a weird strain beacuse of this beaing spred by some little kid that cant keep his fingers out of his nose beacuse thats what kids do pick there nose then touch everything its BS and really @ # ^ % $ # % $ makes me MAD…..I hope someone can find the link to this on fox…….I am mad now. . you for posting this paper .

I just submitted our religious exemption .

However , if the school is not aware of a child ‘s vax status , then I would suggest not bringing it up and just submit the exemption w/o any immunization records .

We also had to give parents the exemption forms if they dind’t want to submit VAx records .

We will be in NY , and have filed the ” religious ” exemption , and submitted the school ‘s forms filled in by our doctor , but I imagine there are plenty of families at the school who selectively vaccinate , so I will have to see what they say .

i intend to submit an exemption regardless (we are in NH and can do the religious exemption) .

But I heard that if a child is going to join a sport club or other activities , people will ask them to submit the exemption form .

accept

I do n’t know where they get the idea that there are only 3 types of religions they can accept for an exemption to vaccines .

Just like anywhere else , as far as I know..if the hospital recieves any kind of federal funding they must accept your exemption just as a school would .

I talked with my dh tonight , and he said that the school is going to accept the religious exemption !

I said , ” but you will accept an exemption , right ?

Were it not for the diocesan rule , parents could choose not to have the vaxes with aborted fetal tissue (or no vaxes , or whatever) , file an exemption , and submit the exemption to the schooln (although the school could refuse to accept it pursuant to the Texas AG ‘s opinion stating that private schools do not have to accept exemptions ; although I strongly feel that if anyone ever mounted a serious legal challenge on this point , the courts would come to a different conclusion on this issue) .

Were it not for the diocesan rule , parents could choose not to have the vaxes with aborted fetal tissue (or no vaxes , or whatever) , file an exemption , and submit the exemption to the schooln (although the school could refuse to accept it pursuant to the Texas AG ‘s opinion stating that private schools do not have to accept exemptions ; although I strongly feel that if anyone ever mounted a serious legal challenge on this point , the courts would come to a different conclusion on this issue) .

I talked to the director of the CDC (child development center) and the director of the family childcare program and both told me they do not accept any sort of exemption .

Here in MS , where we only have a JOKE of a medical exemption available to parents , the state health department will accept titers on all diseases except DTaP .

I am in CO , not CA , but CO has the three exemptions like CA , and two of my children go to private schools , neither one has any problem with accepting our exemptions , we are given the same vax [exemption] form to fill in as the public schools .

The only reason why I did n't – the nursery program was private and accepted my exemption without batting an eyelash , so I did n't want to rock the boat by refusing the " examination .

I know for a fact that the school accepts another child who has a religious exemption , so I 'm not too worried about that angle (well–I 'm somewhat worried or I would have done it by now) .

I am not too worried about the school , but do n't know if they 'll accept the exemption without any medical records , and of course it 's on the same piece of paper .

I reiterated his request in an email , and this is what I recieved : " I spoke with AETC and they requested the following from you in order to make a determination on whether we can accept an exemption from the State of Texas for required immunications : A statement of why you requested the exemption A copy of the exemption from the State that lists the immunizations that are exempt Any statements from the medical advisor of why the exemption is suggested or necessary , if any I will forward this information to AETC and they will provide us with an answer as to whether we can accept the exemption at the CDC .

I reiterated his request in an email , and this is what I recieved : " I spoke with AETC and they requested the following from you in order to make a determination on whether we can accept an exemption from the State of Texas for required immunications : A statement of why you requested the exemption A copy of the exemption from the State that lists the immunizations that are exempt Any statements from the medical advisor of why the exemption is suggested or necessary , if any I will forward this information to AETC and they will provide us with an answer as to whether we can accept the exemption at the CDC .

I know the schools have to accept a religious exemption but does a daycare ?

I also handed the principal a copy of the NYS guidelines for accepting religious exemptions found here : Somewhat off topic , but does anyone know if Synagis would fall under the category of exemption based on the above definition ?

I had put off calling because I was fearful I would n't find a place that would accept our exemption .

I would think since they receive funding from government that they would HAVE to accept your exemption and keep your coverage .

She finally says , fine , okay , she will accept the exemption form that I had gotten from the health dept before I got there .

When I responded with the religious exemption , I was told that they can not accept exemptions , even religious state certified ones .

requir

Since NJ statutes require the parent to explain their religious beliefs , even public schools in NJ can reject an exemption .

I thought you are only required to get an exemption if your child is attending school .

/hijack off , sorry went to my local WIC office today (colorado) to set up an intake appt and when she got to bringing in dd 's immunizations record I said " We do n't vax will I need to bring in a philosophical exemption " and she said " no thats fine just tell your counselor and she wo n't require that record " YAY…. now I 'm hearing horrible things from my friend about the ped I was going to take dd to so we might have to shop around for a GP… . 'm leaving in a few hours to visit DH 's dad for Thanksgiving and I 'm very concerned about the possibility of my unvaxed child catching hep B from him .

We are planning on handing in a religous exemption for the required vax sheet .

: Here is the link to the TX exemption information , although I 'm not sure maybe they can require you to get certain vaccines for medical programs .

Even if the Church told all Catholic parents not to let their child get the MMR for instance , most parents would have to still be required to submit a religous exemption which would exempt all vaccines .

I was n't clear on your posts as to whether you plan on exempting your child completely , but wanted to make you aware just case you are n't using an exemption that in Florida four (4) doses of a pertussis-containing vaccine are required unless the 4th dose is given on or after the fourth birthday .

Since you are pressed for time , he can email you a " crash course " guide to writing a personal religious exemption in NY (since the law requires us to explain our beliefs) .

As far as I know there is no regular waiver or exemption for vaxes for immigration because they are not required .

When we applied for Medi-Cal and Welfare I was required to bring in their immunization cards as well and I printed out the school exemption form and signed it as requred by law (i also had the law quoted on the bottom of my letter of exemption) and brought that in instead of their card .

Oregon does not have a Philosophical exemption – only medical and religious That said , I can not find anywhere that the Flu vaccine is required for public schools/daycares .

In fact , the rules include a provision that requires all child care facilities and elementary/secondary schools to use the official MDH record form or a similar document approved by MDH when communicating to parents regarding immunizations requirements and exemption procedures .

I reiterated his request in an email , and this is what I recieved : " I spoke with AETC and they requested the following from you in order to make a determination on whether we can accept an exemption from the State of Texas for required immunications : A statement of why you requested the exemption A copy of the exemption from the State that lists the immunizations that are exempt

Any statements from the medical advisor of why the exemption is suggested or necessary , if any I will forward this information to AETC and they will provide us with an answer as to whether we can accept the exemption at the CDC .

i ‘ll be homeschooling , but i ‘m doing it under an umbrella school (same one i graduated from) and since they ‘re registered as a ” private school ” they require a health exam and up to date vax (or religious exemption , which i ‘ve had for her for a couple years .

The provisions of this chapter shall not apply if : (1) In the absence of an epidemic or immediate threat thereof , the parent or guardian of the child shall object thereto in writing on grounds that such immunization or testing conflicts with his religious tenets and practices ; or (2) Certification by a competent medical authority providing individual exemption from the required immunization or testing is presented the admissions officer of the school .

Â The exemption form does state that it is good for two years. Â But am I legally required to honor that statement ? Â Does the law give the state health department the right to make me get it again ? Â I tried looking through the language of the law , but I ‘m confused as to whether I ‘m reading actual law or interpretation of the law… Â This is what I ‘ve been piecing through : Â Â If you have any insight on this , please let me know. Â In case you ‘re wondering , this is merely a matter of principle. Â The exemption is really easy to obtain .

Can we get a religious exemption as our state (VA) requires if we ‘re doing only some of them or would we then be required to ” catch up ” ?

The next thing I want to mention is , as previously posted , there is no reason for the ped to require an exemption form .

Here is my predicament – since this is a private camp , I have no legal right to require them to accept a religious exemption .

They are required for school in our state , but if I got with the Philosophical exemption I plan on just claiming he never had any shots .

attend

I was lucky enough to have my dds attend a private preschool where the owner was very respectful of everyones differing opinions and did n’t give us any problem as long as she had the exemption to show the state .

I thought you are only required to get an exemption if your child is attending school .

We have attended two super traditional , mainstream Baptist preschools and have n’t had a bit of a problem with an exemption .

My kids are partially vaxed and we just submitted a religious exemption to the school she will be attending this fall .

In my state,I can not get an exemption for my son.They are not given unless a child is enrolled in

school or daycare.I will need one if he attends preschool next year (I ‘m considering this,depending on where he will be placed) .But,if you can get an exemption in your state,definatly do it.Just to CYA .

i believe it is required by law to attend school which a parent could then get an exemption , so are they asking if it should be the law that it is or should it just be law period ?

They are under no obligation to accept a child who presents an exemption . ‘re currently homeschooling but are looking into a Catholic school for our two oldest to attend after we move .

Anyway , do I have to do anything special as far as an exemption form since he wo n't be attending school ?

It would not be because the government told me I had to or my child would need an exemption to attend school .

Even though NC has a religious exemption – your doc will just administer the shots you request (if they are willing) and then you just do not report them to anyone . my DD refused to go back to preschool , so she is not going , this year , but i was wondering since next year she will be attending a different school i will need another exemption form , so do you think the school will give me back her exemption form ?

The same day care my unvaxed kids attended with a religious exemption that they did not even question " forced " a mom I know to do vax to be admitted at all .

Thanks LI , yes all of them will be attending the same school , and actually our parochial school is closing so it will be a different one , but I was told DD ‘s records go with her and no need for another exemption .

continued I ‘m not sure if they can legally require proof of vaccination or exemption in your situation , but if they do , this is all you need to write (includes statute language) : My homeschooled child , [FULL NAME] will be attending [NAME OF SCHOOL] for certain activities , including , but not limited to , music and art and I am submitting this letter in order to satisfy state immunization requirements for school entry .

Then , when I was talking w/ him he told me that in Massachusetts , if I want DS to attend school , he ‘ll need to be up to date w/ required vaxes , that declining vaxes is only possible w/ a religious exemption or a medical exemption .

You only need to have an exemption if your child will be attending daycare or school.My dd was fully vaxed until 2.5 years.I used a religious exemption for her to start kindergarten this year (we pulled her out for many reasons later) .You do n't have to tell anyone what your child has already had.I never told them,but if they had found out I would have explained that I had changed my religious beliefs.I hope this helps ! !

I also wonder what effect mixing the SA with another acidic material (citric acid in OJ , for instance) would have on its efficacy , as you would likely get cross-reactions . unvaccinated 21 month old daughter will be attending preschool in a few weeks , and I plan on submitting the religious

exemption form for the state of CT .

Luckily , DD is attending school in a state WITH a philosophical exemption , so I did n't have to lie in order to get her out of the Meningitus vaccine .

I have an exemption form in 2 counties here in Ontario saying that my teen will not be vax 'd for reason of conscience so she can attend school .

But when I went ; downloaded the exemption form it was basically a check box that seemed only to apply to school attendance (my child may not attend if there is an outbreak bla bla bla) .

So if I go to the doctor for other matters , and I do not attend church (for example – if I were still CS) I would have a hard time making the exemption stick .

file

I know some people think that we should not sign the waiver , but to me it is no different than when I file the exemption form for school .

The main point to understand here is that these parents are n't anti-vax , on top of their paperwork , and still getting picked on by The Man to get shots … this is all happening because the parents did n't file the paperwork , be it a vax record or an exemption .

3 fewer vaxes she COULD receive if i vax . was just wondering " when " I should file my non-vaxing exemption form with the state ?

I did n't know if I should keep my choice of not to vax out of the system for as long as I can , or if I need to file my exemption now .

What I 'm wondering is should I file my exemption " NOW " or wait until they are of " school age " ? ?

But what I meant was you should find out if you need to file an exemption if you are homeschooling .

There has to be some way around that (otherwise , " they " are forcing selective vax parents to not vax at all , or do all vaxes) I 've read it in several places , basically once you have filed the exemption form , that 's it .

If both parents want to claim religious beliefs against vaxing , then the active duty spouse would have to file an exemption for him/herself .

I 'd file an exemption with the preschool .

Yes , the reason for getting it would just be so he 'd be 'complete ' , and I would n't have to file the exemption form for 1 shot .

I think I 'm going to go ahead and just not get it and file the exemption .

In that case – I think personally I would file the exemption .

Â I ‘ve done both , so I can assure you that in some states it ‘s actually easier to just file the exemption .

I would think you can file a religious exemption .

I ‘d file a religious exemption : United States ; Code of Federal Regulations ; Title 29 – Labor ; CHAPTER XIV–EQUAL EMPLOYMENT OPPORTUNITY COMMISSION 29 C.F.R .

My then 4 yr old was only due for boosters , but I declined them , and filed a religious exemption when I enrolled him in Kindergarten .

Hi , I am also in Florida (Broward County) and have filed a religious exemption .

You can ” find ” religion at any time -Angela That is good because I am filing a religious exemption this year for my 8th grader .

I am by no means an expert , but I believe you will have to file a religious exemption , and they ca n’t not cover you based solely on that .

” I would send the exemption back stating that you want it filed with your child ‘s records .

need

I just got a letter home from her school saying she needs her shots or an exemption filled out before the 27th or she can not come back .

They are going to have to work a lot harder to change my mind . I need to have a Clergy member sign the exemption form ?

Yet another tetanus from splinter case documented on CDC (again , not a child) : I also find it interesting that they spent so much time documenting the exemption , when it would appear that besides having an infected wound that needed to be expressed and cleansed properly , he was apparently healthy for 2 weeks prior to : Wait , so FIRST they give tetanus toxoid and antibiotics and send the boy home , before removing the splinter and expressing the pus to properly cleanse the wound ?

I asked her what I needed to turn in if my son had already received some vaccinations but I have since obtained a religious exemption .

We ‘re already ” behind ” , and without a philosophical exemption available in Maryland , I feel like I need to know NOW whether a religious exemption is ” do-able ” and how I ‘d go about it .

For this next drs visit I ‘d tell your dr that you ‘re in the process of getting the exemption so there ‘s no more need to discuss it , thanks though .

However , if the parent submits an exemption , then the vax record is not needed by the school .

” I ‘ll pass it along that the family just needs to sign a religious exemption .

This is just what I have thought of right now so there may be holes in it but this is something of what I would probably use as my religious exemption if I needed it .

/hijack off , sorry went to my local WIC office today (colorado) to set up an intake appt and when she got to bringing in dd ‘s immunizations record I said ” We do n’t vax will I need to bring in a philosophical exemption ” and she said ” no thats fine just tell your counselor and she wo n’t require that record ” YAY…. now I ‘m hearing horrible things from my friend about the ped I was going to take dd to so we might have to shop around for a GP… . ‘m leaving in a few hours to visit DH ‘s dad for Thanksgiving and I ‘m very concerned about the possibility of my unvaxed child catching hep B from him .

I did n’t know if I should keep my choice of not to vax out of the system for as long as I can , or if I need to file my exemption now .

But what I meant was you should find out if you need to file an exemption if you are homeschooling .

Also , he is 7 months old , exclusivly breastfed and has never been vaxed against anything and has never been sick – does any of that matter ? everyone , I need a religious exemption form for California for my son tomorrow .

I just want to clarify for morgan ‘s mom that , because she lives in Texas , she can ” pick and choose ” using the personal/religious exemption . is all so much for me to handel,every little sickness : out there every vax.I try to feel good about not vaxing and I just feel overwelhemed.I truly think that OCD has taken over my life since I got into not vaxing,I have become a weirdo,and all the info out there is to much to take.I belive that not vaxing is healthy yes you dont get all that garbace into your system.I also belive that the vax companys are out for $ $ $ $ but I also belive they want to protect us to ….And my Family thinks I am crazy for being able to prevent someting and not doing it..I really feel pulled in both derections and I dont want my lo to be one with a vax reaction,and I dont want my lo be one with a vpd..I feel I have no one to talk to this about,my mom acts bipoler,my DH is a BLEEP that just tells me do what you want…Vaxes I dont think are needed Depthira,Tetnus,Polio,Hep B,Hep A,MMR,Ones I would feel safer with but scared of the reactions would be Pertussis,Hib,and I dont know about Prevnar the ones that are probbly the most reactive,I truly feel like I am turnning into nut case and I really dont think I could get a shot for my lo,beacuse of the what if reactions….I just want to run,and run ,and run until I pass out and then go to the bar and get drunk and forget about all this stress of worring…I had to vent Momma , I ‘m sending you a big ol ‘ psychic hug .

Are there any other Canadians/Albertans out there who have used an exemption and if so , do I need a specific form from somewhere ?

I dont know about your state but I would start by telling the school that you just need the exemption form .

It seems that they are also making it easier for parents to get the things they need for an exemption in general : I am still very much against this bill and will continue speaking out against it to anybody

that will listen .

I think the only time you would need to obtain an exemption form would be to offer to an organization that is requesting a vax record (or exemption) .

And really why the hell should we need to get an exemption for a parenting choice , it ‘s backwards and quite frankly messed up .

I thought I needed to get the county health dept exemption before this next check-up .

enrol

I enrolled my daughter into a Catholic pre-school in NJ and turned in a religious exemption…about a month later I received a letter from the principal stating ” the Bishops of New Jersey have mandated that requests for religious exemption from the requirements for immunizations will not be granted ” .

I ‘ve heard that you can not do a selective schedule and still enroll your child in public school with an exemption .

I plan to get my exemption tomorrow since my son will be enrolled in daycare in the next few weeks .

We ‘re in the process of enrolling DS in a christian private school and have had no problem with our religious exemption .

My then 4 yr old was only due for boosters , but I declined them , and filed a religious exemption when I enrolled him in Kindergarten .

Here ‘s something else I found from John Hopkins : Take note about what it says regarding nursing mothers . have never ever had any issues with enrolling my daughter in any school in all of the states we lived in before (including military schools) , using a vaccination exemption .

But , when it comes time to enroll him in kindergarten in the fall , we have our exemption ready .

If I am correct , you could possibly enroll your child into a private school and print yourself up a nice religious exemption of some sort .

If you decide to send her to school – do n’t worry about your exemption ! I ‘ve been homeschooling my two kiddos and they ‘ve been talking about the option of enrolling in a public e-school next year .

In my state,I can not get an exemption for my son.They are not given unless a child is enrolled in school or daycare.I will need one if he attends preschool next year (I ‘m considering this,depending on where he will be placed) .But,if you can get an exemption in your state,definatly do it.Just to CYA .

4) Ds is not (currently) enrolled in daycare but is watched at home where his risk for contracting

stuff is much lower 5) It is against our religious beliefs (VA has a religious exemption that I just need to get notarized) ——————– The only little snafu is that we are going to be starting him in d/c 3 hours a day and they need a letter of general good health from the ped .

She made all sorts of disagreeable noises from the moment she deduced my son was on an exemption (” oh , well , I do n't know about THAT..We have a pg teacher here and , blah , blah , blah ”) I had to enroll him the first day and she could n't say didly about his legal , notarized exemtion (though I could tell she really wanted to !

Even back when I did n't have any issues with vaccines and had n't even thought to do any research , it did n't make sense to give a chickenpox vaccine . 'm in Ohio and my oldest is 4 and so I 've been looking at enrollment forms for school and I noticed on the Immunization Exemption form for the box Religious/Philosophical Objection it says I have to explain. Â I was under the impression they were n't allowed to ask my religion. Â So what do you put there and have you had any issues with public schools accepting it ? Â Thanks Â I 'm enrolling DD in private school (which in my state has to take exemptions) . Â I simply did n't answer the question and wrote : ” Pursuant to my legal right under State Code XYZ , I am claiming a philosophical exemption from the Hep B vaccine .

I enrolled him in school with a religious exemption so he never got another shot .

Things may be slightly different for you because you have enrolled your kids in a private setting which gives them more control and power , but I still think that in the end legally you can claim whatever you want regarding religious exemption , whenever you want .

While you do n't have to write your exemption explanation until your son is ready to be enrolled , it helps to write a quick statement that ” we have religious objections to the practice of vaccination ” and give that to your new doc to put in their file .

Those requirements being : in order for a child to remain enrolled in a licensed setting , a immunization record or an exemption must be provided by the parent in order to comply with public health law .

If I did n't see one , I 'd enroll my child and submit the card the state requires be followed , filling out the exemption portion .

When it came time to enroll my son in public school , I simply checked the religious exemption box on the form and turned it in .

We live in NH. Â Although we 'll be homeschooling our daughter this September we originally enrolled her in public school in April. Â It was incredibly easy to get the religious exemption form. Â I downloaded it , brought it to a notary public- it was at a postal store in Manchester , and she just signed it after looking at my ID .

sign

I know some people think that we should not sign the waiver , but to me it is no different than when I file the exemption form for school .

” I ‘ll pass it along that the family just needs to sign a religious exemption .

(long story) I signed a religious waiver exemption and let the daycare provider know that I would like to be informed when another child at the center (she will be in the Infant Toddler room until Oct.) is vaccinated and returns to center same day which apparently happens quite a bit .

My dd has all but varicella and we still signed the exemption saying she ‘s unimmunized . 18 mo ds is fully vaccinated minus one we delayed because he was due for 3 shots at once .

Have the parent who did n’t sign the exemption form take the child in .

The church had the exemption forms , and we had to pick one up , and probably have it signed by the clerk to verify that we were Christian Scientists .

When we applied for Medi-Cal and Welfare I was required to bring in their immunization cards as well and I printed out the school exemption form and signed it as requred by law (i also had the law quoted on the bottom of my letter of exemption) and brought that in instead of their card .

When signing the vaccination waver form for the ped ‘s office I did not write ” Religions exemption ” Instead I wrote something about doing research on side effects .

I have absolutely no problem signing my religious exemption at all .

2) I believe KS has either medical exemption (no dr here will sign off from what I hear) or religious exemption .

I ‘m trying to find a new pediatrician… Do I need to sign a form from them , just refuse vac ‘s , or do I need to get a religious exemption to show the doc ?

I have questions…. thanks mamdawg I ‘m definitely not signing anything I had to learn the hard way.Our son is vaccine damaged with seizures.You do n’t have to be on the defense,well researched,or particularly articulate.JUST DO N’T SIGN ANYTHING ! I would get an exemption form (religious or philosphical) to hand to whoever to keep on file.If I had done this 5 babies ago I ‘d have saved ourselves a lot of suffering and grief .

So….when she goes to sign up her kids for school , she only needs to bring the religious exemption (which also happens to be blue) and the yellow physical form from the doctor ‘s office . all , Well a mum on one of the parenting sites I ‘m a member of said this when asked ‘why ppl chose to or not to vaccinate ‘ She knew it was going to be a hard decision to make as when she was in hospital there was a little boy who had had a bad reaction to the chicken pox vaccine and had to be hospitalised and almost died .

I ‘ll tell you that in my case I was able to find a pediatrician who would not insist on vaxing , and who agreed to sign a form for me that said I had a medical exemption (based on my true story about ds ‘s bad reaction to his two-month round of vaccinations) for preschool .

I will start looking for someone to sign a medical exemption right now .

So , am I lying by signing a religious exemption for school ?

I so wish I had takend my religious exemption out and said this is the only document I will sign in regards to vaccinating my children .

38) RELIGIOUS EXEMPTION FORM Religious exemption requests must contain the wording found in the Alaska Administrative Code [4 AAC06.55 (b) (3) or 4 AAC 62.450 (c) (2)] stating all vaccines must be received unless the child â   has an affidavit signed by his [4 AAC 62.450 (c) (2) says â   the child â   s â   ] parent or guardian affirming that immunization conflicts with the tenets and practices of the church or religious denomination of which the applicant [4 AAC 62.450 (c) (2) says â   the parent or guardian â   ] is a member .

I have my exemption in my children ‘s files and it would probably lessen the liklihood of your ped asking you to sign the Refusal to Vaccinate form .

I ‘m pretty sure my mom signed the exemption forms after that , because I do n’t recall ever receiving another vax .

Ronna If they do n’t provide the school with a vax record , they must submit the Virginia religious exemption form – no way around it .

However , if the parent submits an exemption , then the vax record is not needed by the school .

If it ‘s a scenerio where they are submitting the exemption , but the school knows the child was previously vaccinated and wants the record , then they can submit both the vax record and exemption .

If it ‘s a scenerio where they are submitting the exemption , but the school knows the child was previously vaccinated and wants the record , then they can submit both the vax record and exemption .

Were it not for the diocesan rule , parents could choose not to have the vaxes with aborted fetal tissue (or no vaxes , or whatever) , file an exemption , and submit the exemption to the schooln (although the school could refuse to accept it pursuant to the Texas AG ‘s opinion stating that private schools do not have to accept exemptions ; although I strongly feel that if anyone ever mounted a serious legal challenge on this point , the courts would come to a different conclusion on this issue) .

I have written and submitted our religious exemption form for all of his vaccinations to the school and am awaiting their decision .

If your child will be attending a center (school) based program , then you will need to submit an exemption .

My kids are partially vaxed and we just submitted a religious exemption to the school she will be attending this fall .

I would submit your exemption without prior vax history when you register for preschool and again

for kindergarten .

My now 8 yr old is in 2nd grade , same as my 12 yr old , his exemption was submitted with his initial school enrollment package . seen it on the health call on fox news this morning I think he was saying how its easy for kids…And how the virus is so weak..Well I dont want my lo getting the flu a weird strain beacuse of this beaing spred by some little kid that cant keep his fingers out of his nose beacuse thats what kids do pick there nose then touch everything its BS and really @ # ^ % $ # % $ makes me MAD…..I hope someone can find the link to this on fox…….I am mad now. . you for posting this paper .

Has anyone had their kids denied admission to a private school after they submitted a religious exemption ?

However , if the school is not aware of a child 's vax status , then I would suggest not bringing it up and just submit the exemption w/o any immunization records .

We will be in NY , and have filed the " religious " exemption , and submitted the school 's forms filled in by our doctor , but I imagine there are plenty of families at the school who selectively vaccinate , so I will have to see what they say .

For off base schools you do not have to vaccinate just submit a phlosophical or religious exemption .

I 'm simply curious b/c I submitted a written exemption to our preschool (that accepts unvaxed children and is funded) but they kicked it back giving me one day to fill out and have a form notorized .

Not sure about MA regulations , but I 'm sure there is some type of exemption you can submit to the daycare center .

In our school , the only ppl who know SHOULD be the school nurse , the school superintendent , and the school board (we had to submit our exemption to the super , through the board) .

That said , I would n't tell a school you 're going to vaccinate when pressed for an immunization record and then submit a religious exemption the next day .

Has anybody sucessfully submitted a religious exemption to an Orthodox Jewish school ?

The time is steadfastly approaching when I must submit an exemption letter for my daughter in order for her to stay in the local public school PreK program .

file

I know some people think that we should not sign the waiver , but to me it is no different than when I file the exemption form for school .

What I 'm wondering is should I file my exemption " NOW " or wait until they are of " school age " ? ?

I 'd file an exemption with the preschool .

If you file a religious exemption for school , but it ‘s not in your peditrician ‘s records , that does n’t matter .

We will be in NY , and have filed the ” religious ” exemption , and submitted the school ‘s forms filled in by our doctor , but I imagine there are plenty of families at the school who selectively vaccinate , so I will have to see what they say .

My girls just started preschool 2 weeks ago and we filed the religious exemption with no questions asked .

What I ‘m wondering is if I will have to file some kind of exemption for each vax we do n’t do for the school system if we decide not to homeschool when he ‘s old enough ?

The only way I can think of to maybe pull that off is to file the exemption with your school , then vax as you choose to vax with your doctor , and not give the school the vax record… but I doubt that would work because I ca n’t imagine you could tell the school that you are not giving them a vax record because there is nothing on it and have them take you at your word…If I were the school I would want to see the vax record , blank or not .

(They wo n’t be going to school [hs] , so we wo n’t be filing anything with the school district , so I ‘m thinking the exemption will just go with my homeschooling records ?

For the record I am actually am an atheist so I obviously have no religious beliefs against vaxs , but is ever questioned , ” my religion is private to me and I wish not to discuss it ” is my answer. Â My dd will be starting at a private Catholic school in the fall. Â I Â called and spoke with the nurse about vaccination exemption before we sent in the first check. Â She said that they would accept our Â exemption. Â They do n’t have to accept it , I do n’t think , because they do n’t accept any funding from the government. Â They take no money , no food , etc. Â The whole school is tuition Â funded. Â So they Â are allowed to exclude people. Â We will file a religious exemption because Â that is what our state allows. Â I have heard of Â Catholic schools refusing exemption letters before. Â Â Here is what makes me nervous. Â The school we are looking at is in the midst of new leadership. Â they have an interm principle that is leaving and two nuns are coming. Â It makes me nervous to commit to a year ‘s tuition w/ this change. Â What if the nuns come in and decide no exemptions ? Â The school nurse will be the same. Â Registration is this week , and I have to bring my exemption w/ birth cert in lieu of vaccine records. Â If they accept us , and we sign a contract with the exemption on file , can they change their minds mid year ? Â I would HATE to lose that money. Â I suppose it ‘s possible , but very unlikely .

I sent my DH with my son , and had told my DH I ‘d rather the doctor left off what vaxes our son already had , in case the school get in a huff about why he had some , but not all the vaxes , yet we are filing an exemption .

Contact the school office and ask them to help you file an exemption .

I have n’t filed the exemption with the older children ‘s schools yet .

– Do I have to file a vax exemption even though the DC wo n’t be in school ?

! completely non vaxed kids are 2 and 4. Â As we start to look at public Kindergarten for my son , I 'm looking over the forms. Â Â I know I 'll probably just state my religious beliefs and get an exemption based on that , but I ca n't help thinking it 'd be SO much easier to just fill out the form as it 's " supposed " to look and sign a Â non existant Â doctor 's name. Â I live in a metro area and I ca n't imagine they call all the doctors on all the kids that come into the school. Â I imagine they just check to see that the form filled out and they file it. Â Â NO ONE knows we do n't vaccinate. Â We have n't told friends or neighbors or any one. Â Â And if we were caught , so what ? Â To sign a name for someone that does n't exist is n't forgery. Â And , there 's no law saying you have to tell the truth on school forms. Â Overall , the only places you legally ca n't lie is under oath in a court of law. Â Â Any lawyers out there ? Â Whadya think ? Â Personally Â I would not do that – unless totally backed into a corner and felt I had NO other options whatsoever .

) Â Â But , I guess it 's just an academic question since I will file for an exemption based on my religious beliefs. Â I agree with what others said , that the lie would have to be perpetuated for 12 years and what about if my son started saying the truth when it comes up in science class or wherever. Â He 'd probably be the one to spill the beans. Â It might not be so big a deal .

Cate I have n't filed with the school yet but I 've found the form and letter to be pretty simple here in KY , though it 's really strange they wanted our doctor or the health dept to sign the exemption form – I mean what do they have to do with personal religious beliefs beyond trying to argue against them ?

I researched just for a few days , quoted some state and federal laws regarding the exemption pertaining to our state , and i learned some things , like athesim is considered a religion ie athetist can file religious exemptions and that only one parent has to be 'religous ' to file the claim which is good because dh is an atheist and I loosely follow unitarian beliefs… Â but i would n't lie about being vaccinated for things that ds or dd is not , i do however forgo that ds has been vaccinated for the first 9 months as it would bring up a lot of questions of his partial vax status and i do n't want to go into that.. Â Would I knowingly violate the law , in order to receive public school benefits ? Â Nope. Â There 's even less of an excuse if you have a religious exemption available to which you adhere. Â The schools are required to monitor the vaccination status of children under their care , and you 'd be interfering with that , and for no good reason .

I researched just for a few days , quoted some state and federal laws regarding the exemption pertaining to our state , and i learned some things , like athesim is considered a religion ie athetist can file religious exemptions and that only one parent has to be 'religous ' to file the claim which is good because dh is an atheist and I loosely follow unitarian beliefs… Â but i would n't lie about being vaccinated for things that ds or dd is not , i do however forgo that ds has been vaccinated for the first 9 months as it would bring up a lot of questions of his partial vax status and i do n't want to go into that.. Â Would I knowingly violate the law , in order to receive public school benefits ? Â Nope. Â There 's even less of an excuse if you have a religious exemption available to which you adhere. Â The schools are required to monitor the vaccination status of children under their care , and you 'd be interfering with that , and for no good reason .

You can file a medical (practically impossible to get) or a religious exemption in Florida , but you have to not vax at all to file for school .

claim

You just claim the exemption with the school system and leave it at that .

They are required for school in our state , but if I got with the Philosophical exemption I plan on just claiming he never had any shots .

So if her daughters vax records are being sent as part of the previous records from the other school she can still claim exemption ?

Can I still claim an exemption for school ?

First , because if you ever move to another state that does n't have a philosophical exemption , only a religious one , they may give you a hard time after seeing school transfer records showing your philosophical exemption and you are now suddenly claiming religious convictions .

If it were true that a private preschool COULD deny her because we had claimed an exemption , I really think that would be in writing somewhere , or at least some reference to something like that .

Some states make it easy for parents to claim an exemption by simply signing a prewritten statement on the school immunization form .

It ‘s just that , for school , we do n't really want to claim religious exemption because we are not religious at all and it feels really disingenuous to do so .

So I wrote up a letter stating dh and I were dd ‘s parents/legal guardians and we were claiming a religious exemption based on PA school code # # # .

She also needs an epipen , so if I claim an exemption would they NOT give her the epipen if ever needed at school ?

Even back when I did n't have any issues with vaccines and had n't even thought to do any research , it did n't make sense to give a chickenpox vaccine . ‘m in Ohio and my oldest is 4 and so I ‘ve been looking at enrollment forms for school and I noticed on the Immunization Exemption form for the box Religious/Philosophical Objection it says I have to explain. Â I was under the impression they were n't allowed to ask my religion. Â So what do you put there and have you had any issues with public schools accepting it ? Â Thanks Â I ‘m enrolling DD in private school (which in my state has to take exemptions) . Â I simply did n't answer the question and wrote : ” Pursuant to my legal right under State Code XYZ , I am claiming a philosophical exemption from the Hep B vaccine .

My younger daughter may be going to preschool next year and I am either going to have to immunize or claim a religious exemption , and I want my thoughts together on the topic so I can advocate for her .

Many times , dealing with cdc and schools , you ‘ll have to take paperwork with you stating why you claim an exemption because they just expect everyone to fall in line with what the majority of the people are doing .

Parents who have n't vaxed at all that I have spoken to say that you could probably get away with doing the vaxing you want to before school , then claiming a change of heart and taking the

exemption , but they thought that this would only work for the first child , and that you probably could n't get away with it for the second child .

: You can claim religious exemption – you just never report to school , etc .

My mother wrote a curt note back reminding the nurse of the back of the vaccination form for the schools in California which requires only a check mark that states you are claiming a philosophical exemption for vaccinations .

If the school has no way of knowing what vaccines you are selectively giving , then you can get away with claiming a religious exemption .

My thought was to just keep my mouth closed on the subject until it came up and casually claim my exemption but most of these schools are expensive and require a down payment , which I do n't want to lose .

The question is whether or not the schools will have access to them and will catch you if you claim an exemption and still do some vaccinations .

Neither of us have received any vaccines at all , and it was my thinking that she would be able to claim the same philophical exemption that she used in elementary/high school .

have

~ Religious Exemptions The Wexler Decision To find out what exemptions are offered by your state (for school and such) check here : All states except West Virginia and Mississippi offer religious and/or philosophical exemptions , and the supreme court ruled that you do not have to belong to a non-vaxing religion or have clergy sign your religious exemption .

~ Religious Exemptions The Wexler Decision To find out what exemptions are offered by your state (for school and such) check here : All states except West Virginia and Mississippi offer religious and/or philosophical exemptions , and the supreme court ruled that you do not have to belong to a non-vaxing religion or have clergy sign your religious exemption .

It does n't mention anything about the school system having the right to reject the exemption .

I was lucky enough to have my dds attend a private preschool where the owner was very respectful of everyones differing opinions and did n't give us any problem as long as she had the exemption to show the state .

So , in a few years I will have to endure the whole exemption thing from my kid 's school , but I 'll deal with that when it comes .

I know New York has been difficult for non-vaccinating parents in the past , but have they changed their laws to put school authorities in the position to determine whose religion qualifies for the exemption and whose does not ?

For example , a private preschool or school for that matter who does not accept money from the state , does not have to accept the state 's exemption for vaxes .

They do not have a religious or philosophical exemption for school entry .

Our dd is 2 , so we have some time on the school issue and we know we will homeschool…..but the issue of exemption for children is very important for us .

I picked up a vaccine exemption form from one of the local public schools when I was touring (got a nasty look from them going through the forms trying to find it) , and I plan on filling it out and having it on hand with our other homeschooling paperwork .

it might have a personal exemption but that is only for school .

You do not have to tell the school so it should n't interfere with your exemption .

You can show proof of immunity and not have to provide an exemption for school .

The only thing i have ever done regarding paperwork is to sigh a little exemption form saying that I am religiously or philosophically opposed for the daycare to have on file .

The only thing i have ever done regarding paperwork is to sigh a little exemption form saying that I am religiously or philosophically opposed for the daycare to have on file .

We have attended two super traditional , mainstream Baptist preschools and have n't had a bit of a problem with an exemption .

We have attended two super traditional , mainstream Baptist preschools and have n't had a bit of a problem with an exemption .

I honestly have not looked into the exemption issue very much , we are not using public school and the ped does n't really care if we get shots or not .

Were it not for the diocesan rule , parents could choose not to have the vaxes with aborted fetal tissue (or no vaxes , or whatever) , file an exemption , and submit the exemption to the schooln (although the school could refuse to accept it pursuant to the Texas AG 's opinion stating that private schools do not have to accept exemptions ; although I strongly feel that if anyone ever mounted a serious legal challenge on this point , the courts would come to a different conclusion on this issue) .

Were it not for the diocesan rule , parents could choose not to have the vaxes with aborted fetal tissue (or no vaxes , or whatever) , file an exemption , and submit the exemption to the schooln (although the school could refuse to accept it pursuant to the Texas AG 's opinion stating that private schools do not have to accept exemptions ; although I strongly feel that if anyone ever mounted a serious legal challenge on this point , the courts would come to a different conclusion on this issue) .

sign

I know some people think that we should not sign the waiver , but to me it is no different than when I file the exemption form for school .

(long story) I signed a religious waiver exemption and let the daycare provider know that I would like to be informed when another child at the center (she will be in the Infant Toddler room until Oct.) is vaccinated and returns to center same day which apparently happens quite a bit .

For school , Maryland has a religious exemption here : You would just sign it and turn it in .

When we applied for Medi-Cal and Welfare I was required to bring in their immunization cards as well and I printed out the school exemption form and signed it as requred by law (i also had the law quoted on the bottom of my letter of exemption) and brought that in instead of their card .

Please keep in mind that namecalling is not acceptable . live in Kansas and am looking for an MD who would sign the medical exemption needed for school entrance .

So….when she goes to sign up her kids for school , she only needs to bring the religious exemption (which also happens to be blue) and the yellow physical form from the doctor ‘s office . all , Well a mum on one of the parenting sites I ‘m a member of said this when asked ‘why ppl chose to or not to vaccinate ‘ She knew it was going to be a hard decision to make as when she was in hospital there was a little boy who had had a bad reaction to the chicken pox vaccine and had to be hospitalised and almost died .

I ‘ll tell you that in my case I was able to find a pediatrician who would not insist on vaxing , and who agreed to sign a form for me that said I had a medical exemption (based on my true story about ds ‘s bad reaction to his two-month round of vaccinations) for preschool .

So , am I lying by signing a religious exemption for school ?

Nissa aka ” The ” Children ‘s Librarian Here in CA it ‘s very easy , all you have to do is sign the back of the school ‘s vaccination form , stating you have a philosophical (or whichever) exemption .

In Florida , all you have to do is go to the health department , request a religious exemption and they give you a blue form to sign (which you turn in to the school district in lieu of the blue form which the pediatrician gives you with the list of vaccines that child has received) .

In CO , it was as easy as flipping over their shot record and signing it (never had a weird look from the office ladies) In NM , we have to get the exemption notarized (easy) , and then approved by the health department (easy , except that it ‘s an hr drive away – I was worried about them getting lost in the mail) and then turn them into the school nurse .

For the record I am actually am an atheist so I obviously have no religious beliefs against vaxs , but is ever questioned , ” my religion is private to me and I wish not to discuss it ” is my answer. Â My dd will be starting at a private Catholic school in the fall. Â I Â called and spoke with the nurse about vaccination exemption before we sent in the first check. Â She said that they would accept our Â exemption. Â They do n’t have to accept it , I do n’t think , because they do n’t accept any funding from the government. Â They take no money , no food , etc. Â The whole school is tuition Â funded. Â So they Â are allowed to exclude people. Â We will file a religious exemption because Â that is

what our state allows. Â I have heard of Â Catholic schools refusing exemption letters before. Â Â Here is what makes me nervous. Â The school we are looking at is in the midst of new leadership. Â they have an interm principle that is leaving and two nuns are coming. Â It makes me nervous to commit to a year ‘s tuition w/ this change. Â What if the nuns come in and decide no exemptions ? Â The school nurse will be the same. Â Registration is this week , and I have to bring my exemption w/ birth cert in lieu of vaccine records. Â If they accept us , and we sign a contract with the exemption on file , can they change their minds mid year ? Â I would HATE to lose that money. Â I suppose it ‘s possible , but very unlikely .

Some states make it easy for parents to claim an exemption by simply signing a prewritten statement on the school immunization form .

! completely non vaxed kids are 2 and 4. Â As we start to look at public Kindergarten for my son , I ‘m looking over the forms. Â Â I know I ‘ll probably just state my religious beliefs and get an exemption based on that , but I ca n’t help thinking it ‘d be SO much easier to just fill out the form as it ‘s ” supposed ” to look and sign a Â non existant Â doctor ‘s name. Â I live in a metro area and I ca n’t imagine they call all the doctors on all the kids that come into the school. Â I imagine they just check to see that the form filled out and they file it. Â Â NO ONE knows we do n’t vaccinate. Â We have n’t told friends or neighbors or any one. Â Â And if we were caught , so what ? Â To sign a name for someone that does n’t exist is n’t forgery. Â And , there ‘s no law saying you have to tell the truth on school forms. Â Overall , the only places you legally ca n’t lie is under oath in a court of law. Â Â Any lawyers out there ? Â Whadya think ? Â Personally Â I would not do that – unless totally backed into a corner and felt I had NO other options whatsoever .

Cate I have n’t filed with the school yet but I ‘ve found the form and letter to be pretty simple here in KY , though it ‘s really strange they wanted our doctor or the health dept to sign the exemption form – I mean what do they have to do with personal religious beliefs beyond trying to argue against them ?

Are there certain schools that can deny us or should we be able to sign an exemption and be accepted to any school we choose ?

I do have my religious exemption form (provided by the state…meant for children entering school) signed and notarized , so it ‘s sitting here waiting in case something come up .

I went to the school board office with a notarized letter to the effect of the above code and was given to sign a new Certificate of Religious Exemption with the physical exam part added to the bottom .

We were given the blue forms that allows you to sign the exemption (California) , but I guess private daycares do n’t have to abide by that exemption .

2) As far as the schools , you sign an exemption form and yes , if there is an outbreak of a vaccinatable (is that a word ?

You can be Wiccan , Lutheran , Catholic , Jewish , church of the flip flops , and still claim a religious exemption do to your personal religious beliefs against the practice of vaccination .

He actually HAS been vax ‘d (before I knew better) so I have one , BUT I am not thinking I should turn it into them since I will be claiming religious exemption for all of my children .

Like : ” well if you vaxed her before , why are you suddenly claiming religious exemption ?

I had no problems rejecting all vaccines once claiming the exemption .

I think it would be tricky to claim an exemption then get additional vaxes .

If both parents want to claim religious beliefs against vaxing , then the active duty spouse would have to file an exemption for him/herself .

But you do n’t need to give the vax history anyway to claim a medical exemption .

But I have heard that in some states (Florida comes to mind) people have had problems with selective vaxing , because once you claim a religious exemption there , if you give any more vaxes the exemption is no longer valid .

I am not sure on that one but most people who inject some vaccines into their child still claim a complete exemption and just do n’t mention the vaccines they gave .

I claim personal exemption for my child , (child ‘s name) , regarding all vaccines (or list certain ones you do n’t do) .

We are not claiming a religious exemption (our religion does not forbid vax , though it does have a few things to say about lying !

Has anyone successfully claimed a religious exemption after partially vaxing ?

They can not force you , as an adult , to get vaccinated , even if you do n’t claim an exemption .

Â Also , as far as I know , you can claim a religious exemption for just some vaccines , it does n’t have to be all or nothing. Â For instance , some people religiously oppose only a few of the vaccines based on how they were created (in aborted fetal lung cells for example) .

And dh would really like for elliot to get a few vaccines , like dtap and hepA , prior to going overseas , but if we do a few we ca n’t claim religious exemption , right ?

As a dependent (they already have my vax record on file) can I still claim a religious exemption ?

LongIsland is the expert on exemptions and there is some good info in the stickies above but if I am understanding you correctly , you do n’t specify certain vaxes when you claim an exemption .

Now I think where questions arise is if you vax for a while then stop (but you can always claim that your religious beliefs evolved , if that is what happened) or if you want to have some vaxes but not others… say for the sake of argument you were ok with IPV , HEP B , DTaP but nothing else… How then do you claim to have a religious exemption to some and not the others ?

Now I think where questions arise is if you vax for a while then stop (but you can always claim that your religious beliefs evolved , if that is what happened) or if you want to have some vaxes but not others… say for the sake of argument you were ok with IPV , HEP B , DTaP but nothing else… How then do you claim to have a religious exemption to some and not the others ?

They are required for school in our state , but if I got with the Philosophical exemption I plan on just claiming he never had any shots .

exempt

I do n't think they 're allowed to ask anything to get the waiver , but the exemption statute specifically states that it is for exempting from all vaccines , not specific shots .

Even if the Church told all Catholic parents not to let their child get the MMR for instance , most parents would have to still be required to submit a religous exemption which would exempt all vaccines .

If a parent wants to exempt their child only from the MMR , Hep A and varicella vaccines because of the aborted fetal tissue , the religious exemption would be invalid in almost every state .

I was n't clear on your posts as to whether you plan on exempting your child completely , but wanted to make you aware just case you are n't using an exemption that in Florida four (4) doses of a pertussis-containing vaccine are required unless the 4th dose is given on or after the fourth birthday .

Exemption from vaccination A student shall not be required to receive a vaccination pursuant to section 2 or 3 of this act [C. 18A : 61D-9 and 18A : 40-21.1] based upon one of the following : a. a written statement submitted to the secondary school or institution of higher education , as applicable , by a licensed physician indicating that the vaccine is medically contraindicated for a specific period of time and the reasons for the medical contraindication , based upon valid medical reasons as determined by regulation of the Commissioner of Health and Senior Services , which shall exempt the student from the vaccination for the stated period of time ; or b. a written statement submitted to the secondary school or institution of higher education , as applicable , by the student , or the student 's parent or guardian if the student is a minor , explaining how the administration of the vaccine conflicts with the bona fide religious tenets or practices of the student , or the parent or guardian , as appropriate ; except that a general philosophical or moral objection to the vaccination shall not be sufficient for an exemption on religious grounds .

Illinois Vaccine Awareness Coalition (IVAC) has this to say ; " Day-Care Exemptions The Day-care religious vaccine exemption contains the language that children can exempt if the " parents object thereto on the grounds that they conflict with the tenets and practices of a recognized church or religious organization , of which the parent is an adherent or member " .

One or two vaccines – you can get away with it if it 's worded correctly , but any attempt to use a medical exemption as a " blanket " to exempt most/all of the vaccines required – the parent is taking a huge risk .

I recall Beth Topp (Robert her son was injured by the hepB vaccine age 11.His photo and story is at

the Whale site) became very active in helping other parents learn about exemptions.When a bunch of parents were facing expulsion of their children for not doing the hepB (this was in CA somewhere) she marched into the office and showed them where they had to sign to exempt for that vaccine.I assume no questions were asked about why that one vaccine was done when all the others were.Hopefully someone on board who has actually done this can tell what they faced.My kids have not had any vaccines,so I ca n't say but from what I have read CA is easy going and the whole point of a philos exemption is to allow a parent to pick and choose .

Now if you claim a religious exemption you technically should not vaccinate anymore unless you exempted from certain vaccines for ingredient reasons only (human fetal cells,pork cells,and so on) .

I wasnt sure if I should get religious exemption for myself , so that in the future I can be exempt from any vaxes school or employers may try to force on me , or just submit the form and possibly deal with crap in the future .

I would exempt your family from any vaccination registry in case you decide to do some vaccines after you get your exemption .

Should you pick the medical exemption , it would only exempt your child for the ONE vaccine that was previously a problem to his health and it needs to be reevaluated every single year .

A medical exemption is out of the question – you 'd have to first find a doctor willing to exempt your child from all shots – not going to happen – and then have the medical exemption renewed every year .

Ok first of all , I 'm quite sure that you do not need to vaccinate to enter school , you can claim an exemption , and say it is against your beliefs " In every state of the union except two (Mississippi and West Virginia) , parents have the legal right to exempt their children from all vaccinations .

Â The exemption is easier for the schools than it has been in the past ; simply check the box and sign. Â Even though we still vaccinate , the schools seem less confused than if we do n't exempt , then get a call from the school nurse , then explain , then have to update records for each vax received .

There is no philisophical exemption in CT , and religious exemption only exempts from the IDEA of vaccinating , not specific vaccinations.. .

blek : state (NM) has a philisophical exemption , but there is only one exemption form that I can find and it reads : …I hereby certify that the administration of vaccine and other immunizing agents to my child as named above is contrary to my religious beliefs , held either individually or jointly by others , and therefore request that my child be exempted from the school immunization requirements… That to me reads as a religious exemption .

Most states only accept serological evidence to immunity from one or more of the following : measles mumps rubella chicken pox (varicella) hepatitis b Do you live in a state with an exemption that allows you to pick and chose which vaccines you can exempt ?

School exemptions exempt you from state law require vaccination in order to attend ; so when the exemption ceases to apply , they can not longer attend without vaccinations .

Though true , that has nothing to do with the exemption , which specifically exempts one from the attendance requirement to be vaccinated .

submit

I live in NJ and this is what the law states : A written statement should be submitted by the student , or the student ‘s parent or guardian if the student is a minor , explaining how the administration of the vaccine conflicts with the bona fide religious tenets or practices of the student , or the parent or guardian , as appropriate ; except that a general philosophical or moral objection to the vaccination shall not be sufficient for an exemption on religious grounds .

Ronna If they do n’t provide the school with a vax record , they must submit the Virginia religious exemption form – no way around it .

However , if the parent submits an exemption , then the vax record is not needed by the school .

If it ‘s a scenerio where they are submitting the exemption , but the school knows the child was previously vaccinated and wants the record , then they can submit both the vax record and exemption .

If it ‘s a scenerio where they are submitting the exemption , but the school knows the child was previously vaccinated and wants the record , then they can submit both the vax record and exemption .

IIRC , the exemption is part of the vax form that everyone else submits .

Even if the Church told all Catholic parents not to let their child get the MMR for instance , most parents would have to still be required to submit a religous exemption which would exempt all vaccines .

Were it not for the diocesan rule , parents could choose not to have the vaxes with aborted fetal tissue (or no vaxes , or whatever) , file an exemption , and submit the exemption to the schooln (although the school could refuse to accept it pursuant to the Texas AG ‘s opinion stating that private schools do not have to accept exemptions ; although I strongly feel that if anyone ever mounted a serious legal challenge on this point , the courts would come to a different conclusion on this issue) .

I have written and submitted our religious exemption form for all of his vaccinations to the school and am awaiting their decision .

My kids are partially vaxed and we just submitted a religious exemption to the school she will be attending this fall .

I would submit your exemption without prior vax history when you register for preschool and again for kindergarten .

You are not obligated to disclose prior vax history if you submit an exemption in lieu of a vaccination record .

However , if the school is not aware of a child ‘s vax status , then I would suggest not bringing it up and just submit the exemption w/o any immunization records .

We also had to give parents the exemption forms if they dind’t want to submit VAx records .

We will be in NY , and have filed the ” religious ” exemption , and submitted the school ‘s forms filled in by our doctor , but I imagine there are plenty of families at the school who selectively vaccinate , so I will have to see what they say .

You would keep your child ‘s shot records at home and NOT submit that to the school…only your exemption from all shots .

For off base schools you do not have to vaccinate just submit a phlosophical or religious exemption .

When you submit an exemption you can just state that you are religiously opposed to the practice of vaccination , and not submit any vax records .

When you submit an exemption you can just state that you are religiously opposed to the practice of vaccination , and not submit any vax records .

I ‘m simply curious b/c I submitted a written exemption to our preschool (that accepts unvaxed children and is funded) but they kicked it back giving me one day to fill out and have a form notorized .

requir

POLIOMYELITIS AND OTHER DISEASES , NY CLS Pub Health Â § 2164 (2002) , to receive Religious Exemption from Vaccination , ALL injections , prophylaxis , ; testing due to our genuine and sincere religious beliefs which are contrary to the practices herein required .

In this case they really are legally required to either go get the shots or else file the exemption .

/hijack off , sorry went to my local WIC office today (colorado) to set up an intake appt and when she got to bringing in dd ‘s immunizations record I said ” We do n’t vax will I need to bring in a philosophical exemption ” and she said ” no thats fine just tell your counselor and she wo n’t require that record ” YAY…. now I ‘m hearing horrible things from my friend about the ped I was going to take dd to so we might have to shop around for a GP… . ‘m leaving in a few hours to visit DH ‘s dad for Thanksgiving and I ‘m very concerned about the possibility of my unvaxed child catching hep B from him .

We are planning on handing in a religous exemption for the required vax sheet .

: Here is the link to the TX exemption information , although I ‘m not sure maybe they can require you to get certain vaccines for medical programs .

Even if the Church told all Catholic parents not to let their child get the MMR for instance , most parents would have to still be required to submit a religous exemption which would exempt all vaccines .

The duty element in Negligence is ” to act as a reasonably prudent person in the same or similar circumstances ” towards foreseeable plaintiffs. Â If vaxing is required by law , and a person does n’t have an exemption there could be an argument for ” negligence per se ” to establish the duty element , but then it would depend on the statutory language and how it had been interpreted .

I was n’t clear on your posts as to whether you plan on exempting your child completely , but wanted to make you aware just case you are n’t using an exemption that in Florida four (4) doses of a pertussis-containing vaccine are required unless the 4th dose is given on or after the fourth birthday .

As far as I know there is no regular waiver or exemption for vaxes for immigration because they are not required .

Oregon does not have a Philosophical exemption – only medical and religious That said , I can not find anywhere that the Flu vaccine is required for public schools/daycares .

i ‘ll be homeschooling , but i ‘m doing it under an umbrella school (same one i graduated from) and since they ‘re registered as a ” private school ” they require a health exam and up to date vax (or religious exemption , which i ‘ve had for her for a couple years .

The law does require vaxes for school entry ; some daycare and so forth – and that ‘s where an exemption comes in .

is campaigning the vaccine ? of my greatest concerns with this concept is that the government will start requiring vaccines for everyone , no exemptions allowed for any reason (well , except for the nearly impossible-to-obtain ” medical exemption ”) .

This may or may not require membership in an established religious organization , as individual state laws vary ; and 3) Philosophical or Personal Exemption : An increasing number of states allow one of these exemptions , in recognition of the controversy and/or violation of freedom that mandated vaccination laws impose .

My understanding is that if you selectively vax , you can still use a religious exemption (and you will need an exemption if you choose not to get any of the vaxes that are required for school entry) .

Vaccines are not required for school here , and it ‘s not necessary to fill in any forms or get an ” exemption ” , as vaccines are not compulsory in any way .

For example , if you plan to give your daughter the rubella vax when she is nine , if she tests negative for rubella , but the law requires that all children be vaxed for rubella at age 12 months and then again at 5 years , unless you go underground or get a medical exemption , you are out of luck .

There are so many neurotoxins in vaccines , it ‘s insane to think they are safe . the state (GA) does not require or provide a form for exemption can the county or other schools require a notorized

exemption on their own form or are they obligated to accept a written one ?

There are so many neurotoxins in vaccines , it ‘s insane to think they are safe . the state (GA) does not require or provide a form for exemption can the county or other schools require a notorized exemption on their own form or are they obligated to accept a written one ?

to consider a religious or philosophical exemption invalid and require a complete vax schedule (this really angers me , actually–the idea of informed consent should n't be all or nothing) .

vax

I ‘m leaning towards selectively vaxing , but am worried it ‘s going to be next to impossible to do in PA since the exemption laws are only for religious or medial reasons , which seem to me to be a blanket no vax situation .

Some people are pretending to be Christian Scientists to get the religious exemption ; others are Christian Scientists and get vaxed .

Now , I have an actual conscientious exemption in my possession , so I would just whip that out , but then , I had nothing but my right as a parent whose children were not in any group setting to not vaccinate them (and the way the law is written here , and in most states I have seen , it specifies children in such settings must be vaxed , NOT children in general !

Like : ” well if you vaxed her before , why are you suddenly claiming religious exemption ?

As for religious exemptions if you were denied from participating based on not vaxing and you had a religious exemption , they open themselves up for a civil lawsuit based on religious dicrimination .

/hijack off , sorry went to my local WIC office today (colorado) to set up an intake appt and when she got to bringing in dd ‘s immunizations record I said ” We do n't vax will I need to bring in a philosophical exemption ” and she said ” no thats fine just tell your counselor and she wo n't require that record ” YAY…. now I ‘m hearing horrible things from my friend about the ped I was going to take dd to so we might have to shop around for a GP… . ‘m leaving in a few hours to visit DH ‘s dad for Thanksgiving and I ‘m very concerned about the possibility of my unvaxed child catching hep B from him .

3 fewer vaxes she COULD receive if i vax . was just wondering ” when ” I should file my non-vaxing exemption form with the state ?

I did n't know if I should keep my choice of not to vax out of the system for as long as I can , or if I need to file my exemption now .

Also , he is 7 months old , exclusivly breastfed and has never been vaxed against anything and has never been sick – does any of that matter ? everyone , I need a religious exemption form for California for my son tomorrow .

There has to be some way around that (otherwise , ” they ” are forcing selective vax parents to not vax at all , or do all vaxes) I ‘ve read it in several places , basically once you have filed the exemption

form , that ‘s it .

The only way to avoid this is by getting a religious exemption which would put you in a position where you could not vax at all .

Could you get an exemption for a child who is already partially vaxed ?

My now 10 yr old is fully vaxed to age 4 , no Kindergarten boosters , just entered 5th grade , was enrolled into Kindergarten with a religious exemption .

Me : (long winded defensive rapid reasons why I do n’t want to vaccinate my dd , medical and personal reasons…. * * shakes in boots Nurse : Relax honey , I will send out an exemption for you ~ Then she went on to have a 20 minute conversation with me about how she thinks we should n’t vaccinate either , and that most parents who feel they have to vax are not fully informed , if they were , they would n’t put there children through it so much and at such an early age.. she then told me that only 1 of her 5 children got vaccinated , and that was only because she thought she HAD to vaccinate the first one .

I just want to clarify for morgan ‘s mom that , because she lives in Texas , she can ” pick and choose ” using the personal/religious exemption . is all so much for me to handel,every little sickness : out there every vax.I try to feel good about not vaxing and I just feel overwelhemed.I truly think that OCD has taken over my life since I got into not vaxing,I have become a weirdo,and all the info out there is to much to take.I belive that not vaxing is healthy yes you dont get all that garbace into your system.I also belive that the vax companys are out for $ $ $ $ but I also belive they want to protect us to ….And my Family thinks I am crazy for being able to prevent someting and not doing it..I really feel pulled in both derections and I dont want my lo to be one with a vax reaction,and I dont want my lo be one with a vpd..I feel I have no one to talk to this about,my mom acts bipoler,my DH is a BLEEP that just tells me do what you want…Vaxes I dont think are needed Depthira,Tetnus,Polio,Hep B,Hep A,MMR,Ones I would feel safer with but scared of the reactions would be Pertussis,Hib,and I dont know about Prevnar the ones that are probbly the most reactive,I truly feel like I am turnning into nut case and I really dont think I could get a shot for my lo,beacuse of the what if reactions….I just want to run,and run ,and run until I pass out and then go to the bar and get drunk and forget about all this stress of worring…I had to vent Momma , I ‘m sending you a big ol ‘ psychic hug .

I just want to clarify for morgan ‘s mom that , because she lives in Texas , she can ” pick and choose ” using the personal/religious exemption . is all so much for me to handel,every little sickness : out there every vax.I try to feel good about not vaxing and I just feel overwelhemed.I truly think that OCD has taken over my life since I got into not vaxing,I have become a weirdo,and all the info out there is to much to take.I belive that not vaxing is healthy yes you dont get all that garbace into your system.I also belive that the vax companys are out for $ $ $ $ but I also belive they want to protect us to ….And my Family thinks I am crazy for being able to prevent someting and not doing it..I really feel pulled in both derections and I dont want my lo to be one with a vax reaction,and I dont want my lo be one with a vpd..I feel I have no one to talk to this about,my mom acts bipoler,my DH is a BLEEP that just tells me do what you want…Vaxes I dont think are needed Depthira,Tetnus,Polio,Hep B,Hep A,MMR,Ones I would feel safer with but scared of the reactions would be Pertussis,Hib,and I dont know about Prevnar the ones that are probbly the most reactive,I truly feel like I am turnning into nut case and I really dont think I could get a shot for my lo,beacuse

of the what if reactions….I just want to run,and run ,and run until I pass out and then go to the bar and get drunk and forget about all this stress of worring…I had to vent Momma , I 'm sending you a big ol ' psychic hug .

The duty element in Negligence is " to act as a reasonably prudent person in the same or similar circumstances " towards foreseeable plaintiffs. Â If vaxing is required by law , and a person does n't have an exemption there could be an argument for " negligence per se " to establish the duty element , but then it would depend on the statutory language and how it had been interpreted .

I 'll vax him as I see fit , and I do n't want to be held down to an exemption at this point because there 's so many different schools of thought on this.. .

If both parents want to claim religious beliefs against vaxing , then the active duty spouse would have to file an exemption for him/herself .

I 've been getting letters saying either do an exemption , get her vaxed , or risk having her suspended from school .

file

The main point to understand here is that these parents are n't anti-vax , on top of their paperwork , and still getting picked on by The Man to get shots … this is all happening because the parents did n't file the paperwork , be it a vax record or an exemption .

The law says either get the shots or file an exemption , and they have done neither .

3 fewer vaxes she COULD receive if i vax . was just wondering " when " I should file my non-vaxing exemption form with the state ?

I did n't know if I should keep my choice of not to vax out of the system for as long as I can , or if I need to file my exemption now .

There has to be some way around that (otherwise , " they " are forcing selective vax parents to not vax at all , or do all vaxes) I 've read it in several places , basically once you have filed the exemption form , that 's it .

If both parents want to claim religious beliefs against vaxing , then the active duty spouse would have to file an exemption for him/herself .

Yes , the reason for getting it would just be so he 'd be 'complete ' , and I would n't have to file the exemption form for 1 shot .

Plus , there are more boosters and even totally new shots down the road (like when they get to 11-12) so you would be filing the exemption for those things too . stupid post/question ahead.. .

His 5 yr old brother did n't get past the 6 month round of shots , and an exemption was filed for him as well .

Their 2 yr old brother is vaccine free , and he too will have the exemption filed when the time comes . (due in 7 weeks) will be our first child that is completely not vaccinated .

We will be in NY , and have filed the ” religious ” exemption , and submitted the school ‘s forms filled in by our doctor , but I imagine there are plenty of families at the school who selectively vaccinate , so I will have to see what they say .

You can only file an exemption if you want to opt out of ALL vaccines .

Imagine explaining to your child , ” well you had this happen becasue you had a reaction to a vax , I knew it might be bad for you but figured i ‘d flip a coin and hope for the best.. sorry ” … trust me.. its hard , I in a way had to do that with my son , except i was able to blame his father since I had filed an exemption and he went over my head and got the vax anyway , then DS had a seizure .

From what I ‘ve ascertained , vaxing is NOT compulsory in Australia but they will ask for either the documentation that your child has been vaxed upon registering for school/medicare or for the conscientious exemption form that you can file with the gov’t .

And I mentioned that we would be filing an exemption with the state and would n’t be doing any vaxes and whether we not be seen because of this .

What I ‘m wondering is if I will have to file some kind of exemption for each vax we do n’t do for the school system if we decide not to homeschool when he ‘s old enough ?

The only way I can think of to maybe pull that off is to file the exemption with your school , then vax as you choose to vax with your doctor , and not give the school the vax record… but I doubt that would work because I ca n’t imagine you could tell the school that you are not giving them a vax record because there is nothing on it and have them take you at your word…If I were the school I would want to see the vax record , blank or not .

If they are positive , we will submit the results in lieu of the booster , and if they are negative we will either file an exemption or get her the single measles vaccine (still undecided) In VA it seems pretty straightforward to file an exemption .

They do n’t ask to see prior vax records when you file an exemption and in my understanding they do n’t ask if they have been vaxed before either .

We have to update his records , and they already have his current vax record- it seems a little late to file an exemption .

vaccin

I did read a sample exemption letter somewhere (I ca n’t post it cause I do n’t remember where it is right now) but it basically stated that I believe in God and God created the perfect immune system , and vaccinating is akin to a lack of faith in God .

I do know that Dr. Robert Mendelsohn testified on behalf of Orthodox Rabbis in Monsey , NY who had become ministers of the Universal Life Church to avoid vaxing their children and meet NY ‘s

then necessary religious exemption for not being vaccinated .

Even though I live in CA , which has a liberal application of its philosophical exemption , my siblings and I came home many times from both private and public schools with a note from the nurse warning that we would be excluded because we were not vaccinated .

Now , I have an actual conscientious exemption in my possession , so I would just whip that out , but then , I had nothing but my right as a parent whose children were not in any group setting to not vaccinate them (and the way the law is written here , and in most states I have seen , it specifies children in such settings must be vaxed , NOT children in general !

If it ‘s a scenerio where they are submitting the exemption , but the school knows the child was previously vaccinated and wants the record , then they can submit both the vax record and exemption .

(long story) I signed a religious waiver exemption and let the daycare provider know that I would like to be informed when another child at the center (she will be in the Infant Toddler room until Oct.) is vaccinated and returns to center same day which apparently happens quite a bit .

My dd has all but varicella and we still signed the exemption saying she ‘s unimmunized . 18 mo ds is fully vaccinated minus one we delayed because he was due for 3 shots at once .

Who is requesting an exemption from you ? have chosen not to vaccinate my children for our own personal reasons .

Me : (long winded defensive rapid reasons why I do n’t want to vaccinate my dd , medical and personal reasons…. * * shakes in boots Nurse : Relax honey , I will send out an exemption for you ~ Then she went on to have a 20 minute conversation with me about how she thinks we should n’t vaccinate either , and that most parents who feel they have to vax are not fully informed , if they were , they would n’t put there children through it so much and at such an early age.. she then told me that only 1 of her 5 children got vaccinated , and that was only because she thought she HAD to vaccinate the first one .

Me : (long winded defensive rapid reasons why I do n’t want to vaccinate my dd , medical and personal reasons…. * * shakes in boots Nurse : Relax honey , I will send out an exemption for you ~ Then she went on to have a 20 minute conversation with me about how she thinks we should n’t vaccinate either , and that most parents who feel they have to vax are not fully informed , if they were , they would n’t put there children through it so much and at such an early age.. she then told me that only 1 of her 5 children got vaccinated , and that was only because she thought she HAD to vaccinate the first one .

Me : (long winded defensive rapid reasons why I do n’t want to vaccinate my dd , medical and personal reasons…. * * shakes in boots Nurse : Relax honey , I will send out an exemption for you ~ Then she went on to have a 20 minute conversation with me about how she thinks we should n’t vaccinate either , and that most parents who feel they have to vax are not fully informed , if they were , they would n’t put there children through it so much and at such an early age.. she then told me that only 1 of her 5 children got vaccinated , and that was only because she thought she HAD to vaccinate the first one .

Me : (long winded defensive rapid reasons why I do n't want to vaccinate my dd , medical and personal reasons…. * * shakes in boots Nurse : Relax honey , I will send out an exemption for you ~ Then she went on to have a 20 minute conversation with me about how she thinks we should n't vaccinate either , and that most parents who feel they have to vax are not fully informed , if they were , they would n't put there children through it so much and at such an early age.. she then told me that only 1 of her 5 children got vaccinated , and that was only because she thought she HAD to vaccinate the first one .

was , can you use this exemption if your child has been partially vaccinated .

There is no requirement to vaccinate or provide an exemption letter in Alberta .

So I did a few searches and found a form for the NC religious exemption and I am going to take it to office tomorrow with a brief over view of our religious beliefs when it comes to vaccinating .

It 's on the North side of town apparently , no idea how many others she came in contact with : Call me selfish : , but this is why I do n't advertise my concerns with vaccines , the fact that I do n't vaccinate , or the fact that you can obtain an exemption .

Here is the Indiana weblink for religious exemption can I just file the letter for now and then give it to the school system (I do n't want to fight the health department who has n't seemed to notice that we are n't vaccinating anymore) I 'm not sure what your question is .

laughup Speaking of newborns , here 's a good example : It 's just like the flu vaccine – vaxing the kids to protect gramma – except with pertussis you vaccinate the older kids to protect the babies I bet both work equally well love and peace . know I have seen a few simple religious exemption letters on this board and now I ca n't seem to find one .

The deal is that you have to fill out an exemption form and just say you are n't vaccinating .

If you wanted to put the religious exemption with your ped office records , you could just tell you doctor that in the years since you had dd , you discovered it was against your religious beliefs to vaccinate , and wanted a copy of your exemption letter in her files .

herein

POLIOMYELITIS AND OTHER DISEASES , NY CLS Pub Health Â § 2164 (2002) , to receive Religious Exemption from Vaccination , ALL injections , prophylaxis , ; testing due to our genuine and sincere religious beliefs which are contrary to the practices herein required .

(see below) " Sample Letter " I found on Mothering : To whom it may concern ; We , and as the parents of , are exercising our rights under the US Constitution , Army Regulation 40-562 , BUMEDINST 6230.15A , AF JI 48-110 , CG COMDTINST M6230.4F , section 3-2 , paragraph 4 and Army Regulation 608-10 , section 4-6 , to receive Religious Exemption from Vaccination , due to our genuine and sincere religious beliefs which are contrary to the practices herein required .

" To whom it may concern ; (We / I) { First and Last name (s) } , as the { (parent (s) / guardian (s) } of (name of newborn child) are exercising (our/my) rights under the US Constitution , Army

Regulation 40-562 , BUMEDINST 6230.15A , AF JI 48-110 , CG COMDTINST M6230.4F , section 3.2 , para4 , and Army Regulation 608-10 , section 4-6 , para 2 , to receive Religious Exemption from Vaccination , due to our genuine and sincere religious beliefs which are contrary to the practices herein required .

So , we 'll see what happens now To whom it may concern ; (We / I) { First and Last name (s) } , as the { (parent (s) / guardian (s) } of (name of newborn child) are exercising (our/my) rights under the US Constitution , Army Regulation 40-562 , BUMEDINST 6230.15A , AF JI 48-110 , CG COMDTINST M6230.4F , section 3.2 , para4 , and Army Regulation 608-10 , section 4-6 , para 2 , to receive Religious Exemption from Vaccination , due to our genuine and sincere religious beliefs which are contrary to the practices herein required .

To whom it may concern ; (We / I) { First and Last name (s) } , as the { (parent (s) / guardian (s) } of (name of newborn child) are exercising (our/my) rights under the US Constitution , to receive Religious Exemption from Vaccination , ALL injections , prophylaxis , ; testing due to our genuine and sincere religious beliefs which are contrary to the practices herein required .

So we submitted the school vaccination form (which allows for all exemptions) with the religious exemption signed , along with a signed copy of this letter : To whom it may concern ; (We / I) { First and Last name (s) } , as the { (parent (s) / guardian (s) } of (name of newborn child) are exercising (our/my) rights under the US Constitution , Army Regulation 40-562 , BUMEDINST 6230.15A , AF JI 48-110 , CG COMDTINST M6230.4F , section 3.2 , para4 , and Army Regulation 608-10 , section 4-6 , para 2 , to receive Religious Exemption from Vaccination , due to our genuine and sincere religious beliefs which are contrary to the practices herein required .

NRS Â § 432A.230 (2007) , Â § 432A.230) to receive Religious Exemption from Vaccination , ALL injections , prophylaxis , ; testing due to our genuine and sincere religious beliefs which are contrary to the practices herein required .

Section 15C ; to receive Religious Exemption from Vaccination , ALL injections , prophylaxis , ; testing due to our genuine and sincere religious beliefs which are contrary to the practices herein required .

To whom it may concern ; I { First and Last name (s) am exercising my rights under the US Constitution , AR 40 â   562/BUMEDINST 6230.15A/AFJI 48 â   110/CG COMDTINST M6230.4F Immunizations and Chemoprophylaxis , and 29 CFR 1605 ; to receive Religious Exemption from Vaccination , due to my genuine and sincere religious beliefs which are contrary to the practices herein required .

To whom it may concern ; (We / I) { First and Last name (s) } , as the { (parent (s) / guardian (s) } of (name of newborn child) are exercising (our/my) rights under the US Constitution , Army Regulation 40-562 , BUMEDINST 6230.15A , AF JI 48-110 , CG COMDTINST M6230.4F , section 3.2 , para4 , and Army Regulation 608-10 , section 4-6 , para 2 , to receive Religious Exemption from Vaccination , due to our genuine and sincere religious beliefs which are contrary to the practices herein required .

For my reason , I copied something I found here : I am exercising my rights under the US Constitution to receive Religious Exemption from Vaccination due to our genuine and sincere

religious beliefs which are contrary to the practices herein required .

[quote] To whom it may concern ; (We / I) { First and Last name (s) } , as the { (parent (s) / guardian (s) } of (name of newborn child) are exercising (our/my) rights under the US Constitution , AFI34-248 1 OCTOBER 1999 , and The National Resource Center for Health and Safety in Childcare , to receive Religious Exemption from Vaccination , due to our genuine and sincere religious beliefs which are contrary to the practices herein required .

POLIOMYELITIS AND OTHER DISEASES , NY CLS Pub Health 2164 (2002) , to receive Religious Exemption from Vaccination , ALL injections , prophylaxis , ; testing due to our genuine and sincere religious beliefs which are contrary to the practices herein required .

I am the spouse of an active duty AF member. Â We have enrolled our son on an on base DODEA pre-school class. Â I used all of the information provided on this site. Â We live in Alabama which allows for religious exemption. Â The school nurse had asked for our son ‘s ‘blue card ‘ which is Alabama ‘s immunization certificate. Â Â I brought in the certificate , the DODEA exemption request (which you can not find on their site but can find here) and a letter which stated the following : Â To whom it may concern ; I , XXXX , as the parent of XXXXX , am exercising my rights under the US Constitution , Army Regulation 40-562 , BUMEDINST 6230.15A , AF JI 48-110 , CG COMDTINST M6230.4F , section 3.2 , para4 , and Army Regulation 608-10 , section 4-6 , para 2 , to receive Religious Exemption from Vaccination , due to my genuine and sincere religious beliefs which are contrary to the practices herein required .

Anyway here ‘s a copy of it ; any critiques are appreciated : I , , as the parent of and am exercising my rights under 028 PA Code 23.84 , to receive Religious Exemption from vaccinations due to our genuine and strong ethical convictions which are contrary to the practices herein required .

I , , as the parent of and am exercising my rights under 028 PA Code 23.84 , to receive a Religious Exemption from vaccinations due to our genuine and strong ethical convictions which are contrary to the practices herein required .

need

I just got a letter home from her school saying she needs her shots or an exemption filled out before the 27th or she can not come back .

-For a religious exemption you need to be ” against the practice of vaccination ” .

I asked her what I needed to turn in if my son had already received some vaccinations but I have since obtained a religious exemption .

However , if the parent submits an exemption , then the vax record is not needed by the school .

In order to get your exemption , you will need to ” prove ” in a written statement that your view on vaccinations are of a sincere religious nature and not philosophical .

/hijack off , sorry went to my local WIC office today (colorado) to set up an intake appt and when she got to bringing in dd ‘s immunizations record I said ” We do n’t vax will I need to bring in a

philosophical exemption ” and she said ” no thats fine just tell your counselor and she wo n’t require that record ” YAY…. now I ‘m hearing horrible things from my friend about the ped I was going to take dd to so we might have to shop around for a GP… . ‘m leaving in a few hours to visit DH ‘s dad for Thanksgiving and I ‘m very concerned about the possibility of my unvaxed child catching hep B from him .

I did n’t know if I should keep my choice of not to vax out of the system for as long as I can , or if I need to file my exemption now .

Also , he is 7 months old , exclusivly breastfed and has never been vaxed against anything and has never been sick – does any of that matter ? everyone , I need a religious exemption form for California for my son tomorrow .

I just want to clarify for morgan ‘s mom that , because she lives in Texas , she can ” pick and choose ” using the personal/religious exemption . is all so much for me to handel,every little sickness : out there every vax.I try to feel good about not vaxing and I just feel overwelhemed.I truly think that OCD has taken over my life since I got into not vaxing,I have become a weirdo,and all the info out there is to much to take.I belive that not vaxing is healthy yes you dont get all that garbace into your system.I also belive that the vax companys are out for $ $ $ $ but I also belive they want to protect us to ….And my Family thinks I am crazy for being able to prevent someting and not doing it..I really feel pulled in both derections and I dont want my lo to be one with a vax reaction,and I dont want my lo be one with a vpd..I feel I have no one to talk to this about,my mom acts bipoler,my DH is a BLEEP that just tells me do what you want…Vaxes I dont think are needed Depthira,Tetnus,Polio,Hep B,Hep A,MMR,Ones I would feel safer with but scared of the reactions would be Pertussis,Hib,and I dont know about Prevnar the ones that are probbly the most reactive,I truly feel like I am turnning into nut case and I really dont think I could get a shot for my lo,beacuse of the what if reactions….I just want to run,and run ,and run until I pass out and then go to the bar and get drunk and forget about all this stress of worring…I had to vent Momma , I ‘m sending you a big ol ‘ psychic hug .

But you do n’t need to give the vax history anyway to claim a medical exemption .

I think the only time you would need to obtain an exemption form would be to offer to an organization that is requesting a vax record (or exemption) .

Google ” your state ” vaccine exemption information and you ‘ll find everything you need to know .

Also , you do not need a vaccination exemption just to see a doctor , nor do you need to sign any refusal papers .

Also , you do not need a vaccination exemption just to see a doctor , nor do you need to sign any refusal papers .

Someone did that for me on the babycenter board and I ‘ve been forever grateful ! is strarting pre-school next week and I thought our pediatrician was sending us whatever would be needed to use for exemption , but all she sent was the completed form from the school with a note that ” the parents have chosen a delayed vaccine schedule .

Â And then , at some point , if you send your kids to school , someone will ask for their shot records , and you will need to fill out an exemption form .

– I think state requirements need to be loosened (instead of being increased) or exemptions need to be more available (instead of becoming less) – and there could be different exemptions for sel/non – really when x % of a school has exemption , it does not mean that x % is completely unvaccinated – that misleading figure does not help anyone , it causes more panic on provax and nonvax bears the brunt. Â Â I have not been arguing for total end of vaccine campaigns or ending all vaccine production .

– I think state requirements need to be loosened (instead of being increased) or exemptions need to be more available (instead of becoming less) – and there could be different exemptions for sel/non – really when x % of a school has exemption , it does not mean that x % is completely unvaccinated – that misleading figure does not help anyone , it causes more panic on provax and nonvax bears the brunt. Â Â I have not been arguing for total end of vaccine campaigns or ending all vaccine production .

Â Thanks ! I have decided to stop vaxing , do I need to get a religious exemption form ASAP ?

My son had gone through the same school system as my daughter…obviously with him being vaccinated an exemption was not necessary , but in my daughters case it needed to be provided .

sign

(long story) I signed a religious waiver exemption and let the daycare provider know that I would like to be informed when another child at the center (she will be in the Infant Toddler room until Oct.) is vaccinated and returns to center same day which apparently happens quite a bit .

My dd has all but varicella and we still signed the exemption saying she 's unimmunized . 18 mo ds is fully vaccinated minus one we delayed because he was due for 3 shots at once .

Also , you do not need a vaccination exemption just to see a doctor , nor do you need to sign any refusal papers .

When signing the vaccination waver form for the ped 's office I did not write " Religions exemption " Instead I wrote something about doing research on side effects .

If you do n't intend to give anymore shots , then you 're probably better off to sign the exemption to avoid future shots .

I have questions…. thanks mamdawg I 'm definitely not signing anything I had to learn the hard way.Our son is vaccine damaged with seizures.You do n't have to be on the defense,well researched,or particularly articulate.JUST DO N'T SIGN ANYTHING ! I would get an exemption form (religious or philosphical) to hand to whoever to keep on file.If I had done this 5 babies ago I 'd have saved ourselves a lot of suffering and grief .

So….when she goes to sign up her kids for school , she only needs to bring the religious exemption (which also happens to be blue) and the yellow physical form from the doctor 's office . all , Well a

mum on one of the parenting sites I ‘m a member of said this when asked ‘why ppl chose to or not to vaccinate ‘ She knew it was going to be a hard decision to make as when she was in hospital there was a little boy who had had a bad reaction to the chicken pox vaccine and had to be hospitalised and almost died .

I ‘ll tell you that in my case I was able to find a pediatrician who would not insist on vaxing , and who agreed to sign a form for me that said I had a medical exemption (based on my true story about ds ‘s bad reaction to his two-month round of vaccinations) for preschool .

I so wish I had takend my religious exemption out and said this is the only document I will sign in regards to vaccinating my children .

38) RELIGIOUS EXEMPTION FORM Religious exemption requests must contain the wording found in the Alaska Administrative Code [4 AAC06.55 (b) (3) or 4 AAC 62.450 (c) (2)] stating all vaccines must be received unless the child â has an affidavit signed by his [4 AAC 62.450 (c) (2) says â the child â s â] parent or guardian affirming that immunization conflicts with the tenets and practices of the church or religious denomination of which the applicant [4 AAC 62.450 (c) (2) says â the parent or guardian â] is a member .

Nissa aka ” The ” Children ‘s Librarian Here in CA it ‘s very easy , all you have to do is sign the back of the school ‘s vaccination form , stating you have a philosophical (or whichever) exemption .

I have my exemption in my children ‘s files and it would probably lessen the liklihood of your ped asking you to sign the Refusal to Vaccinate form .

The CDC here told me they would n’t accept the any kids w/out shots , even with a signed exemption .

The exemption is really easy to get , that cant not sign it , you just have to listen to them tell you the risks of not vaxing , then they sign it , and you send it off , this also gets you the vax allowance eve if you are not vaxing .

In Florida , all you have to do is go to the health department , request a religious exemption and they give you a blue form to sign (which you turn in to the school district in lieu of the blue form which the pediatrician gives you with the list of vaccines that child has received) .

I ‘m pretty sure my mom signed the exemption forms after that , because I do n’t recall ever receiving another vax .

I have also taken in kids in the past who were not vaxed , and they just had to get an exemption form signed .

In CO , it was as easy as flipping over their shot record and signing it (never had a weird look from the office ladies) In NM , we have to get the exemption notarized (easy) , and then approved by the health department (easy , except that it ‘s an hr drive away – I was worried about them getting lost in the mail) and then turn them into the school nurse .

For the record I am actually am an atheist so I obviously have no religious beliefs against vaxs , but

is ever questioned , ” my religion is private to me and I wish not to discuss it ” is my answer. Â My dd will be starting at a private Catholic school in the fall. Â I Â called and spoke with the nurse about vaccination exemption before we sent in the first check. Â She said that they would accept our Â exemption. Â They do n’t have to accept it , I do n’t think , because they do n’t accept any funding from the government. Â They take no money , no food , etc. Â The whole school is tuition Â funded. Â So they Â are allowed to exclude people. Â We will file a religious exemption because Â that is what our state allows. Â I have heard of Â Catholic schools refusing exemption letters before. Â Â Here is what makes me nervous. Â The school we are looking at is in the midst of new leadership. Â they have an interm principle that is leaving and two nuns are coming. Â It makes me nervous to commit to a year ‘s tuition w/ this change. Â What if the nuns come in and decide no exemptions ? Â The school nurse will be the same. Â Registration is this week , and I have to bring my exemption w/ birth cert in lieu of vaccine records. Â If they accept us , and we sign a contract with the exemption on file , can they change their minds mid year ? Â I would HATE to lose that money. Â I suppose it ‘s possible , but very unlikely .

Just sign the exemption form saying they havent had any vaccines .

have

He actually HAS been vax ‘d (before I knew better) so I have one , BUT I am not thinking I should turn it into them since I will be claiming religious exemption for all of my children .

You may want to search under ” Your state ‘s name ” vaccination exemption forms Depending where you live you may have to request them directly from the state and have them notarized and THEN turn them in .

You may want to search under ” Your state ‘s name ” vaccination exemption forms Depending where you live you may have to request them directly from the state and have them notarized and THEN turn them in .

As in lying your butt off , fabricating a vax record from out of town , or just white-lie lying (” yes , she ‘s up to date ”) Lest anyone think this in any way relates to me , you should know that we have ‘religious exemption ‘ papers on file for each of our boys with their family doctor .

I asked her what I needed to turn in if my son had already received some vaccinations but I have since obtained a religious exemption .

For example , a private preschool or school for that matter who does not accept money from the state , does not have to accept the state ‘s exemption for vaxes .

Now we have a medical exemption against that shot since I had documented proof she had cp already . , I have a few questions to ask .

Now we have a medical exemption against that shot since I had documented proof she had cp already . , I have a few questions to ask .

I had to have proof of vaccinations for the girl scouts (which we no longer do) and went ahead and got the religious exemption .

Now , I have an actual conscientious exemption in my possession , so I would just whip that out , but then , I had nothing but my right as a parent whose children were not in any group setting to not vaccinate them (and the way the law is written here , and in most states I have seen , it specifies children in such settings must be vaxed , NOT children in general !

Now , I have an actual conscientious exemption in my possession , so I would just whip that out , but then , I had nothing but my right as a parent whose children were not in any group setting to not vaccinate them (and the way the law is written here , and in most states I have seen , it specifies children in such settings must be vaxed , NOT children in general !

We currently have a vax exemption on file for my daughter (we plan on eventually selective/delay/not at all depending on the vaccine .

The law says either get the shots or file an exemption , and they have done neither .

I picked up a vaccine exemption form from one of the local public schools when I was touring (got a nasty look from them going through the forms trying to find it) , and I plan on filling it out and having it on hand with our other homeschooling paperwork .

DD has been VAX sort of on schedule (she has a medical exemption for some due to reaction at 2 months) which is when i began researching and so we have avoided all but Dt since then .

/hijack off , sorry went to my local WIC office today (colorado) to set up an intake appt and when she got to bringing in dd ‘s immunizations record I said ” We do n’t vax will I need to bring in a philosophical exemption ” and she said ” no thats fine just tell your counselor and she wo n’t require that record ” YAY…. now I ‘m hearing horrible things from my friend about the ped I was going to take dd to so we might have to shop around for a GP… . ‘m leaving in a few hours to visit DH ‘s dad for Thanksgiving and I ‘m very concerned about the possibility of my unvaxed child catching hep B from him .

Who is requesting an exemption from you ? have chosen not to vaccinate my children for our own personal reasons .

: am moving the Illinois at the beginning of next year , and have looked over the vaccine exemption information .

There has to be some way around that (otherwise , ” they ” are forcing selective vax parents to not vax at all , or do all vaxes) I ‘ve read it in several places , basically once you have filed the exemption form , that ‘s it .

I just sent in religious exemption and did n’t tell them what shots she did have .

get

I could probably get a medical exemption for her because she is allergic to eggs and she GOT the measles from the first vaccine at 12 months , but I decided to fill out an exemption instead .

I do n’t know where they get the idea that there are only 3 types of religions they can accept for an

exemption to vaccines .

I ‘m going to look into getting an exemption form right away (newborn wo n’t be getting the Hep B vaccine) , so I guess I should be prepared .

I ‘m going to look into getting an exemption form right away (newborn wo n’t be getting the Hep B vaccine) , so I guess I should be prepared .

Google yourstate vaccine exemption law to get more info (or search for your state ‘s name in this forum , every state has come up at least once !) .

I do n’t think they ‘re allowed to ask anything to get the waiver , but the exemption statute specifically states that it is for exempting from all vaccines , not specific shots .

– were you able to get an exemption or did you HAVE to get the Hep B vax ? ?

– were you able to get an exemption or did you HAVE to get the Hep B vax ? ?

Some people are pretending to be Christian Scientists to get the religious exemption ; others are Christian Scientists and get vaxed .

Some people are pretending to be Christian Scientists to get the religious exemption ; others are Christian Scientists and get vaxed .

The main point to understand here is that these parents are n’t anti-vax , on top of their paperwork , and still getting picked on by The Man to get shots … this is all happening because the parents did n’t file the paperwork , be it a vax record or an exemption .

The main point to understand here is that these parents are n’t anti-vax , on top of their paperwork , and still getting picked on by The Man to get shots … this is all happening because the parents did n’t file the paperwork , be it a vax record or an exemption .

In order to get your exemption , you will need to ” prove ” in a written statement that your view on vaccinations are of a sincere religious nature and not philosophical .

Talking about the ” dangers ” of vaxes , or even their ingredients in your request for exemption will get you DENIED .

If you stay with her I would say that you will be getting any future vaccinations from the health department/local clinic because you do not want your dd to be afraid to see her when she is ill. California has a personal belief exemption .

Is there a way to privately get vaccines without the school knowing so that you can still utilize the religious exemption ?

It might not matter anyway…not sure what state you are in , but here you can pick and choose which vaxes you want and get an exemption for the rest (it ‘s literally a checklist sheet) .

} } Generally the easiest thing to do is to get an exemption and not mention that the child has had some shots .

The only way to avoid this is by getting a religious exemption which would put you in a position where you could not vax at all .

I do n't know what vaccines you 're thinking about or your childcare sitution , but some people give the 1 or 2 vaxes they 're going to do before getting the exemption .

fill

I just got a letter home from her school saying she needs her shots or an exemption filled out before the 27th or she can not come back .

I could probably get a medical exemption for her because she is allergic to eggs and she GOT the measles from the first vaccine at 12 months , but I decided to fill out an exemption instead .

I picked up a vaccine exemption form from one of the local public schools when I was touring (got a nasty look from them going through the forms trying to find it) , and I plan on filling it out and having it on hand with our other homeschooling paperwork .

I am in CO , not CA , but CO has the three exemptions like CA , and two of my children go to private schools , neither one has any problem with accepting our exemptions , we are given the same vax [exemption] form to fill in as the public schools .

The deal is that you have to fill out an exemption form and just say you are n't vaccinating .

Â And then , at some point , if you send your kids to school , someone will ask for their shot records , and you will need to fill out an exemption form .

We will be in NY , and have filed the " religious " exemption , and submitted the school 's forms filled in by our doctor , but I imagine there are plenty of families at the school who selectively vaccinate , so I will have to see what they say .

Vaccines are not required for school here , and it 's not necessary to fill in any forms or get an " exemption " , as vaccines are not compulsory in any way .

I 'm simply curious b/c I submitted a written exemption to our preschool (that accepts unvaxed children and is funded) but they kicked it back giving me one day to fill out and have a form notorized .

3 out of my 1st 6 children have reacted badly.My next to youngest had a seizure collapse within an hour of multiple vaxes.He 's since gone on to develop an " atypical " seizure disorder.Our youngrst dd has no vaxes whatsoever.I filled out a religious exemption which I 've had to turn into the health dept.They called me,harrased and harangued me,but there 's nothing they can do about it , period .

Submit a religious exemption ; there is one in the first link of my siggy . chiropractor offered to fill inand sign my ds ' shot records ds1 has one as we vaxed him for the first 9 months However , ds2

does n’t even have the record to dishonestly fill in –any ideas where I could get one ?

Submit a religious exemption ; there is one in the first link of my siggy . chiropractor offered to fill inand sign my ds ‘ shot records ds1 has one as we vaxed him for the first 9 months However , ds2 does n’t even have the record to dishonestly fill in –any ideas where I could get one ?

You just must do it ” privately ” , which means you need to go to a different doctor (if that doctor has to fill out what vaccines you ‘ve had or sign off on your exemption , as some states require) for the vaxes , not telling the school .

So , my DH dropped off the papers at the school today , the director looked over the papers to make sure everything was there , noticed that the vax record was not filled in all the way , and then saw the vax waiver (I got one off of dr. tenpenny ‘s site , since she is in ohio) , and said to my DH , ” Oh good , I ‘m glad you included your own exemption form (we used the philosophical one) , b/c I have to report it to the state and if you did n’t fill one of these out , I would have to have you fill one out .

So , my DH dropped off the papers at the school today , the director looked over the papers to make sure everything was there , noticed that the vax record was not filled in all the way , and then saw the vax waiver (I got one off of dr. tenpenny ‘s site , since she is in ohio) , and said to my DH , ” Oh good , I ‘m glad you included your own exemption form (we used the philosophical one) , b/c I have to report it to the state and if you did n’t fill one of these out , I would have to have you fill one out .

! completely non vaxed kids are 2 and 4. Â As we start to look at public Kindergarten for my son , I ‘m looking over the forms. Â Â I know I ‘ll probably just state my religious beliefs and get an exemption based on that , but I ca n’t help thinking it ‘d be SO much easier to just fill out the form as it ‘s ” supposed ” to look and sign a Â non existant Â doctor ‘s name. Â I live in a metro area and I ca n’t imagine they call all the doctors on all the kids that come into the school. Â I imagine they just check to see that the form filled out and they file it. Â Â NO ONE knows we do n’t vaccinate. Â We have n’t told friends or neighbors or any one. Â Â And if we were caught , so what ? Â To sign a name for someone that does n’t exist is n’t forgery. Â And , there ‘s no law saying you have to tell the truth on school forms. Â Overall , the only places you legally ca n’t lie is under oath in a court of law. Â Â Any lawyers out there ? Â Whadya think ? Â Personally Â I would not do that – unless totally backed into a corner and felt I had NO other options whatsoever .

! completely non vaxed kids are 2 and 4. Â As we start to look at public Kindergarten for my son , I ‘m looking over the forms. Â Â I know I ‘ll probably just state my religious beliefs and get an exemption based on that , but I ca n’t help thinking it ‘d be SO much easier to just fill out the form as it ‘s ” supposed ” to look and sign a Â non existant Â doctor ‘s name. Â I live in a metro area and I ca n’t imagine they call all the doctors on all the kids that come into the school. Â I imagine they just check to see that the form filled out and they file it. Â Â NO ONE knows we do n’t vaccinate. Â We have n’t told friends or neighbors or any one. Â Â And if we were caught , so what ? Â To sign a name for someone that does n’t exist is n’t forgery. Â And , there ‘s no law saying you have to tell the truth on school forms. Â Overall , the only places you legally ca n’t lie is under oath in a court of law. Â Â Any lawyers out there ? Â Whadya think ? Â Personally Â I would not do that – unless totally backed into a corner and felt I had NO other options whatsoever .

The vaccine record of my oldest grandson ‘showed ‘ that he was all up to date by the time he started school and in 2nd grade we decided NO MORE and filled out a religious exemption .

Well , I explained that it is against our religion to vax and asked if there was an exemption form I could fill out .

I found that for our daycare , I filed an exemption but then the physical report form the doctor has to fill out as part of daycare application has vaccination information .

us

I honestly have not looked into the exemption issue very much , we are not using public school and the ped does n’t really care if we get shots or not .

I just want to clarify for morgan ‘s mom that , because she lives in Texas , she can ” pick and choose ” using the personal/religious exemption . is all so much for me to handel,every little sickness : out there every vax.I try to feel good about not vaxing and I just feel overwelhemed.I truly think that OCD has taken over my life since I got into not vaxing,I have become a weirdo,and all the info out there is to much to take.I belive that not vaxing is healthy yes you dont get all that garbace into your system.I also belive that the vax companys are out for $ $ $ $ but I also belive they want to protect us to ….And my Family thinks I am crazy for being able to prevent someting and not doing it..I really feel pulled in both derections and I dont want my lo to be one with a vax reaction,and I dont want my lo be one with a vpd..I feel I have no one to talk to this about,my mom acts bipoler,my DH is a BLEEP that just tells me do what you want…Vaxes I dont think are needed Depthira,Tetnus,Polio,Hep B,Hep A,MMR,Ones I would feel safer with but scared of the reactions would be Pertussis,Hib,and I dont know about Prevnar the ones that are probbly the most reactive,I truly feel like I am turnning into nut case and I really dont think I could get a shot for my lo,beacuse of the what if reactions….I just want to run,and run ,and run until I pass out and then go to the bar and get drunk and forget about all this stress of worring…I had to vent Momma , I ‘m sending you a big ol ‘ psychic hug .

was , can you use this exemption if your child has been partially vaccinated .

I was n’t clear on your posts as to whether you plan on exempting your child completely , but wanted to make you aware just case you are n’t using an exemption that in Florida four (4) doses of a pertussis-containing vaccine are required unless the 4th dose is given on or after the fourth birthday .

Search for IL + exemption good luck , and sorry I could n’t be of more help just tell them aborted fetus parts are used in the manufacture of vaccines , and thus a business and government propagation of abortion .

) FWIW , I used the vax exemption for DS ‘s school .

Someone did that for me on the babycenter board and I ‘ve been forever grateful ! is strarting pre-school next week and I thought our pediatrician was sending us whatever would be needed to use for exemption , but all she sent was the completed form from the school with a note that ” the parents have chosen a delayed vaccine schedule .

-Angela Are you aware that you can avoid all vaccinations if you use an exemption ?

Even if you do vaccinate on a selective/delayed schedule privately , you can still use an exemption for school entry purposes .

The statutes I ‘m referring to are those ” requiring ” vaccination for school entry . ways the cdc influence our programming should same type of exemption be used for all siblings , regardless of previous vax status ?

I ‘m not in NJ , but where I am a very high percentage of parents do n’t vaccinate and many of the private daycares do n’t take any exemptions whether medical or religious. Â The ones that do are typically not the good ones. Â You can always call the preschools you are thinking of using later and ask about it. Â I do n’t think you should make this decision based on your child ‘s chances of getting into a good preschool though. Â I live in NJ and have been sending in religious exemption letters for my kids for years .

The only statement they made was that a person CAN use the exemption because of aborted fetus parts in the vaccine but they should consider the consequences of not vaccinating and how it may impact a whole community etc .

We all know that that is not necessarily true…I had the MMR 3 times and still show no immunity to the the R. I also came down with both of the Ms…..I guess I could never be a nurse…or I could just use an exemption Oh…yeah…CP was fine…Measles kinda sucked and the mumps was pretty uncomfortable…so much for vaccines Seriously ?

We have a medical exemption ~ however when our oldest daughter began Kindergarten this year we used the religious exemption since medical exemptions have had a bit of a history around here of being contested (or at the very least allowing exemption from only the single vaccination that poses a potential risk for your children) where we want to abstain from all vaccinations .

not the least bit religious here but had no qualms about using a religious exemption The Amish are a Christian sect . ‘m not getting the vaccine , but this came out in the Seattle Times today and if it ‘s happening in this state , it may happen in others .

My understanding is that if you selectively vax , you can still use a religious exemption (and you will need an exemption if you choose not to get any of the vaxes that are required for school entry) .

WRT the exemption issue , could n’t you use your exemption and then selectively vaccinate without telling the schools about it ?

double post I use a Religious Exemption for both vaccination and the school physical .

I just do n’t want it public information that the schools can access since I ‘d like to delay/selectively vaccinate and use either a religous or philosophical exemption for school .

I ‘m guessing the definition and what is covered by a religious exemption must vary state by state then as well. Â I know that in Kansas , Missouri , and Iowa there are families that have used a religious exemption to only opt out of some vaccinations .

oppos

Some states , like NY (contact Gary Krasner for help – On the NYS form they request that your letter : -explains why are you are requesting the exemption -describes the religious principles that guide your objection to vaxes -indicates whether you are opposed to all vaxes , and if not , why you are opposed to some vaxes but not others As long as your letter covered those 3 points then they should n't ask for any " supporting evidence " .

Some states , like NY (contact Gary Krasner for help – On the NYS form they request that your letter : -explains why are you are requesting the exemption -describes the religious principles that guide your objection to vaxes -indicates whether you are opposed to all vaxes , and if not , why you are opposed to some vaxes but not others As long as your letter covered those 3 points then they should n't ask for any " supporting evidence " .

This Pertussis Act makes it easier for a child to obtain and keep a medical exemption to pertussis vaccination as opposed to having their exemption challenged based on the CDC 's laughable contraindication list .

Â Also , as far as I know , you can claim a religious exemption for just some vaccines , it does n't have to be all or nothing. Â For instance , some people religiously oppose only a few of the vaccines based on how they were created (in aborted fetal lung cells for example) .

When you submit an exemption you can just state that you are religiously opposed to the practice of vaccination , and not submit any vax records .

My state 's (Arizona) religious exemption form allows you to check off which vax you are opposed to .

If so- if they ask if all of his vaccines are up to date u could just say " we are religiously opposed to vaccinations and I have an exemption form if you would like to see it " .

" In most states , you technically can not pick and choose using a religious exemption – some states do allow this however and have boxes to check off which vaccine you religiously oppose .

-Angela If you 're going to selectively vax (and I agree w/the above , keep researching before you make hat decision) – when it comes to exemption it 's best to just state that you are n't vaxing at all – esp in those " all or nothing " states where the law says that you must be opposed to the PRACTICE of vaccination , not individual vaccines . was going to apply for a promotion in Huntington .

If it were me , I 'd first say that our religious beliefs have changed but not get into anything about the aborted fetal tissue ; in most states with a religious exemption you have to be opposed to the practice of vaccination , not specific ingredients , and only a few vaccines even contain aborted fetal cell lines .

In ND where we live , we can claim a moral , religious , or philosophical exemption (my anti-vax Dad wondered " why is there no option for being SCIENTIFICALLY opposed to vaccines ?

I do n’t live in ND but I have a religious exemption even though our church is not opposed to vaccinations (they have no stance) .

i do have some reliougious reasons for not vaxing so i ‘m not opposed to using an exemption only .

Still , I would opt out regardless if your ped is enrolled in the registry b/c these days a child may be entered into the registry at birth (if new mother signs forms at the hospital opting in) , even if no vaccines are entered by a participating provider .

It was funny when the ped tried to say it was mild due to the vax , then realized the vaxed kid actually had the worst case between the two of them .

The HiB vaccine , which is supposedly one of the ” least problematic ” (according to the doctors) gave my 2 month old son a horrific reaction (high fever , sensitivity to light and sound , arching back , screeching hours on end , refusing to nurse) that even my ped agreed warranted a VAERS report .

My son had a reaction to his vaxes and even then , one ped .

Our ped does it at 15 months but we did n’t do it for either child .

I ‘ve only heard about 1 ped in the area who is okay w/ not-vaxing , and that was n’t the one he gave me (that one is n’t taking new patients) . aksed a question a few days ago about my 4 year old son .

Especially when people like my ped make comments like , ” you better let the staff of your church nursery or day care know that your daughter is n’t vax ‘d because she puts pregnant mothers at risk for disease , you know .

He does not get the pertussis vax due to a severe reaction to it , the MMR ; Chickenpox vaxes due to them being live vaxes–his ped does n’t like to give kids with autism or other severe developmental delays live vaxes , and the flu vax because I do n’t see the point of it for him .

Even if you know you will never vaccinate your children under any circumstances , that is n’t info your ped has to know the first visit .

My kid ‘s ped does recommend vaxing according to the CDC guidelines , however , he has many non-vaxing or selective vaxing patients and I have never been given a hard time about it .

We see the same ped so I ‘m sure when if he gets something and in just the right amount of time she ‘ll act like I ‘m crazy for suggesting it came from the baby that she just vaxed that swaps spit with my son every day .

Our ped recommended avoiding young children and groups of people , as well as public places the first two months with a newborn (vax status irrelevant) .

Well , having seen this ped for over 2 years now with my daughter and never questioning a thing about the vaccines she was administered (I am new to this selective/non-vaxing thing) , I have no clue as to how her reaction is going to be .

rach I did n't talk to my ped before having my son and refusing things like PKU , Hep B and Vit K. She did n't have rights to the hospital I gave birth .

I got my kids ' records released and mailed to me this week , and yep , there 's a ton of messages from the birth hospital to my ped about " mother refusing " this and that , and their comments about it .

We only see one ped , never another in the same practice , we only schedule appts for first thing in the morning to avoid all the sick kids .

You should read " How to Raise a Healthy Child In Spite of Your Doctor " ; I think it will help you decide when a ped is really necessary (there is a group read for this book in progress on this board) .

Our ped informed us that if they (him or anyone) give a child a vaccine without his/her parents signature they could be arrested for assult with a deadly weapon .

Check your local library or maybe even your ped if you have one to find groups for you and your child to join .

This ped is a seriously not non-vax friendly if they insist you come in for a WBV a week after you just saw them and before she examines your sick child ; she just wants your daughter to " catch up " on vaxes .

vaccin

And here 's a nurse speaking of her pediatrician after birthing her 3rd child , the first 2 were vaccinated and autistic .

I can understand that you 're worried KayCSmommy but there is a reason that your doctor wanted your child to be vaccinated .

If the OP took your advice and got her child vaccinated , and god forbid , something went wrong and her child ended up chronically ill…would you or the doctor do anything about it ?

I also have a 13 year old daughter – vaccinated on schedule , 11 year old daughter , vaccinated on schedule unitl 2 month shots caused 105 fever and high pitched screaming , no more " P " Dr recommendation .

He has an MD friend in California who does n't vaccinate any of his children .

The CDC urges doctors to not miss any chance to vaccinate and says vaccines are not contraindicated for an ill child . know i do not want to vaccinate…my boyfriend is not sure if he wants to just do selective vaccination…what exactly are the vaccines they want us to get ?

Even if you know you will never vaccinate your children under any circumstances , that is n't info your ped has to know the first visit .

For other vaccinations…My childhood records have been lost (I was fully vaccinated as a child) so my doctor did titers for all the diseases that they wanted vax info for and the titers showed I had immunity so I had no issues at all .

Most doctors view not vaccinating as child neglect and would turn your child over to CPS asap .

Mothers are very involved , of course doctors swear by their unscientific theories , but only about 1/3 of all children are fully vaccinated here .

The second caller was a doctor who said that 1/3 of physicians in this country do n't vaccinate their children , he has never seen the need to vaccinate anyone in his practice , then they cut him off and ended the show .

The second caller was a doctor who said that 1/3 of physicians in this country do n't vaccinate their children , he has never seen the need to vaccinate anyone in his practice , then they cut him off and ended the show .

The reason why they start to vaccinate early in the US is because moms are more afraid that very young children will get sick and so they are more inclined to bring them to the doctor .

Do not ask your pediatrician to confirm diagnostically the illness , because then they have to report it and have on record that a child under their supervision is not being vaccinated .

So if you did the research and decided what was important to you that your daughter be vaccinated for , then take that list to any pediatrician , you can only allow them to do certain ones .

When I talked to our old pediatrician about vaccines and told him I did not want any for my children , he said to me " I understand your concerns , but I think you should vaccinate your kids for meningitis .

Me : (long winded defensive rapid reasons why I do n't want to vaccinate my dd , medical and personal reasons…. * * shakes in boots Nurse : Relax honey , I will send out an exemption for you ~ Then she went on to have a 20 minute conversation with me about how she thinks we should n't vaccinate either , and that most parents who feel they have to vax are not fully informed , if they were , they would n't put there children through it so much and at such an early age.. she then told me that only 1 of her 5 children got vaccinated , and that was only because she thought she HAD to vaccinate the first one .

Me : (long winded defensive rapid reasons why I do n't want to vaccinate my dd , medical and personal reasons…. * * shakes in boots Nurse : Relax honey , I will send out an exemption for you ~ Then she went on to have a 20 minute conversation with me about how she thinks we should n't vaccinate either , and that most parents who feel they have to vax are not fully informed , if they were , they would n't put there children through it so much and at such an early age.. she then told me that only 1 of her 5 children got vaccinated , and that was only because she thought she HAD to vaccinate the first one .

Me : (long winded defensive rapid reasons why I do n't want to vaccinate my dd , medical and personal reasons…. * * shakes in boots Nurse : Relax honey , I will send out an exemption for you ~

Then she went on to have a 20 minute conversation with me about how she thinks we should n't vaccinate either , and that most parents who feel they have to vax are not fully informed , if they were , they would n't put there children through it so much and at such an early age.. she then told me that only 1 of her 5 children got vaccinated , and that was only because she thought she HAD to vaccinate the first one .

Me : (long winded defensive rapid reasons why I do n't want to vaccinate my dd , medical and personal reasons…. * * shakes in boots Nurse : Relax honey , I will send out an exemption for you ~ Then she went on to have a 20 minute conversation with me about how she thinks we should n't vaccinate either , and that most parents who feel they have to vax are not fully informed , if they were , they would n't put there children through it so much and at such an early age.. she then told me that only 1 of her 5 children got vaccinated , and that was only because she thought she HAD to vaccinate the first one .

told

A doctor whom I truly admire told me (kindly) that if I had been alive during the polio epidemics and had seen people in the iron lungs (like she had) , I too would be completely on-board with vaxing my kids .

Her assistant doctor that was a family doctor not pediatrician was who we always almost always saw and he really pushed immunizations and told me they were mandatory and was the one who gave my son the flu shot when I told him that I did n't want him to have it .

Her assistant doctor that was a family doctor not pediatrician was who we always almost always saw and he really pushed immunizations and told me they were mandatory and was the one who gave my son the flu shot when I told him that I did n't want him to have it .

The nurse there told me she would re-vax her son for HepB when he looked at girls , meaning she knew then in 1995 that the HepB in infants was n't going to hold .

Can you believe my son 's (FORMER) doctor told me just that ?

Our pediatrician (who is usually very laid-back when it comes to vaxing , and has even told us he thinks Hep B and polio are useless) is pushing us to get these for our son , and my DH is thinking we should take his advice .

The school nurse that has jurisdiction over the daycare because it is run by the school district called her today and told her that now she has to have her son get all of his vaccinations or he wont be able to go to school or daycare anywhere (which I know is n't true) .

When I talked to our old pediatrician about vaccines and told him I did not want any for my children , he said to me " I understand your concerns , but I think you should vaccinate your kids for meningitis .

Me : (long winded defensive rapid reasons why I do n't want to vaccinate my dd , medical and personal reasons…. * * shakes in boots Nurse : Relax honey , I will send out an exemption for you ~ Then she went on to have a 20 minute conversation with me about how she thinks we should n't

vaccinate either , and that most parents who feel they have to vax are not fully informed , if they were , they would n't put there children through it so much and at such an early age.. she then told me that only 1 of her 5 children got vaccinated , and that was only because she thought she HAD to vaccinate the first one .

exactly I wish all parents , not just the non-vaxers , would keep their sick kids at home. Â Â Why would you think it would be helpful if only the non-vaxers kept their sick kids at home ? Â This comment screams to me that you assume that vaxed kids do n't get sick and/or are incapable of spreading disease. Â You specifically mention pertussis-are you aware that the vaccine does n't prevent transmission of whooping cough ? Â That means that vaxxed kids can and do contract pertussis and are capable of spreading it to others. Â In fact , the stats from the so called oubreak in CA confirms that the vast majority of confirmed pertussis cases were in vaxxed persons. Â Â Â And it 's because we 've done our re s earch that we do n't vax. Â Through our research my family has learned so much that directly contradicts what the pro-vaccine doctors have told us. Â I think it comes down to making an informed decision based on your own particular set of circumstances and how you perceive the risks vs. the benefits .

Our pediatrician accepts my choice without criticism ; actually will not vax according to the AAP schedule. Â Other medical professionals have told me to not vax before age 3 because the immune system is more mature by then. Â I also know several medical pros who will not vax their own kids or do it on an alternative schedule. Â I do feel that there is a link between autism ; the current vaccine schedule , although anyone has yet to prove it. Â There is a very serious reason that 1 in 110 (vs 1 in 15000 kids decades ago) have autism in this country ; I doubt it has anything to do with new diagnosis standards as some " experts " suggest .

I told her I was hearing this third-hand , but I needed to verify whether the pediatrician was refusing to authorize therapy because my children were not vaccinated .

My friend says her dr told her the reason they give the chicken pox vax is because 1 in every 100 kids who gets chicken pox gets flesh eating disease .

Kiddo had a doctor who tried to force vaccines. Â I explained that we wanted to delay and were only getting two that day. Â She kind of freaked out and ended up ordering an extra one for us without telling me. Â I had told the nurse prior to talking to this new doctor so she popped in and double checked to see if I really did change my mind .

I have a friend who had CPS called on her for not doing exactly what the doctors were saying about her son , so I was really freaked out about trying to get out of anything she told me I " had " to do .

I said them : I am nursing mom and I plan a baby , but doctor told me that vaccinations are safe for me , my son and future baby .

In fact , her doctor told her , when her son came down with it , that she would n't get it .

EITHER this ped is terribly ignorant (scary) or lying (not sure which is scarier…) -Angela When I told a woman (in her late 50s , her son is grown) about the whole vax issue (how parents have to fight this issue , how peds push SO many shots , etc…) , she could n't believe it .

That is one that my daughter ‘s doc ca n’t try and pressure me into at her 1 year appt on Monday (she ‘s unvaxed and will stay that way) but this is one she always pushes because ” she knew a doctor ‘s kid who went deaf because of meningitis when their was a shortage of the Hib vaccine ” but yes , I agree , that article is scaremongering at its best Yeah at My son ‘s 2 and 4 month appt when I declined HIB vax the doctor told he about the ” many deaths ” she had seen from HIB (menengitis) .

Another anecdote , by the way : when I was a kid , the pediatrician told my mom to wait with some vaccines , and gave me exemptions for others , because the ped thought I would be too sensitive to them and might have a bad reaction .

take

A family friend of ours is a Dr , she said if you want your kids to catch a cold the best place to take them is Walmart or the mall in flu season because of ventilations systems there .

When I would take my kids to well visits I always felt like they got sick from the doctors office !

I also remember seeing stuff in those junk mags in the supermarket line about them not taking their kid to the doctor , like it was a big scandal or something .

No one has to take the child to the doctor unless there is a very serious illness and that is up to your discretion .

I wo n’t be taking my son back to the doctor anytime soon , although this doctor did see fluid in his ears and insist on giving him antibiotics although his dad ‘s side of the family has allergies to them .

I posted on your thread in H ; H. I would n’t bother taking a child with pertussis to the doctor .

I ‘ve only heard about 1 ped in the area who is okay w/ not-vaxing , and that was n’t the one he gave me (that one is n’t taking new patients) . aksed a question a few days ago about my 4 year old son .

Another little note though , and I do n’t know if this is ” standard ” for well child visits : The ped was almost done checking her out , and then she said , ok , I am going to need you to take off your pants I just need to check your privates .

I do n’t even take my kids to the doctor unless they ‘re actually sick !

I didnt want to take him into get vaxed , but the dr * assured * me that kids do fine , he would be fine .

Half the time when I go in for an appointment for my daughter , whether we are seeing the Neurologist , Immuniologist , or taking a ride in an ambulance , I am on POINT… because I have seen doctors screw up MANY MANY times… and I wont let it happen again.. .

Great info on tetanus That ‘s exactly why I would n’t want to take my kid to the ER , too many authoratative doctors around .

I had to take my dd for stitches last month when she split open her forehead and not a lot of

questions were asked about pediatricians , nor implications made about calling child welfare .

/hijack off , sorry went to my local WIC office today (colorado) to set up an intake appt and when she got to bringing in dd 's immunizations record I said " We do n't vax will I need to bring in a philosophical exemption " and she said " no thats fine just tell your counselor and she wo n't require that record " YAY.... now I 'm hearing horrible things from my friend about the ped I was going to take dd to so we might have to shop around for a GP... . 'm leaving in a few hours to visit DH 's dad for Thanksgiving and I 'm very concerned about the possibility of my unvaxed child catching hep B from him .

If they truly believe this child has a problem , take the child to a doctor and have her evaluated .

However , if you take your baby in with a cold , or even a fever , the doctor might still try to give the kid 7 or 8 vaccines during the visit .

Our pediatrician (who is usually very laid-back when it comes to vaxing , and has even told us he thinks Hep B and polio are useless) is pushing us to get these for our son , and my DH is thinking we should take his advice .

My daughter (4.5 yo and 42 lbs) is low and takes 3,000 IU/day...I (also low) take 6,000 daily and my doctor put me on 50,000 IU once/week for 8 weeks in addition .

My daughter (4.5 yo and 42 lbs) is low and takes 3,000 IU/day...I (also low) take 6,000 daily and my doctor put me on 50,000 IU once/week for 8 weeks in addition .

Well , you could simply cancel the check up , if the child seems happy and healthy there is no reason to take him to a doctor .

said

In countries that do n't vax for CP routinely , it 's still a disease of 80-90 year olds. Â My boys are older now-teenagers. Â The shot came out when they were preschool age and it was n't mandatory yet. Â The doctor would ask if I wanted my boys to get it and I 'd say no , because I was hoping they 'd catch it on their own. Â (this is before I learned health reasons not to give shots , etc) Â But they did n't get it and two years later I gave in and said OK. Â If I could do it again , and I found a way to expose them to it , I would for sure ! Â It 's good for them to get it when they 're young , that way they have lifetime immunity and wo n't Â have any chance of getting Â it when they 're older. Â I wished my boys would have got the chicken pox on their own , way back then. Â Chicken pox is n't a big deal to get. Â It 's a pain , but they get over it just like they do colds and the flu. Â Plus it 's better to get it young before they 're in school and have to be out , or miss whatever other activities they may be into as an older child .

And like you said , the immunity wears off (if it 's even there to start with , DTaP is n't a very effective vax to start with) so you 're susceptible to it , your DH is susceptible to it , your child 's dr is , etc etc etc unless you 've all been keeping up with the boosters (which I think are now or will soon be recommended every 5 years instead of 10) .

Again , it was also surprising that our ped said that the measles that came from the shot was NOT

contagious , leading me to think that many kids must get asymptomatic measles .

Her child got rubella and the dr said ” see this is what happens when you dont vaccinate .

Last year my kids came down with CP right on Easter.I took them in warning the Dr that I thought it might be CP.The Dr looked at them and said it was just their eczema and prescribed hydrocortisone and benadryl.I asked him twice , at this point they were covered from head to toe in a fine little rash with blisters sprinkled throughout.He said no and that my oldest was just fine going back to school.My oldest still did n’t feel right so I kept him home and treated it like pox and sent pictures to my mom and a friend of my sister ‘s who is in med school , both said its def .

Last year my kids came down with CP right on Easter.I took them in warning the Dr that I thought it might be CP.The Dr looked at them and said it was just their eczema and prescribed hydrocortisone and benadryl.I asked him twice , at this point they were covered from head to toe in a fine little rash with blisters sprinkled throughout.He said no and that my oldest was just fine going back to school.My oldest still did n’t feel right so I kept him home and treated it like pox and sent pictures to my mom and a friend of my sister ‘s who is in med school , both said its def .

Last year my kids came down with CP right on Easter.I took them in warning the Dr that I thought it might be CP.The Dr looked at them and said it was just their eczema and prescribed hydrocortisone and benadryl.I asked him twice , at this point they were covered from head to toe in a fine little rash with blisters sprinkled throughout.He said no and that my oldest was just fine going back to school.My oldest still did n’t feel right so I kept him home and treated it like pox and sent pictures to my mom and a friend of my sister ‘s who is in med school , both said its def .

The doctor said not to go to my best friend ‘s house (she has 6 kids and is a foster parent who always has sick kids coming in and out of her home) .

A family friend of ours is a Dr , she said if you want your kids to catch a cold the best place to take them is Walmart or the mall in flu season because of ventilations systems there .

Another little note though , and I do n’t know if this is ” standard ” for well child visits : The ped was almost done checking her out , and then she said , ok , I am going to need you to take off your pants I just need to check your privates .

I saw it too , OMG I was so infuriated when I saw that and when Sam , the nurse said ” we ‘ll have to get a court order ” because the mother did n’t want her daughter tested for HIV , or her children to receive AZT therapy , well that pissed me off even more .

As I said before , I have spent a long time researching vaccination and also homeopathy , there are no toxins , it is completely safe and it provides parents of unvaccinated children the opportunity to treat diseases that conventional doctors do not know how to treat anymore .

I met my Bradley instructor , and she said she had never vaxed her daughter , either , and that her daughter had never even been to a doctor , she was so healthy .

She said ” okay , we will see you in a year ” and the student doctor said ” what about their shots today and for the rest of the year ” My doctor looked at me , grinned , and then said ” she doesnt do

shots so she doesnt need to come in before a year , and look how healthy her kids are ” awesome ! !

She said ” okay , we will see you in a year ” and the student doctor said ” what about their shots today and for the rest of the year ” My doctor looked at me , grinned , and then said ” she doesnt do shots so she doesnt need to come in before a year , and look how healthy her kids are ” awesome ! !

My close friend does n’t vax her kids either and said this dr has always been supportive of her decision not to vax her children and never given her any trouble about it .

The dr came in and one of the first things out of his mouth was ” So I hear you ‘re not getting any immunizations ” I said no and then he said ” Do you want your son to die of pertussis ?

My reasoning is that the Kennedy sister who more recently died in an institution was often said by some to have been the victim of a medical doctor who talked old Joe Sr. into a frontal lobotomy (before the full prognosis for such a procedure was known) for this daughter who did not fit Joe Sr. ‘s image of a Kennedy daughter .

They said after speaking to several pediatricians , they can not ” risk ” that my non-vaxed son be around their newborn !

HepB killed my newborn neice … DO N’T LET THEM PUT THAT IN YOUR BABY ! thread………………….. feel free to delted this mods… . case anyone missed it : And from the Daily Express : ” Hundreds of autistic children with the serious bowel disease first identified by Dr Wakefield have been unable to get any treatment in the UK , and the drawn-out GMC investigation has played a major role in this disgraceful state of affairs , ” she said .

see

But for the most part , people do so many other things to screw up themselves ; their kids that I can see how it ‘s hard for many doctors to connect vaccines to things like asthma .

They ‘ve had their well child check , they really do n’t need to see the doctor again .

I also remember seeing stuff in those junk mags in the supermarket line about them not taking their kid to the doctor , like it was a big scandal or something .

I went to a different doctor although I liked my son ‘s doctor I could never get in to see her .

I wo n’t be taking my son back to the doctor anytime soon , although this doctor did see fluid in his ears and insist on giving him antibiotics although his dad ‘s side of the family has allergies to them .

He gave me the name of an MD that his children went to and did n’t see any harm in taking her in .

She said ” okay , we will see you in a year ” and the student doctor said ” what about their shots today and for the rest of the year ” My doctor looked at me , grinned , and then said ” she doesnt do shots so she doesnt need to come in before a year , and look how healthy her kids are ” awesome ! !

He does not get the pertussis vax due to a severe reaction to it , the MMR ; Chickenpox vaxes due to

them being live vaxes–his ped does n’t like to give kids with autism or other severe developmental delays live vaxes , and the flu vax because I do n’t see the point of it for him .

Half the time when I go in for an appointment for my daughter , whether we are seeing the Neurologist , Immuniologist , or taking a ride in an ambulance , I am on POINT… because I have seen doctors screw up MANY MANY times… and I wont let it happen again.. .

We see the same ped so I ‘m sure when if he gets something and in just the right amount of time she ‘ll act like I ‘m crazy for suggesting it came from the baby that she just vaxed that swaps spit with my son every day .

We only see one ped , never another in the same practice , we only schedule appts for first thing in the morning to avoid all the sick kids .

We only get to see the sanitised version after doctors have ” decided ” if it was a reaction or not this point we have decided to wait on vaxing and check for titers at 5 , however I am unsure at that point what to do if the kids are not immune at that time ?

Last year the school nurse sent home a note saying my daughter had been exposed to a classmate who had pertussis , and that we should see the doctor and put her on antibiotics prophylactically .

That ‘s not true at all. Â It ‘s interesting though , that that ‘s how you see it. Â I ‘m an Atheist…no magic thinking here. Â My son had heart surgery not long ago…ca n’t say that I ‘m anti-medical or anti-science. Â I have a friend who ‘s a doctor and they do n’t vaccinate .

So , for my on the spectrum son , should I just keep his incomplete vax form on file and tell my ped I ‘m taking a wait and see approach to vax’ing ?

I had to have my older child that was in the program seen by their peditrican before they would allow him to be in the program because I could n’t get the our doctor ‘s office here to see him without his records and his records had been ” lost ” by the hospital when we moved .

So after calling all the doctors near us I finally found one that would see my son before December 1st .

Though you know I really dont know what was around for vaccines back then : My ds has Tetralogy of Fallot,and a VSD is a part of that.He had open heart surgery on 10/5/01,at the age of 10 weeks.His pulmonary valve was removed during the surgery,but not replaced,the VSD was patched (using a dacron patch and muscle from around his heart so that it will grow with him) ,and there was extra muscle in his valves which had to be removed.He is now 5.5 years old,and has only had one hep b vax,which was forced on him in the hospital (long story,it ‘s on MDC somewhere) .He ‘s very healthy,healthier than many kids his age ! His sister was vaxed until 2.5 years old,and she is always sicker longer than her brother.It took a little searching,but he has a ped that is wonderful.He ‘s a homeopath as well,and we have to travel an hour to see him (and he ‘s covered by state insurance !

i vaccinate and i would n’t subject my kid to that. Â of course it would cause some kind of reaction. Â i am sorry for your friend and sister. Â Â Thanks for the heads up ! Â I do n’t see how it could happen at my Ped .

though. Â I tell him which ones I want , the nurse brings them in , opens them. Â fills out my chart , gives me the inserts and I hold my kids so I can see how many they get .

give

In countries that do n't vax for CP routinely , it 's still a disease of 80-90 year olds. Â My boys are older now-teenagers. Â The shot came out when they were preschool age and it was n't mandatory yet. Â The doctor would ask if I wanted my boys to get it and I 'd say no , because I was hoping they 'd catch it on their own. Â (this is before I learned health reasons not to give shots , etc) Â But they did n't get it and two years later I gave in and said OK. Â If I could do it again , and I found a way to expose them to it , I would for sure ! Â It 's good for them to get it when they 're young , that way they have lifetime immunity and wo n't Â have any chance of getting Â it when they 're older. Â I wished my boys would have got the chicken pox on their own , way back then. Â Chicken pox is n't a big deal to get. Â It 's a pain , but they get over it just like they do colds and the flu. Â Plus it 's better to get it young before they 're in school and have to be out , or miss whatever other activities they may be into as an older child .

I think your doctor is giving you good advice here , and sounds very supportive of your right to choose what you want to do with your daughter , which is great .

I may also feel this way because I find all the doctor can ever do is give my child an antibiotic and we tend to do alternative medicine for that anyway .

I do n't understand how the state of Utah would even know that your child had n't been to the doctor , and what would give them the right to call you .

I wo n't be taking my son back to the doctor anytime soon , although this doctor did see fluid in his ears and insist on giving him antibiotics although his dad 's side of the family has allergies to them .

He does not get the pertussis vax due to a severe reaction to it , the MMR ; Chickenpox vaxes due to them being live vaxes–his ped does n't like to give kids with autism or other severe developmental delays live vaxes , and the flu vax because I do n't see the point of it for him .

Then , when you enroll your child , do not give the school the shot card from the doctor 's office .

Re : Albuterol……..when my son was 7 months the dr said he had a slight bout of RSV and wheezing so he prescribed me albuterol but said it was up to me to give it or not but if it was his child he wouldnt because he said it had some " yucky " side affects whatever that means .

Specifically , my kid (20 months) is going to the doctor today , and I was looking at what vaccinations , if any , it makes sense to give him ; DTaP and IPV seem to be the most sensible thing : I do want him to have these eventually ; they seem relatively safe compared to some , he 's had both before without problems .

I know his pediatrician likes this combination vaccine : fewer injections , maybe fewer unpleasant associations with doctor visit , she gets the Hep B in there (she says the only reason she prefers giving that one young is that empirically older kids end up never getting it ; this argument is n't meaningless to me) .

Go in , say you are in the midst of your research regarding vaxes and for now you are uncomfortable giving any at all , that you will be continuing your quest for what is right for you and your child and will consult with the doctor once you have decided , that if you should have questions along the way you might ask the doctor…but that until then can the doctor respect your need to research and be certain where you stand on the vaxing issue .

Many doctors tell you to give your child baby tylonal after shots to prevent a fever .

However , if you take your baby in with a cold , or even a fever , the doctor might still try to give the kid 7 or 8 vaccines during the visit .

The pediatricians here highly recommend giving your child the hepatitis A vaccine .

Â I was vaccinated with MMR at 9 months old. Â When I worked in the hospital years later , I had titers taken. Â I was not immune to measles , so I had the single measles shot again. Â Titers taken again. Â Still , I am not immune to measles. Â MMR was never studied in children under 18 months , but doctors and the manufacturer have pushed giving it younger and younger .

Just the fact that you think ALL people who vax give the Hep B at birth is kind of annoying. Â My ped did n't want my ds to have it at birth , and the FSBC he was born in does n't give vaxes at all. Â He got the Hep B at 2mo when he got the rest of his first round of shots. Â Â Â Yes , I am aware of what causes febrile seizures. Â My ds was in a great hospital where all of this was explained to me – I 'm still giving fever reducers b/c it raises MY comfort level , and makes him more comfortable while he 's ill. No , I 'm REALLY positive it had nothing to do with Vax. Â There was a viral infection going around his daycare (b/c I 'm a bad mom and I work and my ds ca n't stay home with me day in and day out and he gets exposed to those types of things) Â You are not a bad mom just because you have to put your child in daycare !

Just the fact that you think ALL people who vax give the Hep B at birth is kind of annoying. Â My ped did n't want my ds to have it at birth , and the FSBC he was born in does n't give vaxes at all. Â He got the Hep B at 2mo when he got the rest of his first round of shots. Â Â Â Yes , I am aware of what causes febrile seizures. Â My ds was in a great hospital where all of this was explained to me – I 'm still giving fever reducers b/c it raises MY comfort level , and makes him more comfortable while he 's ill. No , I 'm REALLY positive it had nothing to do with Vax. Â There was a viral infection going around his daycare (b/c I 'm a bad mom and I work and my ds ca n't stay home with me day in and day out and he gets exposed to those types of things) Â You are not a bad mom just because you have to put your child in daycare !

Just the fact that you think ALL people who vax give the Hep B at birth is kind of annoying. Â My ped did n't want my ds to have it at birth , and the FSBC he was born in does n't give vaxes at all. Â He got the Hep B at 2mo when he got the rest of his first round of shots. Â Â Â Yes , I am aware of what causes febrile seizures. Â My ds was in a great hospital where all of this was explained to me – I 'm still giving fever reducers b/c it raises MY comfort level , and makes him more comfortable while he 's ill. No , I 'm REALLY positive it had nothing to do with Vax. Â There was a viral infection going around his daycare (b/c I 'm a bad mom and I work and my ds ca n't stay home with me day in and day out and he gets exposed to those types of things) Â You are not a bad mom just because you have to put your child in daycare !

My friend says her dr told her the reason they give the chicken pox vax is because 1 in every 100 kids who gets chicken pox gets flesh eating disease .

If anything I praise the pro vax FULL information websites (or what some people like to call anti vax but I disagree with the title when they give GOOD solid info /shrug) for putting out information that makes sense logically and sure gave me answers when the CDC , drug mfg ‘s , and my daughter ‘s ex ped failed .

tell

About 3 years ago the pediatricians at NAS Corpus Christi (a naval base) were telling parents that they could not diagnose CP in vaccinated children .

I tell my son how lucky he is to have the info out there , so readily accessible , but he still thinks the doctors are not to be questioned .

I even have a friend who ‘s a nurse who I ‘ve turned over.. LOL She knew I did n’t and why.. but she ‘d never looked into it before.. she ‘s not planning on any kids right off the bat , so I ‘ve never bothered her , but i was telling her about something I heard about and she wanted to read it too , so i sent her the link.. now she does n’t like Vaccines at all .

i ‘d encourage both of you to read the book ” what your child ‘s doctor may not tell you about vaccines ” (it ‘s close to that wording) by stephanie cave .

Many doctors tell you to give your child baby tylonal after shots to prevent a fever .

Then again at 12 months and again at 18 months (when they gave the MMR in the 90 ‘s) If the doc is telling you that you are behind on visits and shots before examining a sick child I would seriously find a new doctor .

I do not tell people about my children ‘s non vax status , not even doctors .

I ‘m thinking that too…you know they ca n’t let that happen… : First they tell you they do n’t contain thimerosal , then they tell the doctors to inject in in kids anyway while it is in stock .

So , I wanted to go over my daughter ‘s shot records carefully with the school nurse so she would n’t tell me she needed to have shots that were not really required (per the footnotes on the recommended catch-up schedule) .

I was even recently informed from a doctor that the next vaccine shots my son receives , is to make sure that my sister , (who has 3 autoimmune disorders) stays clear of him for at least 30 days. Â So , where does that put EVERYONE. Â If you receive vaccines , you must be careful and if you do n’t , you are also putting others at risk. Â Personally I do vaccinate my son , but on my own chosen schedule. Â I am the kind of person (that makes a lot of people mad) that believes that I should not tell others what religion they should practice , that they should have an abortion , what they should eat , how to raise their kids , or if vaccinating your child is a must. Â BUT , do not tell me what to do either , especially the for profit government , whose only concern is money and not the people. Â So , I guess I have to first say is : # 1. Â I am tired of the ” I am going to sue you ” attitude. Â Lawyers

have become a house hold commodity and society has become a point your finger and blame others. Â I have learned some things for sure , that life is never fair , crap happens , and some times people die. Â # 2 Â I believe that vaccinating vs not vaccinating comes down to personal choice and therefore no matter what you choose you are responsible for your choice. Â Why , because either choice could hurt some one else , as true with all choices we make. Â And , after reading what I just wrote I realized that I still did not answer the question. Â I did n't have time to read the responses but here ‘s my quick two cents on the issue. Â Â First of all this is completely ridiculous .

I was even recently informed from a doctor that the next vaccine shots my son receives , is to make sure that my sister , (who has 3 autoimmune disorders) stays clear of him for at least 30 days. Â So , where does that put EVERYONE. Â If you receive vaccines , you must be careful and if you do n't , you are also putting others at risk. Â Personally I do vaccinate my son , but on my own chosen schedule. Â I am the kind of person (that makes a lot of people mad) that believes that I should not tell others what religion they should practice , that they should have an abortion , what they should eat , how to raise their kids , or if vaccinating your child is a must. Â BUT , do not tell me what to do either , especially the for profit government , whose only concern is money and not the people. Â So , I guess I have to first say is : # 1. Â I am tired of the " I am going to sue you " attitude. Â Lawyers have become a house hold commodity and society has become a point your finger and blame others. Â I have learned some things for sure , that life is never fair , crap happens , and some times people die. Â # 2 Â I believe that vaccinating vs not vaccinating comes down to personal choice and therefore no matter what you choose you are responsible for your choice. Â Why , because either choice could hurt some one else , as true with all choices we make. Â And , after reading what I just wrote I realized that I still did not answer the question. Â I did n't have time to read the responses but here ‘s my quick two cents on the issue. Â Â First of all this is completely ridiculous .

Then , I received a call from our EI service coordinator telling me that she talked to our pediatrician ‘s office and that hte pediatrician refused to authorize any more services because my children were not vaccinated .

I think what bothers me the most is that people just assume that doctors are brilliant and know everything and we should do all that they tell us to do with our children .

The doctors office was aghast that my son has not had a well child check up in about a year or longer , and immeadatly started telling me which shots we * needed * to make up .

though. Â I tell him which ones I want , the nurse brings them in , opens them. Â fills out my chart , gives me the inserts and I hold my kids so I can see how many they get .

Kiddo had a doctor who tried to force vaccines. Â I explained that we wanted to delay and were only getting two that day. Â She kind of freaked out and ended up ordering an extra one for us without telling me. Â I had told the nurse prior to talking to this new doctor so she popped in and double checked to see if I really did change my mind .

brain … nervous tissue can not rid itself of aluminum and vaccines are not regulated like other aluminum injectables… usually the aluminum in a shot is more than most can handle unless you are 250lbs + ; or somewhere in that range Find a NMD… and as usuall i tell this to everyone… STOP VACCINATING it ‘s ridiculous to do this to our children… people would not be vaccinating w50 vaccines if it were not mandatory… absolutely no one would do this… this is a fear based practice

that in the long run really does alot of damage… only profits pediatricians and pharma…. and given the consequences it is much more costly to the child and the parents in the long run… autism also causes bankruptcy.. its ‘ a very profitable result of vaccination… most of these things we vaccinate for are not life threatening for the average person , so the average person should not vaccinate for it… the next generations are going to have serious immune system problems because there will be no natural immunity to these diseases and nothing to pass to the younger generation…. as long as they have people captivated and vaccinated with fear they will come up with a vaccine for everything and the campaign will make sneezing into a deadly symptom.. .

As far as I can tell regular peds are general practice doctors for children ; if your gp sees something of concern he can recommend a specialist then .

Then said coworker takes her daughter to the pediatrician , who says , ” We ca n’t diagnose CP in vaccinated children , because it looks different , so there ‘s really no way to tell what it is .

I ‘m not sure – it ‘s been mild so I did n’t call the ped but my kids had fevers… so I called my midwife because I was n’t sure what I should do (or if I should worry) if I get a fever , and she said that the doctors she ‘s been talking to said that the only real way around here that there ‘s been a differentiation between swine flu and a regular old cold was the presence of a fever… in so many people it ‘s so mild that it ‘s hard to tell beyond that .

vax

In countries that do n’t vax for CP routinely , it ‘s still a disease of 80-90 year olds. Â My boys are older now-teenagers. Â The shot came out when they were preschool age and it was n’t mandatory yet. Â The doctor would ask if I wanted my boys to get it and I ‘d say no , because I was hoping they ‘d catch it on their own. Â (this is before I learned health reasons not to give shots , etc) Â But they did n’t get it and two years later I gave in and said OK. Â If I could do it again , and I found a way to expose them to it , I would for sure ! Â It ‘s good for them to get it when they ‘re young , that way they have lifetime immunity and wo n’t Â have any chance of getting Â it when they ‘re older. Â I wished my boys would have got the chicken pox on their own , way back then. Â Chicken pox is n’t a big deal to get. Â It ‘s a pain , but they get over it just like they do colds and the flu. Â Plus it ‘s better to get it young before they ‘re in school and have to be out , or miss whatever other activities they may be into as an older child .

It was funny when the ped tried to say it was mild due to the vax , then realized the vaxed kid actually had the worst case between the two of them .

I just switched to a new ped myself and although he is super cool with not vaxing , he is very authoritarian and thinks that my kids need to learn how to ” fit into society ” and stuff like that .

Dr Sears ‘s son , he is very open to not vaxing .

I met my Bradley instructor , and she said she had never vaxed her daughter , either , and that her daughter had never even been to a doctor , she was so healthy .

Jeez , how many visits does a 9 month old vaxed child make to the ped that the file should be so thick ?

He does not get the pertussis vax due to a severe reaction to it , the MMR ; Chickenpox vaxes due to them being live vaxes–his ped does n't like to give kids with autism or other severe developmental delays live vaxes , and the flu vax because I do n't see the point of it for him .

I didnt want to take him into get vaxed , but the dr * assured * me that kids do fine , he would be fine .

Whether or not you choose to vax your child at all after that is n't any of their business or something they even have to know , unless they have some policy of following up with the doctor 's office or something , which I do n't think would happen .

My close friend does n't vax her kids either and said this dr has always been supportive of her decision not to vax her children and never given her any trouble about it .

My close friend does n't vax her kids either and said this dr has always been supportive of her decision not to vax her children and never given her any trouble about it .

I simply stated we were not getting vaxed at their office to avoid the " fear of the doctor " I had as a child .

My kid 's ped does recommend vaxing according to the CDC guidelines , however , he has many non-vaxing or selective vaxing patients and I have never been given a hard time about it .

We see the same ped so I 'm sure when if he gets something and in just the right amount of time she 'll act like I 'm crazy for suggesting it came from the baby that she just vaxed that swaps spit with my son every day .

/hijack off , sorry went to my local WIC office today (colorado) to set up an intake appt and when she got to bringing in dd 's immunizations record I said " We do n't vax will I need to bring in a philosophical exemption " and she said " no thats fine just tell your counselor and she wo n't require that record " YAY…. now I 'm hearing horrible things from my friend about the ped I was going to take dd to so we might have to shop around for a GP… . 'm leaving in a few hours to visit DH 's dad for Thanksgiving and I 'm very concerned about the possibility of my unvaxed child catching hep B from him .

So and so doctor says that it 's just fine to vax your kids if they have only a slight fever or are on abx for other illnesses !

Our pediatrician (who is usually very laid-back when it comes to vaxing , and has even told us he thinks Hep B and polio are useless) is pushing us to get these for our son , and my DH is thinking we should take his advice .

Me : (long winded defensive rapid reasons why I do n't want to vaccinate my dd , medical and personal reasons…. * * shakes in boots Nurse : Relax honey , I will send out an exemption for you ~ Then she went on to have a 20 minute conversation with me about how she thinks we should n't vaccinate either , and that most parents who feel they have to vax are not fully informed , if they were , they would n't put there children through it so much and at such an early age.. she then told me that only 1 of her 5 children got vaccinated , and that was only because she thought she HAD to

vaccinate the first one .

exactly I wish all parents , not just the non-vaxers , would keep their sick kids at home. Â Â Why would you think it would be helpful if only the non-vaxers kept their sick kids at home ? Â This comment screams to me that you assume that vaxed kids do n't get sick and/or are incapable of spreading disease. Â You specifically mention pertussis-are you aware that the vaccine does n't prevent transmission of whooping cough ? Â That means that vaxxed kids can and do contract pertussis and are capable of spreading it to others. Â In fact , the stats from the so called oubreak in CA confirms that the vast majority of confirmed pertussis cases were in vaxxed persons. Â Â Â And it 's because we 've done our re s earch that we do n't vax. Â Through our research my family has learned so much that directly contradicts what the pro-vaccine doctors have told us. Â I think it comes down to making an informed decision based on your own particular set of circumstances and how you perceive the risks vs. the benefits .

exactly I wish all parents , not just the non-vaxers , would keep their sick kids at home. Â Â Why would you think it would be helpful if only the non-vaxers kept their sick kids at home ? Â This comment screams to me that you assume that vaxed kids do n't get sick and/or are incapable of spreading disease. Â You specifically mention pertussis-are you aware that the vaccine does n't prevent transmission of whooping cough ? Â That means that vaxxed kids can and do contract pertussis and are capable of spreading it to others. Â In fact , the stats from the so called oubreak in CA confirms that the vast majority of confirmed pertussis cases were in vaxxed persons. Â Â Â And it 's because we 've done our re s earch that we do n't vax. Â Through our research my family has learned so much that directly contradicts what the pro-vaccine doctors have told us. Â I think it comes down to making an informed decision based on your own particular set of circumstances and how you perceive the risks vs. the benefits .

took

Last year my kids came down with CP right on Easter.I took them in warning the Dr that I thought it might be CP.The Dr looked at them and said it was just their eczema and prescribed hydrocortisone and benadryl.I asked him twice , at this point they were covered from head to toe in a fine little rash with blisters sprinkled throughout.He said no and that my oldest was just fine going back to school.My oldest still did n't feel right so I kept him home and treated it like pox and sent pictures to my mom and a friend of my sister 's who is in med school , both said its def .

If the OP took your advice and got her child vaccinated , and god forbid , something went wrong and her child ended up chronically ill…would you or the doctor do anything about it ?

and if it were me in that situation , it would be pretty easy to photoshop something : When we lived in Hong Kong , we took our child to a local doctor for the required physical exam .

Here is the Hawaii immunization brochure , which states the exemption forms can also be obtained from the school : Immunization and TB code : Surprisingly , I do n't see anything about religiously exempting a child from the TB screening requirement in the code .

Even if the Church told all Catholic parents not to let their child get the MMR for instance , most

parents would have to still be required to submit a religous exemption which would exempt all vaccines .

If a parent wants to exempt their child only from the MMR , Hep A and varicella vaccines because of the aborted fetal tissue , the religious exemption would be invalid in almost every state .

I was n't clear on your posts as to whether you plan on exempting your child completely , but wanted to make you aware just case you are n't using an exemption that in Florida four (4) doses of a pertussis-containing vaccine are required unless the 4th dose is given on or after the fourth birthday .

You are not even exempting your child from anything , so it 's not even an technically an exemption letter .

ETA : Parental recall is not acceptable evidence of varicella immunity – it has to be a doctor 's note : VARICELLA (CHICKENPOX) EXEMPTION : If a physician has documented that your child has had chickenpox disease , your child can be exempted from the varicella requirement .

Illinois Vaccine Awareness Coalition (IVAC) has this to say ; " Day-Care Exemptions The Day-care religious vaccine exemption contains the language that children can exempt if the " parents object thereto on the grounds that they conflict with the tenets and practices of a recognized church or religious organization , of which the parent is an adherent or member " .

Florida medical exemption statute : (b) A physician licensed under the provisions of chapter 458 or chapter 459 certifies in writing , on a form approved and provided by the Department of Health , that the child should be permanently exempt from the required immunization for medical reasons stated in writing , based upon valid clinical reasoning or evidence , demonstrating the need for the permanent exemption ; It would be highly unlikely for a school district/health department to leave unchallenged a medical exemption attempting to exempt a child from most or all the vaccines/doses required for school entry .

I recall Beth Topp (Robert her son was injured by the hepB vaccine age 11.His photo and story is at the Whale site) became very active in helping other parents learn about exemptions.When a bunch of parents were facing expulsion of their children for not doing the hepB (this was in CA somewhere) she marched into the office and showed them where they had to sign to exempt for that vaccine.I assume no questions were asked about why that one vaccine was done when all the others were.Hopefully someone on board who has actually done this can tell what they faced.My kids have not had any vaccines,so I ca n't say but from what I have read CA is easy going and the whole point of a philos exemption is to allow a parent to pick and choose .

The exemption form for your state would only be used to exempt your child from public health law requirements related to daycare or school entry .

Should you pick the medical exemption , it would only exempt your child for the ONE vaccine that was previously a problem to his health and it needs to be reevaluated every single year .

Religious exemption If the bona fide religious beliefs of an adult or the parent , guardian or person in loco parentis of a child are contrary to the immunization requirements contained in this Chapter

, the adult or the child shall be exempt from the requirements .

A medical exemption is out of the question – you ‘d have to first find a doctor willing to exempt your child from all shots – not going to happen – and then have the medical exemption renewed every year .

Ok first of all , I ‘m quite sure that you do not need to vaccinate to enter school , you can claim an exemption , and say it is against your beliefs ” In every state of the union except two (Mississippi and West Virginia) , parents have the legal right to exempt their children from all vaccinations .

The exemption is used to exempt a child from the state immunization requirements for daycare/school entry and would be submitted to the facility/school . thought I would share this with you all : looks to me like NH is just a straight religious/medical exemption .

The philosophical exemption exists in order to exempt your child from daycare and school entry requirements .

blek : state (NM) has a philisophical exemption , but there is only one exemption form that I can find and it reads : …I hereby certify that the administration of vaccine and other immunizing agents to my child as named above is contrary to my religious beliefs , held either individually or jointly by others , and therefore request that my child be exempted from the school immunization requirements… That to me reads as a religious exemption .

A state exemption is only to exempt a child from state requirements ; while in the states , the only time an exemption would come up is when using DODs schools or daycare in the states–the OP is going to Japan .

” The religious exemption HRT Fonn 681 , JAN 92 , is only to be used to exempt a non-immunized child ” because of his family ‘s religious tenets or practices .

Here ‘s the exemption form stating you ‘re able to do this : Note : you can religiously exempt your child from the TB testing requirement .

submit

If it ‘s a scenerio where they are submitting the exemption , but the school knows the child was previously vaccinated and wants the record , then they can submit both the vax record and exemption .

If it ‘s a scenerio where they are submitting the exemption , but the school knows the child was previously vaccinated and wants the record , then they can submit both the vax record and exemption .

Even if the Church told all Catholic parents not to let their child get the MMR for instance , most parents would have to still be required to submit a religous exemption which would exempt all vaccines .

If your child will be attending a center (school) based program , then you will need to submit an

exemption .

My kids are partially vaxed and we just submitted a religious exemption to the school she will be attending this fall .

My now 8 yr old is in 2nd grade , same as my 12 yr old , his exemption was submitted with his initial school enrollment package . seen it on the health call on fox news this morning I think he was saying how its easy for kids…And how the virus is so weak..Well I dont want my lo getting the flu a weird strain beacuse of this beaing spred by some little kid that cant keep his fingers out of his nose beacuse thats what kids do pick there nose then touch everything its BS and really @ # ^ % $ # % $ makes me MAD…..I hope someone can find the link to this on fox…….I am mad now. . you for posting this paper .

Has anyone had their kids denied admission to a private school after they submitted a religious exemption ?

However , if the school is not aware of a child 's vax status , then I would suggest not bringing it up and just submit the exemption w/o any immunization records .

You would keep your child 's shot records at home and NOT submit that to the school…only your exemption from all shots .

But I heard that if a child is going to join a sport club or other activities , people will ask them to submit the exemption form .

I 'm simply curious b/c I submitted a written exemption to our preschool (that accepts unvaxed children and is funded) but they kicked it back giving me one day to fill out and have a form notorized .

they may try as there are many , many people who are terribly uneducated about not vaxing and the lasw that surround it , but all they can do is either ask you to vax your child or ask that you submit and exemption form .

The time is steadfastly approaching when I must submit an exemption letter for my daughter in order for her to stay in the local public school PreK program .

She should just submit her son 's exemption letter with his registration paperwork and not say a word unless a school official actually calls it into question .

One school my child went to , for example , had instructions for submitting and exemption (though never vocally communicated that to anyone) , while another had a strict policy that everyone must be vaccinated (that they repealed after dealing with me) .

You can submit a religous exemption letter at any time , regardless of whether a child has been previously vaccinated .

Which form would I need to submit to my child 's school to receive an exemption from this requirement ?

Additionally , should you ever move to another state where you can only submit a religious exemption , you do not want a philosophical statement being transferred to your child ‘s new school with their records .

In many districts , this is the time of year that children without the necessary vax have to get them , submit an exemption , or be kicked out of school .

continued I ‘m not sure if they can legally require proof of vaccination or exemption in your situation , but if they do , this is all you need to write (includes statute language) : My homeschooled child , [FULL NAME] will be attending [NAME OF SCHOOL] for certain activities , including , but not limited to , music and art and I am submitting this letter in order to satisfy state immunization requirements for school entry .

whom

N.Y. 1987) Sample Religious Exemption Letter (change the state law reference) : Quote : To whom it may concern ; (We / I) { First and Last name (s) } , as the { (parent (s) / guardian (s) } of (name of newborn child) are exercising (our/my) rights under the US Constitution , PUBLIC HEALTH LAW , ARTICLE 21 .

Sample Religious Exemption Letter Quote : To whom it may concern ; (We / I) { First and Last name (s) } , as the { (parent (s) / guardian (s) } of (name of newborn child) are exercising (our/my) rights under the US Constitution , PUBLIC HEALTH LAW , ARTICLE 21 .

You should try this site as well ; they also have a discussion board : Inside Vaccines Sample Religious Exemption Letter Quote : To whom it may concern ; (We / I) { First and Last name (s) } , as the { (parent (s) / guardian (s) } of (name of newborn child) are exercising (our/my) rights under the US Constitution , PUBLIC HEALTH LAW , ARTICLE 21 .

” Sample Religious Exemption Letter (change the state law reference to your state) : Quote : To whom it may concern ; (We / I) { First and Last name (s) } , as the { (parent (s) / guardian (s) } of (name of newborn child) are exercising (our/my) rights under the US Constitution , PUBLIC HEALTH LAW , ARTICLE 21 .

N.Y. 1987) Sample Religious Exemption Letter (change the state law reference to your state) : Quote : To whom it may concern ; (We / I) { First and Last name (s) } , as the { (parent (s) / guardian (s) } of (name of newborn child) are exercising (our/my) rights under the US Constitution , PUBLIC HEALTH LAW , ARTICLE 21 .

N.Y. 1987) Sample Religious Exemption Letter Quote : To whom it may concern ; (We / I) { First and Last name (s) } , as the { (parent (s) / guardian (s) } of (name of newborn child) are exercising (our/my) rights under the US Constitution , PUBLIC HEALTH LAW , ARTICLE 21 .

” To whom it may concern ; (We / I) { First and Last name (s) } , as the { (parent (s) / guardian (s) } of (name of newborn child) are exercising (our/my) rights under the US Constitution , Army Regulation 40-562 , BUMEDINST 6230.15A , AF JI 48-110 , CG COMDTINST M6230.4F , section 3.2 , para4 , and Army Regulation 608-10 , section 4-6 , para 2 , to receive Religious Exemption from Vaccination , due to our genuine and sincere religious beliefs which are contrary to the practices

herein required .

So , we 'll see what happens now To whom it may concern ; (We / I) { First and Last name (s) } , as the { (parent (s) / guardian (s) } of (name of newborn child) are exercising (our/my) rights under the US Constitution , Army Regulation 40-562 , BUMEDINST 6230.15A , AF JI 48-110 , CG COMDTINST M6230.4F , section 3.2 , para4 , and Army Regulation 608-10 , section 4-6 , para 2 , to receive Religious Exemption from Vaccination , due to our genuine and sincere religious beliefs which are contrary to the practices herein required .

Here is a sample religious exemption letter (getting the letter notarized at your bank will help it look official) : Sample Religious Exemption Letter (change the state law reference) : Quote : To whom it may concern ; (We / I) { First and Last name (s) } , as the { (parent (s) / guardian (s) } of (name of newborn child) are exercising (our/my) rights under the US Constitution , PUBLIC HEALTH LAW , ARTICLE 21 .

TIA From the link I gave you previously To whom it may concern : Re : Religious Exemption In accordance with 77 Illinois Administrative Code 655.510 , we hereby state that as parents , based on our religious beliefs , we object to the following vaccinations , including but not limited to : diphtheria , pertussis , tetanus , (DPT , DTaP) , poliomyelitis , measles , rubella , mumps (MMR) , varicella , Hib , hepatitis B for our child .

N.Y. 1987) Sample Religious Exemption Letter : To whom it may concern ; (We / I) { First and Last name (s) } , as the { (parent (s) / guardian (s) } of (name of newborn child) are exercising (our/my) rights under the US Constitution , PUBLIC HEALTH LAW , ARTICLE 21 .

Sample Religious Exemption Letter (change the state law reference to your state) : Quote : To whom it may concern ; (We / I) { First and Last name (s) } , as the { (parent (s) / guardian (s) } of (name of newborn child) are exercising (our/my) rights under the US Constitution , PUBLIC HEALTH LAW , ARTICLE 21 .

To whom it may concern ; (We / I) { First and Last name (s) } , as the { (parent (s) / guardian (s) } of (name of newborn child) are exercising (our/my) rights under the US Constitution , to receive Religious Exemption from Vaccination , ALL injections , prophylaxis , ; testing due to our genuine and sincere religious beliefs which are contrary to the practices herein required .

N.Y. 1987) ; a mp ; n bsp ; Sample Religious Exemption Letter : To whom it may concern ; (We / I) { First and Last name (s) } , as the { (parent (s) / guardian (s) } of (name of newborn child) are exercising (our/my) rights under the US Constitution , PUBLIC HEALTH LAW , ARTICLE 21 .

" Sample Religious Exemption Letter (change the state law reference) : Quote : To whom it may concern ; (We / I) { First and Last name (s) } , as the { (parent (s) / guardian (s) } of (name of newborn child) are exercising (our/my) rights under the US Constitution , PUBLIC HEALTH LAW , ARTICLE 21 .

And that doctrinal support BS is old : Sample Religious Exemption Letter Quote : To whom it may concern ; (We / I) { First and Last name (s) } , as the { (parent (s) / guardian (s) } of (name of newborn child) are exercising (our/my) rights under the US Constitution , PUBLIC HEALTH LAW , ARTICLE 21 .

N.Y. 1987) ; n bsp ; Sample Religious Exemption Letter : To whom it may concern ; (We / I) { First and Last name (s) } , as the { (parent (s) / guardian (s) } of (name of newborn child) are exercising (our/my) rights under the US Constitution , PUBLIC HEALTH LAW , ARTICLE 21 .

So we submitted the school vaccination form (which allows for all exemptions) with the religious exemption signed , along with a signed copy of this letter : To whom it may concern ; (We / I) { First and Last name (s) } , as the { (parent (s) / guardian (s) } of (name of newborn child) are exercising (our/my) rights under the US Constitution , Army Regulation 40-562 , BUMEDINST 6230.15A , AF JI 48-110 , CG COMDTINST M6230.4F , section 3.2 , para4 , and Army Regulation 608-10 , section 4-6 , para 2 , to receive Religious Exemption from Vaccination , due to our genuine and sincere religious beliefs which are contrary to the practices herein required .

Re : Religious Exemption 77 Ill. Adm. Code 655.510 To whom it may concern : In accordance with 77 Illinois Administrative Code 655.510 we hereby state that as parents , based on our religious beliefs , we object to the following vaccinations , including but not limited to : measles/mumps/rubella (MMR) , Hepatitis A , and varicella for our child , NAME .

Box 149347 Mail Code 1933 Austin , Texas 78714-9347 voice : (512) 341-4400 fax : (512) 341-4447 toll-free-in-state : 1-800-WIC-FOR-U or 1-800-942-3678 toll-free-out-of-state : 877-341-4491 email : mike.montgomery @ dshs.state.tx.us website : Oh , I have another goody for you : Sample Religious Exemption Letter (change the state law reference) : Quote : To whom it may concern ; (We / I) { First and Last name (s) } , as the { (parent (s) / guardian (s) } of (name of newborn child) are exercising (our/my) rights under the US Constitution , PUBLIC HEALTH LAW , ARTICLE 21 .

requir

I thought you are only required to get an exemption if your child is attending school .

/hijack off , sorry went to my local WIC office today (colorado) to set up an intake appt and when she got to bringing in dd 's immunizations record I said " We do n't vax will I need to bring in a philosophical exemption " and she said " no thats fine just tell your counselor and she wo n't require that record " YAY…. now I 'm hearing horrible things from my friend about the ped I was going to take dd to so we might have to shop around for a GP… . 'm leaving in a few hours to visit DH 's dad for Thanksgiving and I 'm very concerned about the possibility of my unvaxed child catching hep B from him .

Even if the Church told all Catholic parents not to let their child get the MMR for instance , most parents would have to still be required to submit a religous exemption which would exempt all vaccines .

I was n't clear on your posts as to whether you plan on exempting your child completely , but wanted to make you aware just case you are n't using an exemption that in Florida four (4) doses of a pertussis-containing vaccine are required unless the 4th dose is given on or after the fourth birthday .

They were n't asking for an exemption b/c it 's not required to get EI services unless your child was going to receive services at a center-based program like a special education toddler or preschool program/school .

In fact , the rules include a provision that requires all child care facilities and elementary/secondary schools to use the official MDH record form or a similar document approved by MDH when communicating to parents regarding immunizations requirements and exemption procedures .

The provisions of this chapter shall not apply if : (1) In the absence of an epidemic or immediate threat thereof , the parent or guardian of the child shall object thereto in writing on grounds that such immunization or testing conflicts with his religious tenets and practices ; or (2) Certification by a competent medical authority providing individual exemption from the required immunization or testing is presented the admissions officer of the school .

For example , if you plan to give your daughter the rubella vax when she is nine , if she tests negative for rubella , but the law requires that all children be vaxed for rubella at age 12 months and then again at 5 years , unless you go underground or get a medical exemption , you are out of luck .

I plan on no vaxes for this new baby and if needed I will get the exemption , but TX requires an exemption request sent to the Board of Health including your child ‘s name and birth date before you can get an exemption .

A FREE way to get her out of the booster : you can assume that she ‘s one of the 95 out of 100 kids who is now immune to M , M , and R , and simply file an exemption for your state and get her into school without any boosters required .

I have 3 children , and all have had the minimum vaccines required in NYS to avoid needing an exemption .

The schools are required to keep paperwork on the children and that ‘s all the NJ school cared about – that they had the exemption paperwork on file in case they got audited .

So you would be taking a risk by telling My daughter did home daycare and the required paperwork included either proof of immunization or an exemption form .

yes If they require the shot records of your child they must also offer the religious exemption .

I have never known anyone who used them . have been under the impression that an actual vax exemption is only required in Vermont if your child is in public school- but the other day our ped .

I researched just for a few days , quoted some state and federal laws regarding the exemption pertaining to our state , and i learned some things , like athesim is considered a religion ie athetist can file religious exemptions and that only one parent has to be ‘religous ‘ to file the claim which is good because dh is an atheist and I loosely follow unitarian beliefs… Â but i would n’t lie about being vaccinated for things that ds or dd is not , i do however forgo that ds has been vaccinated for the first 9 months as it would bring up a lot of questions of his partial vax status and i do n’t want to go into that.. Â Would I knowingly violate the law , in order to receive public school benefits ? Â Nope. Â There ‘s even less of an excuse if you have a religious exemption available to which you adhere. Â The schools are required to monitor the vaccination status of children under their care , and you ‘d be interfering with that , and for no good reason .

Trust me , do n’t bother with a medical exemption – it will never get approved : (b) A physician

licensed under the provisions of chapter 458 or chapter 459 certifies in writing , on a form approved and provided by the Department of Health , that the child should be permanently exempt from the required immunization for medical reasons stated in writing , based upon valid clinical reasoning or evidence , demonstrating the need for the permanent exemption ; Since the exemption does n't expire , you can get it now or later .

Florida medical exemption statute : (b) A physician licensed under the provisions of chapter 458 or chapter 459 certifies in writing , on a form approved and provided by the Department of Health , that the child should be permanently exempt from the required immunization for medical reasons stated in writing , based upon valid clinical reasoning or evidence , demonstrating the need for the permanent exemption ; It would be highly unlikely for a school district/health department to leave unchallenged a medical exemption attempting to exempt a child from most or all the vaccines/doses required for school entry .

In some states , homeschoolers are also required to do the same vaccine paperwork as school kids (ie exemption or proof of vaccines) , but in most states HSers are " under the radar " in this regard .

I 'll doublecheck anyway but a friend of mine who hs her son was required to do an exemption (they completely do n't vax) .

attend

I thought you are only required to get an exemption if your child is attending school .

If your child will be attending a center (school) based program , then you will need to submit an exemption .

My kids are partially vaxed and we just submitted a religious exemption to the school she will be attending this fall .

The only reason you need an exemption is for your child to attend school (or preschool) .

Alabama has a religious exemption and your children can attend school .

In my state,I can not get an exemption for my son.They are not given unless a child is enrolled in school or daycare.I will need one if he attends preschool next year (I 'm considering this,depending on where he will be placed) .But,if you can get an exemption in your state,definatly do it.Just to CYA .

State law mandates what vaccines must be received at what ages for children to attend child care programs without an exemption .

Others , like tetanus , are less of a danger due to better hygeine . son is now attending child care 3 days a week and the sitter needs an exemption by end of next week to continue to sit for me .

They are under no obligation to accept a child who presents an exemption . 're currently homeschooling but are looking into a Catholic school for our two oldest to attend after we move .

The services are governed by a joint instruction (AKA regulation) that allows for religious exemption for children attending DOD-sponsored schools or daycare centers – see 3-2.b (4) on page 10 of that document .

For children attending school in Ontario , a written immunization record or proof of immunization is required , by law , for diphtheria , tetanus , polio , measles , mumps and rubella unless there is a valid written exemption .

You might want to tell her that in most states she can file an exemption and her kids can attend school without any vax 's .

It would not be because the government told me I had to or my child would need an exemption to attend school .

The same day care my unvaxed kids attended with a religious exemption that they did not even question " forced " a mom I know to do vax to be admitted at all .

continued I 'm not sure if they can legally require proof of vaccination or exemption in your situation , but if they do , this is all you need to write (includes statute language) : My homeschooled child , [FULL NAME] will be attending [NAME OF SCHOOL] for certain activities , including , but not limited to , music and art and I am submitting this letter in order to satisfy state immunization requirements for school entry .

My DS2 has a medical exemption for all vaccines until his first birthday due to a casein allergy (dr can only write the exemption for one year at a time , that 's why it 's only until his first b-day) and they tried to tell me that he could n't attend family child care .

The baby could/will die with out them , the child wo n't be able to attend school , the mom will be fired as a patient , different treatments in the ER , she 'll never be able to take the baby out of the house , there 's a rise of x and x disease , the baby will catch stuff from people from other countries , you ca n't pick and choose vaxes and use an exemption , ect .

You only need to have an exemption if your child will be attending daycare or school.My dd was fully vaxed until 2.5 years.I used a religious exemption for her to start kindergarten this year (we pulled her out for many reasons later) .You do n't have to tell anyone what your child has already had.I never told them,but if they had found out I would have explained that I had changed my religious beliefs.I hope this helps ! !

I also wonder what effect mixing the SA with another acidic material (citric acid in OJ , for instance) would have on its efficacy , as you would likely get cross-reactions . unvaccinated 21 month old daughter will be attending preschool in a few weeks , and I plan on submitting the religious exemption form for the state of CT .

Kids undergoing chemo still attend school From my reading , the school was saying they needed proof of vax or exemption .

enrol

I enrolled my daughter into a Catholic pre-school in NJ and turned in a religious exemption…about a month later I received a letter from the principal stating " the Bishops of New Jersey have mandated that requests for religious exemption from the requirements for immunizations will not be granted " .

I 've heard that you can not do a selective schedule and still enroll your child in public school with an exemption .

I plan to get my exemption tomorrow since my son will be enrolled in daycare in the next few weeks .

It 's odd , because in general it 's quite lax about parental choice (you can enroll kids in school with a philosophical exemption to vaccinations , for example , and it does n't have to be to all vaccines .

) Â Anyhoo , the school nurse at my youngest child 's school wrote me a letter informing me that her vaccine exemption is about to expire , and that she wo n't be allowed to enroll into 1st grade without an up to date exemption .

Here 's something else I found from John Hopkins : Take note about what it says regarding nursing mothers . have never ever had any issues with enrolling my daughter in any school in all of the states we lived in before (including military schools) , using a vaccination exemption .

If I am correct , you could possibly enroll your child into a private school and print yourself up a nice religious exemption of some sort .

In my state,I can not get an exemption for my son.They are not given unless a child is enrolled in school or daycare.I will need one if he attends preschool next year (I 'm considering this,depending on where he will be placed) .But,if you can get an exemption in your state,definatly do it.Just to CYA .

When our non-vaxing friends were enrolling their son in Kindy , they just said they needed the exemption forms and the lady gave it .

But if you are enrolling a child in school for the first time with an exemption , the school does n't need to know about prior vax status .

In other words , you enroll an unvaxed kid – you 'd better get an exemption into that child 's file or else you 're not in compliance .

Otherwise , they are not obligated to enroll every child who walks in with an exemption .

She made all sorts of disagreeable noises from the moment she deduced my son was on an exemption (" oh , well , I do n't know about THAT..We have a pg teacher here and , blah , blah , blah ") I had to enroll him the first day and she could n't say didly about his legal , notarized exemtion (though I could tell she really wanted to !

Things may be slightly different for you because you have enrolled your kids in a private setting which gives them more control and power , but I still think that in the end legally you can claim

whatever you want regarding religious exemption , whenever you want .

While you do n't have to write your exemption explanation until your son is ready to be enrolled , it helps to write a quick statement that " we have religious objections to the practice of vaccination " and give that to your new doc to put in their file .

Those requirements being : in order for a child to remain enrolled in a licensed setting , a immunization record or an exemption must be provided by the parent in order to comply with public health law .

No , the school is not required to enroll any child who comes in with an exemption .

The only thing they 're required to do is comply with public health and education law – if a child is enrolled without all the required immunizations , then an exemption must be kept on file in order for the child to be enrolled and remain in school .

The only thing they 're required to do is comply with public health and education law – if a child is enrolled without all the required immunizations , then an exemption must be kept on file in order for the child to be enrolled and remain in school .

If I did n't see one , I 'd enroll my child and submit the card the state requires be followed , filling out the exemption portion .

file

Their 2 yr old brother is vaccine free , and he too will have the exemption filed when the time comes . (due in 7 weeks) will be our first child that is completely not vaccinated .

" I would send the exemption back stating that you want it filed with your child 's records .

Imagine explaining to your child , " well you had this happen becasue you had a reaction to a vax , I knew it might be bad for you but figured i 'd flip a coin and hope for the best.. sorry " … trust me.. its hard , I in a way had to do that with my son , except i was able to blame his father since I had filed an exemption and he went over my head and got the vax anyway , then DS had a seizure .

From what I 've ascertained , vaxing is NOT compulsory in Australia but they will ask for either the documentation that your child has been vaxed upon registering for school/medicare or for the conscientious exemption form that you can file with the gov't .

(currently , my kids are on a conscientious exemption , but at the time , they were n't on any exemption since 1. they are unschooled and 2. only religious and medical were available then (though I would have filed for a religious if it came to that/have used that in another state before) They 've only had one check-up (medical ; we 've used the dental regularly) since we started getting it , just a few mths ago (neither having seen a dr for illness or check-up in 6 yrs !

I sent my DH with my son , and had told my DH I 'd rather the doctor left off what vaxes our son already had , in case the school get in a huff about why he had some , but not all the vaxes , yet we are filing an exemption .

I have n't filed the exemption with the older children 's schools yet .

! completely non vaxed kids are 2 and 4. Â As we start to look at public Kindergarten for my son , I 'm looking over the forms. Â Â I know I 'll probably just state my religious beliefs and get an exemption based on that , but I ca n't help thinking it 'd be SO much easier to just fill out the form as it 's " supposed " to look and sign a Â non existant Â doctor 's name. Â I live in a metro area and I ca n't imagine they call all the doctors on all the kids that come into the school. Â I imagine they just check to see that the form filled out and they file it. Â Â NO ONE knows we do n't vaccinate. Â We have n't told friends or neighbors or any one. Â Â And if we were caught , so what ? Â To sign a name for someone that does n't exist is n't forgery. Â And , there 's no law saying you have to tell the truth on school forms. Â Overall , the only places you legally ca n't lie is under oath in a court of law. Â Â Any lawyers out there ? Â Whadya think ? Â Personally Â I would not do that – unless totally backed into a corner and felt I had NO other options whatsoever .

) Â Â But , I guess it 's just an academic question since I will file for an exemption based on my religious beliefs. Â I agree with what others said , that the lie would have to be perpetuated for 12 years and what about if my son started saying the truth when it comes up in science class or wherever. Â He 'd probably be the one to spill the beans. Â It might not be so big a deal .

I researched just for a few days , quoted some state and federal laws regarding the exemption pertaining to our state , and i learned some things , like athesim is considered a religion ie athetist can file religious exemptions and that only one parent has to be 'religous ' to file the claim which is good because dh is an atheist and I loosely follow unitarian beliefs… Â but i would n't lie about being vaccinated for things that ds or dd is not , i do however forgo that ds has been vaccinated for the first 9 months as it would bring up a lot of questions of his partial vax status and i do n't want to go into that.. Â Would I knowingly violate the law , in order to receive public school benefits ? Â Nope. Â There 's even less of an excuse if you have a religious exemption available to which you adhere. Â The schools are required to monitor the vaccination status of children under their care , and you 'd be interfering with that , and for no good reason .

I researched just for a few days , quoted some state and federal laws regarding the exemption pertaining to our state , and i learned some things , like athesim is considered a religion ie athetist can file religious exemptions and that only one parent has to be 'religous ' to file the claim which is good because dh is an atheist and I loosely follow unitarian beliefs… Â but i would n't lie about being vaccinated for things that ds or dd is not , i do however forgo that ds has been vaccinated for the first 9 months as it would bring up a lot of questions of his partial vax status and i do n't want to go into that.. Â Would I knowingly violate the law , in order to receive public school benefits ? Â Nope. Â There 's even less of an excuse if you have a religious exemption available to which you adhere. Â The schools are required to monitor the vaccination status of children under their care , and you 'd be interfering with that , and for no good reason .

When your kids go to school you will have to file an exemption .

Â From the description , it sounds like your folks have been taking your DS for his well-child visits , and making sure that has the necessary medical forms filled out for school and camp and such. Â If you have not filed paperwork for a religious exemption from vaccine requirements , and there is no existing medical contraindication , vaccines are required for school enrollment. Â They ca n't just pass up the tetanus jab and the MMR and still send your kid to school. Â The flu is optional , but if

you did n't discuss it with them , I can see why they may have made that choice. Â Â Â Your situation has certain trade-offs in it. Â You can not ask your parents to , essentially , take custody of your child , and still expect to make all the decisions .

Just keep feeding your kids immunity boosting foods and filing whatever letter is needed to claim the exemption and do n't feel that you have to explain to people .

You might want to tell her that in most states she can file an exemption and her kids can attend school without any vax 's .

we 're in florida , we have n't filed an exemption because our ped does n't think healthy children should be vaxed either (although he WILL vax them even though he agrees with us that our children do n't need vaxed) and my kids are homeschooled .

Just go in and tell her point blank that you 'll be filing a Religious Exemption , and that your children have never had any vaccines previously .

If your child is currently in daycare and you begin to deviate from the schedule , you will need to file for an exemption .

One of my good friends is also sending her daughter to preschool this year and she tried to tell me the MMR was required for preschool (she 's pretty pro-vax) , I mentioned that if that were true I would just file an exemption form or just not send my child to that school .

Sent a prayer up for you that you ; your child will be protected during this time.Hugs,Catherine Here is a website that will help you with filing an exemption : Legal matters : This has been discussed before , pls do a search in the entire forum and vaccine forum .

exercis

N.Y. 1987) Sample Religious Exemption Letter (change the state law reference) : Quote : To whom it may concern ; (We / I) { First and Last name (s) } , as the { (parent (s) / guardian (s) } of (name of newborn child) are exercising (our/my) rights under the US Constitution , PUBLIC HEALTH LAW , ARTICLE 21 .

Sample Religious Exemption Letter Quote : To whom it may concern ; (We / I) { First and Last name (s) } , as the { (parent (s) / guardian (s) } of (name of newborn child) are exercising (our/my) rights under the US Constitution , PUBLIC HEALTH LAW , ARTICLE 21 .

You should try this site as well ; they also have a discussion board : Inside Vaccines Sample Religious Exemption Letter Quote : To whom it may concern ; (We / I) { First and Last name (s) } , as the { (parent (s) / guardian (s) } of (name of newborn child) are exercising (our/my) rights under the US Constitution , PUBLIC HEALTH LAW , ARTICLE 21 .

" Sample Religious Exemption Letter (change the state law reference to your state) : Quote : To whom it may concern ; (We / I) { First and Last name (s) } , as the { (parent (s) / guardian (s) } of (name of newborn child) are exercising (our/my) rights under the US Constitution , PUBLIC HEALTH LAW , ARTICLE 21 .

There is this : (Current Date) (Health Department Director) (Name of Health Department) (Health Department Address) (City , State , Zip) REF : Submission for Religious Exemption from Vaccination for : (Child â s name , Birthdate) Dear (Health Dept Director â s name) : I , , as the parent of the above named child am exercising my rights under the First Amendment of the US Constitution and Alabama Government Code Section 16-30-3 to receive Religious Exemption from Vaccinations .

N.Y. 1987) Sample Religious Exemption Letter (change the state law reference to your state) : Quote : To whom it may concern ; (We / I) { First and Last name (s) } , as the { (parent (s) / guardian (s) } of (name of newborn child) are exercising (our/my) rights under the US Constitution , PUBLIC HEALTH LAW , ARTICLE 21 .

N.Y. 1987) Sample Religious Exemption Letter Quote : To whom it may concern ; (We / I) { First and Last name (s) } , as the { (parent (s) / guardian (s) } of (name of newborn child) are exercising (our/my) rights under the US Constitution , PUBLIC HEALTH LAW , ARTICLE 21 .

” To whom it may concern ; (We / I) { First and Last name (s) } , as the { (parent (s) / guardian (s) } of (name of newborn child) are exercising (our/my) rights under the US Constitution , Army Regulation 40-562 , BUMEDINST 6230.15A , AF JI 48-110 , CG COMDTINST M6230.4F , section 3.2 , para4 , and Army Regulation 608-10 , section 4-6 , para 2 , to receive Religious Exemption from Vaccination , due to our genuine and sincere religious beliefs which are contrary to the practices herein required .

So , we ‘ll see what happens now To whom it may concern ; (We / I) { First and Last name (s) } , as the { (parent (s) / guardian (s) } of (name of newborn child) are exercising (our/my) rights under the US Constitution , Army Regulation 40-562 , BUMEDINST 6230.15A , AF JI 48-110 , CG COMDTINST M6230.4F , section 3.2 , para4 , and Army Regulation 608-10 , section 4-6 , para 2 , to receive Religious Exemption from Vaccination , due to our genuine and sincere religious beliefs which are contrary to the practices herein required .

Here is a sample religious exemption letter (getting the letter notarized at your bank will help it look official) : Sample Religious Exemption Letter (change the state law reference) : Quote : To whom it may concern ; (We / I) { First and Last name (s) } , as the { (parent (s) / guardian (s) } of (name of newborn child) are exercising (our/my) rights under the US Constitution , PUBLIC HEALTH LAW , ARTICLE 21 .

N.Y. 1987) Sample Religious Exemption Letter : To whom it may concern ; (We / I) { First and Last name (s) } , as the { (parent (s) / guardian (s) } of (name of newborn child) are exercising (our/my) rights under the US Constitution , PUBLIC HEALTH LAW , ARTICLE 21 .

(We / I) { First and Last name (s) } , as the { (parent (s) / guardian (s) } of (name of newborn child) are exercising (our/my) rights under the First Amendment of the US Constitution and [insert California immunization/testing exemptions birth statutes here] to receive Religious Exemption from Vaccination , ALL injections , ; testing .

Sample Religious Exemption Letter (change the state law reference to your state) : Quote : To whom it may concern ; (We / I) { First and Last name (s) } , as the { (parent (s) / guardian (s) } of (name of newborn child) are exercising (our/my) rights under the US Constitution , PUBLIC

HEALTH LAW , ARTICLE 21 .

To whom it may concern ; (We / I) { First and Last name (s) } , as the { (parent (s) / guardian (s) } of (name of newborn child) are exercising (our/my) rights under the US Constitution , to receive Religious Exemption from Vaccination , ALL injections , prophylaxis , ; testing due to our genuine and sincere religious beliefs which are contrary to the practices herein required .

N.Y. 1987) ; a mp ; n bsp ; Sample Religious Exemption Letter : To whom it may concern ; (We / I) { First and Last name (s) } , as the { (parent (s) / guardian (s) } of (name of newborn child) are exercising (our/my) rights under the US Constitution , PUBLIC HEALTH LAW , ARTICLE 21 .

” Sample Religious Exemption Letter (change the state law reference) : Quote : To whom it may concern ; (We / I) { First and Last name (s) } , as the { (parent (s) / guardian (s) } of (name of newborn child) are exercising (our/my) rights under the US Constitution , PUBLIC HEALTH LAW , ARTICLE 21 .

(We / I) { First and Last name (s) } , as the { (parent (s) / guardian (s) } of (name of child) are exercising (our/my) rights under the First Amendment of the US Constitution , Oregon Revised Statutes 333-020-0165 (1) , and 433.306 (2) to receive Religious Exemption from Vaccination , ALL injections , ; testing .

And that doctrinal support BS is old : Sample Religious Exemption Letter Quote : To whom it may concern ; (We / I) { First and Last name (s) } , as the { (parent (s) / guardian (s) } of (name of newborn child) are exercising (our/my) rights under the US Constitution , PUBLIC HEALTH LAW , ARTICLE 21 .

N.Y. 1987) ; n bsp ; Sample Religious Exemption Letter : To whom it may concern ; (We / I) { First and Last name (s) } , as the { (parent (s) / guardian (s) } of (name of newborn child) are exercising (our/my) rights under the US Constitution , PUBLIC HEALTH LAW , ARTICLE 21 .

So we submitted the school vaccination form (which allows for all exemptions) with the religious exemption signed , along with a signed copy of this letter : To whom it may concern ; (We / I) { First and Last name (s) } , as the { (parent (s) / guardian (s) } of (name of newborn child) are exercising (our/my) rights under the US Constitution , Army Regulation 40-562 , BUMEDINST 6230.15A , AF JI 48-110 , CG COMDTINST M6230.4F , section 3.2 , para4 , and Army Regulation 608-10 , section 4-6 , para 2 , to receive Religious Exemption from Vaccination , due to our genuine and sincere religious beliefs which are contrary to the practices herein required .

sign

(long story) I signed a religious waiver exemption and let the daycare provider know that I would like to be informed when another child at the center (she will be in the Infant Toddler room until Oct.) is vaccinated and returns to center same day which apparently happens quite a bit .

Have the parent who did n’t sign the exemption form take the child in .

I have questions…. thanks mamdawg I ‘m definitely not signing anything I had to learn the hard way.Our son is vaccine damaged with seizures.You do n’t have to be on the defense,well

researched,or particularly articulate.JUST DO N'T SIGN ANYTHING ! I would get an exemption form (religious or philosphical) to hand to whoever to keep on file.If I had done this 5 babies ago I 'd have saved ourselves a lot of suffering and grief .

So....when she goes to sign up her kids for school , she only needs to bring the religious exemption (which also happens to be blue) and the yellow physical form from the doctor 's office . all , Well a mum on one of the parenting sites I 'm a member of said this when asked 'why ppl chose to or not to vaccinate ' She knew it was going to be a hard decision to make as when she was in hospital there was a little boy who had had a bad reaction to the chicken pox vaccine and had to be hospitalised and almost died .

I so wish I had takend my religious exemption out and said this is the only document I will sign in regards to vaccinating my children .

38) RELIGIOUS EXEMPTION FORM Religious exemption requests must contain the wording found in the Alaska Administrative Code [4 AAC06.55 (b) (3) or 4 AAC 62.450 (c) (2)] stating all vaccines must be received unless the child â   has an affidavit signed by his [4 AAC 62.450 (c) (2) says â   the child â   s â   ] parent or guardian affirming that immunization conflicts with the tenets and practices of the church or religious denomination of which the applicant [4 AAC 62.450 (c) (2) says â   the parent or guardian â   ] is a member .

Nissa aka " The " Children 's Librarian Here in CA it 's very easy , all you have to do is sign the back of the school 's vaccination form , stating you have a philosophical (or whichever) exemption .

I have my exemption in my children 's files and it would probably lessen the liklihood of your ped asking you to sign the Refusal to Vaccinate form .

The CDC here told me they would n't accept the any kids w/out shots , even with a signed exemption .

In Florida , all you have to do is go to the health department , request a religious exemption and they give you a blue form to sign (which you turn in to the school district in lieu of the blue form which the pediatrician gives you with the list of vaccines that child has received) .

I have also taken in kids in the past who were not vaxed , and they just had to get an exemption form signed .

Yea... the women who did n't want to give me the religious exemption finds another way to keep my kids out of childcare... even though the note saying to give my girls benadryl for a food allergy was signed by their pediatrician ! ! !

! completely non vaxed kids are 2 and 4. Â As we start to look at public Kindergarten for my son , I 'm looking over the forms. Â Â I know I 'll probably just state my religious beliefs and get an exemption based on that , but I ca n't help thinking it 'd be SO much easier to just fill out the form as it 's " supposed " to look and sign a Â non existant Â doctor 's name. Â I live in a metro area and I ca n't imagine they call all the doctors on all the kids that come into the school. Â I imagine they just check to see that the form filled out and they file it. Â Â NO ONE knows we do n't vaccinate. Â We have n't told friends or neighbors or any one. Â Â And if we were caught , so what ? Â To sign a name

for someone that does n't exist is n't forgery. Â And , there 's no law saying you have to tell the truth on school forms. Â Overall , the only places you legally ca n't lie is under oath in a court of law. Â Â Any lawyers out there ? Â Whadya think ? Â Personally Â I would not do that – unless totally backed into a corner and felt I had NO other options whatsoever .

I do have my religious exemption form (provided by the state…meant for children entering school) signed and notarized , so it 's sitting here waiting in case something come up .

* * Yes they do , but if you do not vax then you get an exemption form co signed by a lawyer , and you have to agree that if there are outbreaks of any disease then your kids have to stay home from school .

K In CA you just sign the exemption area on the back of the blue shot record the school will keep in your son 's records .

I recall Beth Topp (Robert her son was injured by the hepB vaccine age 11.His photo and story is at the Whale site) became very active in helping other parents learn about exemptions.When a bunch of parents were facing expulsion of their children for not doing the hepB (this was in CA somewhere) she marched into the office and showed them where they had to sign to exempt for that vaccine.I assume no questions were asked about why that one vaccine was done when all the others were.Hopefully someone on board who has actually done this can tell what they faced.My kids have not had any vaccines,so I ca n't say but from what I have read CA is easy going and the whole point of a philos exemption is to allow a parent to pick and choose .

If your kids have n't been in school yet , you could just leave the form blank and then sign the exemption waiver part .

I have never had a problem be it with school (or preschool) , you just sign the exemption of your choice on the back of the vaccination form , or with doctors , I have very little need for allopathic medicine , so choose my practioners with that in mind and have never had a problem with my children 's vax status .

I did n't have a problem with my daughter 's last preschool (I just handed them the immunization form with the exemption part signed) .

claim

I know that for the first child we could probably get away with doing the ones we want to , and then claim a change of heart and use the exemption laws to avoid having to do the rest .

He actually HAS been vax 'd (before I knew better) so I have one , BUT I am not thinking I should turn it into them since I will be claiming religious exemption for all of my children .

If so , you can claim a religious exemption and politely inform them that they are breaking the law by asking you to explain , and if they deny your son services based on this they are guilty of religious descrimination .

I am not sure on that one but most people who inject some vaccines into their child still claim a

complete exemption and just do n't mention the vaccines they gave .

I claim personal exemption for my child , (child 's name) , regarding all vaccines (or list certain ones you do n't do) .

That alarmed me , and not just for big-brother reasons : If I want to claim an exemption , but I 'm a partial/delaying vaxer , this could bust me , unless I figure out how to get my son 's name removed from it .

and she actually has a ped in Georgia who is open to it) 2) homeschool him 3) send her daughter and find a private kindergarten for her son that would be half day and she could claim religious exemption 4) try to claim her religious beliefs changed after educating herself and discovering aborted fetal tissue in vaccinations .

and she actually has a ped in Georgia who is open to it) 2) homeschool him 3) send her daughter and find a private kindergarten for her son that would be half day and she could claim religious exemption 4) try to claim her religious beliefs changed after educating herself and discovering aborted fetal tissue in vaccinations .

So if her daughters vax records are being sent as part of the previous records from the other school she can still claim exemption ?

A written statement must be included in the record of any child for whom such an exemption is being claimed .

I am just wondering if I can claim it an exemption for her after vaccinating my older three children .

One very important thing to keep in mind – if you have this letter in your child 's medical file or if the dr. knows you have these objections , it can always be used to claim you are falsely taking a religious exemption , if it results in you deciding to .

My younger daughter may be going to preschool next year and I am either going to have to immunize or claim a religious exemption , and I want my thoughts together on the topic so I can advocate for her .

My concern is whether you can claim exemption for a part of a vax series your child already received… or do you just not fill anything out and lie ?

These two instructions give more information on how and where to claim the religious exemption for your child .

Parents who have n't vaxed at all that I have spoken to say that you could probably get away with doing the vaxing you want to before school , then claiming a change of heart and taking the exemption , but they thought that this would only work for the first child , and that you probably could n't get away with it for the second child .

Just keep feeding your kids immunity boosting foods and filing whatever letter is needed to claim the exemption and do n't feel that you have to explain to people .

Had child 4 gotton MMR , I believe she would be on the Autism spectrum today . cant make me finish them right – just because i started it i dont have to get them all – i did not really research any of them and now that i have i stopped doing it You will need to claim a religious exemption if you will not be vaccinating anymore…..one can vax and stop…after all you can have a change in your religious beliefs at any time .

Things may be slightly different for you because you have enrolled your kids in a private setting which gives them more control and power , but I still think that in the end legally you can claim whatever you want regarding religious exemption , whenever you want .

” Religious â   A child â   s parent or guardian may claim exemption for religious reasons .

need

Yet another tetanus from splinter case documented on CDC (again , not a child) : I also find it interesting that they spent so much time documenting the exemption , when it would appear that besides having an infected wound that needed to be expressed and cleansed properly , he was apparently healthy for 2 weeks prior to : Wait , so FIRST they give tetanus toxoid and antibiotics and send the boy home , before removing the splinter and expressing the pus to properly cleanse the wound ?

I asked her what I needed to turn in if my son had already received some vaccinations but I have since obtained a religious exemption .

/hijack off , sorry went to my local WIC office today (colorado) to set up an intake appt and when she got to bringing in dd ‘s immunizations record I said ” We do n’t vax will I need to bring in a philosophical exemption ” and she said ” no thats fine just tell your counselor and she wo n’t require that record ” YAY…. now I ‘m hearing horrible things from my friend about the ped I was going to take dd to so we might have to shop around for a GP… . ‘m leaving in a few hours to visit DH ‘s dad for Thanksgiving and I ‘m very concerned about the possibility of my unvaxed child catching hep B from him .

So , you do not need any exemption until your child goes to school .

Also , he is 7 months old , exclusivly breastfed and has never been vaxed against anything and has never been sick – does any of that matter ? everyone , I need a religious exemption form for California for my son tomorrow .

If your child will be attending a center (school) based program , then you will need to submit an exemption .

Â And then , at some point , if you send your kids to school , someone will ask for their shot records , and you will need to fill out an exemption form .

The only time you will need to fill out an exemption is if your child attends daycare or school but it sounds like you will likely be homeschooling (as are we) and so there is no need at all .

My son had gone through the same school system as my daughter…obviously with him being

vaccinated an exemption was not necessary , but in my daughters case it needed to be provided .

Also , you do not need to get the exemption until the kids are school-aged or going to daycare .

So….when she goes to sign up her kids for school , she only needs to bring the religious exemption (which also happens to be blue) and the yellow physical form from the doctor 's office . all , Well a mum on one of the parenting sites I 'm a member of said this when asked 'why ppl chose to or not to vaccinate ' She knew it was going to be a hard decision to make as when she was in hospital there was a little boy who had had a bad reaction to the chicken pox vaccine and had to be hospitalised and almost died .

The only reason you need an exemption is for your child to attend school (or preschool) .

and people need to be aware that a school exemption form does n't " officially " render a child safe from harm in the eyes of CPS .

Directions to my house , I do n't want you coming to my house , place of employment for my husband changes every 18 months or so , family physician , hmm , we are using the religious exemption for vacc and I might want to use it for the school physical and to keep the school and health dept from screening my child at school (another noticifation that " Your child will receive…. " , the school does n't need my insurance information if there were an emergancy our local hospital treats all patients and I can bring it at that time .

Not only do they have to admit her child (she has a 14-day " grace period ") , but they can not exclude her child w/o first technically denying the exemption from immunization and then they would need to inform her (in writing) her child will be excluded from school and the date the exclusion begins .

" Â It was an LtC doctor and I was terrified he 'd look at that exemption paper and start grilling me. Â But it was very easy , he was trying to find out if there were any special needs they would need to accommodate overseas – therapy for special needs kids , chronic medical conditions , or wheel-chair access in housing , stuff like that. Â After that I was so relieved it felt like I 'd built a mountain out of a molehill. Â And after we got there , the DOD schools accepted the exemption and that was it. Â No sweat. Â But the daycare is another ballgame and I never even tried to use it after seeing their tight statement on vaxing in the paperwork .

In my state,I can not get an exemption for my son.They are not given unless a child is enrolled in school or daycare.I will need one if he attends preschool next year (I 'm considering this,depending on where he will be placed) .But,if you can get an exemption in your state,definatly do it.Just to CYA .

When our non-vaxing friends were enrolling their son in Kindy , they just said they needed the exemption forms and the lady gave it .

I plan on no vaxes for this new baby and if needed I will get the exemption , but TX requires an exemption request sent to the Board of Health including your child 's name and birth date before you can get an exemption .

But if you are enrolling a child in school for the first time with an exemption , the school does n't need to know about prior vax status .

accept

I talked to the director of the CDC (child development center) and the director of the family childcare program and both told me they do not accept any sort of exemption .

I am in CO , not CA , but CO has the three exemptions like CA , and two of my children go to private schools , neither one has any problem with accepting our exemptions , we are given the same vax [exemption] form to fill in as the public schools .

I know for a fact that the school accepts another child who has a religious exemption , so I 'm not too worried about that angle (well–I 'm somewhat worried or I would have done it by now) .

The CDC here told me they would n't accept the any kids w/out shots , even with a signed exemption .

If I have entered her into the registry can I get her out (in case we decide to selectivley vaccinate , I do n't want the school system to not accept my religious exemption) Are my other 2 children automatically in the " registry " ? ? ?

" Â It was an LtC doctor and I was terrified he 'd look at that exemption paper and start grilling me. Â But it was very easy , he was trying to find out if there were any special needs they would need to accommodate overseas – therapy for special needs kids , chronic medical conditions , or wheel-chair access in housing , stuff like that. Â After that I was so relieved it felt like I 'd built a mountain out of a molehill. Â And after we got there , the DOD schools accepted the exemption and that was it. Â No sweat. Â But the daycare is another ballgame and I never even tried to use it after seeing their tight statement on vaxing in the paperwork .

My older daughter was partially vaccinated and I was able to use the exemption with no trouble at public school (aside from judging looks…) , but I am concerned about not being accepted into day cares .

What they said was that , basically , although private schools are supposed to accept this exemption , they really do n't have to b/c they can reject your child for any reason – they do n't like you , your hair color , your politics , your choices , whatever . article : The study : I think the potential for flaws definitely exists in this study .

Once your child is accepted , submit the religious exemption just as you would the immunization record (I think the less of a deal you make of it , the less they will) .

Also , do daycares/preschools have to take my exemption , or can they refuse accepting my children ?

If they do , you can try using the exemption , but legally they do n't have to accept it and can deny your child if they choose to .

Â We have found my son a daycare center and they are sending me the enrollment forms next week. Â I have not spoken to them about vaccination. Â How do I know if they are licensed ? Â And if they are licensed to they have to accept the exemption form ?

They are under no obligation to accept a child who presents an exemption . 're currently homeschooling but are looking into a Catholic school for our two oldest to attend after we move .

The cdc did n't have a problem with my kids not being vaccinated , but they hassled me for months about getting myself re-vaccinated (they would n't accept my titer for being vaccinated several years ago or religious exemption) .

If we go , DD would be going to a DOD American school , not a private one so hopefully they 'll accept an exemption . just got a free copy of American Baby (I know , I know) and it had a blurb about " experts " now recommending not one , but TWO flu shots for babies and toddlers .

Â Wait until your child is accepted into the school before revealing that she will be using an exemption .

While MA licensed daycare providers must comply with public health law , they are not forced to accept any child whose parent walks in with an exemption .

My son goes to one but she does take part in the state food program so she had to accept our religious exemption .

My concern is that they might not accept our religious exemption and we would be forced to either vaccinate my son or he would not be accepted into the school .

My concern is that they might not accept our religious exemption and we would be forced to either vaccinate my son or he would not be accepted into the school .

have

I know that for the first child we could probably get away with doing the ones we want to , and then claim a change of heart and use the exemption laws to avoid having to do the rest .

Yet another tetanus from splinter case documented on CDC (again , not a child) : I also find it interesting that they spent so much time documenting the exemption , when it would appear that besides having an infected wound that needed to be expressed and cleansed properly , he was apparently healthy for 2 weeks prior to : Wait , so FIRST they give tetanus toxoid and antibiotics and send the boy home , before removing the splinter and expressing the pus to properly cleanse the wound ?

I enrolled my daughter into a Catholic pre-school in NJ and turned in a religious exemption…about a month later I received a letter from the principal stating " the Bishops of New Jersey have mandated that requests for religious exemption from the requirements for immunizations will not be granted " .

He actually HAS been vax 'd (before I knew better) so I have one , BUT I am not thinking I should

turn it into them since I will be claiming religious exemption for all of my children .

I asked her what I needed to turn in if my son had already received some vaccinations but I have since obtained a religious exemption .

So , in a few years I will have to endure the whole exemption thing from my kid ‘s school , but I ‘ll deal with that when it comes .

Our dd is 2 , so we have some time on the school issue and we know we will homeschool…..but the issue of exemption for children is very important for us .

Now , I have an actual conscientious exemption in my possession , so I would just whip that out , but then , I had nothing but my right as a parent whose children were not in any group setting to not vaccinate them (and the way the law is written here , and in most states I have seen , it specifies children in such settings must be vaxed , NOT children in general !

Now , I have an actual conscientious exemption in my possession , so I would just whip that out , but then , I had nothing but my right as a parent whose children were not in any group setting to not vaccinate them (and the way the law is written here , and in most states I have seen , it specifies children in such settings must be vaxed , NOT children in general !

We currently have a vax exemption on file for my daughter (we plan on eventually selective/delay/not at all depending on the vaccine .

/hijack off , sorry went to my local WIC office today (colorado) to set up an intake appt and when she got to bringing in dd ‘s immunizations record I said ” We do n’t vax will I need to bring in a philosophical exemption ” and she said ” no thats fine just tell your counselor and she wo n’t require that record ” YAY…. now I ‘m hearing horrible things from my friend about the ped I was going to take dd to so we might have to shop around for a GP… . ‘m leaving in a few hours to visit DH ‘s dad for Thanksgiving and I ‘m very concerned about the possibility of my unvaxed child catching hep B from him .

Who is requesting an exemption from you ? have chosen not to vaccinate my children for our own personal reasons .

Me : (long winded defensive rapid reasons why I do n’t want to vaccinate my dd , medical and personal reasons…. * * shakes in boots Nurse : Relax honey , I will send out an exemption for you ~ Then she went on to have a 20 minute conversation with me about how she thinks we should n’t vaccinate either , and that most parents who feel they have to vax are not fully informed , if they were , they would n’t put there children through it so much and at such an early age.. she then told me that only 1 of her 5 children got vaccinated , and that was only because she thought she HAD to vaccinate the first one .

Me : (long winded defensive rapid reasons why I do n’t want to vaccinate my dd , medical and personal reasons…. * * shakes in boots Nurse : Relax honey , I will send out an exemption for you ~ Then she went on to have a 20 minute conversation with me about how she thinks we should n’t vaccinate either , and that most parents who feel they have to vax are not fully informed , if they were , they would n’t put there children through it so much and at such an early age.. she then told

me that only 1 of her 5 children got vaccinated , and that was only because she thought she HAD to vaccinate the first one .

Even if the Church told all Catholic parents not to let their child get the MMR for instance , most parents would have to still be required to submit a religous exemption which would exempt all vaccines .

I did have one lady tell me though that her child had been at the CDC at several bases and some of the bases allowed a non-medical exemption and some did n't….so it depended on the base you were at .

laughup Speaking of newborns , here 's a good example : It 's just like the flu vaccine – vaxing the kids to protect gramma – except with pertussis you vaccinate the older kids to protect the babies I bet both work equally well love and peace . know I have seen a few simple religious exemption letters on this board and now I ca n't seem to find one .

What about kids that have an exemption ?

If you have a school exemption form for an older child , I 'd bring a copy of it .

I know for a fact that the school accepts another child who has a religious exemption , so I 'm not too worried about that angle (well–I 'm somewhat worried or I would have done it by now) .

If I remember correctly , there was a pretty strong consensus on that thread as well as any other thread I 've ever seen on the subject that you should keep your exposed children away from other children for awhile. Â There may be a few opposing views in each thread , but the idea that most people here that do n't vaccinate are just fine with exposing other children to diseases is not what I 've seen. Â Â While I do n't think that it 's legally enforceable , I do think that if someone knows that their child is ill or exposed to something serious and they willfully ignore the fact that that there is a known immuno-compromised person where they will be , there should be some kind of liability. Â I go to church and there are a lot of older people there , some that I know are battling cancer , so I do try to take extra precautions when I 'm there…washing my hands , not attending when sick , being where I wo n't have to hold hands with anyone .

I cited religious beliefs , and he has specifically stated in the paperwork as follows : Â " The Mother has failed to allow the child to be vaccinated and refuses to allow the father to administer to the child any medication including Tylenol citing religious beliefs , however , she does not attend church regularly .

Plus , the " plan " for the future at this point is that her dd will attend our parish school (even though they go to a different parish) with our kids .

I always warned the new moms against the church daycare because…. A) typically run by amateurs who do not have a good cleanliness regime B) the mixing of kids who attend regular day care during the week with other kids from other homes and centers… too many germs in my opinion So , the pattern often became for my working , churchgoing mom friends that by attending church and

putting their kids in the church nursery… the kid was often sick by Tuesday and the spousal fights would ensue about whose turn it was to stay home with the sick kid… they get the kid well , go to church on Sunday and the whole cycle repeated itself all winter and early spring long .

I havent attended church yet , but if I did I would want my kids involved…. For an hour during church , I personally do n't think twice about my unvaxed child being in a room with other kids , vaxed or otherwise . catid = 126 Because the current Prophylactic HPV vaccine is so safe and effective . thought the reason to justify the chicken pox vaccine was money , that it would save money by keeping parents at work instead of caring for a sick child .

Both my kids have attended church preschools with just a basic exemption letter and we 've had no problems .

Never mind she was a therapist working with immunocompromised children in a school setting , she still went to the grocery store , she went to church (where my children attended regularly) , and everywhere else she normally went .

He is being transferred elsewhere , and he is worried that there are not the resources to help his boys that we have here…His sons can not even attend the Church School .

Yes , it may bother me if another child gets the chicken pox especially since its the school of the church we attend .

I was especially scared though , because my kids attend a private church school at the church we belong to .

Unfortunately , money will always come before the health of children and unborn babies . nutritionist at the WIC office said that in order for my children to attend school w/o being fully vaccinated , a clergy member would have to sign a religous exemption form .

On top of the 5 in 1 , they offer prevnar and meng c at 2 , 4 and 6 months : sad I feel so lucky to have a friend who brought up vax risks and does n't vax her dd- it 's something most people do n't even know is an option- they think you have to vax Yes , we have confidence in our plumbers , but we have FAITH in our doctors ; the relationship between a doctor and a patient is a sacred relationship…it is a religious experience ! currently have a religious exemption with the school district so my daughter can attend public school unvaxed .

The church school my son goes to (the church we attend) is not accredited by the state , they are independent , so they do n't care about AJ 's vax status .

I can only have up to six kids at any one time , but we also attend a number of playgroups of over 20 kids at a time , and DD attends Sunday School at church .

(a) No principal , teacher , owner or person in charge of a school shall permit any child to be admitted to such school , or to attend such school , in excess of fourteen days , without the certificate provided for in subdivision five of this section or some other acceptable evidence of the child 's immunization against poliomyelitis , mumps , measles , diphtheria , rubella , hepatitis B , varicella and , where applicable , Haemophilus influenza type b (Hib) ; provided , however , such

fourteen day period may be extended to not more than thirty days for an individual student by the appropriate principal , teacher , owner or other person in charge where such student is transferring from out-of-state or from another country an can show a good faith effort to get the necessary certification or other evidence of immunization .

We no longer attend a church and both my kids run at the mention of ” going to church ” .

Bethany I wish I had seen a list like this earlier in my parenting….we have a close family history of all of these : vaccine reactions (step daughter)) convulsions or neurological disorders (maternal Grandpa) severe allergies (me) immune system disorders (several of us) I would have never vaxed my oldest 4 kids knowing what I know now of you who do n’t/selectively vax and attend church , do you allow your children to go into the nusery or Sunday school classes ?

Both boys (9 and 3) go to kids programs at church and I attend the church service , non-vaccinated mmmmm , I guess that , but my DS (now 2) contracted pertussis at 4 wks old , and while I have the confidence to deal with it again if I have to (i have a 4 mo DD) i ‘d just as soon not have to !

” even though ” she attended a gathering of some 500 church members that included many other unvaccinated children .

In NC we do n’t have philo so we use relig , we do n’t attend an organized religion but I will be damed if I am going to have my child injected with poison because the government says I should .

vaccin

I do know that Dr. Robert Mendelsohn testified on behalf of Orthodox Rabbis in Monsey , NY who had become ministers of the Universal Life Church to avoid vaxing their children and meet NY ‘s then necessary religious exemption for not being vaccinated .

I was even recently informed from a doctor that the next vaccine shots my son receives , is to make sure that my sister , (who has 3 autoimmune disorders) stays clear of him for at least 30 days. Â So , where does that put EVERYONE. Â If you receive vaccines , you must be careful and if you do n’t , you are also putting others at risk. Â Personally I do vaccinate my son , but on my own chosen schedule. Â I am the kind of person (that makes a lot of people mad) that believes that I should not tell others what religion they should practice , that they should have an abortion , what they should eat , how to raise their kids , or if vaccinating your child is a must. Â BUT , do not tell me what to do either , especially the for profit government , whose only concern is money and not the people. Â So , I guess I have to first say is : # 1. Â I am tired of the ” I am going to sue you ” attitude. Â Lawyers have become a house hold commodity and society has become a point your finger and blame others. Â I have learned some things for sure , that life is never fair , crap happens , and some times people die. Â # 2 Â I believe that vaccinating vs not vaccinating comes down to personal choice and therefore no matter what you choose you are responsible for your choice. Â Why , because either choice could hurt some one else , as true with all choices we make. Â And , after reading what I just wrote I realized that I still did not answer the question. Â I did n’t have time to read the responses but here ‘s my quick two cents on the issue. Â Â First of all this is completely ridiculous .

I was even recently informed from a doctor that the next vaccine shots my son receives , is to make sure that my sister , (who has 3 autoimmune disorders) stays clear of him for at least 30 days. Â So ,

where does that put EVERYONE. Â If you receive vaccines , you must be careful and if you do n't , you are also putting others at risk. Â Personally I do vaccinate my son , but on my own chosen schedule. Â I am the kind of person (that makes a lot of people mad) that believes that I should not tell others what religion they should practice , that they should have an abortion , what they should eat , how to raise their kids , or if vaccinating your child is a must. Â BUT , do not tell me what to do either , especially the for profit government , whose only concern is money and not the people. Â So , I guess I have to first say is : # 1. Â I am tired of the " I am going to sue you " attitude. Â Lawyers have become a house hold commodity and society has become a point your finger and blame others. Â I have learned some things for sure , that life is never fair , crap happens , and some times people die. Â # 2 Â I believe that vaccinating vs not vaccinating comes down to personal choice and therefore no matter what you choose you are responsible for your choice. Â Why , because either choice could hurt some one else , as true with all choices we make. Â And , after reading what I just wrote I realized that I still did not answer the question. Â I did n't have time to read the responses but here 's my quick two cents on the issue. Â Â First of all this is completely ridiculous .

I was even recently informed from a doctor that the next vaccine shots my son receives , is to make sure that my sister , (who has 3 autoimmune disorders) stays clear of him for at least 30 days. Â So , where does that put EVERYONE. Â If you receive vaccines , you must be careful and if you do n't , you are also putting others at risk. Â Personally I do vaccinate my son , but on my own chosen schedule. Â I am the kind of person (that makes a lot of people mad) that believes that I should not tell others what religion they should practice , that they should have an abortion , what they should eat , how to raise their kids , or if vaccinating your child is a must. Â BUT , do not tell me what to do either , especially the for profit government , whose only concern is money and not the people. Â So , I guess I have to first say is : # 1. Â I am tired of the " I am going to sue you " attitude. Â Lawyers have become a house hold commodity and society has become a point your finger and blame others. Â I have learned some things for sure , that life is never fair , crap happens , and some times people die. Â # 2 Â I believe that vaccinating vs not vaccinating comes down to personal choice and therefore no matter what you choose you are responsible for your choice. Â Why , because either choice could hurt some one else , as true with all choices we make. Â And , after reading what I just wrote I realized that I still did not answer the question. Â I did n't have time to read the responses but here 's my quick two cents on the issue. Â Â First of all this is completely ridiculous .

Â I 'm pretty sick of being told over and over and over again how absolutely positively TERRIBLE vaxes are. Â I did my research , I vax my ds. Â Â The body works fine on its own , I get it. Â I 'm not stupid. Â I give my ds tylenol when he has a fever , and alternate motrin and tylenol when the fever is persistent. Â NOT b/c I do n't have faith in his body to heal , but b/c I do n't want him to end up in the hospital again with febrile seizures (NO , they were NOT related to vaccines , it happened at least a month after his last set of vaccines) . Â It was the MOST terrifying experience of my ENTIRE life – I 'm pretty sure if that happened to your child you would give them fever-reducers too. Â Likewise , I 'm pretty sick of being told how TERRIBLE VPDs are and how if I do n't vaccinate my child , she will surely die .

Â SSM , If I remember correctly , there was a pretty strong consensus on that thread as well as any other thread I 've ever seen on the subject that you should keep your exposed children away from other children for awhile. Â There may be a few opposing views in each thread , but the idea that most people here that do n't vaccinate are just fine with exposing other children to diseases is not what I 've seen. Â Â While I do n't think that it 's legally enforceable , I do think that if someone knows that their child is ill or exposed to something serious and they willfully ignore the fact that

that there is a known immuno-compromised person where they will be , there should be some kind of liability. Â I go to church and there are a lot of older people there , some that I know are battling cancer , so I do try to take extra precautions when I ‘m there…washing my hands , not attending when sick , being where I wo n't have to hold hands with anyone .

) . Â B goes to a school where he has a whopping 8 people in his class. Â He ‘s also almost all of the way vaccinated in order to protect his sister. Â Before he has any contact with her , he washes his hands and during epidemics , he also changes his clothing before coming near her. Â Same goes for every activity. Â And yes , her music class is the first class of the day. Â It takes place in a church on a Thursday that has no children in the building from Sunday until Thursday. Â It ‘s a mom ; tot class where she sits on my lap the entire time and uses all of her own material. Â The odds of her catching something are much slimmer. Â I was told by her pediatrician to consider homeschooling her (which we are going to do) , to avoid daycares (I had to turn down an awesome job opportunity because of this) , and to avoid crowds during certain parts of the year in addition to giving her a whole foods diet with no processed foods and giving her higher doses of some vitamins than other children might take. Â He also said to avoid known Â contact with newly vaccinated Â children , and to avoid known contact with unvaccinated children during epidemics (right now in our area , it ‘s pertussis) . Â Since I ca n't keep her in a bubble , this is what I have to do to protect her since she has no ability , beyond what she gets from breastmilk , to protect herself from gastrointestinal and respiratory illnesses. Â Â In a school of 500 children , if pertussis goes around and all 10 unvaccinated children get it , they are contagious from the point of exposure through the point of symptoms–they ca n't wash that off of them. Â Yes , vaccinated people can transmit it too , but not in the same way unvaccinated children do , and the amount of time it ‘s transmittable is much shorter. Â The chances that my child comes into contact with one of the 10 children in the course of an 8 hour day (and over multiple days when the child is contagious but not yet showing symptoms) is much higher than the risk of catching something in a once weekly room with 2 other children that meets for 30 minutes (and does n't meet late November-February when most children are passing along illnesses) . Â That ‘s just simple math. Â Yeah , if one of the 2 kids in the room has a communicable disease , she ‘ll still catch it. Â But the risk of her being exposed in the two situations is vastly different. Â That ‘s why many children with immune deficiencies are homeschooled–because that takes the risk of exposure and reduces it a LOT .

) . Â B goes to a school where he has a whopping 8 people in his class. Â He ‘s also almost all of the way vaccinated in order to protect his sister. Â Before he has any contact with her , he washes his hands and during epidemics , he also changes his clothing before coming near her. Â Same goes for every activity. Â And yes , her music class is the first class of the day. Â It takes place in a church on a Thursday that has no children in the building from Sunday until Thursday. Â It ‘s a mom ; tot class where she sits on my lap the entire time and uses all of her own material. Â The odds of her catching something are much slimmer. Â I was told by her pediatrician to consider homeschooling her (which we are going to do) , to avoid daycares (I had to turn down an awesome job opportunity because of this) , and to avoid crowds during certain parts of the year in addition to giving her a whole foods diet with no processed foods and giving her higher doses of some vitamins than other children might take. Â He also said to avoid known Â contact with newly vaccinated Â children , and to avoid known contact with unvaccinated children during epidemics (right now in our area , it ‘s pertussis) . Â Since I ca n't keep her in a bubble , this is what I have to do to protect her since she has no ability , beyond what she gets from breastmilk , to protect herself from gastrointestinal and respiratory illnesses. Â Â In a school of 500 children , if pertussis goes around and all 10 unvaccinated children get it , they are contagious from the point of exposure through the point of

symptoms–they ca n't wash that off of them. Â Yes , vaccinated people can transmit it too , but not in the same way unvaccinated children do , and the amount of time it 's transmittable is much shorter. Â The chances that my child comes into contact with one of the 10 children in the course of an 8 hour day (and over multiple days when the child is contagious but not yet showing symptoms) is much higher than the risk of catching something in a once weekly room with 2 other children that meets for 30 minutes (and does n't meet late November-February when most children are passing along illnesses) . Â That 's just simple math. Â Yeah , if one of the 2 kids in the room has a communicable disease , she 'll still catch it. Â But the risk of her being exposed in the two situations is vastly different. Â That 's why many children with immune deficiencies are homeschooled–because that takes the risk of exposure and reduces it a LOT .

) . Â B goes to a school where he has a whopping 8 people in his class. Â He 's also almost all of the way vaccinated in order to protect his sister. Â Before he has any contact with her , he washes his hands and during epidemics , he also changes his clothing before coming near her. Â Same goes for every activity. Â And yes , her music class is the first class of the day. Â It takes place in a church on a Thursday that has no children in the building from Sunday until Thursday. Â It 's a mom ; tot class where she sits on my lap the entire time and uses all of her own material. Â The odds of her catching something are much slimmer. Â I was told by her pediatrician to consider homeschooling her (which we are going to do) , to avoid daycares (I had to turn down an awesome job opportunity because of this) , and to avoid crowds during certain parts of the year in addition to giving her a whole foods diet with no processed foods and giving her higher doses of some vitamins than other children might take. Â He also said to avoid known Â contact with newly vaccinated Â children , and to avoid known contact with unvaccinated children during epidemics (right now in our area , it 's pertussis) . Â Since I ca n't keep her in a bubble , this is what I have to do to protect her since she has no ability , beyond what she gets from breastmilk , to protect herself from gastrointestinal and respiratory illnesses. Â Â In a school of 500 children , if pertussis goes around and all 10 unvaccinated children get it , they are contagious from the point of exposure through the point of symptoms–they ca n't wash that off of them. Â Yes , vaccinated people can transmit it too , but not in the same way unvaccinated children do , and the amount of time it 's transmittable is much shorter. Â The chances that my child comes into contact with one of the 10 children in the course of an 8 hour day (and over multiple days when the child is contagious but not yet showing symptoms) is much higher than the risk of catching something in a once weekly room with 2 other children that meets for 30 minutes (and does n't meet late November-February when most children are passing along illnesses) . Â That 's just simple math. Â Yeah , if one of the 2 kids in the room has a communicable disease , she 'll still catch it. Â But the risk of her being exposed in the two situations is vastly different. Â That 's why many children with immune deficiencies are homeschooled–because that takes the risk of exposure and reduces it a LOT .

Religious belief in divine healing through faith not to excuse refusal to have children vaccinated .

Unless I spend the entire first year of my baby 's life refusing to leave her in any kind of group care situation (church nursery , etc) , she 's running the very real risk of being exposed to some recently vaccinated 2 , 4 , or 6 month old who is shedding rota in their poop without actually being sick enough to be home instead of in child care getting MY baby sick .

I researched just for a few days , quoted some state and federal laws regarding the exemption pertaining to our state , and i learned some things , like athesim is considered a religion ie athetist can file religious exemptions and that only one parent has to be 'religous ' to file the claim which is

good because dh is an atheist and I loosely follow unitarian beliefs… Â but i would n’t lie about being vaccinated for things that ds or dd is not , i do however forgo that ds has been vaccinated for the first 9 months as it would bring up a lot of questions of his partial vax status and i do n’t want to go into that.. Â Would I knowingly violate the law , in order to receive public school benefits ? Â Nope. Â There ‘s even less of an excuse if you have a religious exemption available to which you adhere. Â The schools are required to monitor the vaccination status of children under their care , and you ‘d be interfering with that , and for no good reason .

I researched just for a few days , quoted some state and federal laws regarding the exemption pertaining to our state , and i learned some things , like athesim is considered a religion ie athetist can file religious exemptions and that only one parent has to be ‘religous ‘ to file the claim which is good because dh is an atheist and I loosely follow unitarian beliefs… Â but i would n’t lie about being vaccinated for things that ds or dd is not , i do however forgo that ds has been vaccinated for the first 9 months as it would bring up a lot of questions of his partial vax status and i do n’t want to go into that.. Â Would I knowingly violate the law , in order to receive public school benefits ? Â Nope. Â There ‘s even less of an excuse if you have a religious exemption available to which you adhere. Â The schools are required to monitor the vaccination status of children under their care , and you ‘d be interfering with that , and for no good reason .

I agree , I would not tell about any vaccines and say that your child was never vaccinated since it is against your religion .

” vaccinated children get the best , high standard health care ” …or how about ” vaccinated children get the worst , number 1 death-inducing health care , according to Life Extension ‘s findings here Yeah the one thing I know is that this will never fly simply because it ignores the religion aspect of it .

” vaccinated children get the best , high standard health care ” …or how about ” vaccinated children get the worst , number 1 death-inducing health care , according to Life Extension ‘s findings here Yeah the one thing I know is that this will never fly simply because it ignores the religion aspect of it .

I do n’t even know what religion does n’t want their children vaccinated .

I cited religious beliefs , and he has specifically stated in the paperwork as follows : Â ” The Mother has failed to allow the child to be vaccinated and refuses to allow the father to administer to the child any medication including Tylenol citing religious beliefs , however , she does not attend church regularly .

If she does n’t have them , then ask yourself what was the point in having the vaccines , …. and…. how many vaccinated children who DO N’T have titres done , actually have immunity . ‘m wonding what religion it is that I ‘m going to need to ” convert ” to so that I can get my partially vaxed child into school .

Thanks Tell them that they are NOT to vaccinate your child , it ‘s against your religion .

object

Illinois Vaccine Awareness Coalition (IVAC) has this to say ; ” Day-Care Exemptions The Day-care religious vaccine exemption contains the language that children can exempt if the ” parents object thereto on the grounds that they conflict with the tenets and practices of a recognized church or religious organization , of which the parent is an adherent or member ” .

This Section specifically states that children may be exempt to immunization if the ” parents object thereto on the grounds that they conflict with the tenets and practices of a recognized church or religious organization , of which the parent is an adherent or member ” .

(2) Subsection (1) does not apply where a parent of the child objects in writing to the immunization on the ground that the immunization conflicts with the sincerely held convictions of the parent â s religion or conscience or a legally qualified medical practitioner gives medical reasons in writing to the operator as to why the child should not be immunized .

Source states : LCC communicants are specifically directed by their ecclesiastical supervisors to object to any of the following unholiness and abomination entering the Body Temples of themselves or their children , as a violation of the free exercise of their religious beliefs and the moral obligations of their religion .

Looks like religious would be the way to go – does n’t have to be a known religion , but the beliefs have to be outlined : ” Parents who object to health examinations or any part thereof , or to immunizations , on religious grounds shall not be required to submit their children or wards to the examinations or immunizations to which they so object if such parents or legal guardians present to the appropriate local school authority a signed statement of objection , detailing the grounds for the objection .

On the IL CPS website , I saw this… ” Any standards involving physical examinations , immunization , or medical treatment shall include appropriate exemptions : â ¢ for children whose parents object thereto on the grounds that they conflict with the tenets and practices of a recognized church or religious organization , of which the parent is an adherent or member… ” Can they adhere to a different standard or is the regular ” We do n’t vax due to conflicts with our religious practices ” enough ?

Ugh , adding , I have had to (on numerous occasions) remind doctors that I object to VACCINES not that I would decline necessary medical treatment for my child…there was a family at church that MIGHT have had TB and exposed my children (they ended up not having TB) but I called my dr to ask about what to do about poss TB exposure and the nurse asked about vaccines and when I said we do n’t vaccinate she made some noise about ” Well , even if it WAS TB you ‘d probably refuse treatment since you do n’t vaccinate .

We the parents of are submitting a religious exemption in lieu of vaccination documents as per Florida Statutes , Title XLVIII , Chapter 1003.22 which states The provisions of this section shall not apply if : (a) The parent of the child objects in writing that the administration of immunizing agents conflicts with his or her religious tenets or practices ; Under the Constitution of the United States we are granted ” freedom of religion .

Request to Withdraw from the Utah Statewide Immunization Information System Vermont School Immunization Exemption Form Virginia Certificate of Religious Exemption Washington state

Certificate of Exemption (COE) From School , Child Care , and Preschool Immunization Requirements West Virginia Only allows medical exemptions to vaccines If you want to work toward changing the law , here is a link to ” West Virginians for Vaccination Exemption ” Wisconsin (page 2) ” The immunization requirement is waived if the student , if an adult , or the student â s parent , guardian or legal custodian submits a written statement to the school , day care center or nursery school objecting to the immunization for reasons of health , religion or personal conviction .

I ‘m assuming preschools fall under ” daycare ” laws : Day-Care Exemptions The Day-care religious vaccine exemption contains the language that children can exempt if the ” parents object thereto on the grounds that they conflict with the tenets and practices of a recognized church or religious organization , of which the parent is an adherent or member ” .

Here is the specific language from the Illinois Vaccine Awareness Coalition : ” The Day-care religious vaccine exemption contains the language that children can exempt if the ” parents object thereto on the grounds that they conflict with the tenets and practices of a recognized church or religious organization , of which the parent is an adherent or member ” .

The regulations I found and excerpted above refer to the Child Care Act of 1969 , the link to which is : That Act specifically says that regulations must provide for exemptions : (h) Any standards involving physical examinations , immunization , or medical treatment shall include appropriate exemptions for children whose parents object thereto on the grounds that they conflict with the tenets and practices of a recognized church or religious organization , of which the parent is an adherent or member , and for children who should not be subjected to immunization for clinical reasons .

Section seven of the Child Care Act of 1969 (225 ILCS 10/7) states that children attending child care facilities may be exempt to immunization if the ” parents object thereto on the grounds that they conflict with the tenets and practices of a recognized church or religious organization , of which the parent is an adherent or member ” .

Here is the letter I used : To whome it may concern In accordance with FL Statutes , 232.032 (3a) which reads in part as follows : Immunizations against communicable diseases ; school attendance requirements ; exemptions.– 232.032 (a) ” The provision of this section shall not apply if : (a) the parent or guardian of the child objects in writing that the admistration of immunizing agents conflictswith his or her religious tenets or pratices ; ” Under the Constitution of the United States we are granted ” freedom of religion ” I know of no law that requires one to belong to an organized church or religious sect to practice his sacred beliefs .

1003.22 (5) (a) : â The provisions of this sections shall not apply if : (a) The parent or guardian of the child objects in writing that the administration of immunizing agents conflicts with his or her religious tenets or practices ; â Under the Constitution of the United States we are granted â freedom of religion. â I know of no law that requires one to belong to an organized church or religious sect to practice his sacred beliefs .

Freedom of religion is one of my rights , and I do sincerely object when and how these vaccines are insulted onto our children .

Looks like religious would be the way to go – does n’t have to be a known religion , but the beliefs

have to be outlined : ” Parents who object to health examinations or any part thereof , or to immunizations , on religious grounds shall not be required to submit their children or wards to the examinations or immunizations to which they so object if such parents or legal guardians present to the appropriate local school authority a signed statement of objection , detailing the grounds for the objection .

Ugh , adding , I have had to (on numerous occasions) remind doctors that I object to VACCINES not that I would decline necessary medical treatment for my child…there was a family at church that MIGHT have had TB and exposed my children (they ended up not having TB) but I called my dr to ask about what to do about poss TB exposure and the nurse asked about vaccines and when I said we do n’t vaccinate she made some noise about ” Well , even if it WAS TB you ‘d probably refuse treatment since you do n’t vaccinate .

Freedom of religion is one of my rights , and I do sincerely object when and how these vaccines are insulted onto our children .

requir

Even if the Church told all Catholic parents not to let their child get the MMR for instance , most parents would have to still be required to submit a religous exemption which would exempt all vaccines .

If the CDC wants parents to have faith in the vaccines their kids are receiving and laws require vaccines (even with exemptions available) , the data collection and analysis of possible reactions should be excellent .

I researched just for a few days , quoted some state and federal laws regarding the exemption pertaining to our state , and i learned some things , like athesim is considered a religion ie athetist can file religious exemptions and that only one parent has to be ‘religous ‘ to file the claim which is good because dh is an atheist and I loosely follow unitarian beliefs… Â but i would n’t lie about being vaccinated for things that ds or dd is not , i do however forgo that ds has been vaccinated for the first 9 months as it would bring up a lot of questions of his partial vax status and i do n’t want to go into that.. Â Would I knowingly violate the law , in order to receive public school benefits ? Â Nope. Â There ‘s even less of an excuse if you have a religious exemption available to which you adhere. Â The schools are required to monitor the vaccination status of children under their care , and you ‘d be interfering with that , and for no good reason .

Your rights in this situation , are to receive a waiver from ” required ” vaccinations for both yourself and your children , based on your personal religious beliefs , without any requirement about which denomination of church you belong to or to which denomination of church the chaplain you speak to belong .

He ‘s going to get MEO (military equal opportunity) involved if they hassle me more because they have no right to question my or my husband ‘s religious beliefs or require that we be part of a particular religion in order to make certain medical decisions for our son .

You are never required to leave your child unattended , and you are also not required to give any information regarding your religion .

You are never required to leave your child unattended , and you are also not required to give any information regarding your religion .

Looks like religious would be the way to go – does n't have to be a known religion , but the beliefs have to be outlined : " Parents who object to health examinations or any part thereof , or to immunizations , on religious grounds shall not be required to submit their children or wards to the examinations or immunizations to which they so object if such parents or legal guardians present to the appropriate local school authority a signed statement of objection , detailing the grounds for the objection .

Like Marnica said , you can certainly find religion any time. Â I do n't know if this is true for all states when it comes to religious exemptions but I know most states do n't require you to belong to an organized religion and religious exemptions may be based on personal religious beliefs. Â I certainly Â could n't imagine resorting to getting the children caught up for school entrance if that is not what you want to do. Â There are certainly ways around it. Â Â Just take a look at your state 's requirements for exemptions and go from there. Â As long as you follow the requirements , there should be no issue. Â If for some odd reason there is an issue , there is always the option of hiring a lawyer if need be , but like I said , I do n't think you 'd even have to worry about that. Â (I only always keep the option of a lawyer in my mind because I live in one of the more stricter states , but Â it sounds Â like your Â state may be much more lenient .

Check out the Kansas info on this site : khrisday- On the website you provided above it states that children can be exempt if : " Pupil is of a religion whose teachings are opposed to immunizations â the school reserves the right to require verification by the clergy of the religious tenets that preclude immunizations .

But a church or religious group , that requires you to be a member to send your kids there , CAN .

Federal courts have determined more than once that it is illegal for states to require membership in any specific religion in order for parents to exempt their children from vaccines .

Therefore , it would be illegal for the DoD to require your husband to be a member of a specific church or religious organization as a condition of accepting a waiver for his children , since he is a civilian .

On religious exemptions–what religion requires you to inject your child with substances you consider hazardous ?

We are converting to the flying spaghetti monster as a religion so that my son does not have to get ONE vaccine that was only required recently .

I was comforted to learn from the state department 's brochure on religious exemptions that I would not be required to document my " religion " , that any system of beliefs or ethics would count , and -also- that documenting my child 's vax would not disqualify her for the exemption .

If you file a religious exemption you have to get a member of the clergy to written statement or affidavit that " that such child 's parents or guardians are bona fide members of a denomination whose religious teaching requires reliance upon prayer or spiritual means alone for healing " For the

philisophical you do n’t , the parents just needs to write a written statement that vaccinating conflicts with their religious beliefs .

To me , to put my children into the hands of these people requires more than a leap of faith .

The other thing , as I stated in my exemption , is that the Catholic Church gives the PARENT complete rights over their child ‘s health and that we are BOUND to make the decisions for our children that WE feel are safest , therefore , if I thought some vaccines were a good thing , and some bad , my religion would require me as the parent to use some and to not use others .

Here is a link to Maine ‘s child protective services : Theoretically , by law , they ca n’t require it be a specific religion (that would of course be discriminating against people who have other beliefs) however I know that some states try .

conflict

38) RELIGIOUS EXEMPTION FORM Religious exemption requests must contain the wording found in the Alaska Administrative Code [4 AAC06.55 (b) (3) or 4 AAC 62.450 (c) (2)] stating all vaccines must be received unless the child â has an affidavit signed by his [4 AAC 62.450 (c) (2) says â the child â s â] parent or guardian affirming that immunization conflicts with the tenets and practices of the church or religious denomination of which the applicant [4 AAC 62.450 (c) (2) says â the parent or guardian â] is a member .

To be completed by the child â s parent or guardian : I/We affirm that immunization conflicts with the tenets and practices of the church or religious denomination of which the applicant is a member .

Illinois Vaccine Awareness Coalition (IVAC) has this to say ; ” Day-Care Exemptions The Day-care religious vaccine exemption contains the language that children can exempt if the ” parents object thereto on the grounds that they conflict with the tenets and practices of a recognized church or religious organization , of which the parent is an adherent or member ” .

This Section specifically states that children may be exempt to immunization if the ” parents object thereto on the grounds that they conflict with the tenets and practices of a recognized church or religious organization , of which the parent is an adherent or member ” .

(2) Subsection (1) does not apply where a parent of the child objects in writing to the immunization on the ground that the immunization conflicts with the sincerely held convictions of the parent â s religion or conscience or a legally qualified medical practitioner gives medical reasons in writing to the operator as to why the child should not be immunized .

On the IL CPS website , I saw this… ” Any standards involving physical examinations , immunization , or medical treatment shall include appropriate exemptions : â ¢ for children whose parents object thereto on the grounds that they conflict with the tenets and practices of a recognized church or religious organization , of which the parent is an adherent or member… ” Can they adhere to a different standard or is the regular ” We do n’t vax due to conflicts with our religious practices ” enough ?

here are what the regs from Georgia say : (A) There is appropriate evidence from the local health department or a physician that an immunization sequence has been started and can be completed within a period of up to 180 days , in which case a waiver of the immunization requirement for up to 180 days shall be granted ; (B) After examination by the local board of health or a physician , any preschool age child is found to have a physical disability which may make vaccination undesirable , in which case a certificate to that effect issued by the local board of health or the physician may be accepted in lieu of a certificate of immunization and shall exempt the child from obtaining a certificate of immunization until the disability is relieved ; (C) The parent or legal guardian furnishes an affidavit swearing or affirming that the immunization conflicts with the religious beliefs of the parent or legal guardian ; or (D) The implementation of such an immunization requirement violates any federal law or regulations or would result in the loss of any federal funds to this state ; and Also , remember in some states religion can mean " the church of jen " .

The AR law says this about religious : " The provisions of this section shall not apply if the parents or legal guardian of that child object thereto on the grounds that immunization conflicts with the religious tenets and practices of a recognized church or religious denomination of which OR PHILOSOPHICAL BELIEFS OF the parent or guardian is an adherent or member .

Oregon : Submit a statement signed by the parent that the child has not been immunized as required because the child is being reared as an adherent to a religion the teachings of which are opposed to such immunization Nebraska 's laws are interesting in that they require membership , but then give you an " out " : An affidavit stating that the immunization conflicts with the tenets and practice of a recognized religious denomination of which the student is an adherent or member or that immunization conflicts with the personal and sincerely followed religious beliefs of the student .

I 'm assuming preschools fall under " daycare " laws : Day-Care Exemptions The Day-care religious vaccine exemption contains the language that children can exempt if the " parents object thereto on the grounds that they conflict with the tenets and practices of a recognized church or religious organization , of which the parent is an adherent or member " .

The religious objection statement shall be considered valid if : 1) The parent or guardian of a child entering a child care facility objects to the immunization (s) on the grounds that they conflict with the tenets and practices of a recognized church or religious organization of which the parent is an adherent or member ; or 2) The objection by the parent or guardian of a child entering school (including programs under the kindergarten level) sets forth the specific religious belief which conflicts with the immunization (s) .

Here is the specific language from the Illinois Vaccine Awareness Coalition : " The Day-care religious vaccine exemption contains the language that children can exempt if the " parents object thereto on the grounds that they conflict with the tenets and practices of a recognized church or religious organization , of which the parent is an adherent or member " .

The regulations I found and excerpted above refer to the Child Care Act of 1969 , the link to which is : That Act specifically says that regulations must provide for exemptions : (h) Any standards involving physical examinations , immunization , or medical treatment shall include appropriate exemptions for children whose parents object thereto on the grounds that they conflict with the tenets and practices of a recognized church or religious organization , of which the parent is an

adherent or member , and for children who should not be subjected to immunization for clinical reasons .

Section seven of the Child Care Act of 1969 (225 ILCS 10/7) states that children attending child care facilities may be exempt to immunization if the " parents object thereto on the grounds that they conflict with the tenets and practices of a recognized church or religious organization , of which the parent is an adherent or member " .

Illinois Vaccine Awareness Coalition (IVAC) has this to say ; " Day-Care Exemptions The Day-care religious vaccine exemption contains the language that children can exempt if the " parents object thereto on the grounds that they conflict with the tenets and practices of a recognized church or religious organization , of which the parent is an adherent or member " .

This Section specifically states that children may be exempt to immunization if the " parents object thereto on the grounds that they conflict with the tenets and practices of a recognized church or religious organization , of which the parent is an adherent or member " .

On the IL CPS website , I saw this… " Any standards involving physical examinations , immunization , or medical treatment shall include appropriate exemptions : â ¢ for children whose parents object thereto on the grounds that they conflict with the tenets and practices of a recognized church or religious organization , of which the parent is an adherent or member… " Can they adhere to a different standard or is the regular " We do n't vax due to conflicts with our religious practices " enough ?

I 'm assuming preschools fall under " daycare " laws : Day-Care Exemptions The Day-care religious vaccine exemption contains the language that children can exempt if the " parents object thereto on the grounds that they conflict with the tenets and practices of a recognized church or religious organization , of which the parent is an adherent or member " .

The religious objection statement shall be considered valid if : 1) The parent or guardian of a child entering a child care facility objects to the immunization (s) on the grounds that they conflict with the tenets and practices of a recognized church or religious organization of which the parent is an adherent or member ; or 2) The objection by the parent or guardian of a child entering school (including programs under the kindergarten level) sets forth the specific religious belief which conflicts with the immunization (s) .

Here is the specific language from the Illinois Vaccine Awareness Coalition : " The Day-care religious vaccine exemption contains the language that children can exempt if the " parents object thereto on the grounds that they conflict with the tenets and practices of a recognized church or religious organization , of which the parent is an adherent or member " .

belong

Your rights in this situation , are to receive a waiver from " required " vaccinations for both yourself and your children , based on your personal religious beliefs , without any requirement about which denomination of church you belong to or to which denomination of church the chaplain you speak to belong .

Your rights in this situation , are to receive a waiver from ” required ” vaccinations for both yourself and your children , based on your personal religious beliefs , without any requirement about which denomination of church you belong to or to which denomination of church the chaplain you speak to belong .

It ‘s not like you teach your kids something and say ” this is because we belong to the Church of Jesus Christ… that we believe this ” and then tell them ” this is the rule in our family but not because we ‘re members of the Church .

Like Marnica said , you can certainly find religion any time. Â I do n't know if this is true for all states when it comes to religious exemptions but I know most states do n't require you to belong to an organized religion and religious exemptions may be based on personal religious beliefs. Â I certainly Â could n't imagine resorting to getting the children caught up for school entrance if that is not what you want to do. Â There are certainly ways around it. Â Â Just take a look at your state ‘s requirements for exemptions and go from there. Â As long as you follow the requirements , there should be no issue. Â If for some odd reason there is an issue , there is always the option of hiring a lawyer if need be , but like I said , I do n't think you ‘d even have to worry about that. Â (I only always keep the option of a lawyer in my mind because I live in one of the more stricter states , but Â it sounds Â like your Â state may be much more lenient .

I was especially scared though , because my kids attend a private church school at the church we belong to .

I do n't belong to a church or believe in a deity , but I do have one single religious belief , which is that vaccines should not be injected in my child ‘s body .

The CAtholic Church has always felt that the children belong to the parents , and as such , parents made decisions like this for their children , not the state or the church .

The CDC can not legally require you to belong to any specific religion in order to get a waiver from immunizations , since your children are not members of the military and therefore are guaranteed the right to religious freedom .

You do n't have to belong to a spefic religion or any at all to have spiritual/religous beliefs….and if you think that the risk outweighs the possible benefits then it ‘s easy to make that a religous reason….I think it ‘s against most mother ‘s ” Religions ” to inflict harm on their children .

I belong to a church that has stated it ‘s support for vaccination generally , however , my religion also places a strong emphasis on a personal relationship with God and personal revelation for one ‘s own life , and the lives of one ‘s children .

My understanding of this is that I either have to have a physician sign off on the vax for a specific medical reason pertaining to my child , or I must belong to a religion that does not condone vaxing .

(Not to upset anyone who actually belongs to an organised religion which preaches against vaccinations , but my child ‘s health and life are on the line and I will happily lie to protect her if need be .

You do n't have to belong to a particular religion or go to church or anything like that- it 's just based on your personal beliefs (such as being against injecting poison into your child) .

i religiously believe that it is wrong to inject children with neurotoxins and i certainly do n't have to belong to a church to defend that .

I look at it this way,the religion I belong to does not have an official stance on vaxing it is left up to the conscience of the parents to do what is right for their child .

Other Resources : State of Pennsylvania : State of Pennsylvania Immunization Program : health/cwp/ browse.asp ? A = 178 ; BMDRN = 2000 ; BCO B = 0 ; C = 38875 State of Pennsylvania Immunization Requirements for Child Care : health/cwp/view.asp ? A = 178 ; Q = 242063 State of Pennsylvania Immunization Requirements for School : health/cwp/view.asp ? a = 178 ; q = 242070 You do not have to belong to any church or religious organization and no one has the right to question your beliefs (except for NY I think) Here is PA vax exemption law : You do not have to be affiliated with a religion .

) Â Â You don â t have to answer this here , but it â s something to keep in mind. Â Do you profess to any faith or religion , organized or not ? Â If so , Florida doesn â t have any laws requiring you to belong to any established faith. Â Anybody of ANY faith can say that they have a spiritual duty to protect and make decisions for their children ! Â Â If you â re atheist or agnostic , are you ethically comfortable with lying to protect your kids ?

Â On the religion front , are you 100 % atheist/agnostic , or do you believe in the existence of a higher power ? Â If the latter is true , it 's hard to argue that any higher power or Creator would NOT want you to do what you deem best for the lives and health of your children , including doing your own vaccination schedule or not vaccinating at all. Â In that case , it 's sincerely felt. Â And it looks like MA law does n't require that you belong to an organized religion , nor does it require you to explain or justify your beliefs to the State. Â OK , on to the links !

Your rights in this situation , are to receive a waiver from " required " vaccinations for both yourself and your children , based on your personal religious beliefs , without any requirement about which denomination of church you belong to or to which denomination of church the chaplain you speak to belong .

Your rights in this situation , are to receive a waiver from " required " vaccinations for both yourself and your children , based on your personal religious beliefs , without any requirement about which denomination of church you belong to or to which denomination of church the chaplain you speak to belong .

unvax

Â Church and synagogue babysitting rooms in our area request that parents of unvaxed infants and toddlers keep their children home .

We 've never been (knocks on everything that even resembles wood in the house) , but if we ever do I 'll just say that the kids are unvaxed , and that it 's against our religion to hopefully avoid backlash .

If you have to go for religious either claim you changed your religion if you are bugged about it (but do n't say anything unless forced to explain yourself) or file it without saying a word about which vaxs the other kids have had and just file it like they are unvaxed .

In short , put your faith in the innate resistence of a well fed , well loved , unvaxed child , over the commercial products of money-hungry amoral Corporations .

The highly publicized shortage of available vaccine must make a lot of people think they should decide quickly . if you have to have a religious exempition to keep your kiddo unvaxed , then why not start the church of the….well thought out , or my body doesnt need heavy metals , or ……help me out my creative energy was just spent on the religion idea and I cant think of a name that will not offend and is welcoming to folks who question vaccines !

My unvaxed son goes to the daycare at our church services and I have never once worried about it .

So no , I would never let a kid of mine go to a church nursery , vaxed or unvaxed .

I havent attended church yet , but if I did I would want my kids involved…. For an hour during church , I personally do n't think twice about my unvaxed child being in a room with other kids , vaxed or otherwise . catid = 126 Because the current Prophylactic HPV vaccine is so safe and effective . thought the reason to justify the chicken pox vaccine was money , that it would save money by keeping parents at work instead of caring for a sick child .

At our former church , we had a family tell us that they would not allow their kids to play with our DS , because he was unvaxed .

Their reason is if there is an 'outbreak ' of some sort the parents of unvaxed children will be asked to keep them away from school : Christian Scientists are probably the most well-known established religion that is against vaccination .

I stay home with her , but she spends time in a church nursery (with a mix of vaxed/ unvaxed kids) and in childcare at our Mops group .

He recommends the HPV vaccine and called it a cervical cancer vaccine He went a little too far when he said that unvaxed children that go to the nursery at church are at the same risk level as daycare kids and should be vaccinated with dtap , meningitis and flu He recommended the chicken pox vaccine although he did say he was concerned with the rise in shingles He recommended the rotavirus vaccine â ¦.WTF ?

[*] He went a little too far when he said that unvaxed children that go to the nursery at church are at the same risk level as daycare kids and should be vaccinated with dtap , meningitis and flu Sorry…but I have to agree with him on this one and I 'm not at all a Dr. Sears fan .

I have three unvaxed kids and in my state I have to claim it is against my religion or else CPS would take my kids away .

On top of the 5 in 1 , they offer prevnar and meng c at 2 , 4 and 6 months : sad I feel so lucky to have a friend who brought up vax risks and does n't vax her dd- it 's something most people do n't even

know is an option- they think you have to vax Yes , we have confidence in our plumbers , but we have FAITH in our doctors ; the relationship between a doctor and a patient is a sacred relationship…it is a religious experience ! currently have a religious exemption with the school district so my daughter can attend public school unvaxed .

But if they are allowing adults of unknown vax status and germy vaxed children to see the baby , plus going to daycare/ Church nursery/ Gymboree/ shopping at Walmart , then they are clearly excluding the one unvaxed child that they know about .

I know a myriad of other unvaxed kids , through friends and church .

OP – Yes I have worried about those things , especially when there were cases of measles the next town over (my children are 100 % unvaxxed) but I have more faith in their ability to fight a natural disease than I do in their ability to fight against toxins that I would be allowing to be injected into their bodies .

Â Since it was brought up–my fully vaccinated children are vibrantly healthy and thriving. Â No destroyed immune systems here. Â Nor frankly , in anybody I know personally. Â I do have one friend who 's child had a reaction to an initial vaccine , and whose children (both vaxed and Â unvaxed) Â are prone to allergies , and I think she is wise in declining vaccines based on that. Â (And the information provided by both the government and vaccine manufacturers would support that , as they do not claim it is best for * all * , and have specific listings of people for whom the balance falls against vaccinating) I honestly never really thought much about it in terms of my faith , honestly. Â I think some others make a good point that God did a pretty good job designing the immune system , and I think we should trust it to work as planned , but on the other hand , if I have a really bad headache I 'll take a Tylenol. Â So , our decision was based solely on medical and environmental factors . know this sounds like a strange question .

My unvaxed child is in daycare , church nurseries , Sunday School , etc .

exempt

Even if the Church told all Catholic parents not to let their child get the MMR for instance , most parents would have to still be required to submit a religous exemption which would exempt all vaccines .

Illinois Vaccine Awareness Coalition (IVAC) has this to say ; " Day-Care Exemptions The Day-care religious vaccine exemption contains the language that children can exempt if the " parents object thereto on the grounds that they conflict with the tenets and practices of a recognized church or religious organization , of which the parent is an adherent or member " .

This Section specifically states that children may be exempt to immunization if the " parents object thereto on the grounds that they conflict with the tenets and practices of a recognized church or religious organization , of which the parent is an adherent or member " .

Michigan does allow you to exempt your child from certain vaccines based on religion : A parent or legal guardian wishing to exempt his or her child from a particular vaccination must provide a written statement indicating the religious or philosophical objections to the vaccination (s) .

Michigan does allow you to exempt your child from certain vaccines based on religion : A parent or legal guardian wishing to exempt his or her child from a particular vaccination must provide a written statement indicating the religious or philosophical objections to the vaccination (s) .

Check out the Kansas info on this site : khrisday- On the website you provided above it states that children can be exempt if : ” Pupil is of a religion whose teachings are opposed to immunizations â the school reserves the right to require verification by the clergy of the religious tenets that preclude immunizations .

Federal courts have determined more than once that it is illegal for states to require membership in any specific religion in order for parents to exempt their children from vaccines .

here are what the regs from Georgia say : (A) There is appropriate evidence from the local health department or a physician that an immunization sequence has been started and can be completed within a period of up to 180 days , in which case a waiver of the immunization requirement for up to 180 days shall be granted ; (B) After examination by the local board of health or a physician , any preschool age child is found to have a physical disability which may make vaccination undesirable , in which case a certificate to that effect issued by the local board of health or the physician may be accepted in lieu of a certificate of immunization and shall exempt the child from obtaining a certificate of immunization until the disability is relieved ; (C) The parent or legal guardian furnishes an affidavit swearing or affirming that the immunization conflicts with the religious beliefs of the parent or legal guardian ; or (D) The implementation of such an immunization requirement violates any federal law or regulations or would result in the loss of any federal funds to this state ; and Also , remember in some states religion can mean ” the church of jen ” .

In NYC , there is a requirement and you can use religion to exempt a child .

Personally I think that this is a good thing because it gives those of us who are not religious a way to exempt our kids and we do n’t have to ” disclose ” a religion or a set of prescribed beliefs in order to get it .

I ‘m assuming preschools fall under ” daycare ” laws : Day-Care Exemptions The Day-care religious vaccine exemption contains the language that children can exempt if the ” parents object thereto on the grounds that they conflict with the tenets and practices of a recognized church or religious organization , of which the parent is an adherent or member ” .

Â Long story short , my 3 year old is vax free. Â My older child had some medical issues that may or may not have been caused by vax side effects , so I have put off vaccinating the 3 year old. Â The more I research , the more I dont want to vax at all. Â Ok , the dilema part… we live in a state (NC) that doesnt allow philosophical exemptions. Â Even with my strong feelings about not vaccinating any more , I am having a very hard time with the idea of having to lie so my kids can go to public school. Â And my DH is completely against it (lying on the form) . Â Im Methodist.. it is not against my religion to vaccinate. Â However , it is against my religion to lie. Â Â I HATE that I ‘m in the position of having to either sacrifice their health or their education. Â Does anyone have any advice on how to deal with this ? Â Â Thanks in advance , I ‘m really struggling with this , Â Â Â Â ” If the bona fide religious beliefs of an adult or the parent , guardian or person in loco parentis of a child are contrary to the immunization requirements contained in this Part , the adult or the child shall be exempt from the requirements .

Here is the specific language from the Illinois Vaccine Awareness Coalition : " The Day-care religious vaccine exemption contains the language that children can exempt if the " parents object thereto on the grounds that they conflict with the tenets and practices of a recognized church or religious organization , of which the parent is an adherent or member " .

Section seven of the Child Care Act of 1969 (225 ILCS 10/7) states that children attending child care facilities may be exempt to immunization if the " parents object thereto on the grounds that they conflict with the tenets and practices of a recognized church or religious organization , of which the parent is an adherent or member " .

Michigan does allow you to exempt your child from certain vaccines based on religion : A parent or legal guardian wishing to exempt his or her child from a particular vaccination must provide a written statement indicating the religious or philosophical objections to the vaccination (s) .

Michigan does allow you to exempt your child from certain vaccines based on religion : A parent or legal guardian wishing to exempt his or her child from a particular vaccination must provide a written statement indicating the religious or philosophical objections to the vaccination (s) .

Federal courts have determined more than once that it is illegal for states to require membership in any specific religion in order for parents to exempt their children from vaccines .

Personally I think that this is a good thing because it gives those of us who are not religious a way to exempt our kids and we do n't have to " disclose " a religion or a set of prescribed beliefs in order to get it .

immun

(2) Subsection (1) does not apply where a parent of the child objects in writing to the immunization on the ground that the immunization conflicts with the sincerely held convictions of the parent â s religion or conscience or a legally qualified medical practitioner gives medical reasons in writing to the operator as to why the child should not be immunized .

explaining doctrine/beliefs that prohibit immunization (Note : Parents/guardians need not necessarily be a member of an organized religion or religious institution to obtain a religious exemption) ; â ¢ Other writings or sources upon which the parent/guardian relied in formulating religious beliefs that prohibit immunization ; â ¢ A copy of any parental/guardian statements to healthcare providers or school district officials in a district of prior residence explaining the religious basis for refusing immunization ; â ¢ Any documents or other information the parent/guardian may be willing to provide that reflect a sincerely held religious objection to immunization (for example : disclosure of whether parent/guardian or other children have been immunized , parent/guardian â s current position on allowing himself or herself or his or her children to receive or refuse other kinds of medical treatment .

Does her husband get some sort of reward or points if he gets the kids in his church immunized ?

Furthermore the Oregon Revised Statutes under Disease and Condition Control 433.267 ; Immunization of school children ; exceptions ; effect of failure to comply , (c) says that a statement signed by the parent that the child has not been immunized because the child is being reared as an

adherent to a religion the teachings of which are opposed to such immunization is exempt from such immunization .

An attorney for the district would jump right on the parent and ask why they would circ their child if they felt that God created us perfectly and that immunizing would go against faith in God and our perfect immune system , bodies , etc .

I called the ER at the local medical facility here , and I asked if as a matter of policy parents of children who are not immunized as a matter of faith , are turned over to CPS .

In the event that the caretaker relative indicates that immunizations are against his or her religion , the technician shall request that the caretaker indicate in a signed , written statement that s/he is a practicing specified religion and that it is against the religious doctrine to immunize a child .

No law shall in any case whatever control the free exercise , and enjoyment of religious (sic) opinions , or interfere with the rights of conscience. â   Furthermore the Oregon Revised Statutes under Disease and Condition Control 433.267 ; Immunization of school children ; exceptions ; effect of failure to comply , (c) says that a statement signed by the parent that the child has not been immunized because the child is being reared as an adherent to a religion the teachings of which are opposed to such immunization is exempt from such immunization .

Oregon : Submit a statement signed by the parent that the child has not been immunized as required because the child is being reared as an adherent to a religion the teachings of which are opposed to such immunization Nebraska 's laws are interesting in that they require membership , but then give you an " out " : An affidavit stating that the immunization conflicts with the tenets and practice of a recognized religious denomination of which the student is an adherent or member or that immunization conflicts with the personal and sincerely followed religious beliefs of the student .

I usually just say " we do n't immunize " plain and simple like that .It usually stops most people (all but the nosiest) in their tracks .The other daya at church I had a pleasant surprise though.One woman asked how our baby Catherine was doing with her shots and I gave my standard reply and she got so excited because she had been looking for more info on not vaxing their daughter but didnt know who to ask or where to go .She is totally not the type you would picture not vaxing so it was nice to be able to help her out .

All the vaccine package inserts can be found here . We are moving to Oregon and i am a little unsure about the law… it says… " A statement signed by the parent that the child has not been immunized as described in paragraph (a) of this subsection because the child is being reared as an adherent to a religion the teachings of which are opposed to such immunization " THis does mean that I do NOT have to get any clergy person to sign saying that we are part of a religious group that does not agree with vaccinations ?

even if you used the religous exemp you do n't have to talk about your religon or your beleifs ever. , and if its a public school it has to allow the exemptions . son went to school this yr after being homeschooled. Â The school gave me all sorts of trouble. Â He is not vaccinated past 1 yr old. Â The nurse called yesterday looking for his record. Â I was reading some of the schools have there own paper others dont. Â They want the name of your religion if you claim a religious conviction and

good cause reason if you claim that. Â I am not sure at this point which is best to claim. Â I am holding them off till Monday if you can offer any help I would appreciate it Â Which is best Â thank you Â Amy You ‘re in luck. Â Ohio has a philosophical exemption. Â Â Â Here ‘s the specific laws : Â Â Â Â It sounds like you just have to write a letter stating you are opposed to vaccines for personal/philosophical/religious reasons and that should suffice. Â Hopefully someone from Ohio can give you more information. Â Â Good luck. Â Â Ohio law : ” A pupil who presents a written statement of the pupil â s parent or guardian in which the parent or guardian declines to have the pupil immunized for reasons of conscience , including religious convictions , is not required to be immunized .

even if you used the religous exemp you do n’t have to talk about your religon or your beleifs ever. , and if its a public school it has to allow the exemptions . son went to school this yr after being homeschooled. Â The school gave me all sorts of trouble. Â He is not vaccinated past 1 yr old. Â The nurse called yesterday looking for his record. Â I was reading some of the schools have there own paper others dont. Â They want the name of your religion if you claim a religious conviction and good cause reason if you claim that. Â I am not sure at this point which is best to claim. Â I am holding them off till Monday if you can offer any help I would appreciate it Â Which is best Â thank you Â Amy You ‘re in luck. Â Ohio has a philosophical exemption. Â Â Â Here ‘s the specific laws : Â Â Â Â It sounds like you just have to write a letter stating you are opposed to vaccines for personal/philosophical/religious reasons and that should suffice. Â Hopefully someone from Ohio can give you more information. Â Â Good luck. Â Â Ohio law : ” A pupil who presents a written statement of the pupil â s parent or guardian in which the parent or guardian declines to have the pupil immunized for reasons of conscience , including religious convictions , is not required to be immunized .

Here is the letter I used : To whome it may concern In accordance with FL Statutes , 232.032 (3a) which reads in part as follows : Immunizations against communicable diseases ; school attendance requirements ; exemptions.– 232.032 (a) ” The provision of this section shall not apply if : (a) the parent or guardian of the child objects in writing that the admistration of immunizing agents conflictswith his or her religious tenets or pratices ; ” Under the Constitution of the United States we are granted ” freedom of religion ” I know of no law that requires one to belong to an organized church or religious sect to practice his sacred beliefs .

People who had small children who had not been immunized were asked to not come to church .

After the outbreak , many families decided to immunize their children , while other families remained unconvinced about the low risk of autism , Broersma said , noting it was not church policy to withhold immunizations .

An attorney for the district would jump right on the parent and ask why they would circ their child if they felt that God created us perfectly and that immunizing would go against faith in God and our perfect immune system , bodies , etc .

I usually just say ” we do n’t immunize ” plain and simple like that .It usually stops most people (all but the nosiest) in their tracks .The other daya at church I had a pleasant surprise though.One woman asked how our baby Catherine was doing with her shots and I gave my standard reply and she got so excited because she had been looking for more info on not vaxing their daughter but didnt know who to ask or where to go .She is totally not the type you would picture not vaxing so it

was nice to be able to help her out .

After the outbreak , many families decided to immunize their children , while other families remained unconvinced about the low risk of autism , Broersma said , noting it was not church policy to withhold immunizations .

allow

Â Reply Reply But why keep a child out of school because there ‘s possibly unvaccinated kids who could infect your child with something but still allow them to go to extracurricular activities (karate , gymnastics , story time at library , church nursery/school , etc) ?

My hubby and I are presently concerned about the ingredients – our faith prevents us from allowing certain substances into our son ‘s body .

I cited religious beliefs , and he has specifically stated in the paperwork as follows : Â ” The Mother has failed to allow the child to be vaccinated and refuses to allow the father to administer to the child any medication including Tylenol citing religious beliefs , however , she does not attend church regularly .

I cited religious beliefs , and he has specifically stated in the paperwork as follows : Â ” The Mother has failed to allow the child to be vaccinated and refuses to allow the father to administer to the child any medication including Tylenol citing religious beliefs , however , she does not attend church regularly .

You do n’t have to be a part of an organized religion that does n’t allow vax- you can just say that vax ” are against your personal religious beliefs ” – that ‘s what I did- harming my child is against my personal religious beliefs Jenni , I can almost bet that medical wo n’t be used .

I assert that in my religion it is unacceptable to allow my child to be injected with toxins (aluminum , formaldehyde , mercury) , tissues derived from human abortions (MMR , varivax) , or animal tissues (all the other vaccines are grown in cells from monkeys , cows , or chicks) .

At our former church , we had a family tell us that they would not allow their kids to play with our DS , because he was unvaxed .

It also makes no sense to me that she would allow your children around your kids at church or other places , but it ‘s suddenly a bad idea when it comes down to baby-sitting .

I feel we should put all of our faith in God , and not risk giving our children vaccines only for preventative measures – but I also have the thought that God allowed us to invent these vaccines , and they have helped bring down the numbers of these illnesses – so…I ‘m at a loss .

It wasn â t until the bashing started (where people were calling parents who don â t vax child abusers , and saying that un-vaxed children should not be allowed in schools , parks , churches etc .

I live in MA where the only allowed exemption is for religious reasons and I feel violated that info about my son ‘s vax status and therefore , religion , left the nurse ‘s office .

The director freaked and called the church complaining that I was allowed to work there with unvax ‘d kids .

But if they are allowing adults of unknown vax status and germy vaxed children to see the baby , plus going to daycare/ Church nursery/ Gymboree/ shopping at Walmart , then they are clearly excluding the one unvaxed child that they know about .

OP – Yes I have worried about those things , especially when there were cases of measles the next town over (my children are 100 % unvaxxed) but I have more faith in their ability to fight a natural disease than I do in their ability to fight against toxins that I would be allowing to be injected into their bodies .

Request to Withdraw from the Utah Statewide Immunization Information System Vermont School Immunization Exemption Form Virginia Certificate of Religious Exemption Washington state Certificate of Exemption (COE) From School , Child Care , and Preschool Immunization Requirements West Virginia Only allows medical exemptions to vaccines If you want to work toward changing the law , here is a link to ” West Virginians for Vaccination Exemption ” Wisconsin (page 2) ” The immunization requirement is waived if the student , if an adult , or the student â s parent , guardian or legal custodian submits a written statement to the school , day care center or nursery school objecting to the immunization for reasons of health , religion or personal conviction .

But , if they accepted your child ‘s application for enrollment , only to find out the week before school starts that your faith does not allow your child to be vaccinated , and they kicked them out of the school , would n’t that be considered denial of admittance due to religion ?

There â s a leap of faith that has to take place when a parent allows his or her child at the age of two months to get an injection .

I ‘m pumping up for our visit next week- I ‘ve decided to say that my religion does not allow causing harm to children ; it ‘s not God ‘s will- wjen asked what religion I think you handled it well- it ‘s personal .

Saturday , June 5 , 2004 1 : 00 – 5 : 00 p.m. Providence Road Church of Christ 4900 Providence Road , Charlotte , NC No childcare provided A room will be provided for nursing mothers ; infants (no older children) , Only adults allowed in auditorium (DVD will be made so that anyone not able to obtain childcare may purchase a DVD) Tickets $ 5 per person , available at the door Keynote Speakers : Barbara Loe Fisher ; Kathi Williams Also speaking : Arthur Evangelista , former FDA Investigator John Hanna , DC For more information contact Lisa Jillani , director PAVE (People Advocating Vaccine Education) P.O .

I have some resources for claiming religious exemption , but I also live in a state where there have been recent high profile cases of families who are members of a particular church being charged with child abuse/neglect/death of a child because the church tenets do n’t allow for medical intervention .

have

Thank you , thank you , thank you for starting this thread ! Â Â We ‘ve never vax ‘d any of our kids –

due to a strong family history of autoimmune diseases – but over the past winter our youngest developed asthma out of the blue ; he ‘s been in ; out of the hospital 4 times since December. Â We finally have that under control , but our FP ; I had a few long talks about it and we ‘ve decided to very slowly start vaxing. Â Â I am terrified. Â Our oldest ds has a speech delay and sensory issues , our dd has juvenile RA and I have RA as well and I ‘m just so scared that these will damage their immune systems [and mine] down the line. Â I feel so torn because the youngest is only 15 months old now – he ‘s so helpless in all of this – and we ‘re doing it for his benefit mostly ; in case we catch anything , hopefully the vaxes will at least make the disease less severe , and thus less likely to land him in the hospital. Â But how do I reconcile that with the damage this might do to my older 2 ? Â I would never forgive myself if something happened to them. Â Â I ‘m down with the idea that as our lifestyles change we change along with them , but was it easy or hard for those of you going from non-vax to vax ? Â I ‘m having such a moral dilemma about this. Â On the one hand , it ‘s a no-brainer , on the other if anything happens the fault is 100 % mine for giving the ok. Â I guess that ‘s the leap of faith we ‘re taking. Â So thanks for listening , sorry to highjack , and thank you OP for starting this very timely thread !

Thank you , thank you , thank you for starting this thread ! Â Â We ‘ve never vax ‘d any of our kids – due to a strong family history of autoimmune diseases – but over the past winter our youngest developed asthma out of the blue ; he ‘s been in ; out of the hospital 4 times since December. Â We finally have that under control , but our FP ; I had a few long talks about it and we ‘ve decided to very slowly start vaxing. Â Â I am terrified. Â Our oldest ds has a speech delay and sensory issues , our dd has juvenile RA and I have RA as well and I ‘m just so scared that these will damage their immune systems [and mine] down the line. Â I feel so torn because the youngest is only 15 months old now – he ‘s so helpless in all of this – and we ‘re doing it for his benefit mostly ; in case we catch anything , hopefully the vaxes will at least make the disease less severe , and thus less likely to land him in the hospital. Â But how do I reconcile that with the damage this might do to my older 2 ? Â I would never forgive myself if something happened to them. Â Â I ‘m down with the idea that as our lifestyles change we change along with them , but was it easy or hard for those of you going from non-vax to vax ? Â I ‘m having such a moral dilemma about this. Â On the one hand , it ‘s a no-brainer , on the other if anything happens the fault is 100 % mine for giving the ok. Â I guess that ‘s the leap of faith we ‘re taking. Â So thanks for listening , sorry to highjack , and thank you OP for starting this very timely thread !

Thank you , thank you , thank you for starting this thread ! Â Â We ‘ve never vax ‘d any of our kids – due to a strong family history of autoimmune diseases – but over the past winter our youngest developed asthma out of the blue ; he ‘s been in ; out of the hospital 4 times since December. Â We finally have that under control , but our FP ; I had a few long talks about it and we ‘ve decided to very slowly start vaxing. Â Â I am terrified. Â Our oldest ds has a speech delay and sensory issues , our dd has juvenile RA and I have RA as well and I ‘m just so scared that these will damage their immune systems [and mine] down the line. Â I feel so torn because the youngest is only 15 months old now – he ‘s so helpless in all of this – and we ‘re doing it for his benefit mostly ; in case we catch anything , hopefully the vaxes will at least make the disease less severe , and thus less likely to land him in the hospital. Â But how do I reconcile that with the damage this might do to my older 2 ? Â I would never forgive myself if something happened to them. Â Â I ‘m down with the idea that as our lifestyles change we change along with them , but was it easy or hard for those of you going from non-vax to vax ? Â I ‘m having such a moral dilemma about this. Â On the one hand , it ‘s a no-brainer , on the other if anything happens the fault is 100 % mine for giving the ok. Â I guess that ‘s the leap of faith we ‘re taking. Â So thanks for listening , sorry to highjack , and thank you OP for

starting this very timely thread !

CREATE A FREE WEBSITE OR BLOG AT W

Follow •••

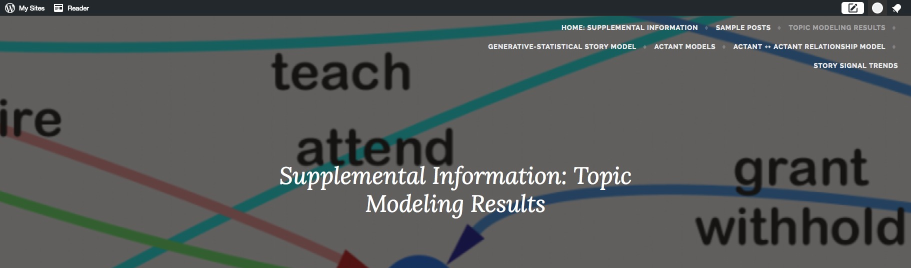

Supplemental Information: Topic Modeling Results

Topics on mothering.com

We apply two topic modeling techniques: Latent Dirichlet Allocation (LDA) and Contextual Random Walk Trap (CRWT). We use all the posts comprising a thread as a single document. Tables 5 through 8 list forty topics obtained by applying CRWT to the mothering.com posts. As explained in the main paper, CRWT creates a hierarchy of topics. Figure 1 presents the hierarchy related to the topics strongly connected to Exemption. Instead of the entire hierarchy, we present here a flattened list of 40 leaf topics, and for each one we list some of the more general words, which are related words higher in the hierarchy. We have avoided putting very general words such as,"Vaccine, Vax" etc. since they will appear in almost all the forum's topics. With CRWT, the number of topics is not specified as an input parameter as it is in LDA. Instead, it is determined as part of the output of the algorithm. We have listed only 40 of the leaf topics here; there are at least 100 more topics of varying sizes that we have left out. The granularity and details of the topics extracted by CRWT are quite revealing, and we encourage readers to explore the words in the individual topics which indicate the different facets that are of interest to forum posters. Here we point out a few of the interesting themes:

(i) Exemption Related: Topics 1, 2, 6, 8, 11, and 33

HOME: SUPPLEMENTAL INFORMATION · SAMPLE POSTS · TOPIC MODELING RESULTS · GENERATIVE-STATISTICAL STORY MODEL · ACTANT MODELS · ACTANT ↔ ACTANT RELATIONSHIP MODEL · STORY SIGNAL TRENDS

(ii) Product Safety and Concerns about Exposure to Ingredients in Vaccine: Topics 3, 10, 19, 31, 32, 35 ; Topic 35 for example focuses on parental concerns regarding vitamin injections given to newborns

(iii) Natural Remedies and supplements: Topics 10, and 12 ; Topic 12 is on Homeopathic inoculation or Nosodes, and Topic 10 deals with the natural immunity boosting benefits of breast milk

(iv) Sharing Information about "non-vax" Friendly Doctors: Topic 34 ;

(v) Researching Adverse Effects of Vaccines: Topic 38

(vi) Studies Linking MMR vaccines to Autism: Topic 13, which focuses on the discredited study by Dr. Wakefield, originally published in the British journal Lancet and since retracted;

(vii) Historical Public Health Programs to eliminate Smallpox: Topic 29.

The breadth and depth of the topics clearly point to a community of parents that are highly engaged, detail oriented, and who process multiple sources of information, including highly technical resources.

Tables 9 and 10 list the topics obtained by the LDA method, when the parameter for the number of topics, K, is set to 20. The topics obtained by LDA tend to include more general words, especially for K = 20. When K is increased, more granular topics often emerge. Since the focus of the algorithm is to explain the underlying documents using the extracted topics, more frequent words always appear as top words in each topic. The LDA-estimated topics, as listed in Tables 9 and 10, provide a clear high level view into the different concerns and ideas expressed by parents in the forum. As noted in the main paper, the topic of Exemption appears in Topics 10 and 14.

CRWT Topics:

Table 5. Topics 1–10 from mothering.com using CRWT.

	Topic Words
**Topic 1**	*sign petition signed draft signing waiver form refusal abc bump background holding accountable accuracy activist obtaining bumped arkansas aap on-line introducing exemptions approaching triple efficient* **General Words:** form sign exemptions signed declined aap
**Topic 2**	*illinois il chicago religious homefirst detailing religion eisenstein harrassed filing philisophical exemption interpretation passage spiritual sworn philosophical calmly non-issue jennifer searched closest delivered arises ren* **General Words:** exemption religious religion philosophical exception interpretation
**Topic 3**	*mercury ethylmercury methylmercury thimerosal preservative ethyl tuna vial vials mcg multi-dose trace fluzone excreted fish hg thimerosal-free epa formulations absorbed thimerasol monkeys bloodstream replaced mercury-free* **General Words:** free exposure mercury level injected directly
**Topic 4**	*tetanus wound spores nail wounds peroxide rusty bled oxygen scrapes rust tetnus puncture tig hydrogen manure soak tet splinter anaerobic stepped nails splinters scrape bleed* **General Words:** tetanus adult conditions cut mouth light
**Topic 5**	*ascorbate acid calcium vitamin sa kefir magnesium minerals iron yogurt ascorbic mt grams grains eat tablets supplement selenium deficiency kg mag coconut taste hylands wheat* **General Words:** food eat diet vitamin sleep levels
**Topic 6**	*god catholic church catholics vatican pa priest christian jesus temple holy doctrine bible teachings christians biblical moral spirit conviction religous pennsylvania churches ethical conscience pastor* **General Words:** sad god bodies belief relationship faith
**Topic 7**	*bill senate senator committee bills governor nj congress legislators assembly jersey fax president ban provisions york bush congressman ny coalition rally elected e-mail representative legislation* **General Words:** state choice house comment attention act
**Topic 8**	*notarized mn notary minnesota georgia forms dated ga bank conscientious nervous statement lieu freaking someplace core till rock send nope yup* **General Words:** statement send forms nervous till nope
**Topic 9**	*malaria traveling travelling yellow costa rica mexico typhoid plane mosquito abroad travel europe africa airplane passport eu trip travelled airport flying austria france india switzerland* **General Words:** stopped country leave researching afraid countries
**Topic 10**	*neomycin phosphate bovine mg micrograms medium adsorbed albumin polysorbate sucrose hemagglutinin hydroxide sulfate formaldehyde residual glutamate lf serum 2-phenoxyethanol yeast chloride potassium hbsag purified protein* **General Words:** virus aluminum harmful hepatitis strain diphtheria

Table 6. Topics 11–20 from mothering.com using CRWT.

	Topic Words
**Topic 11**	*private schools pupil enrollment preschools facility ohio michigan code daycare deny childcare nursery admission pre-school funding law governing california accordance daycares texas school handbook unimmunized* **General Words:** school public opinion personal run law
**Topic 12**	*breast-fed iga pmid indexed medline intestinal dopt pubmed abstract maternal milk breastfeeding publication breast-feeding offspring cytokines pediatr infants zinc tract agents infant human protection binding* **General Words:** human infant infants protection breastfeeding factors
**Topic 13**	*wakefield gmc deer lancet leary study andrew retraction gi mv hypothesis controls rna lab psychological hannah gr8blessings authors britain debunked condition testimony behavioral comprehensive richard* **General Words:** mmr study condition arguments intended lab
**Topic 14**	*bird pandemic avian flu h5n1 birds swine h1n1 seasonal poultry bird-flu sars mutations mutate pandemics chickens mutating mutation influenza squalene virulence announcement tamiflu mutated species* **General Words:** shot flu power mandatory influenza pointed
**Topic 15**	*autism disorder autistic asd speech therapy cognitive auditory processing xoxo diagnosis beth abilities criteria label spectrum processes difficulties tests communication listening language aspergers therapies biomedical* **General Words:** autism rates tests disorders diagnosis autistic
**Topic 16**	*rabies dog cat cats vet dogs pets parvo distemper puppy feline pet puppies indoor vets kitten kitty animals heartworm rabid bitten bite animal outdoor bat* **General Words:** animal animals touch dog annual bite
**Topic 17**	*shingles cp zoster pox herpes chicken build wild blisters chickenpox wild-type sm secondary healed dried survival deals contagious generations stressful naturally* **General Words:** chicken pox naturally chickenpox cp wild
**Topic 18**	*meningitis serotype pneumoniae hib replacement serotypes pneumococcal meningococcal meningitidis influenzae neisseria conjugate college invasive prevnar colleges polysaccharide haemophilus streptococcus students bacterial bacteria menactra non-vaccine rabbits* **General Words:** risk hib bacteria booster boy meningitis
**Topic 19**	*fetal aborted cell cells gsk package diploid insert twinrix aventis wi-38 tissue pasteur merck description version abortion abortions embryonic fetuses proquad lines ii zostavax varivax* **General Words:** cells insert unknown package version lines
**Topic 20**	*rash spots pics glands roseola lymph bumps bites swelling trunk nodes itchy swollen cheeks pic swell insect hives photo pictures itch visible angle mystery benadryl* **General Words:** choices round rash strange swelling legs

Table 7. Topics 21–30 from mothering.com using CRWT.

	Topic Words
**Topic 21**	*rotavirus rotateq shedding recipients rotovirus diarrhea ci rota placebo shed intussusception rotashield vaccinees contacts acquiring characterized poop subjects sheds smell viruses duration vomiting playgroup vomitting* **General Words:** recently viruses individuals transmission rotavirus susceptible
**Topic 22**	*hpv cervical cancer pap warts genital gardasil dysplasia paps smears cervix std condoms women abnormal smear strains cancers sexual sexually sex biopsy virgin high-risk folic* **General Words:** women caught cancer types boys united
**Topic 23**	*cbs nbc station cnn interview reporter radio segment news speaks msnbc channel katie fox suing researcher publicity piece blurb fired upcoming contaminants firing controversial pharma* **General Words:** local news pharma piece frequently feed
**Topic 24**	*insurance medicaid wic coverage company stamps wyeth medicare reminding reminder hmo kaiser denied fraud pocket applying ins pharmaceutical non-vaccinating nebraska fee notices pops covered charge* **General Words:** benefits company immunizations pharmaceutical covered insurance
**Topic 25**	*allergy allergies eczema egg dairy soy peanut lactose excema peanuts goat allergic allergist butter allergens patches intolerance cow eggs casein patch sensitivities cheese colostrum nuts* **General Words:** allergies factor injection allergic allergy nuts
**Topic 26**	*water filter bottled fluoride diapers drink cloth fruits flouride wipes veggies vinegar organic tap soda soap products toys rice drinks oils vegan vegetarian cleaning eats* **General Words:** water buy products city organic drink
**Topic 27**	*navy dependents military army dod overseas regulation japan assignment clearance commission reg commander command youth instruction duty civilian chaplain base screening tour spouse civilians tricare* **General Words:** decisions refuse members active physical official
**Topic 28**	*tylenol fever teething acetaminophen fevers temp pain antipyretics motrin thermometer paracetamol temperature ibuprofen aspirin relief medicate reducers liver migraines lethargic hydrated discomfort chew bath throats* **General Words:** sick high fever pain liver vaxxing
**Topic 29**	*smallpox cowpox vaccinia eradication london jenner quarantine eradicating inoculation isolation 1970s bleach clothing sores laundry bucket goal experts eradicate heat royal npr ring reconsider flu-like* **General Words:** vaccination linked smallpox experts dear campaign
**Topic 30**	*homeopathic nosodes homeopathy remedy nosode thuja diluted remedies homeopath homeopaths classical substance naturopath conventional constitutional alcohol cures homeopathics kit scam potency wave undo waves 2x* **General Words:** explain reference prevention homeopathic energy remedies

Table 8. Topics 31–40 from mothering.com using CRWT.

	Topic Words
**Topic 31**	*th2 th1 humoral adjuvants atopic alum cellular ige cytokine immunisation immunised response adjuvant function proteins mice immune inflammation endotoxin immunological inflammatory vol induce localized foreign* **General Words:** system immune response function pg foreign
**Topic 32**	*daptacel tripedia infanrix acthib pentacel combo pediarix singles combos lowest brands comvax adacel thermisol combined prohibit listing discontinued combinations sear product iu parts prescribing slow* **General Words:** safety product parts contained combined slow
**Topic 33**	*principal student examinations sincere genuine screenings beliefs district districts amended parental relation letter scripture amendment reversed nys sufficient scrutiny deemed agent immunizing superintendent practitioner person* **General Words:** person beliefs letter writing market student
**Topic 34**	*friendly non-vax tribal no-vax nfl mds boulder docs knowledgeable miles knowledgable referrals network md yulia heath area yahoo convert listens actively joined falls intolerances disturbed* **General Words:** area docs areas md published non-vax
**Topic 35**	*clotting oral vit prophylaxis bleeding onset intramuscular jaundice dis arch neonatal newborn bmj plasma maternity al orally pku traumatic gunk heel bruising newborns injectable synthetic* **General Words:** newborn wanting vit oral newborns al
**Topic 36**	*tb test sputum ppd xray x-ray tuberculin skin chest tuberculosis bcg positives positive semester testing latex ri hosp clinic scan dozen hook ordeal teaching ashamed* **General Words:** test testing positive skin clinic chest
**Topic 37**	*polio paralytic opv paralysis poliomyelitis salk poliovirus sv40 ipv ddt tumors sv-40 sabin nigeria counted aseptic tumor coxsackie seed contaminated detected injections acute predisposition susceptibility* **General Words:** polio permanent ipv injections acute opv
**Topic 38**	*vaers reportable mmwr report cases reports reporting assess notifiable deaths underreporting yikes reported tables download including psychiatric detail events limitations timeline regression searching blowing event* **General Words:** cases including figure reported report deaths
**Topic 39**	*cough coughing whooping whoop croup mucus hairs wc seal bouts spells bronchitis vita fits swab choking vomited lasted echinacea coughed inhaler spit noise tummy michelle* **General Words:** sound cough whooping coughing fits lasted
**Topic 40**	*anthrax troops gulf soldiers iraq war george washington deployed bullet reporters disturbing served voluntary tuesday sustained constitution rush tube appeal weapon mysterious stored unique grab* **General Words:** worse groups attack war unique washington

LDA Topics:

Table 9. Topics 1–10 from mothering.com using LDA.

	Topic Words
**Topic 1**	baby hep birth hospital test shot blood babies pregnant born tb pregnancy give time vit risk vitamin nurse newborn eye weeks mother told women make vax positive nursing work hepb
**Topic 2**	measles pox immunity chicken cp vax rubella mumps vaccine mmr kids child immune shingles disease chickenpox virus children exposed vaccinated vaxed case diseases time years people varicella natural age childhood
**Topic 3**	doctor ped child dr doc vax time doctors told medical office care find good asked visit family dd practice pediatrician baby insurance ds call back sign make give nurse vaxing
**Topic 4**	tetanus wound shot rabies vax vaccine dog years risk cat dogs time worry shots tig cut year animal vet people cats give good blood tetnus animals puncture wounds injury kids
**Topic 5**	pertussis kids sick cough child vaxed baby vax months children dd time immune whooping vaccinated healthy ds unvaxed home year worry daycare cold illness babies system weeks son disease day
**Topic 6**	people read article thread back thought good post time thing link news love parents lol show story today make find interesting put great stuff made yeah things big kids heard
**Topic 7**	vaccines mercury vaccine aluminum thimerosal cells ingredients human virus body fetal formaldehyde cell injected insert aborted tissue package blood amounts amount safe dna free brain found trace animal dose live
**Topic 8**	vitamin good food system milk immune body diet eat day water give time things foods natural vit health make oil healthy lot find gut taking formula back organic stuff work
**Topic 9**	vax kids feel child people dh decision time children family make vaccines research vaxing vaxed told vaccinate vaxes dd mom baby son things shots good parents ds thing made issue
**Topic 10**	religious exemption state beliefs religion school exemptions law letter child vaccination medical philosophical states ny church court god parent belief personal immunization health rights reasons practice legal laws catholic vaccines

Table 10. Topics 11–20 from mothering.com using LDA.

	Topic Words
**Topic 11**	vaccine cancer hpv vaccines health drug companies money cervical women fda government children cdc gardasil merck vax bill public years pharma people parents make dr safety company girls information control
**Topic 12**	polio vaccine disease people vax countries country virus diseases smallpox vaccines water travel world live cases opv children measles years vaccination vaccinated africa living time traveling india back clean hep
**Topic 13**	flu shot vaccine year vax people influenza sick virus h1n1 shots pregnant swine flumist work season years pandemic bird live time kids immune strain women military dh good risk make
**Topic 14**	school exemption form state child vax health religious exemptions required schools immunization law public private daycare medical sign children kids records states letter record preschool information care info find vaccination
**Topic 15**	vaccine cases disease children meningitis hib vaccination infection risk deaths reported pertussis bacteria cdc years age death number study rate vaccinated hepatitis infants infections year incidence immunity immune rates numbers
**Topic 16**	autism children study vaccines studies mmr child kids link autistic mercury research years problems disorders parents brain evidence sids son genetic found vaccine damage vaccinated read asthma article age disorder
**Topic 17**	fever reaction days time reactions child day dd doctor vaccine son ear back normal rash baby ds bad months told sick hours symptoms started shots pain infection shot seizures give
**Topic 18**	vax months dtap vaccines vaccine shots hib schedule mmr shot age vaxes time dose hep doses give child month years delayed prevnar research ds polio year pertussis dd delay selective
**Topic 19**	book read vaccine vaccines vax info research good information find dr diseases disease cdc books reading child links vaccinations link decision great doctor found time site lot questions forum start
**Topic 20**	people vaccines vaccine children disease make child medical vaccination point things diseases system time health vaccinate research parents good doctors work fact immune agree lot risk thing question person vaccinated

Topics on the second site

Tables 11–16 below list sixty topics obtained by the Contextual Random Walk Trap (CRWT) applied to the second site posts. As in the case of mothering.com, we present a flattened list of leaf topics, and ignore the hierarchy. Because second site has a much larger number of posts, the topics tend to be much more self explanatory and we have not added the general words. Tables 17-22 list the topics obtained by the LDA method, when the parameter for the number of topics, K, is set to 60. As in the case of mothering.com, for both topic modeling techniques, we use all the posts
comprising a thread as a single document. As noted in the main paper, Topic 1 in the CRWT method, and Topic 46 in the LDA method are related to exemptions.

The rest of the topics discussed in second site are highly diverse, and cover almost every aspect of parenting, along with contemporary societal and political issues, including: (i) Everyday Parenting and Family Life Issues, such as child birth, the right choice of diapers, cleaning and laundry, homework, putting kids to bed, car seats, pets and their care, hair cuts, recipes, nutrition, breastfeeding vs formula feeding, weaning, fevers and treatments, party planning, holidays, movies and entertainment, and activities for kids; and (ii) Socio-Political Issues, such as Religion and teachings, Creationism vs Evolution, Race Issues, Presidential Elections, LGBT rights, Marriage, Crime, Domestic Violence, Gun Violence, and Global Political Events. Even with such diversity, where the topic of exemption has a much smaller percentage share of the overall corpus volume, our automated tools were able to extract the underlying story graphs. Moreover, the story graphs matched those obtained from the mothering.com forum, where the exemption topic was much more dominant.

CRWT Topics:

Table 11. Topics 1–10 from secondary site using CRWT.

	Topic Words
**Topic 1**	*religious exemption beliefs exemptions belief belong supreme required philosophical union individual ny practices title northport sherr northport-east require contrary sincere ruling supp dist determined guardian sch ruled espouses professes genuine*
**Topic 2**	*vaccine vaccines health disease flu mercury study research immune medical vaccination system brain virus cases cancer risk blood thimerosal studies cdc diseases measles drug shot fda vaccinated effects cells vax*
**Topic 3**	*food milk eat water foods oil cup cheese sugar diet chicken organic eating drink add butter juice meat fruit cream salt rice bread veggies fresh dairy raw sauce corn soy*
**Topic 4**	*business team company income website products earn opportunity sales training selling parties monthly inventory product sell avon build commission receive marketing goals residual ameriplan fees prosperity gbg downline consultant success*
**Topic 5**	*church jesus christian bible religion christians catholic christ sin christianity faith roman traditional conservative religions heaven aimee muslims holy sins jewish homosexuality muslim teachings jews testament spirit churches catholics baptist*
**Topic 6**	*owner admin vaccinate educate informed fashioned choosing theinformed-parent poisoning autismmercurypoisoningandeverythingbetween childbirth educatebeforeyouvaccinate educators trackback doulas anti-mainstream animal dawn anti wner allaboutanimalsforanimallovers lovers angels america url openmindedmoms lifestyle entry icio momswhochoosenottovaccinate*
**Topic 7**	*north south california city texas county san ca carolina york florida ohio virginia tx va island michigan diego los angeles beach southern oklahoma dakota kansas louisiana mexico illinois fax minnesota*
**Topic 8**	*white black racist color race skin red racism blacks racial whites african americans native culture dark races yellow slavery hispanic asian racists mexican slurs purple pride flags indian mexicans indians*
**Topic 9**	*fever cough vomiting nose throat rash diarrhea headache nausea runny sore neck muscle swelling breathing coughing irritability redness dizziness chest fatigue erythema aches neuritis strep headaches congestion nasal abdominal tenderness*
**Topic 10**	*breastfeeding co-sleeping natural sahm diapering non-vaxing birthing cio attached baby-led lmt babywearing homeschooling selective pisces tree-hugging libertarian intact baby-wearing harrison christian- earth- non-circumsizing delayed hippie gentle non-vaxxing lovin vaxing tom-boy*

Table 12. Topics 11–20 from secondary site using CRWT.

	Topic Words
**Topic 11**	*obama mccain president bill vote palin bush republican senate political congress voted voting republicans democrats campaign election sisterhood clinton senator candidate relax presidential celebrating passed barack legislation liberal sarah governor*
**Topic 12**	*rape sexual abuse crime prison jail charges guilty charged crimes convicted criminal victim assault raped innocent neglect commit abused sexually penalty felony sentence charge committed sentenced rapist criminals accused offenders*
**Topic 13**	*wear clothes wearing shoes pants dress shirt jeans shirts socks pair shorts clothing wore wears dresses dressed boots flip skirts glasses flops makeup tight uniforms uniform skirt pairs t-shirts t-shirt*
**Topic 14**	*dog dogs cats cat pet foster pets kittens cockatiels tortoises chinchillas geckos kitty leopard vet pit breed puppy maverick indoor shelter kitties breeds spay owners orphaned neuter raccoon rabies puppies*
**Topic 15**	*clean laundry wash soap cleaning dishes washing kitchen detergent rinse dirty dryer dishwasher floors washer towels sink bleach carpet wipe load vacuum cleaner washed sheets mop cleaned borax sweep soaps*
**Topic 16**	*drugs smoke smoking pot alcohol marijuana prescription addiction cigarettes smoked smokers medications addicted weed tobacco cigarette smoker prescribed pharmacy addictive mj smokes pharmacist heroin addicts medicines cannabis over-the-counter prescriptions meth*
**Topic 17**	*vitamin vitamins acid supplements vit calcium minerals supplement acids iron probiotics zinc nutrients deficiency magnesium fatty stomach b12 amino cod d3 reflux enzymes digestive b6 omega mineral folic synthetic nutritional*
**Topic 18**	*diapers cloth diaper covers prefolds aio pocket pads disposable fleece fitted pockets wipes fitteds wool newborn sizes cotton hybrid disposables inserts dipes pul pins flannel waterproof ai2 prefold layer tampons*
**Topic 19**	*hair cut legs shampoo cuts shave nails cutting curly arms budget scissors haircut blonde clippers dye shaving conditioner salon thick scalp trim blond shaved shoulder comb razor lice haircuts chin*
**Topic 20**	*flapping repetitive movements stereotyped spinning stimming interests patterns objects rocking restricted stim preoccupation abnormal spin stims circles routines manifested twisting rituals intensity flaps encompassing mannerisms spins inflexible adherence humming flap*

Table 13. Topics 21–30 from secondary site using CRWT.

	Topic Words
**Topic 21**	*military war iraq army troops soldiers defend mission sustain govern afghanistan weapons baghdad solution base nuclear civilian wives soldier -george gulf saddam deployed forces iran marine vietnam veterans combat enlisted*
**Topic 22**	*key author concepts naturalnews articles david september october friday august november july journalist tuesday monday editor june wednesday thursday citizen writer gutierrez mike adams december march saturday ranger kirby staff*
**Topic 23**	*tenpenny placenta risks benefits sherri dvd glands mammary fear choices provide development ensure guidance genetic sells vaccines-the destiny fulfilled non-vaxed outweigh outweighed mongering predisposition lotus heights fearful benifits detach feared*
**Topic 24**	*court legal attorney filed lawyer file custody lawsuit lawyers complaint suit visitation courts defense lawsuits sue documents attorneys papers appeals filing appeal agreement bathing document ordered judges plaintiffs circuit sued*
**Topic 25**	*store grocery stores shopping shop cart walmart items sale wal-mart brand coupons groceries coupon dollar mall thrift secret carts sold item cheaper kroger brands shoppers shopper aisle fs cashier feedback*
**Topic 26**	*credit card bank account debt cards banks financial mortgage savings loans loan debit accounts economy deposit payments checking checks funds bailout retirement fannie deck freddie diamond spade crisis foreclosure mae*
**Topic 27**	*countries canada china australia germany europe africa france japan uk ireland usa sweden russia india republic spain denmark european britain switzerland norway saudi tab austria arabia asia korea netherlands western*
**Topic 28**	*homework spanking helping bedtime kissing academic pushing dessert activities nightmare benefit cuddling mum organized vaccinating sending spoiling village assignments assignment swat autym nico swats swatting extracurricular forgets swatted hw planner*
**Topic 29**	*rights laws civil constitution discrimination parental amendment privacy consent amended nov 1-guidelines vii constitutional violation sincerely equal protected movement violated violate freedoms enforce exercising erosion liberties violating protecting harassment protections*
**Topic 30**	*animals men murder treated gandhi -mohandas judged moral progress da -leonardo greatness nation vinci stuffed bloody dicks horny di compass humanely unfairly manly harshly furry imperative accessory*

Table 14. Topics 31–40 from secondary site using CRWT.

	Topic Words
**Topic 31**	*marriage gay threatens couples answered marry presents philosophy flying dropping homosexual chimney marriages questioned relationships gays heterosexual murmur lesbian same-sex maher bisexual homosexuals consenting unions marrying sexuality attracted lesbians polygamy*
**Topic 32**	*weight pounds lbs size gain gained double weigh inches tall oz height overweight obese skinny calf stomachs destined weighed weighs thin exercise gaining smaller gym percentile cardio inch larger pound*
**Topic 33**	*insurance cover medicaid coverage covered costs ssi employees employer medicare pays workers employers denied premiums expenses unemployment employee pre-existing uninsured eligible ins tricare deductible deductibles co-pays insured wages copays insurances*
**Topic 34**	*music song sing songs dance singing insane sings danced band monet angelou bird maya -angela cd player dancing lyrics rap guitar concert piano talent sang ballet dancer classical singer musical*
**Topic 35**	*parenting mainstream alternatives science art competitive sport attachment amp unconditional felipe tips mamas handmade practical earthy meandering tribal patricia naomi leticia rhymes style paths tenthings mia- kohn fringe alfie rocket*
**Topic 36**	*seat seats harness facing rear booster belt britax carseat rear-facing buckle belts safer rf stroller rides passenger convertible certified seatbelt installed latch regent ff endorsed graco crash carseats technician marathon*
**Topic 37**	*strong weak sympathetic tolerant striving aged compassionate tender depends someday george georgewashingtoncarver washington carver georgewashingtoncarve 3-day washngton orwell brave willed carlin strait*
**Topic 38**	*potty toilet trained pee poop bathroom accidents underwear wet accident peeing pooping bladder peed flush pooped poops pees undies restroom pull-ups constipated ec bm stall miralax flushing poopy pull-up pullups*
**Topic 39**	*depression risperdal suicide mood mg med prozac adderall zoloft bipolar abilify concerta ritalin clonidine xr antidepressants depressed swings suicidal strattera tenex dosage paxil lexapro depakote seroquel celexa focalin ppd antidepressant*
**Topic 40**	*theory evolution species humans theories conspiracy evolved universe apes creation dinosaurs birds fossil darwin evolutionary origin inexplicable bizarre replaced disappear instantly discovers creationism mammals ancestor bang reptiles evolve biology fossils*

Table 15. Topics 41–50 from camemom.com using CRWT.

	Topic Words
**Topic 41**	*gun guns hunting shoot shooting loaded hunt rifle wolves ammo hunters bb intruder rifles firearms ownership squirrels bullets wolf shootings handguns unloaded firearm shotgun stored handgun wildlife concealed nerf pistol*
**Topic 42**	*police officer officers arrest cops arrested cop ticket emergency traffic speeding tickets ambulance enforcement patrol firefighters incident warrant dec arrived paramedics cardiac arrests departments responders preparedness stephens allegedly highway rounding*
**Topic 43**	*sperm pill cycle condoms hormones bc uterus ovulation fertility pills condom hormone periods iud donor nfp method fertilized hormonal estrogen embryos cycles menstrual progesterone ovaries fertile implanted ivf ovary std*
**Topic 44**	*movie movies horror film theater netflix scene films scenes actors rated disturbing documentary hbo actor convention theaters theatre graphic comedy hollywood imprint audition comedies remake centipede comic hostel comics sequel*
**Topic 45**	*teeth dentist tooth brush dental fluoride brushing fillings toothpaste cavities amalgam decay dentists joint toothbrush flouride cavity loose amalgams braces brushed gums enamel filling compressions fluoridated brushes molars fluoridation grinding*
**Topic 46**	*phone cell phones text texting computers laptop texts verizon wireless desktop rang landline dial emergencies ringing laptops messaging texted prepaid ipods sprint distance pre-paid caller cellphone tvs doorbell voicemail confiscated*
**Topic 47**	*yam behold breastmilk power popeye momma weaning extended breastfed preemie search resources breastfeed warning offends engines lo rangers feature scarce digesting*
**Topic 48**	*christmas santa easter holiday merry celebrate holidays fairy bunny magic claus celebration xmas celebrated solstice yule birthdays seals hanukkah tales celebrates tale festival reindeer ornaments mithras magical celebrations rudolph bunnies*
**Topic 49**	*labor c-section delivery epidural pitocin vaginal induced cesarean contractions vbac section induction breech rupture uterine vaginally induce c-sections deliver cervix epi epidurals dilated distress forceps inductions preterm sections premature laboring*
**Topic 50**	*party wedding shower invited host invite bride guests packages tacky reception invitations weddings showers bridal groom invitation shipped guest decorations registry festive ceremony prizes etiquette bachelorette hostess gourmet preparation brides*

Table 16. Topics 51–60 from camemom.com using CRWT.

	Topic Words
**Topic 51**	*lord psalm isa joshua courageous discouraged formed tongue rises heritage prosper judgment womb psalms weapon condemn servants prayer praise amen wonderfully righteousness fearfully marvelous hellip niv terrified heal parts mighty*
**Topic 52**	*bags plastic bag trash garbage containers cans boxes bpa pack storage plastics empty douche reusable bisphenol seal packed mylar phthalates packing trailer lids polycarbonate punching canvas cardboard bins sealed packs*
**Topic 53**	*test tests testing iq score scores lab scored standardized tb accurate labs neurotypical cognitive performance extensive non-autistic plains beagle quiz ados paternity neg mailed pku pos rogue amnio snapped mensa*
**Topic 54**	*fire desire reality friendly greed sail wealth aimed raging sea distribute stephanie forest unconquerable anti-flag trainwrecks abandoning cave drill deborah mitchell environmentally fighters flames debris lightweight drills burned fires sergeant*
**Topic 55**	*door doors lock locked locks window windows opened gate alarms alarm opens opening locking unlock knob knocking cabinet garage closing unlocked keys slam curtains cabinets slammed blinds slamming deadbolt closes*
**Topic 56**	*seizures seizure eeg encephalopathy mri febrile activity grand mal ct persistent inconsolable bulging unusual scan high-pitched causation scans staring temporal eegs variable determining neurologists sufficient scheme sedated compatible conclusive consensus*
**Topic 57**	*driving driver truck drunk drivers license ford vehicle vehicles honda drives toyota dodge suv chevy trucks minivan lizards chrysler cab batty pickup gerald mileage ram semi taxi stumbling bonkers stumble*
**Topic 58**	*reader poster emotional intellectual responsible suffered communication liable deficiencies expressly comprehension psychological straight capacity challenged metaphors disclosed sarcasm satire presented masterdebaters priorities turmoil nuances rollercoaster instability prolific facilitated*
**Topic 59**	*cash ebay retailers motherhood hsn maternity refer priceline sutterfly dell overstock 1-800-contacts snapfish bryant purchases gap lane kohl paypal qvc sam pinch rebates deposited creek chico bradley fog on-line bebe*
**Topic 60**	*tax taxes property taxed gains bracket capital deductions irs eic refund 5k payers estate smallness deduction payroll expense taxpayers taxing returns deduct ssn excise taxation payer 4k refunds 3k taxable*

LDA Topics:

Table 17. Topics 1–10 from secondary site using LDA for K (# of topics)=60.

	Topic Words
**Topic 1**	black white people race racist word color english american racism language history person man speak racial skin culture blacks women hate words country flag called spanish whites races african point
**Topic 2**	things people feel time life make person good lot important work learn change sense feelings understand part experience love find ways world hard feeling thing mind thinking social point kind
**Topic 3**	play son kids things toys loves games time playing game year thing toy good fun lol great video likes tv day daughter watch make kid boys age love boy computer
**Topic 4**	live area state city county texas center local family california dr north south town florida people ca lived san york move place year moved community ny st west ohio pa
**Topic 5**	police case cps people death gun abuse man crime jail law person happened story guns kill prison court home killed life officer report woman charges time murder children cops back
**Topic 6**	home business free work team company join money website income make opportunity earn products time month start today people training information great visit working group support family check moms sign
**Topic 7**	food eat milk diet foods eating weight healthy organic good fat meat dairy free make sugar things gluten soy eats lot drink raw veggies day give fruit health meal cheese
**Topic 8**	school college kids schools students education high class grade year public work learning learn teachers classes student teacher teach teaching good home children math time years test reading level program
**Topic 9**	water oil natural skin vitamin good tea soap vinegar products body soda make day works great juice vitamins health yeast fluoride helps bottle liquid supplements probiotics bacteria oils baking give
**Topic 10**	buy store money shop shopping make stores back free sell grocery bought stuff items online price cash buying walmart people ebay sale find pay things order cost purchase site join

Table 18. Topics 11–20 from secondary site using LDA for K (# of topics)=60.

	Topic Words
**Topic 11**	house water clean room home put things stuff live cleaning kitchen lol snow laundry yard door living make pool year place garden floor cold small clothes food hot inside trash
**Topic 12**	vaccine disease vaccines children measles cases vaccination tetanus polio meningitis virus diseases immune years infection pertussis age hepatitis child vaccinated risk death bacteria immunity reported hib people deaths months symptoms
**Topic 13**	group baby love owner moms parenting admin lol mom natural ap mainstream fashioned cloth breastfeeding wife great mama check family good anti-mainstream home mommy attachment groups christian diapering join sahm
**Topic 14**	mom family told dad son friends time friend years mother daughter husband sister kids talk dh father brother back things wanted asked house good thing girl call year feel people
**Topic 15**	science world theory human people life earth years evidence real time post fact agree evolution humans question scientific freedom true society point truth nature disclaimer created history change annoyance interesting
**Topic 16**	posted mobile lol love good pretty awesome group yeah lot quoting mod years toddler back smell haven spouse preschool hope cunt weird check shit glad fucking fb ago great
**Topic 17**	drug fda health drugs companies company industry products safety report public food owner million group product information agency pharmaceutical year percent doctors medical market consumers merck administration federal press including
**Topic 18**	christmas year party birthday gift happy day kids family gifts santa fun wedding holiday cake love candy lol time halloween give tree holidays make easter parties celebrate great big people
**Topic 19**	vaccines vax shots child shot vaccinate vaccine research doctor dr son children vaccinated baby vaccinations give good kids months make time decision group told find sick healthy read daughter info
**Topic 20**	school teacher son child iep class special year kids teachers district good meeting home day ed classroom told time schools bus make work education call grade services program kindergarten aide

Table 19. Topics 21–30 from secondary site using LDA for K=60.

	Topic Words
**Topic 21**	son autism speech child therapy dx diagnosis months things time diagnosed early good age started spectrum school ot therapist daughter words issues services aba doctor lot year developmental social luck
**Topic 22**	group post posts people debate thread agree join read cm groups back time members im rules forum member lol ellie drama posted place friends admin reply feel pm posting make
**Topic 23**	baby milk breastfeeding formula nursing months babies breast nurse mother bf feeding bottle child breastmilk feed breastfeed mom breastfed time infant yam moms fed give pump son mothers year good
**Topic 24**	mercury toxic thimerosal vaccines aluminum children chemicals exposure vitamin lead body levels human cells found safe effects blood chemical poisoning ingredients products toxicity brain environmental system damage toxins heavy metals
**Topic 25**	dog animals dogs animal cat pet cats pets people time vet murder home love fish puppy good put house care treated kitty kill wild chickens breed humans owner live years
**Topic 26**	hair wear clothes lol dress wearing picture shoes pictures cute make love long girls shirt beautiful size daughter girl color put pretty pics back pants big pic cut time good
**Topic 27**	son sensory time teeth things thing mouth good hands head put back hand dentist kids issues make ot hair stop started luck cut touch child work hold helps daughter give
**Topic 28**	life smoke smoking people drink drugs drug alcohol depends drinking aged weak young strong compassionate tender sympathetic striving tolerant pot someday marijuana drunk coffee addiction georgewashingtoncarver quit time legal make
**Topic 29**	autism co-admin mom group awareness admin pdd proud love great asperger owner good lol chris happy special today hope moms hugs kat dawn heids day awesome lynn autumn wonderful son
**Topic 30**	obama military president vote war country bush mccain political bill party palin government iraq support american people america republican news years israel voted army soldiers voting freedom americans united office

Table 20. Topics 31–40 from secondary site using LDA for K=60.

	Topic Words
**Topic 31**	fucking follow choice diapering cloth choose learn give lol blue baby queen girl jean prettiest body son life parenting thinking world today totally created love hand solved level exist problems
**Topic 32**	watch show movie love tv movies watching song music video shows watched favorite time people good lol loved thought heard remember names story read man night episode guy listen great
**Topic 33**	meds son adhd medication doctor anxiety dr good depression taking side work give time years issues disorder started med effects put takes behavior child day bipolar luck problems school year
**Topic 34**	vaccines autism vaccine children parents dr health cdc study mercury research public medical thimerosal government safety studies vaccination vaccinate link national mmr evidence vaccinations case group years childhood disease committee
**Topic 35**	lol breathe life understand dismiss people hearts god free im make ellie love cancer true find head girl hands disney good contend roberts tickers fewer gods hate debate -stephen agree
**Topic 36**	feel love life time day hope god family things good heart hard son hugs happy make back mom feeling hear give great child children today thing wonderful world cry sad
**Topic 37**	read book books reading site find information link page info post check good great email website internet found send online list write facebook computer free questions make pictures click links
**Topic 38**	son autism kids autistic daughter mom year child children years spectrum asperger group asd dx aspergers nt boys aspie special diagnosed boy high functioning love day yr moms understand life
**Topic 39**	baby birth hospital born labor weeks pregnancy babies women home time pregnant doctor midwife c-section pain blood mother delivery natural told hours due ob risk wanted medical doctors cord births
**Topic 40**	people government country system world countries money america tax american americans illegal states state make care free million problem years population canada economy taxes live society poor work united citizens

Table 21. Topics 41–50 from secondary site using LDA for K=60.

	Topic Words
**Topic 41**	potty diapers diaper cloth time bathroom toilet training pee trained poop make put day good wash pants son started wet months work underwear big pull shower clean thing dry made
**Topic 42**	baby pregnant abortion child life pregnancy women woman birth choice make mother babies control adoption born decision body support agree children give feel abortions rights fetus person die choose sex
**Topic 43**	people make person feel wrong things thing agree point understand bad time stupid opinion care shit makes fact post made talking good call word woman ass put read matter hate
**Topic 44**	flu vaccine health swine virus people vaccines h1n1 shot gardasil hpv influenza vaccination cancer year cdc disease pandemic deaths cervical women cases public shots girls vaccinate reported adverse children dr
**Topic 45**	god people religion church christian bible jesus christians religious faith beliefs christ life good make love christianity sin belief things catholic wrong hell person heaven man true agree understand word
**Topic 46**	state law religious court rights legal exemption states laws child school public health form sign medical exemptions parents required letter children federal parent act beliefs case immunization consent records information
**Topic 47**	study cancer disease health brain research body risk studies found blood researchers levels system cells patients percent people immune medical treatment dr effects heart high medicine results university diabetes increase
**Topic 48**	sex women men gay sexual girls woman man boys girl people male penis porn female make gender boy feel rape circumcision body thing young wrong agree choice sexually naked find
**Topic 49**	husband marriage married love man woman relationship children wife life years men women divorce make proud care home work person church time good dh happy family agree spouse marry feel
**Topic 50**	money pay job people work make paid food paying income time afford jobs back family give years live credit working welfare month home year bills good taxes tax support hard

Table 22. Topics 51–60 from secondary site using LDA for K=60.

	Topic Words
**Topic 51**	sick doctor fever ear pain back test infection days cold dr time blood antibiotics son infections bad give symptoms cough day hope good told months weeks thing doc er allergies
**Topic 52**	time day home work kids week days hours back feel good house school dh stay things husband mom working make year night lol job hour long today times care weeks
**Topic 53**	sleep bed night room time sleeping baby asleep put back wake months hours crib son nap cry day slept crying fall sleeps bedtime morning started minutes nights times hour wakes
**Topic 54**	insurance care health medical pay services doctor state medicaid cover doctors hospital office healthcare cost plan call coverage covered bill patient treatment ssi private work find patients month company program
**Topic 55**	autism children child autistic spectrum disorder parents disorders research families asd diagnosed special treatment cure support diagnosis social syndrome therapy dan family asperger people information group symptoms jenny awareness son
**Topic 56**	child kids children parents kid parent age mom mother year daughter home young care agree family adult make parenting son older adults school allowed teach wrong girl bad moms feel
**Topic 57**	time behavior things child son hit good back thing bad times told stop discipline understand spanking control give room hard hitting work screaming kids talk hurt upset feel calm put
**Topic 58**	make cup add cheese chicken butter cream cook bread recipe chocolate sugar good water eat salt ice made sauce beans minutes put love mix cooking eggs cups fruit hot fresh
**Topic 59**	lol funny shit love ass lmao fun hell yeah damn thought fuck back fucking time good man ya bitch thing day post laugh hate head life omg pretty call bad
**Topic 60**	car seat phone back door driving drive bus people time walk ride front put call cell safety safe driver road cars run park leave seats minutes stop street accident walking

Edit

BLOG AT WORDPRESS.COM.

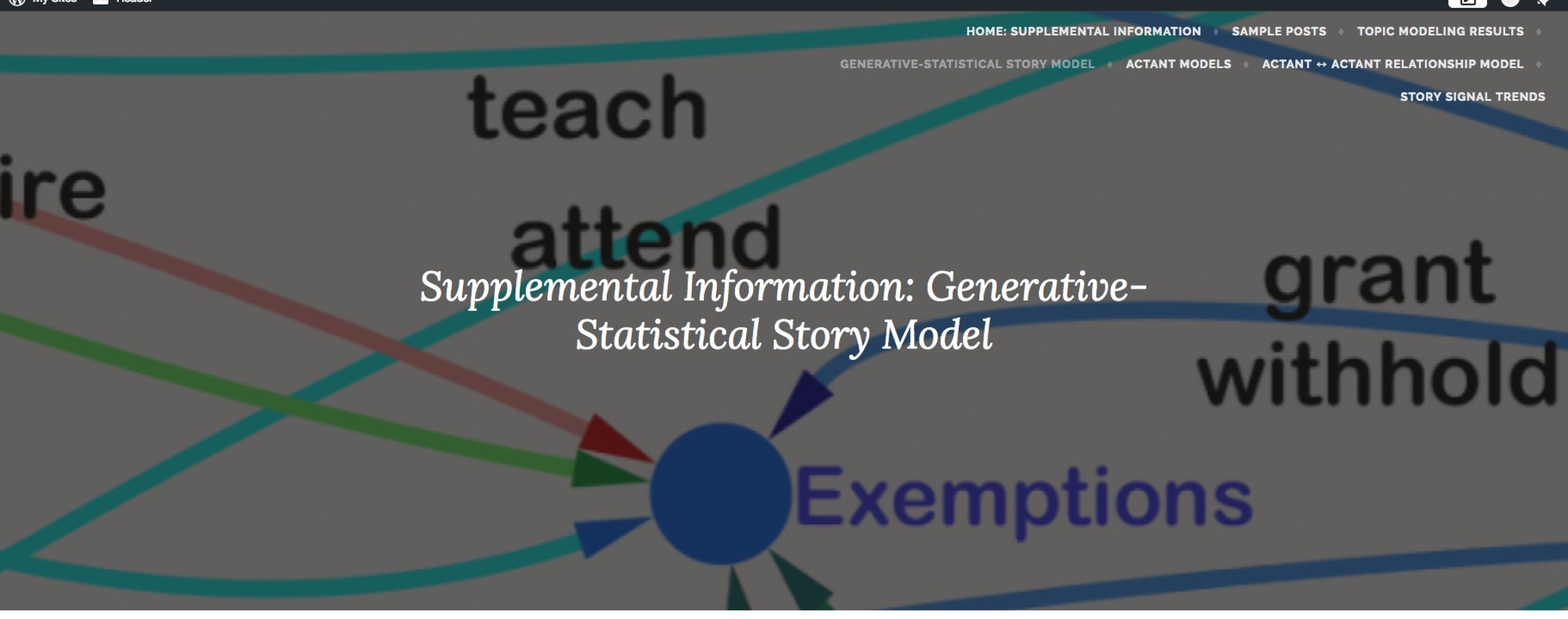

Supplemental Information: Generative-Statistical Story Model

We present a generative statistical-mechanical network model, in which actants (actors and objects) are the nodes, and the relationships between pairs of actants are the edges. These edges are labeled with the nature of the observed relationships (e.g., based on actions), the context of the relationships, and their likelihoods. Note that this approach is not an attempt to redefine and build a semantic network (in the usual sense), or an entity relationship network, such as Google's Knowledge Graph. In such networks, the actant attributes and relationships are usually fixed and are chosen from a set of pre-defined attribute lists. Thus "Apple" is a corporate entity (hence a node) and "ApplePay" is a mobile phone product entity (another node), and they are connected by the relationship of "owned or operated by", which again is a pre-specified category. Similarly, a movie actor is connected by edges to movies she has acted in, and each such edge could be qualified by a number of attributes, such as the type of character role she has played in that part.

Our networks, for example those illustrated in Fig. 3 of the main paper, are primarily aimed at capturing actants and their relationships that emerge under specific circumstances and situations, and that are driven by the necessity to build strategies to deal with such exigencies. Consequently, our networks are particularly suited for representing story and narrative dynamics, where the overarching structure does not vary much, but the specific instances of the actants, their roles, and their relationships vary significantly based on the circumstances. For example, under most circumstances, personal beliefs and beliefs that are based on traditional religions would be at odds with each other, and are separate actants. However, in the context of the exemption seeking behavior, both can play the same role. Since the laws allow both kinds of beliefs as reasons for exemption, both belief complexes can be grouped under the same actant category. This type of grouping is well established in structuralist narrative theory. A crucial observation is that this particular coalition of separate actants happened because of a particular context. Given the combinatorially explosive numbers of potential groupings of all actants, it is just not practical to create and populate an a priori network (such as a traditional knowledge graph) that captures all such potential groupings and that would cover all potential contexts. Similarly, the dominant societal relationship between educational institutions and children is one of learning and teaching; however, for the majority of the exemption seeking parents, the schools represent an authority figure with considerable power over the health outcomes of their children. Of course, the most critical reversal of roles in our case is between VPDs and vaccines: Vaccines pose a threat, not VPDs, to children's health. Again, it is combinatorially prohibitive to build networks that have all possible relationship models built-in.

AL INFORMATION · SAMPLE POSTS · TOPIC MODELING RESULTS · ACTANT MODELS · ACTANT ↔ ACTANT RELATIONSHIP MODEL · STORY SIGNAL TRENDS

A much more computationally feasible approach to modeling story dynamics is to recognize and then capture the underlying invariant structure, allowing the data to subsequently fill in the particulars. In this paper, the invariants are the types of pair-wise relationships that one expects in a typical storytelling environment and the types of typical actions the actants take. Almost all stories are based on such relationships (and expressed via the associated verbs) but the specific instances of actants, and their choice of relationships vary. Since such pair-wise relationships, especially those based on actions/verbs, can be highly context specific, instead of defining a single probabilistic network, where pairs of actants are connected via multiple relationships/edges each corresponding to a particular context, it is more convenient to define a network for each context. Formally, a particular narrative dynamics is characterized by an underlying set of *r* relationships:

$$\mathcal{R} = \{R_1, R_2, \cdots, \check{R}_r\},$$

and *k* contexts:

$$\mathcal{C} = \{\check{C}_1, C_2, \cdots, \check{C}_r\}$$

Each R_i is a binary relationship of the type shown in Table 2. A context C_i is a hidden parameter, or, to a borrow a concept from physics, the 'phase' of the underlying system
that defines the particular environment in which actants operate in. The phase expresses itself in the particular distributions of the relationships among the actants, and is captured by a labeled and weighted network G_{Ci} (V_{Ci} ;E_{Ci}). Here, V_{Ci} = {A_1, A_2, …,A_n where each A_j is an actant, and has associated with it a context specific probability or weight p_{Ci} (A_j) that determines the actant's likelihood of participating in the given context. The edge set E_{Ci} consists of m_{Ci} ordered pairs $e_{(Ci;j)}$ = (A_{j1} ;A_{j2}), where each such pair is labeled with a distribution over the relationship set

$$\mathcal{R},\ D_{(C_i,j)}(\mathcal{R})$$

One can consider the most simple case of a delta function distribution, and say that edge $e_{(Ci;j)}$ is a relationship R_{jCi} along with probability p_{jCi} that determines the probability with which the binary relationship is chosen. Thus, if the binary relationship is (employ, ignore), then with probability p_{jC_i} it is 'employ' and with probability (1 − p_{jC_i}) it is 'ignore'.

Given such a model, to generate a social media post or a story fragment, a user first picks a context C_i and then samples the network G_{Ci} (V_{Ci} ;E_{Ci}). That is, the user draws a set of actants according to the node distributions, p_{Ci} (A_j)'s. Then the user draws for relationships from among the associated distributions $D_{(Ci;j)}$(R). The user then composes the post according to these outcomes, by choosing the proper words, in particular nouns for the actants, and the associated verb phrases for the relationships.

Learning the Model From Data
In a social media corpus, the underlying probabilistic model including the contexts, C, relationships, R, primary actants, A_i, as well as the contextual story networks G_{Ci} (V_{Ci} ;E_{Ci}) are hidden. What we have instead are the samples drawn from the hidden statistical mechanical model. In order to estimate the parameters of the hidden model, we have to rely on the frequencies of verbs, nouns, and other structures in the posts that correlate with the model parameters. In this paper we take a frequentist statistics approach, where we estimate probabilities based on data and then use hard thresholding to make inferences, and leave a Bayesian approach for future work. Since nouns and verb phrases are the main structural elements used in posts to express the underlying story networks, we use an NLP method to process the posts and extract the parsing tree structure. We next follow the steps outlined in the main body of the paper. Here we provide some insights into the statistical underpinnings of the computational steps. Except for the generation of Tables 1 and 2, which needed some supervision to provide classes and labels, the rest of the steps are all automated.

(i) *Actants*: We list the nouns (subjects and objects) in order of their frequencies in the corpus and use a threshold to get the top nouns. Next we cluster the nouns by the similarities of the relationships (verbs) they have with each other. We build a network, where the nodes are the nouns and an edge connecting two actants is labeled with a distribution over the verbs connecting them in sentences. Similarities in these distributions are then used to cluster the nouns into different communities. Thus, for example, "belief" and "religion" have very similar distributions of verbs with other nouns, and get clustered together. We then used a supervised check of the groupings to
remove noise and merge some of the clusters together to get the actants listed in Table 1.

(ii) *Contexts*: As our model suggests, the primary expression of a context lies in the distribution of relationships among the actants. Two contexts are distinct if the relationship distributions are different enough. Since we are using actions as our primary relationship category, different contexts will lead to different distributions of the verbs found in each context. A rigorous maximum likelihood approach would be to partition the posts/sentences into all possible groups, and then determine a partitioning (hence a set of contexts) that maximizes a likelihood function of being generated by corresponding models. Clearly, this is not feasible given the millions of posts we consider. Consequently, we decided to take a heuristic approach to this search, and use our knowledge that contexts are usually defined by a grouping of the actants. To keep the algorithm computationally feasible, we defined a context to be all sentences that involve two actants A_i and A_j , and then considered all possible such pairs. As explained in the main paper, to avoid the bias of usage of verbs in a language, we used the KLD measure to rank verbs in each context. Again we use a threshold of the top 10 verbs to characterize each context.

(iii) *Relationships*: For each context, we use the verbs found in step (ii) and determine the actants that they qualify. Using Table 2, we then map the verbs to the corresponding relationships. Next we use a thresholding to specify the parameters p_{jCi}. For example, if for a pair we find that fraction x of the relationships is "employ" and fraction (1 − x) is "ignore" then we label it as "employ" if x > 0.9. If 0.1 < x < 0.9 then we do not resolve it, and label the edge as "employ/ignore". This thresholding leads to the networks shown in Fig. 3 of the main paper.

In summary, we have taken a frequentist approach to learn our models. The related counts and thresholds attest to the significance of the results.

Edit

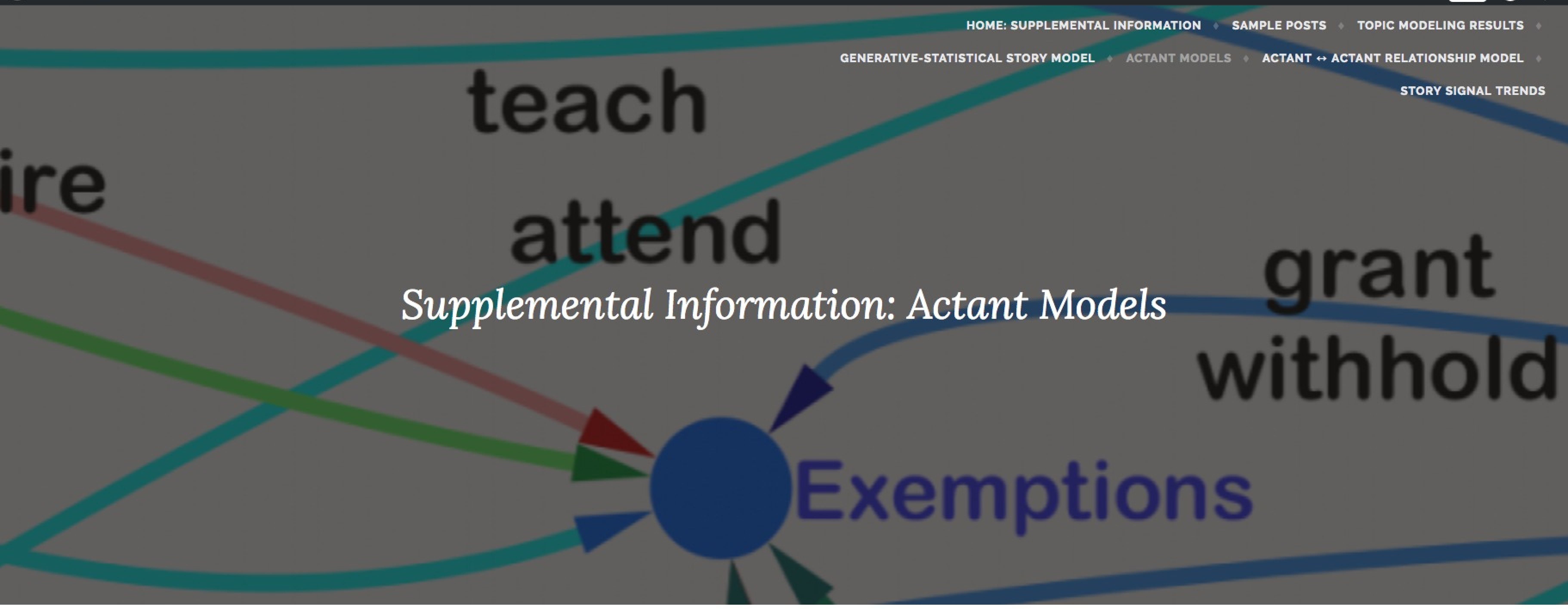

Supplemental Information: Actant Models

Actant Models

As described in the main paper, we used *actant* to refer to a set of entities that serve the same or similar purpose in the decision making ecosystem. We captured each actant by an associated set of words that most commonly referred to that group, and discovered three main actant categories: individual actants, comprised of parents, children, and medical professionals; corporate actants, comprised of government institutions, religious institutions, schools and pharmaceutical companies; and objects, comprised of vaccines, exemptions, VPDs, and adverse effects. The words associated with an actant consist of both synonyms for the actant and entities that have the actant as a super-category. For example, the actant "government" includes the colloquial synonym "the Feds" as well as the government institution, the "the CDC" where government is the super-category for CDC.

**Entities (Nodes)**	**Associated Word Set**
Individual Actants	
· Parents	parents, parent, i, we, us, you
· Children	child, kid, kids, children, daughter, daughters, son, sons, toddler, toddlers, kiddo, boy, d[ear]d[aughter], d[ear]s[on]
· Medical Professionals	doctor, doctors, pediatrician, pediatricians, nurse, nurses, ped, md, dr
Corporate Actants	
· Government	government, cdc, federal, feds, center for disease control, officials, politician, official, law
· Religious Institutions	faith, religion, pastor, pastors, parish, parishes, church, churches, congregation, congregations, clergy
· Schools	teacher, teachers, preschools, preschool, school, schools, class, daycare, daycares, classes
· Pharmaceutical Companies	pharma, big pharma, company, companies
Objects	
· Vaccines	vaccines, vax, vaccine, vaccination, vaccinations, shots, shot, vaxed, unvax, unvaxed, nonvaxed, vaccinate, vaccinated, vaxes, vaxing, vaccinating, substances, ingredients
· Exemptions	exemption, exempt
· VPDs	varicella, chickenpox, flu, whooping cough, tetanus, pertussis, hepatitis, polio, mumps, measles, diphtheria
· Adverse Effects	autism, autistic, fever, fevers, reaction, reactions, infection, infections, inflammation, inflammations, pain, pains, bleeding, bruising, diarrhea, diarrhoea

Edit

CREATE A FREE WEBSITE OR BLOG AT WORDPRESS.COM.

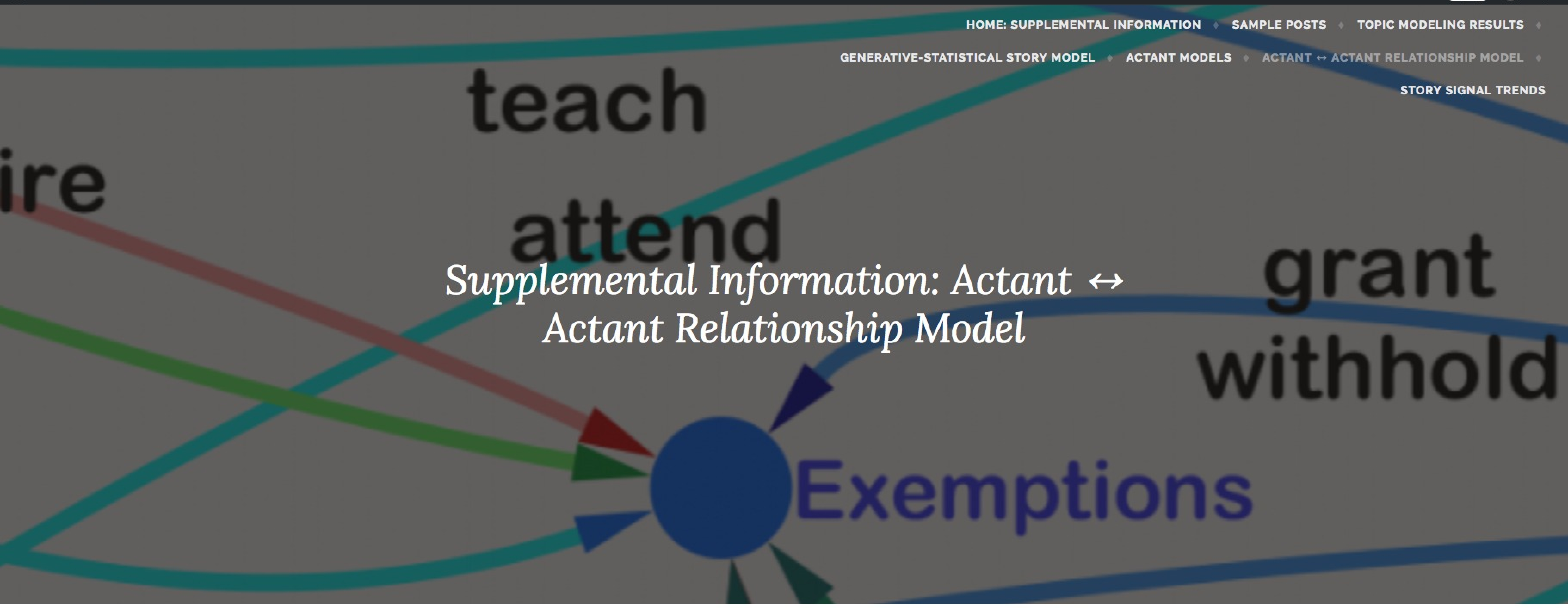

Supplemental Information: Actant ↔ Actant Relationship Model

Actant ↔ Actant Relationship Model
Recognizing that different verbs may capture the same type of relationship between actants, we grouped verbs into relationship categories, just as we grouped nouns into actant categories. Taking a cue from narrative theory, we classified these relationships according to a series of binary oppositions between verbs, with highly ranked synonyms grouped together with their highly ranked antonyms. We then devised labels for these groups of oppositional verbs.

HOME: SUPPLEMENTAL INFORMATION · SAMPLE POSTS · TOPIC MODELING RESULTS · GENERATIVE-STATISTICAL STORY MODEL · ACTANT MODELS · ACTANT ↔ ACTANT RELATIONSHIP MODEL · STORY SIGNAL TRENDS

**Relationships (Edges)**	**Associated Word Set**
Between Individuals or Corporate Actants	
· Require or Resist	force, require, need, follow, mandate
· Advise or Question	recommend, tell, said, object, ask, learn, teach
· Protect or Threaten	protect, injure, damage
· Employ or Ignore	use, submit, ignore
· Accept or Reject	vaccinate, unvaccinate, vax, unvax, receive, have, had, get, inject, exclude, allow, exempt, believe, receive, request, deny, accept
· Attend or Avoid	enter, enroll, attend, go, send, homeschool
Between Individual and/or Corporate Actants and Objects OR between Objects	
· Seek or Aver	seek, file, sign, claim, submit, need, exercise, lie, claim
· Grant or Withold	accept, approve, get, abuse, grant, oppose, deny
· Protect or Threaten	protect, injure, damage
· Cause or Not Cause	expose, get, contract, cause, develop,<br><br>suffer, die, vomit, diagnose

Edit

CREATE A FREE WEBSITE OR BLOG AT WORDPRESS.COM.

Supplemental Information: Story Signal Trends

As explained in the main body of the paper, for every month, we calculated two metrics, story pervasiveness and story persistence. Pervasiveness is calculated as UE_j = (*The number of new users* in the jth month *who committed a post with the word "exemption" in it*); and U_j = (the number of new users in the jth month). Persistence is calculated as PE_j = (*The number of new posts* (every month) *with the word "exemption" in it*); and P_j = (# of new posts in the jth month). In order to smooth out the expected statistical variations, we tracked the cumulative fraction quantities, $\frac{\sum_{k=1}^{j}\mathrm{UE}_k}{\sum_{k=1}^{j}U_k}$ and $\frac{\sum_{k=1}^{j}\mathrm{PE}_k}{\sum_{k=1}^{j}P_k}$. We presented the plot for the cumulative fraction of users who posted on exemption in Fig. 2 of the main paper. Fig 1 presents the cumulative fraction of exemption-related posts; again, we see that approximately 5% of the posts on mothering.com and approximately 0.35% of the posts on secondary site are persistently on the topic of exemptions. In order to further show that both pervasiveness and persistence are not bursty, and that the activity levels are significant over more than five years, we also provide the following plots: (i) The fraction of new exemption-related posts, $\frac{\mathrm{PE}_j}{P_j}$, plotted as a function of j (see Fig. 2), (ii) The sum of the fractions of new exemption-interested users, $\sum_{k=1}^{j}\left(\frac{\mathrm{UE}_k}{U_k}\right)$, plotted as a function of j (see Fig. 3) and (iii) The total number of exemptions-related users i.e., $\sum_{k=1}^{j}\mathrm{UE}_k$, plotted as a function of j (see Fig. 4).

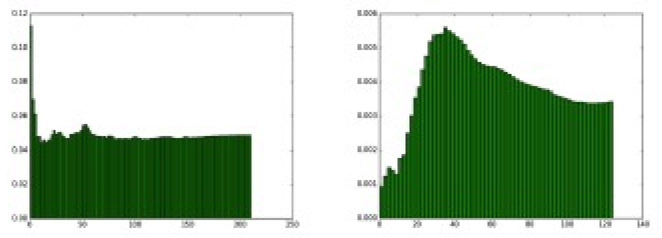

Figure 1. Left: mothering.com (≈5%); **Right:** secondary site (≈0.35%). Cumulative fraction of new **"exemption"-related posts**, i.e. $\frac{\sum_{k=1}^{j}\mathrm{PE}_k}{\sum_{k=1}^{j}P_k}$, **(Y-axis)** as a function of the number of months (j) **(X-axis**, scaled by a factor of 2). These plots show the persistent and endemic nature of the "Exemption" issue: The fraction of new posts every month, with the word "Exemption" in them, remained almost constant throughout the duration (more than 9 successive yrs. for mothering.com and more than 5 successive yrs. for secondary site).

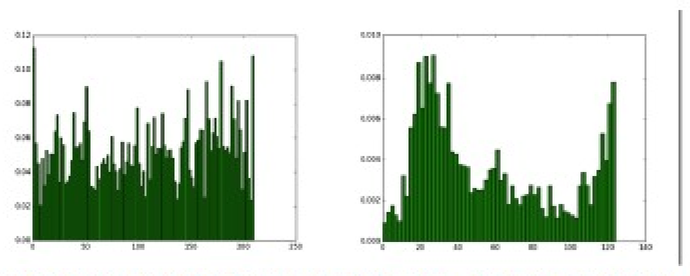

Figure 2. Left: mothering.com; **Right:** secondary site. Fraction of **new "exemption"-related posts** per month, i.e. $\frac{\mathrm{PE}_j}{P_j}$, **(Y-axis)** as a function of the number of months (j) **(X-axis**, scaled by a factor of 2). Both the ratios show a tight distribution around their respective means: ≈5% for mothering.com and ≈0.35% for secondary site.

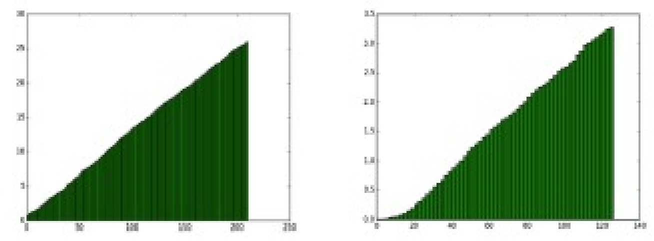

Figure 3. Left: mothering.com; **Right:** secondary site. Sum of fractions of **new "exemption"-related users** per month, i.e. $\sum_{k=1}^{j}\left(\frac{\mathrm{UE}_k}{U_k}\right)$, **(Y-axis)** as a function of the number of months (j) **(X-axis**, scaled by a factor of 2). The almost linearity of these plots show that the fraction of exempted-related users joining every month (i.e., $\left(\frac{\mathrm{UE}_k}{U_k}\right)$) remained almost constant over the observation duration.

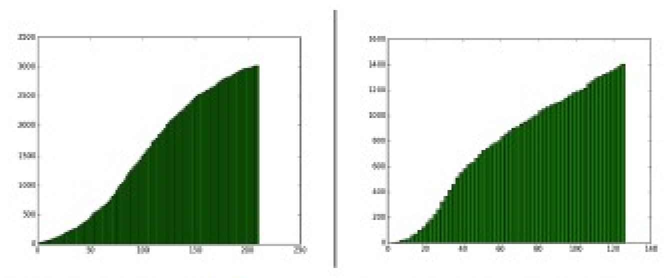

Figure 4. Left: mothering.com; **Right:** secondary site. Total number of **"exemption"-related users**, i.e. $\sum_{k=1}^{j}\mathrm{UE}_k$, **(Y-axis)** as a function of the number of months (j) **(X-axis**, scaled by a factor of 2). Since the fraction of new exemption-interested users remains almost constant while the total number of new users joining the forums decrease, one witnesses a leveling off of the plots.

Edit
